# Discovery of Novel Leptospirosis Vaccine Candidates Using Reverse and Structural Vaccinology

**DOI:** 10.3389/fimmu.2017.00463

**Published:** 2017-04-27

**Authors:** André Alex Grassmann, Frederico Schmitt Kremer, Júlia Cougo dos Santos, Jéssica Dias Souza, Luciano da Silva Pinto, Alan John Alexander McBride

**Affiliations:** ^1^Biotechnology Unit, Technological Development Center, Federal University of Pelotas, Pelotas, Rio Grande do Sul, Brazil; ^2^Gonçalo Moniz Institute, Oswaldo Cruz Foundation, Ministry of Health, Salvador, Bahia, Brazil

**Keywords:** *Leptospira interrogans*, outer membrane protein, epitope prediction, bioinformatics, transport proteins, structural modeling, genome mining, diderm bacteria

## Abstract

*Leptospira* spp. are diderm (two membranes) bacteria that infect mammals causing leptospirosis, a public health problem with global implications. Thousands of people die every year due to leptospirosis, especially in developing countries with tropical climates. Prophylaxis is difficult due to multiple factors, including the large number of asymptomatic hosts that transmit the bacteria, poor sanitation, increasing numbers of slum dwellers, and the lack of an effective vaccine. Several leptospiral recombinant antigens were evaluated as a replacement for the inactivated (bacterin) vaccine; however, success has been limited. A prospective vaccine candidate is likely to be a surface-related protein that can stimulate the host immune response to clear leptospires from blood and organs. In this study, a comprehensive bioinformatics approach based on reverse and structural vaccinology was applied toward the discovery of novel leptospiral vaccine candidates. The *Leptospira interrogans* serovar Copenhageni strain L1-130 genome was mined *in silico* for the enhanced identification of conserved β-barrel (βb) transmembrane proteins and outer membrane (OM) lipoproteins. Orthologs of the prospective vaccine candidates were screened in the genomes of 20 additional *Leptospira* spp. Three-dimensional structural models, with a high degree of confidence, were created for each of the surface-exposed proteins. Major histocompatibility complex II (MHC-II) epitopes were identified, and their locations were mapped on the structural models. A total of 18 βb transmembrane proteins and 8 OM lipoproteins were identified. These proteins were conserved among the pathogenic *Leptospira* spp. and were predicted to have epitopes for several variants of MHC-II receptors. A structural and functional analysis of the sequence of these surface proteins demonstrated that most βb transmembrane proteins seem to be TonB-dependent receptors associated with transportation. Other proteins identified included, e.g., TolC efflux pump proteins, a BamA-like OM component of the βb transmembrane protein assembly machinery, and the LptD-like LPS assembly protein. The structural mapping of the immunodominant epitopes identified the location of conserved, surface-exposed, immunogenic regions for each vaccine candidate. The proteins identified in this study are currently being evaluated for experimental evidence for their involvement in virulence, disease pathogenesis, and physiology, in addition to vaccine development.

## Introduction

Leptospirosis is a zoonosis caused by spirochetes belonging to the *Leptospira* genus. More than 250 antigenically distinct serovars have been described for 15 infectious *Leptospira* spp. (10 pathogenic and 5 intermediate species) to date (1, 2). Leptospirosis is a neglected tropical disease with an estimated incidence of over one million severe cases in humans, resulting in ~60,000 fatalities (3). Furthermore, the disease has a major impact on the health of agricultural and companion animals, with serious economic consequences (4). Recombinant vaccine development is a major research focus because of the lack of effective control measures. Classical immunization strategies based on whole-cell, inactivated leptospires (bacterins), or cell wall components have been well documented (5). However, while bacterin vaccines are highly efficacious, they cause serious adverse reactions and confer short-term immunity that is restricted to the serovars used in the bacterin preparation (6).

Several research groups have used the classical approach for the identification of protein targets for use in recombinant vaccines with mixed results, reviewed in Ref. (7). The most promising targets to date are the leptospiral immunoglobulin-like (Lig) proteins. We recently showed that a LigB-based subunit vaccine protected hamsters against leptospirosis and induced sterile immunity (8). While these results will need to be confirmed by other research groups, protection conferred by LigA, with reports of up to 100% efficacy in the hamster model, has been consistently reproduced by different groups throughout the world. However, sterile immunity was not evident in LigA-vaccinated survivors (9–11). In addition, *ligA* is present in only three pathogenic *Leptospira* spp. (12), further limiting its ability to induce cross-protective immunity. Of note, LipL32, the immunodominant leptospiral lipoprotein, was extensively investigated as a vaccine candidate using different strategies (e.g., subunit, DNA vaccine, BCG, and adenovirus constructs) with inconclusive results; efficacy ranged from 12 to 87% (13–17). Another putative lipoprotein, LemA, was identified using reverse vaccinology (RV) and induced partial protection using a prime-boost strategy (18, 19). Finally, the putative outer membrane protein (OMP) OmpL37, perhaps one of the most promising antigens recently characterized, was not protective against lethal disease in the hamster model (20). The current status of leptospiral vaccine development shows that there is an unmet need for the discovery of new vaccine candidates and that further success will require reevaluation of the *Leptospira* genome (21).

The RV approach was first applied almost two decades ago and led to the discovery of protective vaccine candidates for several bacterial diseases (22). The best example is the recently licensed vaccine against meningococcal disease caused by *Neisseria meningitidis* serogroup B; the protein components of the vaccine were discovered by RV (23). However, while there are some examples of its partial application toward target discovery in the field of leptospirosis (18, 19), it has not been successfully implemented (24), reviewed in Ref. (25). RV targets are generally surface-related proteins that are recognized by the host immune system, thereby eliminating the bacteria and preventing disease. Ideally, these targets should play an important role during pathogenesis, increasing vaccine efficacy. In diderm bacteria, such as *Leptospira* spp. and Gram-negative bacteria, β-barrel transmembrane proteins (βb-OMPs) and some outer membrane (OM) lipid-anchored proteins (lipoproteins) are the only types of proteins that are surface exposed (26, 27). Proteins with a transmembrane α-helix (TMH) structure tend to be localized to the inner membrane and are rarely found in the OM, e.g., the Wza translocon for capsular polysaccharides in *Escherichia coli* (28). Even though various βb-OMPs and lipoproteins have been annotated in the *Leptospira* genome, many are still identified as hypothetical proteins. While some βb-OMPs and lipoproteins were characterized by means other than RV, we believe that a large number of these types of prospective vaccine antigens are yet to be discovered.

In addition to RV, recent advances in vaccine research using the structural information of antigens have led to the development of structural vaccinology (SV) (29). Based primarily on protein design for the optimization of antigen structure and consequently enhanced protection, SV is a combination of structural biology, immunology, and bioinformatics (30). Solving protein structures is time consuming, expensive, and sometimes difficult, especially for proteins such as the βb-OMPs (31, 32). Advances in structural bioinformatics has allowed reliable prediction of three-dimensional (3D) structural models of proteins based on the alignment of the query sequence to known-structure templates, which may be identified using sequence similarity searches (homology modeling) or fold recognition (threading) methods (31, 33). In contrast to homology-based methods, protein threading allows the prediction of structural models of proteins with low similarity to known proteins (no orthologs). This is attractive for leptospiral proteins, as there are no solved structures for leptospiral βb-OMPs. In the current study, we report the application of RV and SV toward the discovery of leptospiral vaccine candidates, i.e., βb-OMPs and OM lipoproteins, structural modeling, the *in silico* identification of major histocompatibility complex (MHC-II)-binding epitopes, and the selection of surface-related immunogenic epitopes.

## Results

### Identification of βb-OMPs and OM Lipoproteins in the *Leptospira interrogans* Genome

**Figure 1:**
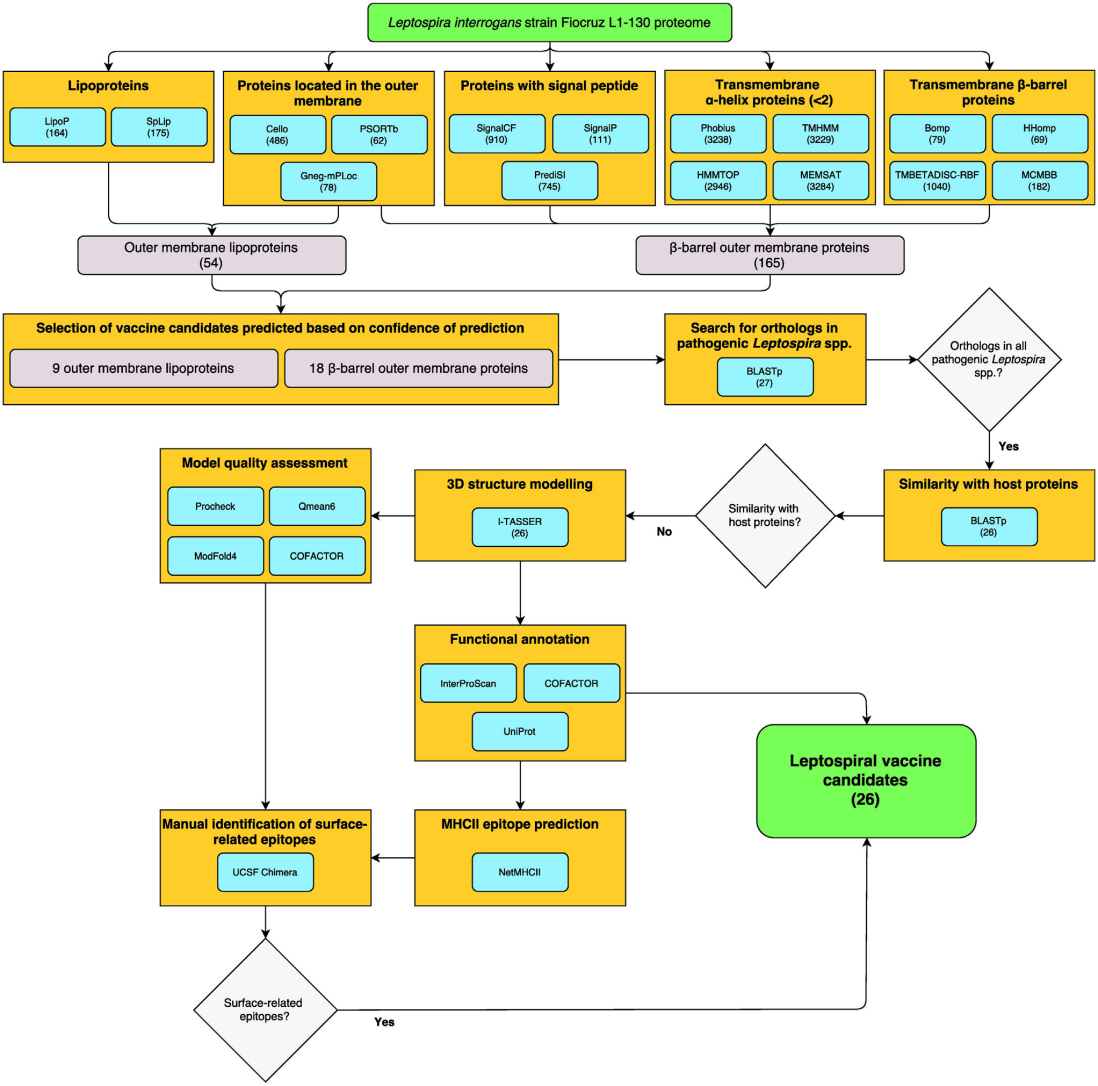
**Reverse vaccinology (RV)- and structural vaccinology (SV)-based bioinformatics workflow for the identification of leptospiral vaccine candidates**. The complete LIC genome-derived proteome provided the input sequences for 16 bioinformatics programs for the prediction of βb-outer membrane proteins (OMPs) and outer membrane (OM) lipoproteins. The number of proteins selected by each program is shown in parentheses. High confidence predicted proteins were screened using a Python algorithm. Proteins conserved among pathogenic *Leptospira* spp. and with no similarity to host proteins were selected for the three-dimensional (3D) structure prediction by fold recognition modeling. Functional annotation was based on the primary structure and on the 3D models. Strong binder major histocompatibility complex-II immunogenic epitopes were predicted for the selected proteins and a structural approach was employed to map the surface-related immunogenic regions in the βb-OMPs. The final list of conserved βb-OMPs and OM lipoproteins and the surface-related epitopes are novel prospective leptospiral vaccine candidates.

Reverse vaccinology was employed for the identification of surface-exposed proteins, including βb-OMPs and OM lipoproteins in the genome of *L. interrogans* serovar Copenhageni strain Fiocruz L1-130 (LIC). The bioinformatics workflow and total numbers of proteins identified by each bioinformatics algorithm (predictor) are shown (Figure 1). The predictors for subcellular localization (Cello, PSORTb, and Gneg-mPLoc) found 523 proteins (by 1 or more predictor) located in the OM of *L. interrogans*, 24 were identified by all three predictors. A total of 1,196 proteins were predicted to contain a signal peptide (SP) for translocation across the cytoplasmic membrane to, e.g., the OM. Of these, 72 proteins were identified by all three SP predictors (SignalCF, SignalP, and PrediSI). One or more of the transmembrane α-helix (TMH) predictors (Phobius, TMHMM, HHTOP, and MEMSAT) determined that 3,302 proteins did not contain a TMH, this was reduced to 2,929 proteins by all 4 predictors. The transmembrane β-barrel (βb) structure predictors (Bomp, HHomp, TMBETADISC-RBF, and MCMBB) identified 1,085 βb-OMPs, 20 of which were confirmed by all 4 predictors. Finally, 230 proteins were identified as lipoproteins by at least one of the predictors, 108 by both (LipoP and SpLip). A complete list of the proteins identified by each individual predictor is provided (Table S1 in Supplementary Material).

A total of 165 βb-OMPs were identified by at least 1 predictor, while only 1 protein (LIC10714) was identified by all predictors. In this study, a βb-OMP was defined as a protein predicted to contain a βb structure, an SP, and <2 TMHs. As an SP can be identified as a TMH, a protein containing a single, N-terminal, TMH was classified as a non-TMH protein. An OM lipoprotein was defined as a protein predicted to be located in the OM and that contained a lipobox. A total of 54 OM lipoproteins were identified by at least one predictor of each feature; however, no proteins were identified at the intersect between all the predictors for lipoproteins and cell localization. A list of the 165 βb-OMPs and 54 OM lipoproteins identified, the gene products as annotated in the LIC genome and the result for each predictor is provided (Table S2 in Supplementary Material).

### Filtering Predicted Protein Features with Increased Confidence

Due to the particularities of each predictor, low agreement between the different bioinformatics tools was expected when identifying the same feature. To reduce the impact of a prediction when non-weighted (naïve) voting resulted in ambiguities (e.g., two negative and one positive result by three different predictors), we used an iterative weighted voting system. An in-house Python script was written to integrate the results and to identify those proteins with a high level of confidence in the consensus prediction among those selected by at least one predictor. The lists of 165 βb-OMPs and 54 OM lipoproteins were used as the script input and, after thousand iterations, each predictor received a final prediction weight (Table S3 in Supplementary Material). A final voting score >0.5 (0–1 scale) for each feature of interest (e.g., >0.5 for OM localization and >0.5 for lipoprotein prediction) was indicative of a high level of confidence in the prediction, resulting in the selection of 18 βb-OMPs and 9 OM lipoproteins (Table 1).

**Table 1:** **Functional annotation of βb-outer membrane proteins (OMPs) and outer membrane (OM) lipoproteins**.

	Gene ID	Product/original annotation^a^	Uniprot protein name	KEGG orthologs	Interpro scan analysis
βb-OMPs	LIC10496	Conserved hypothetical protein	Uncharacterized protein	TolC-like OMP	OM efflux protein
	LIC10714	OM receptor protein	OM receptor protein (*smc*)	OM receptor for Fe3^+^-dicitrate/TonB-dependent receptor (TBDR)	TBDR
	LIC10881	OMP, TonB dependent	OMP, TonB dependent	TBDR	TBDR [plug and β-barrel (βb) domains^b^]
	LIC10896	TonB-dependent outer membrane receptor	TonB-dependent outer membrane receptor (*fecA*)	TonB-dependent outer membrane receptor	TBDR
	LIC10964	TonB-dependent outer membrane hemin receptor	TonB-dependent outer membrane hemin receptor (*phuR*)	TonB-dependent outer membrane hemin receptor	TBDR
	LIC11086	Conserved hypothetical protein	Uncharacterized protein	Hypothetical protein	MetA-pathway of phenol degradation, putative
	LIC11211	Hypothetical protein	Uncharacterized protein	Hypothetical protein	None predicted
	LIC11268	Conserved hypothetical protein	Uncharacterized protein	Hypothetical protein	Alginate export domain
	LIC11458	OMP, porin superfamily	OMP, porin superfamily (outer membrane LPS export porin—lps-ep—family^c^)	Hypothetical protein	None predicted
	LIC11506	OMP	OMP	Hypothetical protein	Predicted OMP, Leptospiraceae
	LIC11623	OMP	OMP	Hypothetical protein, Oma87-like OMP, BamA	Bacterial surface antigen (D15)/surface antigen variable number
	LIC12254	OMP	OMP	Hypothetical protein, Oma87-related protein, surface antigen (D15)	Bacterial surface antigen (D15)
	LIC12374	OMP, TonB dependent	OMP, TonB dependent (*btuB*)	TonB-dependent outer membrane receptor, obalamin receptor protein	TBDR, βb, plug domain
	LIC12575	Cytoplasmic membrane protein	Cytoplasmic membrane protein	Cytoplasmic membrane protein, TolC-like protein	Outer membrane efflux protein
	LIC13477	Conserved hypothetical protein	Uncharacterized protein	Hypothetical protein	None predicted
	LIC20019	Conserved hypothetical protein	Uncharacterized protein	Hypothetical protein	Putative porin/Porin 6
	LIC20087	OMP	OMP	Putative OMP, hypothetical protein	None predicted
	LIC20151	TonB-dependent outer membrane receptor	TonB-dependent outer membrane receptor	TonB-dependent hemin-binding protein	TBDR, plug and βb domains

OM lipoproteins	LIC10024	Adenylate/guanylate cyclase (AGC)	AGC	AGC	7TM-DISM receptor, extracellular domain, type 1; nucleotide cyclase; adenylyl cyclase class-3/4/guanylyl cyclase
	LIC10647	Conserved hypothetical protein	Uncharacterized protein	Hypothetical protein	None predicted
	LIC10713	Putative lipoprotein	Putative lipoprotein	Putative lipoprotein, peptidase M75	None predicted
	LIC11003	LipL71	LipL71	LipL71, peptidoglycan-binding protein LysM	Domain of unknown function DUF4398; LysM domain
	LIC11755	Conserved hypothetical protein	Uncharacterized protein	Hypothetical protein	None predicted
	LIC12048	Conserved hypothetical protein	Uncharacterized protein	Hypothetical protein	None predicted
	LIC13411	Putative lipoprotein	Putative lipoprotein	Hypothetical proteins	None predicted
	LIC20172	Lipoprotein	Lipoprotein	Hypothetical proteins	LruC domain; domain of unknown function DUF4842

*^a^As annotated in the LIC genome*.

^b^Domains corresponding to LIC10881* (LIC10881 + LIC10882).

*^c^Annotated based on results from Transporter Classification Database*.

#### Conservation of Surface-Exposed Proteins among *Leptospira* spp. and Similarity to Mammalian Host Proteins

The genome sequences from 20 additional *Leptospira* spp. (Table S4 in Supplementary Material) were screened for orthologs of the 18 βb-OMPs and the 9 OM lipoproteins. Interestingly, all the pathogenic *Leptospira* spp. contained orthologs of the 18 βb-OMPs (Table 2), except for *L. kmetyi* that did not contain an ortholog of LIC10881. This protein was also absent from the intermediate and saprophytic *Leptospira* spp. Of the 9 OM lipoproteins, LIC12048 was absent in *L. borgpetersenii*, and LIC20172 was not found in *Leptospira kirschneri*, these proteins were, however, retained for further analysis as they were present in the majority of the pathogenic *Leptospira* spp. The OM lipoprotein LIC12690 was excluded from further analysis as it was only found in *L. interrogans, L. kirschneri*, and *Leptospira noguchii*. In addition, the βb-OMPs and OM lipoproteins were screened against selected mammalian proteomes for similarity to any of these potential vaccine candidates. No similarities were found among any of the leptospiral proteins and human, dogs, cattle, pig, horse, or sheep proteins (data not shown).

**Table 2:** **Conservation of the 18 βb-outer membrane proteins (OMPs) and 9 outer membrane (OM) lipoproteins in 20 *Leptospira* spp**.

	Gene ID	Pathogenic *Leptospira* spp.									Intermediate *Leptospira* spp.					Saprophytic *Leptospira* spp.					
		*ale*.	*als*.	*bor*.	*kir*.	*kme*.	*may*.	*nog*.	*san*.	*wei*.	*bro*.	*fai*.	*ina*.	*lic*.	*wolf*.	*bif*.	*mey*.	*ter*.	*van*.	*wolb*.	*yan*.
βb-OMPs	LIC10496	✓	✓	✓	✓	✓	✓	✓	✓	✓	✓	✓	✓	✓	–	✓	✓	✓	✓	✓	✓
	LIC10714	✓	✓	✓	✓	✓	✓	✓	✓	✓	✓	✓	✓	✓	✓	✓	✓	✓	✓	✓	✓
	LIC10881*	✓	✓	✓	✓	–	✓	✓	✓	✓	–	–	–	–	–	–	–	–	–	–	–
	LIC10896*	✓	✓	✓	✓	✓	✓	✓	✓	✓	–	–	–	✓	✓	✓	✓	✓	✓	✓	✓
	LIC10964	✓	✓	✓	✓	✓	✓	✓	✓	✓	✓	✓	✓	✓	✓	–	–	–	✓	–	–
	LIC11086	✓	✓	✓	✓	✓	✓	✓	✓	✓	✓	✓	✓	✓	✓	✓	✓	✓	✓	✓	✓
	LIC11211	✓	✓	✓	✓	✓	✓	✓	✓	✓	✓	✓	✓	–	–	✓	✓	✓	–	–	–
	LIC11268	✓	✓	✓	✓	✓	✓	✓	✓	✓	✓	✓	✓	✓	✓	✓	✓	✓	✓	✓	✓
	LIC11458	✓	✓	✓	✓	✓	✓	✓	✓	✓	✓	✓	✓	✓	✓	✓	✓	✓	✓	✓	✓
	LIC11506	✓	✓	✓	✓	✓	✓	✓	✓	✓	–	–	✓	✓	✓	–	✓	✓	✓	✓	✓
	LIC11623	✓	✓	✓	✓	✓	✓	✓	✓	✓	✓	✓	✓	✓	✓	✓	✓	✓	✓	✓	✓
	LIC12254	✓	✓	✓	✓	✓	✓	✓	✓	✓	✓	✓	✓	✓	✓	–	✓	–	–	–	✓
	LIC12374	✓	✓	✓	✓	✓	✓	✓	✓	✓	✓	✓	✓	✓	✓	✓	✓	✓	✓	✓	✓
	LIC12575	✓	✓	✓	✓	✓	✓	✓	✓	✓	✓	✓	✓	✓	✓	✓	✓	✓	✓	✓	✓
	LIC13477	✓	✓	✓	✓	✓	✓	✓	✓	✓	✓	✓	✓	✓	✓	✓	✓	✓	✓	✓	✓
	LIC20019	✓	✓	✓	✓	✓	✓	✓	✓	✓	✓	✓	✓	✓	✓	✓	✓	✓	✓	✓	✓
	LIC20087	✓	✓	✓	✓	✓	✓	✓	✓	✓	✓	✓	✓	✓	✓	✓	✓	✓	✓	✓	✓
	LIC20151	✓	✓	✓	✓	✓	✓	✓	✓	✓	–	–	✓	✓	✓	✓	✓	✓	✓	✓	✓

OM lipoproteins	LIC10024	✓	✓	✓	✓	✓	✓	✓	✓	✓	✓	✓	✓	✓	✓	–	–	–	–	–	–
	LIC10647	✓	✓	✓	✓	✓	✓	✓	✓	✓	–	–	–	✓	✓	–	–	–	–	–	–
	LIC10713	✓	✓	✓	✓	✓	✓	✓	✓	✓	✓	✓	✓	✓	✓	–	✓	✓	✓	✓	–
	LIC11003	✓	✓	✓	✓	✓	✓	✓	✓	✓	✓	✓	✓	✓	✓	✓	✓	✓	✓	✓	✓
	LIC11755	✓	✓	✓	✓	✓	✓	✓	✓	✓	✓	✓	✓	✓	✓	✓	✓	✓	✓	✓	✓
	LIC12048	✓	✓	–	✓	✓	✓	✓	✓	✓	–	–	–	✓	✓	✓	–	–	–	–	–
	LIC12690	–	–	–	✓	–	–	✓	–	–	–	–	–	✓	–	✓	✓	✓	✓	✓	✓
	LIC13411	✓	✓	✓	✓	✓	✓	✓	✓	✓	✓	✓	✓	✓	✓	✓	✓	✓	✓	✓	✓
	LIC20172	✓	✓	✓	–	✓	✓	✓	✓	✓	✓	–	✓	✓	✓	✓	✓	✓	✓	✓	✓

The search for leptospiral orthologs was repeated using LIC10881* and LIC10896*.

*✓, conserved ortholog; −, no ortholog; ale., L. alexanderi; als., L. alstonii; bor., L. borgpetersenii; kir., L. kirschneri; kme., L. kmetyi; may., L. mayottensis; nog., L. noguchii; san., L. santarosai; wei., L. weilii; bro., L. broomii; fai., L. fainei; ina., L. inadai; lic., L. licerasiae; wolf., L. wolffii; bif., L. biflexa; mey., L. meyeri; ter., L. terpstrae; van., L. vanthielii; wolb., L. wolbachii; yan., L. yanagawae*.

#### Multiple Sequence Alignments of *Leptospira* Surface-Exposed Proteins

A multiple sequence alignment was performed with each βb-OMP and OM lipoprotein and their respective orthologs. The alignments suggested that LIC10881 and LIC10964 were truncated sequences compared to their respective orthologs (Data Sheet S1 in Supplementary Material). An analysis of the genome region that contained the LIC10881 coding sequence (CDS) revealed the presence of a possible point mutation in the last nucleotide of LIC10881, that created a stop codon. When the point mutation was altered to a tryptophan codon (TG**A** → TG**G**), the LIC10881 and LIC10882 CDS were reassembled as a single CDS, LIC10881*, and the multiple alignment of LIC10881* was no longer truncated (Data Sheet S1 in Supplementary Material). Similarly, we identified a potential frameshift mutation in the LIC10896 CDS that was altered by the insertion of a cytosine at position 2,597. Reassembly of LIC10896 and LIC10895 identified a single CDS, LIC10896*. When the LIC10896* protein sequence was included in the multiple alignment, the sequence was no longer truncated (Data Sheet S1 in Supplementary Material). The modified LIC10881* and LIC10896* proteins were used for further analysis.

### Functional Annotation Based on Protein Sequence

Eleven of the 18 βb-OMPs identified in this study were originally annotated as OMPs in the LIC genome, one was annotated as a cytoplasmic membrane protein, and six were annotated as hypothetical proteins (Table 1). Seven of the eight OM lipoproteins were annotated as hypothetical proteins or putative lipoproteins in the original genome annotation. The exception was LIC10024 that was annotated as an adenylate/guanylate cyclase (AGC). The KEGG database was used to identify any corresponding orthologs and their annotations in the genomes of the other pathogenic *Leptospira* spp. Of note, the genome annotations of LIC10496, LIC11458, LIC11506, and LIC12575 differed substantially to those of their orthologs (Table 1).

A functional annotation was performed using InterProScan for the 18 βb-OMPs and 8 OM lipoproteins (Table 1). InterProScan identified OMP-related domains in 14 of the βb-OMPs. However, it failed to identify functional domains in four of the predicted βb-OMPs (LIC11211, LIC11458, LIC13477, and LIC20087) and five out of eight OM lipoproteins (LIC10647, LIC10713, LIC11755, LIC12048, and LIC13411). Of the remaining OM lipoproteins, two (LIC11003 and LIC20172) contained domains with an unknown function and LIC10024 was confirmed as an AGC protein.

### Structural Modeling Improved the Prediction of OMP Function

**Figure 2:**
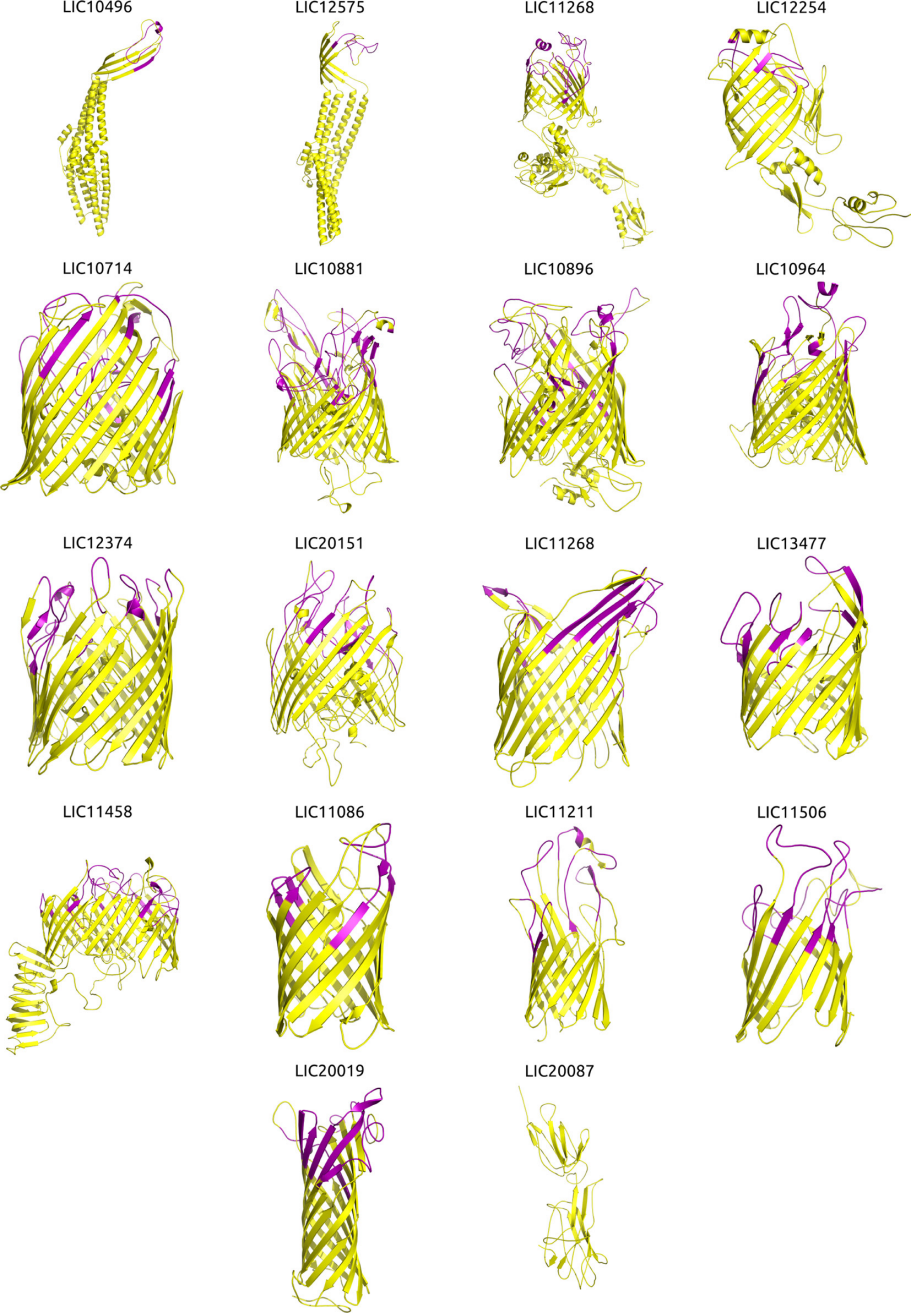
**Three-dimensional (3D) structures of the βb-outer membrane proteins (OMPs) and mapping of the immunogenic surface-related epitopes (SRE)**. Structural modeling was performed using I-TASSER, and the structures were visualized and the images generated using PyMOL. Proteins are orientated as following: the upper portion is surface-exposed on the OM and the lower portion is located in the periplasmic space. The orientation was derived from an interpretation of the orientation and structure of the matching PDB structure. Immunogenic major histocompatibility complex-II epitopes (strong binders) for 14 HLAs were predicted using NetMHCII and mapped onto the βb-OMPs structural models. Immunogenic SRE are indicated in purple in each βb-OMP structure.

**Figure 3:**
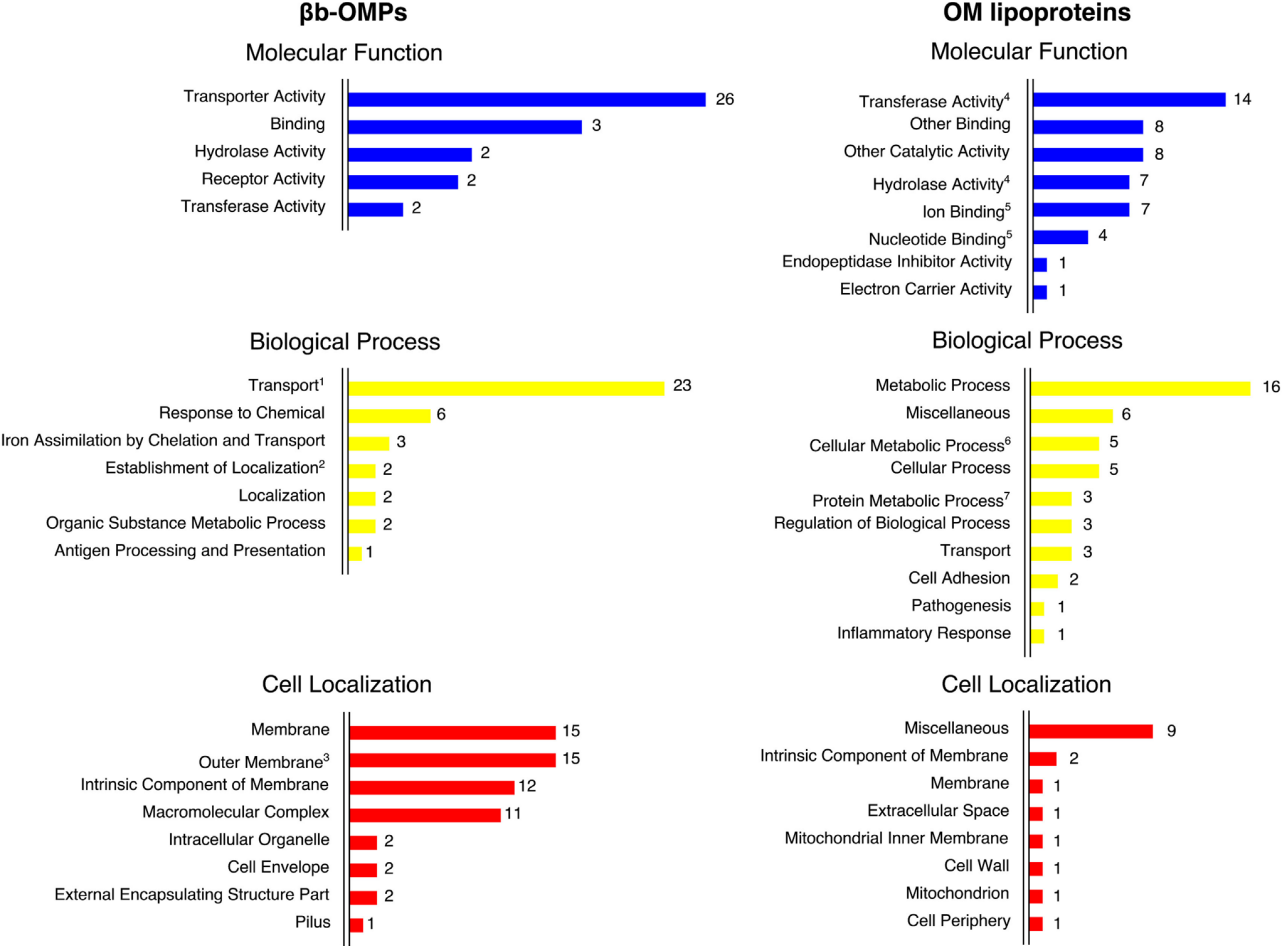
**The Gene Ontology (GO) terms predicted by COFACTOR were based on the structural models of the βb-outer membrane proteins (OMPs) and outer membrane (OM) lipoproteins**. The GO terms were assigned for three domains: molecular function, biological process, and cell localization. COFACTOR usually predicted more than one term for each GO domain for the target protein structure. The GO terms were collapsed to the closest parent term (based on the AmiGO2 database) to facilitate the overall view of the GO terms. In some cases, related terms were maintained for convenience, as indicated: 1—*transport* is also a child term of *establishment of localization*; 2—*establishment of localization* is also a child term of *localization*; 3—*outer membrane* is also a child term of *membrane*; 4—*transferase activity* and *hydrolase activity* are also children terms of *catalytic activity*; 5—*ion binding* and *nucleotide binding* are also children terms of *binding*; 6—*cellular metabolic process* is also a child term of *metabolic process* and *cellular process*; and 7—*protein metabolic process* is also a child term of *metabolic process*.

For each protein sequence, the I-TASSER software predicted five 3D models and each model was ranked in order of quality by a *C*-score. The top-ranking models for 16 of the βb-OMPs contained a typical transmembrane βb structure (Figure 2). While the LIC11268 model that contained a transmembrane βb structure was ranked third, it was included for further analysis. The structural models of the eight OM lipoproteins are shown (Data Sheet 3 in Supplementary Material). The refined βb-OMP and OM lipoprotein models were analyzed by COFACTOR, a structure-based method for assigning biological function to protein molecules. A COFACTOR output includes the top 10 closest structures in PDB ranked by TM-score, Gene Ontology (GO) terms associated with the protein model, and the root-mean-square deviation of atomic position (RMSD) related to the best templates used for modeling. GO terms, including molecular function, biological process, and cellular location, associated with the protein 3D models were predicted based on the GOs assigned to the template structures and provided a functional insight into the selected leptospiral proteins. When more than one GO term per category was predicted for each protein, some were related to more distant templates (the last of the 10 closest structures), they were collapsed to the closest parent term on the AmiGO2 database and are shown (Figure 3) according to their frequency in the βb-OMPs and OM lipoproteins. The complete list of GO terms predicted for each protein structure is provided (Table S5 in Supplementary Material). As expected, most of the βb-OMP molecular function and biological process GO terms were related to transportation. This was supported by the cell location GO term, most of the βb-OMPs were classified as membrane or OM. The GO terms predicted for the OM lipoproteins were distributed among several different categories. Most of the GO terms for molecular function were for catalytic/binding activity and the predicted biological processes were related to metabolism. The predicted cell location GO terms were diverse (Figure 3; Table S5 in Supplementary Material).

#### Quality of Predicted Models

The closest PDB structure (PDB code, protein name, and organism of origin) for the predicted structural models and an estimation of the quality are shown (Table 3). The quality of each predicted model was evaluated using Procheck, QMEAN6, and ModFOLD4. Procheck was used to evaluate the stereochemical quality of each protein structure and the proportion of disallowed residues in the Ramachandran plot ranged from 0.7% for LIC10496 to 11.6% for LIC20151. When assessed by QMEAN and ModFOLD, most of the βb-OMPs models had good quality values for the overall structure.

**Table 3:** **Closest PDB structure and quality assessment for the βb-outer membrane proteins (OMPs) and outer membrane (OM) lipoproteins models**.

	Gene ID	Closest structure on PDB (PDB code), organism of origin^a^	Quality assessment					
			TM-score^a^	RMSD^a^	Ramachandran disallowed residues (%)^b^	ModFold score	Confidence and *P*-value ModFold	QMEAN score
βb-OMPs	LIC10496	OMP TolC (1tqq), *Escherichia coli*	0.862	1.10	0.7	0.5390	HIGH: 3.324E−3	0.403
	LIC10714	TonB-dependent receptor (TBDR)—ferrichrome-iron receptor FhuA (1fI1), *E. coli*	0.840	1.10	8.3	0.6236	HIGH: 1.38E−3	0.327
	LIC10881*	TBDR—transferrin-binding protein A TbpA (3v89), *Neisseria meningitidis*	0.592	4.86	3.4	0.3089	MEDIUM: 3.63E−2	0.344
	LIC10896*	TBDR—ferripyoverdine receptor FpvA (2w78), *Pseudomonas aeruginosa*	0.719	2.25	5.0	0.3127	MEDIUM: 3.50E−2	0.197
	LIC10964	TBDR—Zn-transporter ZnuD (4rdr), *N. meningitidis*	0.837	1.37	6.3	0.5544	HIGH: 2.834E−3	0.326
	LIC11086	Protein involved in meta-pathway of phenol degradation-like protein, Pput2725 (4rl8), *Pseudomonas putida*	0.769	1.31	2.3	0.2233	LOW: 8.848E−2	0.231
	LIC11211	Toluene transporter TbuX (3bry), *Ralstonia pickettii*	0.873	2.28	3.1	0.0000	POOR: 9.017E−1	0.184
	LIC11268	TBDR—transferrin-binding protein A TbpA (3v89), *N. meningitidis*	0.914	1.65	4.2	0.0000	POOR: 9.017E−1	0.137
	LIC11458	LPS assembly protein LptD (4q35), *Shigella flexneri*	0.722	1.56	5.4	0.0777	POOR: 4.02E−1	0.254
	LIC11506	OM Porin OmpG (2iww), *E. coli*	0.660	3.33	3.1	0.1192	POOR: 2.613E−1	0.083
	LIC11623	OMP assembly factor BamA (4k3b), *Neisseria gonorrhoeae*	0.506	3.37	4.9	0.2569	LOW: 6.245E−2	0.349
	LIC12254	OMP assembly factor BamA lacking polypeptide translocation-associated domains 1–3 (4k3c), *H. ducreyi*	0.882	2.29	4.6	0.2076	POOR: 1.042E−1	0.177
	LIC12374	TBDR—vitamin B12 transporter BtuB (2gsk), *E. coli*	0.824	2.11	6.4	0.6066	HIGH: 1.646E−3	0.282
	LIC12575	OMP TolC (1tqq), *E. coli*	0.849	1.01	1.8	0.5207	HIGH: 4.02E−3	0.403
	LIC13477	Alginate production protein AlgE (3rbh), *P. aeruginosa*	0.804	1.81	3.3	0.2664	LOW: 5.653E−2	0.259
	LIC20019	Plasminogen activator Pla/coagulase/fibrinolysin (2x4m), *Yersinia pestis*	0.769	2.02	2.8	0.2117	LOW: 9.987E−2	0.279
	LIC20087	Major Pilin Protein (4s3l), S*treptococcus pneumoniae*	0.896	2.20	3.7	0.0000	POOR: 9.017E−1	0.246
	LIC20151	TBDR—vitamin B12 transporter BtuB (2gsk), *E. coli*	0.794	1.57	11.6	0.5631	HIGH: 2.588E−3	0.315

OM lipoproteins	LIC10024	Adenylate cyclase type 10 (4clf), *Homo sapiens*	0.509	3.15	7.9	0.2229	LOW: 8.883E−2	0.229
	LIC10647	Major fimbrial subunit protein (4q98), *Porphyromonas gingivalis*	0.828	2.89	3.0	0.0000	POOR: 9.017E−1	0.21
	LIC10713	Putative iron-regulated protein A (4ecg), *Parabacteroides distasonis*	0.743	2.02	3.6	0.5401	HIGH: 3.286E−3	0.438
	LIC11003	Cytoplasmic domain of bacterial cell division protein EzrA (4uxv), *Bacillus subtilis*	0.853	2.24	2.0	0.0226	POOR: 7.126E−1	0.31
	LIC11755	RNA-dependent RNA polymerase (3ja4), Cypovirus 1	0.923	1.54	4.3	0.1582	POOR: 1.741E−1	0.042
	LIC12048	Tc toxin/TcdB2/TccC3 (4o9x), *Photorhabdus luminescens*	0.890	2.48	4.5	0.0000	POOR: 9.017E−1	0.144
	LIC13411	Chalcone-flavanone isomerase family protein (4doo), *Arabidopsis thaliana*	0.669	2.46	2.1	0.0000	POOR: 9.017E−1	0.342
	LIC20172	Secretory component of immunoglobulin A (3chn), *H. sapiens*	0.799	2.64	4.9	0.0000	POOR: 9.017E−1	0.018

*^a^Obtained by COFACTOR analysis*.

*^b^Obtained by Procheck analysis*.

### Surface-Related Immunogenic Epitope Prediction

**Figure 4:**
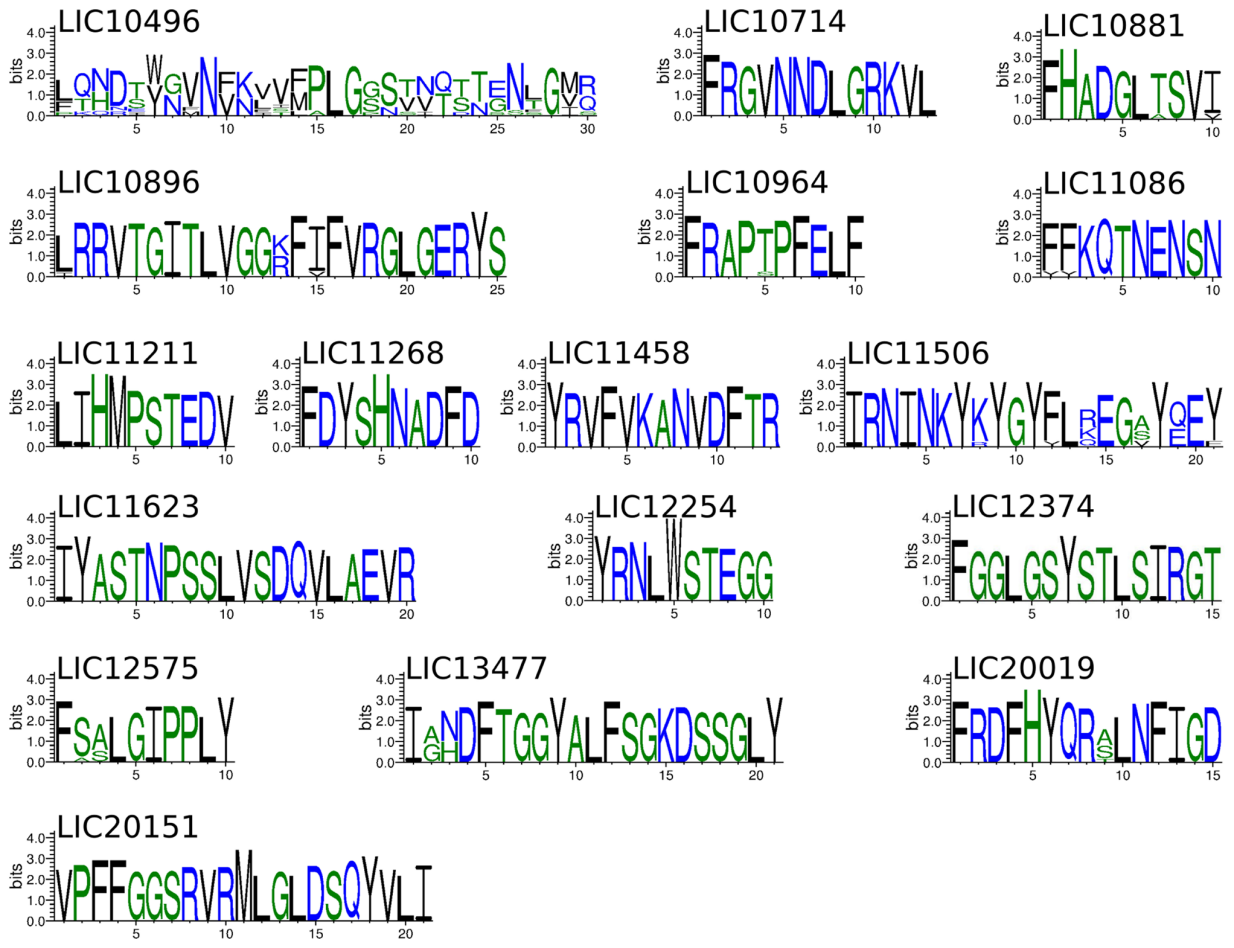
**Sequence logos showing the frequency of amino acid residues at each position of the immunogenic surface-related epitopes (SREs) identified in the βb-outer membrane proteins (OMPs), according to multiple sequence alignment of the orthologs from pathogenic *Leptospira* spp**. Only the most conserved SRE from each βb-OMPs is shown. The overall height of the stack indicates the level of sequence conservation at that position, while the height of symbols within the stack indicates the relative frequency of each amino at that position.

Due to the importance of phagocytosis in the clearance of leptospires during an infection, the presence of MHC class II-binding epitopes in the 18 βb-OMPs and 8 OM lipoproteins was evaluated. NetMHCII was used to predict 15 amino acid long peptides that can bind, at different affinity levels, to MHC class II molecules encoded by several HLAs. Predictions were made for 14 HLA-DRB alleles and only strong binder (SB) epitopes (IC50 <50 nM) were considered for analysis. Each predicted immunogenic epitope had a 9mer core that was aligned, and a consensus sequence was determined. The location of each SB epitope in their respective structural models was identified (Figure 2). The correct orientation of the βb structural models in the OM was deduced using the corresponding PDB structure as a template. The total number of 9mers and 15mers predicted for each βb-OMP and OM lipoprotein, as well as the number of surface-related 9mers and 15mers of the βb-OMPs is provided (Table 4). LIC20087 and the OM lipoproteins were not analyzed for surface-related epitopes (SREs) as they did not contain βb structures; therefore, it was not possible to determine their localization in the OM. Each immunogenic epitope (9mer core or consensus) was evaluated for conservation among the orthologous proteins. A representative set of the most conserved immunogenic SREs for each βb-OMP is shown (Figure 4). The SREs were relatively well conserved in all the βb-OMPs. The alignments of the βb-OMP immunogenic epitopes to the corresponding regions in the orthologs from pathogenic *Leptospira* spp. are highlighted in the alignment files (Data Sheet S1 in Supplementary Material). A list of the βb-OMP and OM lipoprotein SB epitopes for all MHC-II alleles is provided (Tables S6 and S7 in Supplementary Material, respectively).

**Table 4:** **Number of predicted strong binder (SB) major histocompatibility complex-II (MHC-II) epitopes in each βb-outer membrane protein (OMP) and outer membrane (OM) lipoproteins for 14 HLAs**.

	Gene ID	Number of surface-related epitopes (SRE) identified among total SB epitopes—surface-related 9mer (SRE9)/SRE15 (SB9/SB15)^c^													
		HLA-DRB 10101	HLA-DRB 10301	HLA-DRB 10401	HLA-DRB 10404	HLA-DRB 10405	HLA-DRB 10701	HLA-DRB 10802	HLA-DRB 10901	HLA-DRB 11101	HLA-DRB 11302	HLA-DRB 11501	HLA-DRB 30101	HLA-DRB 40101	HLA-DRB 50101
βb-OMPs^a^	LIC10496	3/17 (29/111)	0/0 (8/25)	1/5 (4/16)	0/0 (6/27)	1/5 (4/23)	0/0 (9/42)	0/0 (0/0)	1/3 (3/10)	0/0 (10/39)	2/12 (7/44)	0/0 (3/10)	1/5 (3/16)	0/0 (15/50)	0/0 (14/65)
	LIC10714	14/60 (44/171)	2/12 (5/23)	8/28 (14/46)	3/14 (11/39)	7/21 (11/35)	8/39 (21/74)	1/3 (3/11)	3/17 (7/30)	1/1 (9/35)	1/7 (5/30)	3/8 (8/38)	2/11 (6/35)	2/22 (8/31)	5/17 (19/68)
	LIC10881*	23/56 (49/157)	2/7 (8/20)	2/6 (8/16)	9/30 (16/57)	10/25 (14/38)	5/20 (20/97)	0/0 (0/0)	3/7 (6/15)	8/22 (17/51)	1/7 (8/38)	5/14 (14/41)	4/23 (8/44)	5/14 (6/15)	7/31 (18/89)
	LIC10896*	23/73 (68/251)	2/7 (9/33)	7/19 (17/59)	3/12 (10/37)	6/21 (24/73)	9/41 (30/132)	3/4 (8/23)	2/10 (12/53)	7/29 (28/94)	1/7 (6/34)	1/2 (14/54)	2/10 (6/29)	2/4 (8/21)	5/23 (27/94)
	LIC10964	9/33 (50/182)	2/7 (4/12)	3/8 (10/34)	0/0 (6/32)	5/21 (15/53)	6/35 (26/111)	0/0 (0/0)	4/10 (7/22)	1/4 (21/51)	2/13 (6/37)	2/5 (10/32)	0/0 (2/7)	1/3 (11/34)	5/28 (19/88)
	LIC11086	2/9 (26/79)	0/0 (1/4)	1/5 (3/14)	1/6 (2/7)	1/5 (4/13)	2/6 (11/41)	1/6 (1/6)	0/0 (9/26)	2/11 (9/36)	0/0 (6/26)	0/0 (7/33)	1/7 (1/7)	0/0 (1/6)	1/3 (11/43)
	LIC11211	5/13 (21/57)	1/3 (3/10)	0/0 (9/27)	1/6 (6/24)	1/2 (5/19)	1/4 (12/40)	0/0 (0/0)	0/0 (1/3)	1/2 (11/35)	0/0 (3/11)	1/5 (5/22)	0/0 (1/5)	0/0 (5/14)	0/0 (9/40)
	LIC11268	9/44 (26/105)	2/10 (4/14)	2/6 (6/24)	1/1 (7/23)	3/9 (13/42)	4/18 (10/42)	0/0 (0/0)	0/0 (0/0)	3/10 (6/19)	1/1 (4/13)	0/0 (5/17)	2/9 (9/37)	1/3 (3/9)	3/15 (6/25)
	LIC11458	16/56 (62/205)	1/3 (11/38)	7/30 (19/61)	4/19 (19/76)	7/26 (18/66)	4/21 (25/100)	0/0 (1/2)	3/12 (7/23)	6/23 (31/109)	2/13 (8/42)	2/9 (14/61)	1/7 (5/30)	1/7 (2/8)	3/20 (28/134)
	LIC11506	7/28 (27/96)	0/0 (0/0)	2/7 (7/21)	1/1 (4/12)	2/5 (8/26)	5/18 (20/78)	0/0 (0/0)	1/6 (6/19)	4/20 (8/37)	1/5 (2/12)	4/20 (12/47)	0/0 (4/18)	0/0 (1/6)	5/17 (12/36)
	LIC11623	16/65 (60/221)	1/6 (11/43)	4/16 (12/52)	0/0 (8/25)	2/9 (14/49)	5/24 (29/123)	0/0 (0/0)	1/5 (5/20)	6/16 (26/85)	3/14 (7/30)	1/1 (17/61)	2/12 (9/48)	5/18 (17/56)	2/7 (27/102)
	LIC12254	4/27 (24/89)	0/0 (3/15)	0/0 (2/9)	1/5 (8/42)	0/0 (6/17)	1/7 (10/48)	1/2 (3/6)	4/6 (9/18)	2/7 (10/37)	2/11 (8/30)	0/0 (5/29)	0/0 (8/25)	0/0 (0/1)	1/7 (11/56)
	LIC12347	12/40 (42/144)	1/3 (7/29)	2/11 (8/27)	3/13 (8/37)	2/7 (10/34)	5/21 (17/80)	0/0 (0/0)	0/0 (2/7)	2/7 (10/26)	1/4 (2/10)	1/7 (6/26)	1/7(10/50)	0/0 (0/0)	2/6 (16/55)
	LIC12575	5/16 (32/98)	0/0 (5/19)	0/0 (1/6)	0/0 (5/13)	1/5 (5/27)	2/12 (9/48)	0/0 (0/0)	0/0 (3/9)	0/0 (9/31)	1/2 (5/21)	0/0 (4/14)	0/0 (3/14)	0/0 (7/25)	2/8 (11/46)
	LIC13477	9/27 (32/112)	0/0 (1/7)	1/2 (7/31)	0/0 (5/9)	1/5 (9/31)	1/12 (12/49)	0/0 (0/0)	2/8 (3/12)	2/3 (7/21)	1/2 (2/5)	2/4 (9/33)	1/5 (4/17)	0/0 (1/2)	2/11 (10/41)
	LIC20019	7/24 (21/70)	1/6 (1/6)	2/7 (5/16)	3/13 (4/19)	9/32 (10/38)	4/24 (13/50)	0/0 (0/0)	1/7 (1/7)	5/10 (11/33)	0/0 (2/2)	2/8 (7/19)	3/14 (5/21)	2/9 (3/15)	1/7 (8/27)
	LIC20087	−/− (17/94)	−/− (1/5)	−/− (4/15)	−/− (4/17)	−/− (11/32)	−/− (12/52)	−/− (1/7)	−/− (4/16)	−/− (7/22)	−/− (1/5)	−/− (4/17)	−/− (2/8)	−/− (1/6)	−/− (7/31)
	LIC20151	14/62 (44/157)	3/9 (5/16)	5/20 (10/35)	4/17 (7/24)	5/17 (15/43)	4/22 (24/109)	0/0 (0/0)	1/2 (5/16)	2/8 (10/36)	4/20 (9/43)	4/12 (12/43)	1/2 (5/27)	4/12 (8/21)	5/25 (15/66)

OM lipoproteins^b^	LIC10024	54/223	10/31	9/37	16/74	21/58	39/135	3/8	8/25	36/112	6/24	32/103	6/25	13/35	30/144
	LIC10647	24/88	0/0	10/31	6/27	10/39	14/57	0/0	1/5	8/24	1/5	10/30	2/9	1/1	15/74
	LIC10713	23/86	0/0	7/22	6/23	4/12	8/35	0/0	2/5	3/12	3/11	1/2	2/9	2/8	9/30
	LIC11003	12/36	1/2	0/0	0/0	1/3	4/18	0/0	0/0	4/16	3/12	0/0	1/1	5/17	8/21
	LIC11755	64/222	13/44	17/65	16/62	12/50	34/145	1/3	5/15	19/60	12/67	16/59	9/38	10/32	23/123
	LIC12048	102/331	13/54	20/75	23/85	27/85	38/142	3/4	8/31	18/61	18/69	15/62	10/54	8/28	19/77
	LIC13411	17/60	0/0	3/8	3/8	7/19	6/18	0/0	2/4	1/2	0/0	3/16	0/0	2/12	4/25
	LIC20172	22/101	3/9	7/26	10/31	4/16	10/32	0/0	1/2	8/30	8/30	1/1	3/12	3/8	7/32

*^a^The number of predicted surface-related 9mer (SRE9) and 15mer (SRE15) epitopes and the total number of strong binder 9mer (SB9) and 15mer (SB15) epitopes (including those that are not surface-related) are shown*.

*^b^For the OM lipoproteins, only SB9 and SB15 are shown*.

*^c^SB epitopes predicted by NetMHCII as CI50 <50 nM*.

## Discussion

The main principle of RV is to evaluate all the potential vaccine candidates encoded in the genome of the pathogen of interest and to reduce the number of vaccine targets to a number that can be reasonably tested in the laboratory (34). The initial screening is achieved by using bioinformatics to identify all surface-exposed proteins (potential vaccine candidates) and this typically reduces the number of targets 10-fold, from thousands to hundreds of proteins. Screening using *in vitro* assays further reduces the number of vaccine candidates and hence the number of laboratory animals required for efficacy testing. However, as no immune correlates for leptospirosis have been identified to date, the only way to screen the proteins identified as surface-exposed is to use a lethal animal model and look for protection (35). Therefore, the *in silico* identification of surface-exposed proteins must be sufficiently rigorous to screen out undesirable proteins, yet be sensitive enough to include all potential vaccine candidates.

In diderm bacteria, vaccine targets include transmembrane proteins (βb-OMPs), lipoproteins anchored to the outer leaflet of the OM, and possibly secreted proteins, particularly those that interact with the βb-OMPs. As there is no concrete evidence that *Leptospira* spp. secrete proteins, their role in pathogenesis is unclear, and therefore, there is no supporting data for their use as vaccine candidates. The prediction of the antiparallel transmembrane β-sheets that form βb proteins is usually achieved with a high degree of confidence (36). The identification of the SP, the βb secondary structure, the absence of TMHs, and homology to known proteins is a straightforward process. Lipoprotein prediction is based on the identification of a lipobox and localization to the OM is based on homology to known OM lipoproteins (37, 38). In the current study, 16 different bioinformatics programs were used to analyze the LIC proteome, one of the most studied *Leptospira* strains and one that is routinely used as the challenge strain in animal models of leptospirosis (7). A total of 165 βb-OMPs and 54 OM lipoproteins were identified in the first round of screening. However, the absence of experimental data for leptospiral proteins severely limits the use of protein sets with known subcellular localization and structures that can be used to verify the prediction based on true or false analyses. To overcome this limitation, we developed an algorithm that reduced the final target list of potential vaccine candidates to 18 βb-OMPs and 8 OM lipoproteins, each predicted with a high degree of confidence. The algorithm was intended to normalize, calculate a weight, and transform the data based on a measure of agreement among the predictors. This was achieved by giving higher weights to those predictors whose results were confirmed by other predictors and lower weights to those predictors with higher rates of disagreement; the result of the voting was expected to be more reliable, as confirmed in the current study. The bioinformatics pipeline and algorithm developed in this study, including the SV approach, can be applied to the identification of OMPs and vaccine candidates in any other diderm pathogen with a known genome sequence.

Structural vaccinology represents the cutting edge of vaccine target discovery and development. Several approaches have been investigated (29) although all are based on the common theme of protein structural data. However, most of these approaches use information from experimentally determined protein structures (e.g., X-ray diffraction), which it is time consuming and expensive, especially when compared to *in silico* modeling (39). In the last decade, protein structure modeling has improved enormously, as demonstrated by the critical assessment of protein structure prediction (CASP) experiments (40). I-TASSER, the modeling tool used for leptospiral βb-OMPs and OM lipoproteins structural modeling, was ranked number one for structure and function prediction in the most recent CASP experiments (40, 41). I-TASSER was shown to be as precise as structure solving by crystallography (42); it predicts structure models based on protein threading, even allowing model prediction for proteins with low sequence similarity to any other protein, as it considers sequence to structure fold recognition. Even though I-TASSER was expected to provide high-quality structural models, some of the models generated in the current study were considered poor quality, as expected for proteins with unknown functions (33). The overall quality of a protein structure prediction is indicative that it might not be similar to the template structures; however, the overall folding is representative of how a given protein folds. In the case of βb-OMPs, the most unreliable regions were the folded loops and plug domains (data not shown); however, the transmembrane β-sheets were well predicted, giving a spatial and structural insight into where the surface-exposed part of the protein is located. Therefore, poor quality models were included in the epitope prediction analysis and mapping of the SREs in the structure. Modeling was particularly important for uncharacterized proteins with no known functional domains and that had low or no similarity to other known proteins. Overall model quality can be improved by separating different domains and modeling them separately. However, this approach was not available for proteins with no functional domains and could have generated final models that did not represent the fully resolved structure (39). We generated structural models that included a βb for 17 of the 18 predicted βb-OMPs. LIC20087 was the exception and even though the structural model was highly similar to a pilin (43), it did not contain a βb structure. Although there were some low-quality scores for some of the structural models, all the predicted models had high TM-scores and low RMSDs and this allowed COFACTOR to predict function based on the structural model. Function prediction based on structure is a valuable alternative during an *in silico* investigation of protein function, particularly in cases where no other similarity was found.

Leptospiral βb-OMPs and OM lipoproteins represent ideal targets for vaccine development, they are surface-exposed, and therefore amenable to recognition by the host immune system. Substantial evidence supports the theory that a protective immune response is antibody-based (5); to date, only bovines have been shown to require a cellular immune response for protection (44). As leptospires are extracellular pathogens, phagocytosis plays an important role in the immune response, as pathogenic leptospires can resist complement-mediated killing (44). Following phagocytosis, proteins from the pathogen are processed by antigen-presenting cells and presented via MHC-II molecules to T-helper cells that can stimulate inflammation and activation of B-cells for antibody production (44). Therefore, we screened the novel βb-OMPs and OM lipoproteins for the presence of MHC-II SB epitopes. I-TASSER cannot predict protein structure in the context of the bacterial outer membrane. Therefore, the orientation of the OMPs models in the OM was determined by an interpretation of the orientation of the closest PDB structure from the COFACTOR analysis. We identified the location of the epitopes in the structural models of the βb-OMPs. These epitopes are likely to be exposed on the surface of the bacteria and therefore bind to MHC-II receptors, subsequently triggering an immune response. This approach is particularly relevant to βb-OMPs, these proteins usually contain long surface-exposed loops, but they also contain immunodominant regions that are not surface-exposed (Figure 2; Table 4; Table S6 in Supplementary Material). Therefore, recombinant vaccines should not use the entire βb-OMP in vaccine preparations to prevent stimulation of a non-protective immune response. The inclusion of only the surface-exposed MHC-II epitopes in the vaccine preparation would be more likely to stimulate a protective immune response. Each of the proteins included in the final target list will be discussed in detail in the following sections.

### LIC10496 and LIC12575 Are TolC-Like Proteins

LIC10496 was originally annotated as a hypothetical protein and LIC12575 was annotated as a cytoplasmic membrane protein. However, their orthologs were annotated as TolC-like OMPs in the other pathogenic *Leptospira* spp. Furthermore, functional annotation by InterProScan predicted that they contained an OM efflux protein domain (Table 1), a property of TolC proteins, and a component of Type I secretion systems. In diderm bacteria, TolC is an OM efflux protein that forms a trimeric channel composed of a 12-stranded βb that spans the OM (4 transmembrane β-sheets per monomer) and long periplasmic α-helices that span the periplasm. The channel is connected to the cytoplasm by a TMH inner membrane protein channel. TolC transporters are involved in the transport of a variety of substrates, from small molecules to protein secretion. TolC proteins from *Edwardsiella tarda* (45), *Listeria monocytogenes* (46), and *Salmonella paratyphi* (47) were reported to be protective vaccine antigens. The structural models of the leptospiral TolC proteins were highly similar to an *E. coli* TolC protein (48). Both were characterized as high-quality models, with high ModFOLD and QMEAN scores and the lowest number of disallowed residues on the Ramachandran plot (Table 3). Interestingly, the extracellular loops connecting the β-sheets in the βb were predicted to contain MHC-II epitopes. Both leptospiral TolC-like proteins were conserved in all *Leptospira* spp., except for *L. wolffii* that has no ortholog for LIC10496. Structure-based GO predictions for these proteins suggested a possible relation to copper ions, siderophores, or protein transport (Table S5 in Supplementary Material). A previous study linked LIC12575 to the leptospiral response when exposed to *in vivo* like conditions (49). These data support the idea that LIC10496 and LIC12575 are promising vaccine candidates.

### LIC10714, LIC10881, LIC10896, LIC10964, LIC12374, and LIC20151 Are TonB-Dependent Receptors

A TonB-dependent receptor (TBDR) domain was identified in all these proteins and this was supported by the structural models that predicted TBDRs. All the leptospiral TBDR models were reliable in terms of quality, this was expected as there is a high degree of similarity among TBDRs even in different bacteria (50). TBDRs are a βb proteins comprised of 22 amphipathic β-strands, and a globular plug domain that is folded up inside the barrel. Indeed, this structural folding was observed for LIC10881* and LIC10896* that were originally partial CDSs, supporting our finding that LIC10882 and LIC10895 are not proteins but are instead part of the LIC10881 and LIC10896 CDS, respectively, as in seen in other *Leptospira* spp. Interestingly, LIC10881* was absent in saprophytic and intermediate *Leptospira* spp., suggesting a potential role in pathogenesis. Gram-negative bacteria contain variable numbers of TBDRs, the *E. coli* genome contains 7 TBDRs (50); while other bacteria can contain up to 65 TBDRs (51). The LIC genome contains 13 genes that are annotated as TBDRs (52). While orthologs of LIC10714 in other *Leptospira* spp. genomes were annotated as TBDRs, LIC10714 was annotated as an OM receptor protein. LIC10714 contained the characteristic domains and the model included a 22-stranded βb with high structural similarity to FhuA from *E. coli* (53), even though it was suggested to be a leptospiral FecA (54). An ortholog of LIC10714 in *L. biflexa* was knocked out, resulting in a mutant with impaired ability to use iron citrate, iron chloride, iron sulfate, and aerobactin as iron sources, suggesting a role in iron metabolism (55). LIC10714 was also found to bind fibronectin (renamed as MFn2) and it was suggested that the extracellular loops could play a role in the bacterial adhesion process (56). The structural model of LIC10881* was similar to TbpA, a transferrin transporter in pathogenic *Neisseria* spp. (57). The LIC10896* 3D model was similar to FpvA, a pyoverdine-Fe transporter from *Pseudomonas aeruginosa* (58). LIC10964 was predicted to be a hemin transporter encoded by *phuR*, this gene is absent from all saprophytic *Leptospira* spp. except for *L. vanthielii* (Table 2). Of note, *phuR* was upregulated in the infection-mimicking model of leptospiral growth within dialysis membrane chambers (DMCs) implanted in the rat peritoneal cavity (59). The LIC10964 model was highly similar to the *N. meningitidis* zinc transporter, ZnuD (60). The LIC12374 and LIC20151 models were structurally similar to the *E. coli* BtuB cobalamin (vitamin B12) transporter (61). It has been suggested that pathogenic *Leptospira* spp. are autotrophic for vitamin B12 (12).

The actual molecule transported by a TBDR is difficult to predict by bioinformatics and should be determined experimentally. TBDRs play an important role in pathogenicity, they are conserved among the pathogenic *Leptospira* spp., and a significant portion of its structure was predicted to be surface-exposed. MHC-II epitopes were predicted for the TBDRs and several were located on the surface-exposed region of the protein. While TBDRs have been explored as vaccine candidates in other bacterial diseases (62–64), they have not yet been evaluated as experimental vaccines against leptospirosis. TBDRs transport essential molecules and if this is blocked, e.g., by antibodies, it would be a potentially lethal event.

### Are LIC11268 and LIC13477 Alginate Transporters?

Although both proteins were originally annotated as hypothetical proteins, InterProScan found an alginate export domain in LIC11268 (Table 1). However, the structural model of LIC11268 suggested it was a βb-OMP with high structural similarity to the neisserial TbpA transferrin transporter, a known TBDR (57). LIC13477 was identified as an alginate transporter based on structural annotation (Table 3) and was structurally similar to the OMP AlgE from *P. aeruginosa* (65). AlgE contains 18-stranded β-sheets in the transmembrane βb and is different to other TBDRs. The predicted models of both LIC11268 and LIC13477 contained an 18-stranded βb. Although an alternative, low quality, model of LIC11268 contained 20-stranded β-sheets (data not shown). Alginate is a polysaccharide in biofilms (66) and both *L. biflexa* and *L. interrogans* contain a complete set of genes for alginate biosynthesis (52, 67). Nevertheless, alginate exporters have not yet been identified in *L. interrogans*. The *in silico* modeling of LIC11268 and LIC13477 suggested they may play a role in the transport of alginate to the extracellular milieu, but this will need to be confirmed experimentally. Both proteins were predicted to contain a βb structure, potentially spanning the leptospiral OM. Immunogenic epitopes were identified in the surface-exposed regions and both proteins were conserved among pathogenic *Leptospira* spp., suggesting they are promising vaccine candidates.

### LIC11623 and LIC12254 Are BamA-Like βb-OMPs

Both proteins were originally annotated as OMPs, while in the current study, they were found to contain the bacterial surface antigen D15/Oma87 domains (Table 1). Of note, the orthologs of LIC11623 and LIC12254 in other *Leptospira* spp. were annotated as Oma87-like proteins. These BamA-like proteins are responsible for the assembly of βb-OMPs in Gram-negative bacteria. BamA is the OM component of the βb assembly machinery (Bam) complex and consists of a transmembrane βb and five polypeptide translocation-associated (POTRA) domains that extend into the periplasm. Both the βb and the five POTRA domains were identified in the LIC11623 model, and the closest PDB structure was BamA from *Neisseria gonorrhoeae* (68). Interestingly, although the LIC12254 model was similar to BamA, three of the POTRA domains (P1, P2, and P3) were missing. The closest PDB structure was *Hd*BamAΔ3 from *H. ducreyi*, it too lacks three POTRA domains (68). Immunization with recombinant D15/Oma87 induced protective immune responses against *Haemophilus influenza* (69) and *Pasteurella multocida* (70). In addition, LIC12254 was only found in two of the saprophytic *Leptospira* spp., suggesting a possible role in pathogenesis. Although LIC11623 was described as a BamA-like protein in *L. interrogans* (52, 54), neither of these proteins have been evaluated as a vaccine candidate.

### LIC11458 Is an LptD-Like Export Porin

LIC11458 was identified as LptD/OstA, a member of the OM LPS export porin (LPS-ep) family (Tables 1 and 3). In diderm bacteria, LptD is responsible for LPS assembly in the OM (71). The structural model of LIC11458 was similar to the *Shigella flexneri* LptD, containing a transmembrane βb and a periplasmic β-jellyroll domain (72). The predicted transmembrane architecture of this protein forms a large 26-stranded βb with immunogenic surface-exposed epitopes. LPS is an essential virulence factor in pathogenic *Leptospira* spp. and, in contrast to the highly variable LPS molecule, LIC11458 was conserved among *Leptospira* spp. An immune response directed against LIC11458, as well as stimulating opsonizing leptospires, could potentially impair LPS assembly, ultimately killing the bacteria.

### LIC11086, LIC11211, and LIC11506 Are Transport Proteins

LIC11086 was predicted to contain a MetA-pathway domain for phenol degradation. The structural model of LIC11086 was highly similar to the crystal structure of Pput2725, a protein from *Pseudomonas putida* F1, a microorganism that can biodegrade hydrocarbons in the environment (73). Both structures contained a 12-stranded barrel with an N-terminal segment preceding the first β-strand that blocks the barrel. Proteins with this domain are predicted to transport hydrophobic molecules through the membrane, usually trichlorophenol and some are relatively well characterized (74, 75). LIC11211 was originally annotated as a hypothetical protein. The structural model of LIC11211 resembled TodX, an aromatic hydrocarbon transporter, also from *P. putida* (76). TodX is a 14-stranded βb, with an N-terminal flexible hatch domain. Both Pput2725 and TodX are members of the FadL family, a transporter of hydrophobic molecules in *E. coli* (77). *P. putida* and other biodegradation bacteria, such as *Ralstonia pickettii*, have intracellular pathways for the degradation of toxic hydrocarbons that enter the cells via FadL, with structural changes in the hatch domain (76). The role of hydrophobic molecule transporters in *Leptospira* spp. is unknown. LIC11506 was predicted to contain an OMP domain that is restricted to the Leptospiraceae (*Leptospira* and *Leptonema*). The structural model of LIC11506 was similar to *E. coli* OmpG, a βb with 14 antiparallel β-strands involved in the transport of carbohydrates into the cell (78). All three predicted leptospiral βb-OMPs have orthologs in all pathogenic strains; however, the LIC11506 and LIC11211 orthologs were absent in two species of intermediate pathogenicity (Table 2).

### LIC20019 and LIC20087

Both proteins are encoded on chromosome II, LIC20019 was annotated as a hypothetical protein while LIC20087 was annotated as an OMP. However, functional annotation showed that while LIC20019 contained a putative OMP porin 6 domain, exclusive to *Leptospira* spp., no such domain was identified in LIC20087. Furthermore, LIC20019 was modeled as a perfect βb, while LIC20087 was the only protein among the βb-OMPs identified in the present study that did not contain a βb. The LIC20087 model displayed structural similarity to the Type II pilus protein PitB from *Streptococcus pneumoniae* (79). The presence of pili or their function in *Leptospira* spp. is unknown; however, it was predicted to be surface-exposed and represents a potential vaccine candidate. As it is not expected for pili to be intrinsic to the OM, LIC20087 was not evaluated for the presence of surface-exposed immunogenic epitopes. Whether LIC20087 was misidentified as a βb-OMP due to the presence of several β-sheets in the pilus subunit protein or due to a poor threading template model, will need to be further investigated. This protein was shown to be immunogenic during infection and a possible candidate for early leptospirosis diagnosis (80). LIC20019 was, however, modeled as an βb-OMP, with high structural similarity to the plasminogen activator Pla from *Yersinia pestis* (81). Pla is an OM protease (omptin) whose inactivation drastically reduces *Y. pestis* virulence (82). Omptins are widely distributed among Enterobacteriaceae and have several functions (83). Some leptospiral proteins have been reported to bind plasminogen, however, just a few were investigated for cellular localization. Besides an apparent redundancy in the extracellular component binding proteins in *Leptospira* spp., LIC20019 was predicted to be exposed on the bacterial surface and to be highly immunogenic, a potentially strong vaccine candidate.

### The OM Lipoproteins

Leptospiral lipoproteins that were previously shown to be protective in the hamster model of leptospirosis, such as LigA (LIC10465), LigB (LIC10464), and LemA (LIC11058) were not selected among the list of nine predicted OM lipoproteins in this work. While these proteins were predicted by both LipoP and SpLip as lipoproteins (Table S1 in Supplementary Material), they were not consistently predicted to be located in the OM by the localization predictors. LemA was included in the list of the 54 OM lipoproteins (Table S2 in Supplementary Material) as it was identified by Cello as an OM protein. These observations are indicative that the list of 54 OM lipoproteins and 165 βb-OMPs included potential vaccine candidates that should not necessarily be excluded from future studies.

Of the eight OM lipoproteins selected, LIC10024 was the only lipoprotein originally annotated as an AGC. LIC11003 was annotated as LipL71 and the remaining six proteins were annotated as conserved hypothetical proteins or putative lipoproteins. In the current study, LIC11003 and LIC20172 were identified as peptidoglycan-binding proteins LysM (also known as LruA) and LruC, respectively. LruA and LruC were previously described as leptospiral recurrent uveitis-associated proteins A, B, and C (84, 85). LruC was experimentally demonstrated to be an OM lipoprotein but was not exposed on the bacterial surface, it was located in the inner leaflet of the OM (84). In contrast, LruA was exposed on the leptospiral surface, with a possible role in the modulation of interactions with human apolipoprotein A-I (ApoA-I), contributing to leptospiral virulence (86). Furthermore, LruA was shown to be essential for *L. interrogans* virulence in the hamster model (86). None of the Lru proteins have been evaluated as vaccine candidates and in order to do so, it will be necessary to exclude the regions that are responsible for leptospiral-related uveitis. LIC10024 was originally annotated as a membrane bound AGC, with an undefined cellular location. Another leptospiral protein (LA4008/LIC13201) with an AGC domain was shown to have host cell cAMP-elevating activity (87). The role of AGC proteins in leptospiral pathogenicity and the location of LIC10024 in the cell remain to be determined. Following *in vitro* analysis, LIC13411 was identified as a leptospiral adhesin that binds to VE-cadherin, an endothelial cell receptor (88). LIC13411 was also demonstrated to be present in the OM, supporting the findings of the current study. Unlike the βb-OMPs, the structural models of the OM lipoproteins could not be used to predict function or localization. The best matches to PDB structures included three eukaryotic proteins and a viral protein (Table 3). Most of the PDB structures identified were not bacterial surface-related proteins. This could be due to the absence of known conserved domains and low similarity to known protein structures. However, all eight lipoproteins were predicted to be OM lipoproteins and were highly conserved among *Leptospira* spp., including several immunogenic epitopes, suggesting they could be potential vaccine candidates.

## Conclusion

We report the discovery of 26 new vaccine candidates using an innovative approach that represents the most extensive bioinformatics-based screening of vaccine targets in the field of leptospirosis and, to the best of our knowledge, is the first report using SV. The bioinformatics approach developed in this work can be applied to other diderm pathogens. The proteins identified in the current study are novel and have not yet been evaluated as vaccine candidates. We identified proteins that are likely to be functionally involved in diverse pathways, many related to pathogenesis, including iron and vitamin transport, OMP, and LPS assembly. The inhibition of these proteins by a host immune response will likely impair these essential pathways. Our group is currently evaluating a new experimental approach to confirm the subcellular location of these proteins in the leptospiral cell. Of the proteins identified in the present study, those that are surface-exposed will be evaluated as vaccine candidates in the hamster model of leptospirosis.

## Materials and Methods

### Genome Sequence Retrieval

The genome-derived proteome (chromosomes I and II) of *L. interrogans* serogroup Icterohaemorrhagiae serovar Copenhageni strain Fiocruz L1-130 was downloaded from the *Leptospira* Genome Project website (http://aeg.lbi.ic.unicamp.br/world/lic) in FASTA format, corresponding to GenBank accession numbers AE016823.1 and AE016824.1, respectively. High-quality draft, improved high-quality draft, or complete genomes for 20 additional *Leptospira* spp. (Table S4 in Supplementary Material) were obtained from GenBank.

### Prediction of Primary and Secondary Structure Features

The amino acid sequences corresponding to all 3,773 CDS in both LIC chromosomes were used as the input sequences for 16 bioinformatics programs for the identification of βb integral transmembrane proteins and OM-associated lipoproteins. Subcellular localization was predicted using PSORTb v. 3.0.2 (89), CELLO v. 2.5 (90), and GNeg-mPLoc v. 2.0 (91). The presence of a SP was predicted using SignalP v. 4.1 (92), Signal-CF (93), and PrediSi (94). TMHs were predicted using MEMSAT3 (95), TMHMM v. 2.0 (96), HMMTOP v. 2.0 (97), and Phobius (98). Proteins with more than one TMH (cut-off <2 TMH) were excluded from further analysis. Proteins with a single TMH, especially when located at the N-terminus, were included as a predicted SP can be confused with a TMH. The βb-OMPs were predicted using HHomp (99), BOMP (100), MCMBB (101), and TMBETADISC-RBF (102). Leptospiral lipoproteins were predicted using LipoP v. 1.0 (38) and SpLip (37). Each program was used with the default settings and when available the option for Gram-negative bacteria was selected. Sequence data and results retrieval for each predictor was automated using Python scripts whenever possible.

### Filtering Predicted Protein Features with Increased Confidence

Low agreement is expected between different bioinformatics tools when predicting OMPs (103). To reduce the impact of a prediction when naïve (unweighted) voting would result in ambiguities (e.g., two negative results and two positive results), we used an iterative weighted voting system. A Python script was used to integrate the results, increasing confidence in the consensus prediction of a protein feature identified by more than one predictor. The Consensus by Voting with Iterative Re-weighting based on Agreement (CoVIRA) algorithm, script, and several examples are available on a GitHub repository (https://github.com/biopro/covira) and the algorithm followed the logic:

$${\text{PtAS}}_{j}=\sum_{i=1}^{\text{NPd}-1}\left(V_{i}\times {\text{PdW}}_{i}\right),$$

where PtAS*_j_, Protein Agreement Score*—the value per protein by a given predictor.

j=1,2,…, NPt

NPt—the *total number of proteins* evaluated.

*V_i_, Vote*, always 1 or 0—one (1) when both predictors were in agreement, zero (0) for no agreement.

i=1,2,…,NPd

PdW*_i_, predictor weight*—the calculated weight of each predictor in the total number of votes per protein, varied by iteration.

i=1,2,…,NPd

NPd—total number of predictors used per protein feature.

In the first iteration, PdW was

$${\text{PdW}}_{i}=\frac{1}{\text{NPd}}.$$

From the second to the 1,000 iteration, the PdW for the next iteration was

$${\text{PdW}}_{i}=\frac{\bar{{\text{PtAS}}_{i}}}{\left|\text{max}\left\{\bar{{\text{PtAS}}_{1}},\ldots ,\bar{{\text{PtAS}}_{\text{NPd}}}\right\}-\text{min}\left\{\bar{{\text{PtAS}}_{1}},\ldots ,\bar{{\text{PtAS}}_{\text{NPd}}}\right\}\right|},$$

where $\bar{{\text{PtAS}}_{i}}$—the arithmetic mean per protein per predictor:

$$\bar{{\text{PtAS}}_{i}}=\frac{\sum {\text{PtAS}}_{j}}{\text{NPt}}.$$

A predictor with low accuracy was expected to generate a low agreement score and as accuracy improved, so would the agreement score. Finally, the last vote, based on the final weight of each predictor, was performed to identify those βb-OMPs and OM lipoproteins with a high confidence prediction. A final vote value >0.5 (0–1 scale) for each feature of interest (SP, OM localization, etc.) was considered as a confident prediction, and these proteins were selected for further analysis. The voting algorithm was validated using a set of proteins with and without an SP. The presence of an SP was evaluated using SignalP, PreDiSi, and SignalCF. The results were analyzed by naïve voting, each predictor had an equal weight. Based on the same results, CoVIRA was executed to calculate a final score and prediction for each protein. The results were analyzed by a receiver operating characteristic (ROC) curve, and the CoVIRA prediction was improved compared to the naïve voting (see Data Sheet S2 in Supplementary Material).

### Identification of *Leptospira* spp. Orthologs and Similar Mammalian Host Proteins

Orthologs to the selected proteins were identified in the leptospiral genome sequences (Table S1 in Supplementary Material) using the reciprocal best hit (RBH) method based on protein BLAST (BLASTp) searches. Protein sequences with >70% of similarity and >40% coverage that were also the best reciprocal hit were considered orthologs. A multiple sequence alignment was performed among orthologs using the online MUSCLE tool (MUltiple Sequence Comparison by Log-Expectation) (104). The βb-OMPs and OM lipoproteins with orthologs in other pathogenic *Leptospira* spp. were screened against the human, bovine, canine, equine, ovine, and swine genomes using the online Blastp server (taxIDs: *Homo sapiens* 9606, *Bos taurus* 9913, *Canis familiaris* 9615, *Equus caballus* 9796, *Ovis aries* 9940, and *Sus scrofa domesticus* 9825). Leptospiral proteins with >40% similarity to any host proteins were excluded from the final target list of potential vaccine candidates.

### Structural Modeling of Predicted Surface-Exposed Leptospiral Proteins

The 3D structures of conserved βb-OMPs and OM lipoproteins were generated by protein threading. Prior to modeling, the SP sequence was manually removed from the final FASTA amino acid sequence. βb-spanning OM proteins and OM lipoprotein structures were predicted using the I-TASSER server (41, 42). I-TASSER predicted up to five alternative models and the model with the higher *C*-Score was refined using ModRefiner (105). Quality assessment of models was performed using ModFold4 (106), Qmean6 (107), and Procheck (108). ModFold and Qmean returned a score (0–1) that inferred the overall quality of the structure. ModFold also assigned a *P*-value and a degree of confidence (poor, low, medium, high, and cert) to the model. Procheck was used to analyze the stereochemistry of the refined model by evaluating the Ramachandran plot of each protein structure (109). The 3D structures were visualized using UCSF Chimera (110) and PyMol (111).

### Sequence and Structural Functional Annotation

Functional annotation was performed using the InterProScan tool (112, 113). In addition, the UniProt Knowledgebase was screened using the locus tag (LIC number) to identify functional information and to access the annotation in other *Leptospira* spp. by KEGG database. In addition to the primary amino acid sequence analysis, a 3D structure-based functional annotation was performed using COFACTOR (114), to identify the PDB structures with the closest structure to the target protein model, and assigned GO terms for the protein model based on these PDB structures.

### Epitope Prediction and OMP Structural Allocation

The presence of MHC-II linear epitopes in the amino acid sequence of the selected proteins was predicted using NetMHCII v. 2.2 (115, 116). Fourteen HLA-DRB alleles were used in the prediction of immunogenic epitopes for each protein, including the most frequent alleles in human populations. The location of SB immunogenic epitopes (threshold values of IC50 <50 nM) were determined in the 3D structures of the OMPs, those most likely located within the surface-exposed portion of the OMPs were selected. The orientation of the OMPs models in the OM was determined by an interpretation of the orientation of the closest PDB structure from the COFACTOR analysis. The predicted MHC-II epitopes were aligned to the corresponding region in the ortholog proteins from other pathogenic *Leptospira* spp. (Table 2) by multiple sequence alignment generated by MUSCLE, and sequence logos were generated for each epitope using WebLogo (117). Images of the structural models, highlighting the SREs were generated by PyMol.

## Author Contributions

AG and AM designed the study and wrote the manuscript. FK wrote the Python scripts and automated software input whenever possible. AG, FK, JDS, and JCS performed the bioinformatics analysis. AG and JDS created the figures and tables. AG, FK, JDS, JCS, and LP analyzed data. All authors contributed to and revised the manuscript.

## Conflict of Interest Statement

The authors declare that the research was conducted in the absence of any commercial or financial relationships that could be construed as a potential conflict of interest.

## Abbreviations

βb, β-barrel; βb-OMP, β-barrel transmembrane proteins; CDS, coding sequence; GO, Gene Ontology; Lig, Leptospiral immunoglobulin-like; LIC, *Leptospira interrogans* serovar Copenhageni strain Fiocruz L1-130; MHC, major histocompatibility complex; OM, outer membrane; OMP, outer membrane protein; POTRA, polypeptide translocation-associated domain; RMSD, root-mean-square deviation; RV, reverse vaccinology; SP, signal peptide; SV, structural vaccinology; TBDR, TonB-dependent receptor; TMH, transmembrane α-helix.
