# Supplementary material for: Discovery of Novel Leptospirosis Vaccine Candidates Using Reverse and Structural Vaccinology
Source: Front Immunol. 2017 Apr 27;8:463. doi: 10.3389/fimmu.2017.00463 (PMC5406399; doi:10.3389/fimmu.2017.00463)
Supplement: Supplementary file 3 [file Table_3.DOCX]

| **Supplementary Table S3.** Final weight in the Python voting algorithm. | |
| --- | --- |
| **Predictor** | **Final weight in the voting algorithm^1^** |
| PSORTb | 0.464 |
| Gneg-mPLoc | 0.411 |
| Cello | 0.125 |
| SignalCF | 0.360 |
| PrediSi | 0.345 |
| SignalP | 0.296 |
| HHomp | 0.302 |
| MCMBB | 0.296 |
| Bomp | 0.276 |
| TMBETADISC-RBF | 0.126 |
| TMHMM | 0.256 |
| Phobius | 0.253 |
| Memsat | 0.249 |
| HMMTOP | 0.242 |
| LipoP | 0.500 |
| SpLip | 0.500 |

^1^After 1000 iterations.
