# Supplementary material for: Discovery of Novel Leptospirosis Vaccine Candidates Using Reverse and Structural Vaccinology
Source: Front Immunol. 2017 Apr 27;8:463. doi: 10.3389/fimmu.2017.00463 (PMC5406399; doi:10.3389/fimmu.2017.00463)
Supplement: Supplementary file 4 [file Table_4.DOCX]

| **Supplementary Table S4**. *Leptospira* spp. genome sequences used in this work. | | | | | |
| --- | --- | --- | --- | --- | --- |
| **Species** | **Serovar** | **Strain** | **Lifestyle** | **GenBank Accession** | **Reference** |
| *L. alexanderi* | Manhao 3 | L 60 | pathogenic | AHMT00000000.2 | (1) |
| *L. alstonii* | Pingchang | 80–412 | pathogenic | AOHD00000000.2 | (1) |
| *L. borgpetersenii* | Javanica UI | 09931 | pathogenic | AHNP00000000.2 | (1) |
| *L. interrogans* | Copenhageni | Fiocruz L1-130 | pathogenic | AE016823/4 | (2) |
| *L. kirschneri* | Cynopteri | 3522 C | pathogenic | AHMN00000000.2 | (1) |
| *L. kmetyi* | Malaysia | Bejo-Iso9 | pathogenic | AHMP00000000.2 | (1) |
| *L. mayottensis* | - | 200901116 | pathogenic | AKWB00000000.2 | (3) |
| *L. noguchii* | Panama | CZ 214 | pathogenic | AKWY00000000.2 | (1) |
| *L. santarosai* | Shermani | 1342K | pathogenic | AOHB00000000.2 | (4) |
| *L. weilii* | undetermined | LNT 1234 | pathogenic | AHNC00000000.2 | (1) |
| *L. broomii* | Hurstbridge | 5399 | intermediate | AHMO00000000.2 | (1) |
| *L. fainei* | Hurstbridge | BUT 6 | intermediate | AKWZ00000000.2 | (1) |
| *L. inadai* | Lyme | 10 | intermediate | AHMM00000000.2 | (1) |
| *L. licerasiae* | Varillal | VAR 010 | intermediate | AHOO00000000.2 | (5) |
| *L. wolffii* | Khorat | Khorat-H2 | intermediate | AKWX00000000.2 | (1) |
| *L. biflexa* | Patoc | Patoc I (Paris) | saprophytic | CP000786/7 | (6) |
| *L. meyeri* | Hardjo | Went 5 | saprophytic | AKXE00000000 | (1) |
| *L. terpstrae* | Hualin | LT 11–33 | saprophytic | AOGW00000000.2 | (1) |
| *L. vanthielii* | Holland | WaZ Holland | saprophytic | AOGY00000000.2 | (1) |
| *L. wolbachii* | Codice | CDC | saprophytic | AOGZ00000000.2 | (1) |
| *L. yanagawae* | Saopaulo | Sao Paulo | saprophytic | AOGX00000000.2 | (1) |

**Supplementary References:**

1. Fouts DE, Matthias MA, Adhikarla H, Adler B, Amorim-Santos L, Berg DE, et al. What Makes a Bacterial Species Pathogenic?:Comparative Genomic Analysis of the Genus Leptospira. *PLoS Negl Trop Dis* (2016) **10**(2):e0004403. doi: 10.1371/journal.pntd.0004403. PubMed PMID: 26890609; PubMed Central PMCID: PMCPMC4758666.

2. Nascimento AL, Ko AI, Martins EA, Monteiro-Vitorello CB, Ho PL, Haake DA, et al. Comparative genomics of two Leptospira interrogans serovars reveals novel insights into physiology and pathogenesis. *J Bacteriol* (2004) **186**(7):2164-72. PubMed PMID: 15028702; PubMed Central PMCID: PMCPMC374407.

3. Bourhy P, Collet L, Brisse S, Picardeau M. Leptospira mayottensis sp. nov., a pathogenic species of the genus Leptospira isolated from humans. *Int J Syst Evol Microbiol* (2014) **64**(Pt 12):4061-7. doi: 10.1099/ijs.0.066597-0. PubMed PMID: 25249563.

4. Chou LF, Chen YT, Lu CW, Ko YC, Tang CY, Pan MJ, et al. Sequence of Leptospira santarosai serovar Shermani genome and prediction of virulence-associated genes. *Gene* (2012) **511**(2):364-70. doi: 10.1016/j.gene.2012.09.074. PubMed PMID: 23041083.

5. Ricaldi JN, Fouts DE, Selengut JD, Harkins DM, Patra KP, Moreno A, et al. Whole genome analysis of Leptospira licerasiae provides insight into leptospiral evolution and pathogenicity. *PLoS Negl Trop Dis* (2012) **6**(10):e1853. doi: 10.1371/journal.pntd.0001853. PubMed PMID: 23145189; PubMed Central PMCID: PMC3493377.

6. Picardeau M, Bulach DM, Bouchier C, Zuerner RL, Zidane N, Wilson PJ, et al. Genome sequence of the saprophyte Leptospira biflexa provides insights into the evolution of Leptospira and the pathogenesis of leptospirosis. *PLoS One* (2008) **3**(2):e1607. doi: 10.1371/journal.pone.0001607. PubMed PMID: 18270594; PubMed Central PMCID: PMCPMC2229662.
