# Supplementary material for: Discovery of Novel Leptospirosis Vaccine Candidates Using Reverse and Structural Vaccinology
Source: Front Immunol. 2017 Apr 27;8:463. doi: 10.3389/fimmu.2017.00463 (PMC5406399; doi:10.3389/fimmu.2017.00463)
Supplement: Supplementary file 8 [file Data_Sheet_1.ZIP › Alignment Bb-OMPs/Alignment Information_Bb-OMPs.docx]

**Supplementary Data Sheet S1.** Multiple sequence alignment performed by Muscle for the 18 βb-OMPs and their respective orthologs in pathogenic *Leptospira* spp. Immunogenic MHC-II surface-related epitopes are highlighted (yellow - *L. interrogans* protein, green - orthologs). Each file contains the alignment of one protein, identified by the LIC gene ID in the file name. Orthologs are identified by their name (genome annotation), preceded by the *Leptospira* spp., identified as follow: L_inte = *L. interrogans*; L_alex = *L. alexanderi*; L_alst= *L. alstonii*; L_borg = *L. borgpetersenii*; L_kirs = *L. kirschneri*; L_kmet = *L. kmetyi*; L_mayo = *L. mayottensis*; L_nogu = *L. noguchii*; L_sant = *L. santarosai*; L_weil = *L. weilii*. The tryptophan codon included in the modified LIC10881 amino acid sequence is conserved among the orthologues of this protein in *Leptospira* spp. and is highlighted in turquoise.
