# Supplementary material for: Discovery of Novel Leptospirosis Vaccine Candidates Using Reverse and Structural Vaccinology
Source: Front Immunol. 2017 Apr 27;8:463. doi: 10.3389/fimmu.2017.00463 (PMC5406399; doi:10.3389/fimmu.2017.00463)
Supplement: Supplementary file 8 [file Data_Sheet_1.ZIP › Alignment Bb-OMPs/Mult_alignment_LIC10496_path_spp_orthol_immun_epit_highlighted.docx]

L_kmet_LEP1GSC052_4103 ------------------------------------------------------------

L_alst_LEP1GSC193_3818 ------------------------------------------------------------

L_sant_LEP1GSC048_2963 ------------------------------------------------------------

L_mayo_LEP1GSC190_0013 ------------------------------------------------------------

L_weil_LEP1GSC086_2122 MIFYKIHNHNLWPQGVYFKKPQKQFRLFWTSLHFALRFECVFFLRKKPKINTIYIKKALK

L_borg_LEP1GSC103_0021 ------------------------------------------------------------

L_alex_LEP1GSC062_4247 ------------------------------------------------------------

L_inte_LIC10496 ------------------------------------------------------------

L_kirs_LEP1GSC049_3438 ------------------------------------------------------------

L_nogu_LEP1GSC059_0339 ------------------------------------------------------------

L_kmet_LEP1GSC052_4103 --------------MLFLYSFGKPLNGTAGENLILDLRKSEDIAVENSPDVRLLGSQQMI

L_alst_LEP1GSC193_3818 -------MFARFIVLLLCVCTGI--DASERKTLRINLETATQIALTNYYLLLSLKNKNAA

L_sant_LEP1GSC048_2963 ----------MFACFIFLLCSGIEVRASDKKTLRVNLETATQIALTNYYLLLSLKNKNTA

L_mayo_LEP1GSC190_0013 ------------------------------------METATQIALTNYYLLLSIKNKNNA

L_weil_LEP1GSC086_2122 AILPDSVLFAGFIFLLFCVCPRIEIRASDKKTLRINLETATQIALTNYYLLLSLKNKNTA

L_borg_LEP1GSC103_0021 ------------------------------------------------------------

L_alex_LEP1GSC062_4247 ---------MGVLVSNTLFSNPENKKKVEEAKIKINLEKAVLIGTTNSILLRTLEARKEI

L_inte_LIC10496 MKRKYHTLILGLMFLTLFFSSKIRSQTTKQPPIKITLDQAVLIGSSNSVVLKVLEAKKEV

L_kirs_LEP1GSC049_3438 MKRKYHTLILGLALLMSFFSSKIRSESVKQPPIKITLDQAVLIGSSNSVVLKVLEAKKEV

L_nogu_LEP1GSC059_0339 MKRKYHTLILSLALLMPFFSSKVRSESVKQPPIKITLDQAVLIGSSNSVVLKVLEAKKEV

L_kmet_LEP1GSC052_4103 KSLIVKESWRTYFPTASVSWFRNTNVVENESDSRSQRIALNVDQVIFDGGRRSLALQAAL

L_alst_LEP1GSC193_3818 IKELISERWRDLLPTLGVNMTRQRYIIQDSNDYIYNAILLNVDQIVYDGGKKKLDLDIAQ

L_sant_LEP1GSC048_2963 IKELISERWRDLLPTLGVNMTRQRYIIQDSSDYIYNAILLNVDQIIYDGGKKKLDLDIAQ

L_mayo_LEP1GSC190_0013 IKELISERWRDLLPTLGVNMTRQRYIIQDSSDYIYNAILLNVDQIIYDGGKKKLDLDIAQ

L_weil_LEP1GSC086_2122 IKELISERWRDLLPTLGVNMTRQRYIIQDSNDYIYNAILLNVDQIIYDGGKKKLDLDIAQ

L_borg_LEP1GSC103_0021 -------------------MTRQRYIIQDSNDYIYNAILLNVDQIIYDGGKKKLDLDIAQ

L_alex_LEP1GSC062_4247 FKMLVTERWREFLPKVGIQYLGLRNVNINSLDNIYNDVRLTVQQLVYDGGEAKLNLETAN

L_inte_LIC10496 SKMLITEKWREFLPKFGIQYYGLRNQNVNSADNIYNDIRLTVQQLIFDGGEANLNLEIAK

L_kirs_LEP1GSC049_3438 SKMLITEKWREFLPKFGIQYYGLRNQNVNSADNIYNDIRLTVQQLIFDGGEANLNLEIAK

L_nogu_LEP1GSC059_0339 SKMLITEKWREFLPKFGIQYYGLRNQNVNSADNIYNDIRLTVQQLIFDGGEANLNLEIAK

:. * : : *.*:*:::*** .* *: *

L_kmet_LEP1GSC052_4103 NDLNLSKFDFLISINNLKLKVRNAYYALLSNKAQLEIQARSIQRQKDQLRFSQRELKLGE

L_alst_LEP1GSC193_3818 LEEILTREDFKITSSKVRLEVEKTFINTLVALAKVALNKKSVERAKEQLRLAKLEAKLGF

L_sant_LEP1GSC048_2963 LEEILSREDFKITSSKVRLEVEKTFINTLVALAKVALNKKSVERAKEQLRLAKLEAKLGF

L_mayo_LEP1GSC190_0013 LEEILSREDFKITSSKVRLEVEKTFINTLVALAKVALNKKSVERAKEQLRLAKLEAKLGF

L_weil_LEP1GSC086_2122 LEEILSREDFKITSSKVRLEVEKNFINTLVALAKVALNKKSVERAKEQLRLAKLEAKLGF

L_borg_LEP1GSC103_0021 LEEILSREDFKITSSKVRLEVEKTFINTLVALAKVALNKKSVERAKEQLRLAKLEAKLGF

L_alex_LEP1GSC062_4247 LNEALNEKDFKIGFYKLKYEIEKNYFKALAAKGKVFIGKKSIEKMEEASRKAQTEYKQGF

L_inte_LIC10496 LSELLNEQDFKINLSRLRLDIQKAYFRALALKGKVYIQKKAQEKAQEALRKGQVELRQGF

L_kirs_LEP1GSC049_3438 LSELLNEQDFKINLSRLRLDIQKAYFRALALKGKVFIQKKAQEKAQEALRKGQVELRQGF

L_nogu_LEP1GSC059_0339 LSELLNEQDFKINLSRLRLDIQKAYFRALALKGKVFIQKKAQEKAQEALRKGQVELRQGF

. *. ** * .:. .: : : * .:: : .: :. :: * .: * . *

L_kmet_LEP1GSC052_4103 TTEVQLLQIENRLNEIILQNKRTETAYQSGIEEFKILLRLPSSTKLALSADILHGIKFAY

L_alst_LEP1GSC193_3818 TTQIQVLSIASRLQEIEFSLVKSVNEYLKAKNDLKLAMSLDHLSDLVIEGDLLTDFYISY

L_sant_LEP1GSC048_2963 TTQIQVLSVASRLQEIEFALVKSISEYLKAKNDLKLVMSLDHQSDLVIEGNLLTDFYLSY

L_mayo_LEP1GSC190_0013 TTQIQVLSVASRLQEIEFALVKSINEYLKAKNDLKLIMSLDHQSDLIIEGNLLTDFYLSY

L_weil_LEP1GSC086_2122 TTQIQVLSVASRLQEIEFALVKSVNEYLKAKNDLKLVMSLDHQSDLIIEGNLLTDFYLSY

L_borg_LEP1GSC103_0021 TTQIQVLSVASRLQEIEFALVKSINEYLKAKNDLKLVMSLDHQSDLVIEGNLLTDFYLSY

L_alex_LEP1GSC062_4247 ITKIALLETVSKLRQSQYIFQKYENEYNQSIHDLKQILSIDYDSELEIEENLFTDFVMTL

L_inte_LIC10496 ITKVQLMDLESKLKQTEFNVQKSKNDSDQALLDLKQVMNLDYYAEIELNESIFFDFIINA

L_kirs_LEP1GSC049_3438 ITKVQLMDLESKLKQTEFNVQKSKNDSDQALLDLKQVMNLDYYAEIELNESIFFDFIINA

L_nogu_LEP1GSC059_0339 ITKVQLMDLESKLKQTEFNVQKSKNDSDQALLDLKQVMNLDYYAEIELNESIFFDFIINA

*:: ::. ..*.: . . .. ::* : : :.: :. .:: .: :

L_kmet_LEP1GSC052_4103 K--ELSLENLVGLAFQSRIEFARTRAAEIQTTSEYEIAKSFYIPTVSVGGFYASSGDRYD

L_alst_LEP1GSC193_3818 P--DFKKDTLIANAQNQRSEIVRIKINNKKLAKERELAENAWIPQISVGGSYGRTGVTYP

L_sant_LEP1GSC048_2963 P--DFKRETLIENAQNQRSEVVRIKINNKKLAKERELAENAWIPQISIGGSYGRTGVTYP

L_mayo_LEP1GSC190_0013 P--DLKRETLIENAQNQRSEIVRIKINNKKLAKERELAENAWIPQISIGGSYGRTGITYP

L_weil_LEP1GSC086_2122 P--DLKKETLIENAQNQRSEIVRIKINNKKLAKERELAENAWIPQISVGGSYGRTGITYP

L_borg_LEP1GSC103_0021 P--DLKRETLIENAQNQRSEVVRIKINNKKLAKERELAENAWIPQISVGGSYGRTGITYP

L_alex_LEP1GSC062_4247 P--NLEIRNIVDNARRQRDDIAKSQIVVQKLKNDKEIADNYWMPKLYLGGYAGKNGDTFP

L_inte_LIC10496 PPNTHNLDELISKAKNGREDLKKMQIIVKKLKNEKEVLDNQYMPKVYVGAYAGRNGNNNQ

L_kirs_LEP1GSC049_3438 PPNTHNLDELISKAKNGREDLKKMQIIVKKLKNEKEVLDNQYMPKVYVGAYAGRNGNNNQ

L_nogu_LEP1GSC059_0339 PPNTHNLDDLIFKAKNGREDLKKMQIIVKKLKNEKEILDNQYMPKFYVGAYAGRNGNNNQ

. :: * . * :. . . : .: *: .. ::* . :*. . .*

L_kmet_LEP1GSC052_4103 PKQREYGFNFKFSMALGPNSIQDTSNF-ISRNEDANRSLTS--------------TTTVG

L_alst_LEP1GSC193_3818 LQNDTWNVNVKVVFPLGGSTNTTTENLGMRYNNQASSGFAG----TVNPNFNAATSNNLQ

L_sant_LEP1GSC048_2963 LQNDTWNMNVKVVFPLGGSTNTTTENLGMRYNNQASSGFAG----TVNPNFNAATSNNLQ

L_mayo_LEP1GSC190_0013 LQNDTWNVNVKVIFPLGGSTNTTTENLGMRYNNQASSGFAG----TVNPNFNAATSNNLQ

L_weil_LEP1GSC086_2122 LQNDTWNVNVKVVFPLGGSTNTTTENLGMRYNNQASSGFAG----TVNPNFNAATSNNLQ

L_borg_LEP1GSC103_0021 LQNDTWNVNVKVVFPLGGSTNTTTENLGMRYNNQASSGFAG----TVNPNFNAATSNNLQ

L_alex_LEP1GSC062_4247 LQHNIYGVNFNITLPLGSNVVQSTGSSGIQKDGTGIQTYPGFGNQFVGSGLNGYNSTNVQ

L_inte_LIC10496 FTHDSYGVNFNLVMPLGSSVVQSNGNTGVQKDGNGIQTYPGFGNQTVGPGTNSYNSTSVR

L_kirs_LEP1GSC049_3438 FTHDSYGVNFNLVMPLGSSVVQSNGNTGVQKDGNGIQTYPGFGNQTVGPGTNSYNSTSVR

L_nogu_LEP1GSC059_0339 FTHDSYGVNFNLVMPLGSSVVQSNGNTGVQKDGNGIQTYPGFGNQTVGPGTNSYNSTSVR

: :..*.:. :.** . . . : : . .. :..:

L_kmet_LEP1GSC052_4103 IMDNMQYKRKIAQTGIAAEQAQITRRQLDDIIRIEVSKALQNYKLTWESLKLADENAKVF

L_alst_LEP1GSC193_3818 VLDNMSYSRKVMESKIKMGDSIAEKKRLEQSIAIEVDKASDVVKESYDLITIGSGMVYFR

L_sant_LEP1GSC048_2963 VLDNMSYSRKVMESKIKLGDSIAEKKRLEQSIAIEVEKASDVVKESYDLITIGSGMVYFR

L_mayo_LEP1GSC190_0013 ILDNMSYSRKVMESKIKLGDSIAEKKRLEQSIAIEVEKASDVVKESYDLITIGSGMVYFR

L_weil_LEP1GSC086_2122 ILDNMSYSRKVMESKIKLGDSIAEKKRLEQSIAIEVEKASDVVKESYDLITIGSGMVYFR

L_borg_LEP1GSC103_0021 VLDNMSYSRKVMESKIKLGDSIAEKKRLEQSIAIEVEKASDVVKESYDLITIGSGMVYFR

L_alex_LEP1GSC062_4247 FFNNLSYSRKIVEGEVQLSEALLNYRALENQVGTEIRKSYDKINESWGLIRIANSRVLLQ

L_inte_LIC10496 LFDNLSQSRKAMEGEIQLAEALLNYRNMENQVGFEIKKSVDKLNQSWELINIANSRINLQ

L_kirs_LEP1GSC049_3438 LFDNLSQSRKAMEGEIQLAEALLNYRNMENQVGFEIKKSVDKLNQSWELINIANSRINLQ

L_nogu_LEP1GSC059_0339 LFDNLSQSRKAMEGEIQLAEALLNYRNMENQVGFEIKKSVDKLNQSWELINIANSRINLQ

.::*:. .** : : :: . ::: : *: *: : : :: : :.. .

L_kmet_LEP1GSC052_4103 EKRLLIKEKQVSLGDARRTDLAETEIFYLEAINTMISSRVQYLTAVSQLEMAIGASLDSL

L_alst_LEP1GSC193_3818 YESMRLMNTKIQVGEAKRSDILFAETELVGAQEKLVDAIGKYCTSVYELEWVSGLQPDSL

L_sant_LEP1GSC048_2963 YESMRLMNTKIQVGEAKRSDILFAETELVGAQEKLVDAIGKYCTSVYELEWVSGLQPDSL

L_mayo_LEP1GSC190_0013 YESMRLMGTKIQVGEAKRSDILFAETELVGAQEKLVDAIGKYCTSVYELEWVSGLQPDSL

L_weil_LEP1GSC086_2122 YESMRLMNTKIQVGEAKRSDILFAETELVGAQEKLVDAIGKYCTSVYELEWVSGLQPDSL

L_borg_LEP1GSC103_0021 YESMRLMNTKIQVGEAKRSDILFAETELVGAQEKLVDAIGKYCTSVYELEWVSGLQPDSL

L_alex_LEP1GSC062_4247 WEALKIATVKHGIGHAKKEDLLSIELEFLKAQEDLTDALSSYAIHCTELSYLGRIDLNTS

L_inte_LIC10496 VESGRAMAAKVAYGHAKKEDQINSELEMIKSQEDLTDALTSYAINCYEYAQITTDEGGLR

L_kirs_LEP1GSC049_3438 VESGRAMAAKVAYGHAKKEDQINSELEMIKSQEDLTDALTSYAINCYEYAQVTTDEGGLR

L_nogu_LEP1GSC059_0339 VESGRAMAAKVAYGHAKKEDQINSELEMIKSQEDLTDALTSYAINCYEYAQVTTDEGGLR

: : * *.. * * : : : : .: .* : . .

L_kmet_LEP1GSC052_4103 ELIKTGKN----------------------------------------------------

L_alst_LEP1GSC193_3818 KLFNYKPNHGNTILPLILQNRPIPKSKVSENFKVKDIEEYFNSSDTDLEPDGGLLDAYDP

L_sant_LEP1GSC048_2963 KLFKYKPNHGNTILPLILQNRPISKSKAVESFKVKDIEEYFNSPDTDSETDKGLLDAYDP

L_mayo_LEP1GSC190_0013 KLFKYKPNHGNTILPLILQNRPIPKSKVAENFKVKDIEEYFNSPDT--ETDRGLLDTYDP

L_weil_LEP1GSC086_2122 KLFKYKPNHGNTILPLILQNRPIPKSKAAENFKVKDIEEYFNSPDTDSEADRGLLDAYDP

L_borg_LEP1GSC103_0021 KLFKYKPNHGNTILPLILQNRPIPKSQVTENFKVKDIEEYFNSPDT--EEDRGLLDTHNP

L_alex_LEP1GSC062_4247 KLLQYKKGQGNSLIAALTYNK-----------------------KNIPQNETFKIENQEL

L_inte_LIC10496 KLIQYSKGSGNSILSNLIKNQ-----------------------ETGSKPKK--------

L_kirs_LEP1GSC049_3438 KLIQYSKGSGNSILSNLIKNQ-----------------------ETGSKPKK--------

L_nogu_LEP1GSC059_0339 KLIQYSKGSGNSILSNLIKNQ-----------------------ETGSKPKK--------

:*:: .

L_kmet_LEP1GSC052_4103 ----

L_alst_LEP1GSC193_3818 LKKK

L_sant_LEP1GSC048_2963 IKKK

L_mayo_LEP1GSC190_0013 IKKK

L_weil_LEP1GSC086_2122 IKKK

L_borg_LEP1GSC103_0021 IKKK

L_alex_LEP1GSC062_4247 IIND

L_inte_LIC10496 ----

L_kirs_LEP1GSC049_3438 ----

L_nogu_LEP1GSC059_0339 ----
