# Supplementary material for: Discovery of Novel Leptospirosis Vaccine Candidates Using Reverse and Structural Vaccinology
Source: Front Immunol. 2017 Apr 27;8:463. doi: 10.3389/fimmu.2017.00463 (PMC5406399; doi:10.3389/fimmu.2017.00463)
Supplement: Supplementary file 8 [file Data_Sheet_1.ZIP › Alignment Bb-OMPs/Mult_alignment_LIC10714_path_spp_orthol_immun_epit_highlighted.docx]

L_nogu_LEP1GSC059_1295 MKHRLALPLYIFLFLLFTWETSAQPEETNKPILSESSSNGE------------ETEKEKW

L_inte_LIC10714 MKHRSALPLCIFLFLLFSWEVPAQSEETNKPILSETSSNGE------------ETEKEKW

L_kirs_LEP1GSC049_1462 MKHRSSLPLCIFLFLLFSWETPAQPEETNKPILSESSSNGE------------EIEKEKW

L_kmet_LEP1GSC052_2955 MKQKSILSLSILILFLFSWGVPAQPEENPSVDKDKQTPNGQGQQPVPTTTNGDDSEKEKW

L_alst_LEP1GSC193_3584 MNQKSILPFCIFLLFLFFWGVPAQSQENP-PEKDKQPSNGE--------------DADKE

L_sant_LEP1GSC048_3462 MEQKSILPFCIFLFFLLSWEVLSQPEQNP-------SSNGE--------------EPE--

L_borg_LEP1GSC103_2095 MEQKSILSFCIFLLFLLSWGVFAQSEETQ-----RPSLNGE--------------EPE--

L_mayo_LEP1GSC190_2495 MEQKSIPSFCIFLLFLLSEGVLAQSEETQ-----RPSLNGE--------------ESE--

L_alex_LEP1GSC062_0065 MEQKSISYLCIFLFFLLSWGVFAQSEENP---TQRPSSNGE--------------ESE--

L_weil_LEP1GSC086_1761 MEQKSIPSLCIFLFFLLSWGIFAQSEENP---TQRPSSNGE--------------ESE--

*::. : *::::*: :*.::. . **: : :

L_nogu_LEP1GSC059_1295 NQGTISVIGKRKTDLKRIPGAATVIEKEFLEQTKPVDSMEVLRRIPGASIRYQDTGLILN

L_inte_LIC10714 NQGTISVIGKRKTDLKRIPGSATVIEKEFLEQTKPVDSMEVLRRIPGASIRYQDTGLILN

L_kirs_LEP1GSC049_1462 NQGTISVIGKRKTDLKRIPGSATVIEKEFLEQTKPVDSMEVLRRIPGASIRYQDTGLILN

L_kmet_LEP1GSC052_2955 SKGTISVIGKRKTDLKRIPGSATVIEKEFLDQVKPVDSMEVLRRVPGASIRYQDTGLILN

L_alst_LEP1GSC193_3584 GKGVISVIGRRKIDLKRIPGSATIIEKEFLEQTKPVDSMEVLRRVPGASIRYQDTGLILN

L_sant_LEP1GSC048_3462 -NAAISVIGKRKTDLKRIPGSAIVIEKEFLEQTKPVDSMEVLRRVPGASIRYQDTGLILN

L_borg_LEP1GSC103_2095 -RAAISVIGKRKVDLKRIPGSAIVVEKEFLEQTKPVDSMEVLRRVPGASIRYQDTGLILN

L_mayo_LEP1GSC190_2495 -RAAISVIGKRKVDLKRIPGSAIVIEKEFLEQTKPVDSMEVLRRVPGASIRYQDTGLILN

L_alex_LEP1GSC062_0065 -RAAISVIGKRKVDLKRIPGSAIVIEKEFLEQTRPVDSMEVLRRVPGASIRYQDTGLILN

L_weil_LEP1GSC086_1761 -RAAISVIGKRKVDLKRIPGSAIVIEKEFLEQTKPVDSMEVLRRVPGASIRYQDTGLILN

...*****.** *******:* ::*****:*..**********:***************

L_nogu_LEP1GSC059_1295 LGFRGVNNDLGRKVLILEDGVFTSLNPYSAPEQYYTPNIDRMERIEVVKGSGAILFGPST

L_inte_LIC10714 LGFRGVNNDLGRKVLILEDGVFTSLNPYSAPEQYYTPNIDRMERIEVVKGSGAILFGPST

L_kirs_LEP1GSC049_1462 LGFRGVNNDLGRKVLILEDGVFTSLNPYSAPEQYYTPNIDRMERIEVVKGSGAILFGPST

L_kmet_LEP1GSC052_2955 IGFRGVNNDLGRKVLILEDGIFTSLNPYSAPEQYYTPNIDRMERIEVVKGSGAILFGPST

L_alst_LEP1GSC193_3584 IGFRGVNNDLGRKVLILEDGIFTSLNPYSAPEQYYTPNIDRMERIEVVKGSGAILFGPST

L_sant_LEP1GSC048_3462 LGFRGVNNDLGRKVLILEDGIFTSLNPYSAPEQYYTPNIDRMERIEVVKGSGAILFGPST

L_borg_LEP1GSC103_2095 LGFRGVNNDLGRKVLILEDGIFTSLNPYSAPEQYYTPNIDRMERIEVIKGSGAILFGPST

L_mayo_LEP1GSC190_2495 LGFRGVNNDLGRKVLILEDGIFTSLNPYSAPEQYYTPNIDRMERIEVIKGSGAILFGPST

L_alex_LEP1GSC062_0065 LGFRGVNNDLGRKVLILEDGIFTSLNPYSAPEQYYTPNIDRMERIEVIKGSGAILFGPST

L_weil_LEP1GSC086_1761 LGFRGVNNDLGRKVLILEDGIFTSLNPYSAPEQYYTPNIDRMERIEVIKGSGAILFGPST

:*******************:**************************:************

L_nogu_LEP1GSC059_1295 IGGVVNFITKRPPKNPVFSVSAQGGSYGFFSSQVSYGGTFGNTGIDISILRKQGDGFRDH

L_inte_LIC10714 IGGVVNFITKRPPKDPMLSVSVQGGSYGFFSSQVSYGGTFGNTGIDISILRKQGDGFREH

L_kirs_LEP1GSC049_1462 IGGVVNFITKRPPKDPILSISAQGGSYGFFSSQVSYGGTFGNTGIDISILRKQGDGFREH

L_kmet_LEP1GSC052_2955 IGGVINFITKRPPKEPVLSVSTQGGSYGFFASQISYGGTFGNTGIDISVLRKQGDGFRDH

L_alst_LEP1GSC193_3584 IGGVINFITKRPPKEPVLSVSAQGGSYGFFSSQISYGGTFGNTGIDISILRKQGNGFRDH

L_sant_LEP1GSC048_3462 IGGVVNFITKRPSKEPILSVSAQGGSYGFFASQISYGGTFGNTGIDISFLRKQGNGFRDH

L_borg_LEP1GSC103_2095 IGGVVNFITKRPSKEPTLSVSAQGGSYGFFSSQVSYGGTFGNTGIDISFLRKQGNGFRDH

L_mayo_LEP1GSC190_2495 IGGVVNFITKRPSKEPILSVSAQGGSYGFFSSQVSYGGTFGNTGIDISLLRKQGNGFRDH

L_alex_LEP1GSC062_0065 IGGVVNFITKRPSKEPILSVSAQGGSYGFFSSQVSYGGTFGNTGIDISFLRKQGNGFRDH

L_weil_LEP1GSC086_1761 IGGVVNFITKRPSKEPILSVSAQGGSYGFFSSQVSYGGTFGNTGIDISFLRKQGNGFRDH

****:*******.*:* :*:*.********:**:**************.*****:***:*

L_nogu_LEP1GSC059_1295 QSFRIHEFSFKSVTDLNEKHTLTSKFLATAQDANMTYLGLTTAQFWNHPSSNFAEQDNRK

L_inte_LIC10714 QSFRIHEFSFKSVTDLNDKHTLTSKFLATAQDANMTYLGLTTAQFWNHSSSNFAEQDNRK

L_kirs_LEP1GSC049_1462 QSFRIHEFSFKSVTDLNEKHTLTSKFLATAQDANMTYLGLTTAQFWNHSSSNFAEQDNRK

L_kmet_LEP1GSC052_2955 QDFRLHEFSFKSVTDLNDKHTLTSKFLGTVQNANMTYLGLTTAQFWNNSSSNFAEQDNRQ

L_alst_LEP1GSC193_3584 QDFRLHEFSFKSVTDLNEKHTLTSKFLATAQDANMTYLGLTTAQFWNNSSSNFAEQDNRK

L_sant_LEP1GSC048_3462 QNFRLHEFSFKSTTDLNEKHVLTSKFLATVQDANMTYLGLTTAQFWNHSSSNFAEQDNRK

L_borg_LEP1GSC103_2095 QDFRLHEFSFKSITDLNEKHILTSKFLATAQDANMTYLGLTTAQFWNHSSSNFAEQDNRK

L_mayo_LEP1GSC190_2495 QDFRLHEFSFKSITDLNEKHILTSKFLATAQDANMTYLGLTTAQFWNHSSSNFAEQDNRK

L_alex_LEP1GSC062_0065 QDFRLHEFSFKSITDLNEKHILTSKFLATAQDANMTYLGLTTAQFWNHSSSNFAEQDNRK

L_weil_LEP1GSC086_1761 QDFRLHEFSFKSITDLNEKHILTSKFLATAQDANMTYLGLTTAQFWNHSSSNFAEQDNRK

*.**:******* ****:** ******.*.*:***************:.**********:

L_nogu_LEP1GSC059_1295 LERYSGDIGHEWKLADHSKLVTKVYSAYTERNWARQNYLRNTGSNFSAIPSNVIKSYDTE

L_inte_LIC10714 LERYSGDIGHEWKLTDHSKLVTKVYSAYTERNWARQNYLRNTGSNFNTIPSNVIKSYDTE

L_kirs_LEP1GSC049_1462 LERYSGDIGHEWKLADHSKLVTKVYSAYTERNWARQNYLRNTGSNFSTIPSNVIKSYDTE

L_kmet_LEP1GSC052_2955 LQRYSGDLGHEWKLTDNSKLVTKVYAAYTERNWARQNYVRNTGSYYSNYPGNVIKAYDTE

L_alst_LEP1GSC193_3584 LQRYSGDIGHEWKLTDHSKLVTKVYAAYTERNWVRQNYVRNTGSNFGAIPGNVIKSYDTE

L_sant_LEP1GSC048_3462 LQRYSGDIGHEWKFADHSKLVTKVYAAYTERNWARQNYLRNTGPNFGAIPGNVIKTYDTE

L_borg_LEP1GSC103_2095 LQRYSGDIGHEWKFTDHSKLVTKVYAAYTERNWARQNYLRNTGSNFGAIPGNVIKTYDTE

L_mayo_LEP1GSC190_2495 LQRYSGDIGHEWKFTDHSKLVTKVYAAYTERNWARQNYLRNTGSNFGAIPGNVIKTYDTE

L_alex_LEP1GSC062_0065 LQRYSGDIGHEWKLADHSKLVTKVYAAYTERNWARQNYLRNTGSNFGAIPGNVIKTYDTE

L_weil_LEP1GSC086_1761 LQRYSGDIGHEWKLTDHSKLVTKVYAAYTERNWARQNYLRNTGSNFGAIPDNVIKTYDTE

*:*****:*****::*:********:*******.****:****. :. *.****:****

L_nogu_LEP1GSC059_1295 PFVNRPGDTVYMLDSVGHRNRSYRFVGVESRYQQDYQFLGIKNQLDTGLRYHYETADIKY

L_inte_LIC10714 PFVNRPGDTVYMLDSVGHRNRSYRFVGAESRYQQDYQFLGIKNQLDAGLRYHYETADIKY

L_kirs_LEP1GSC049_1462 PFVNRPGDTVYMLDSVGHRNRSYRFVGVESRYQQDYQFLGIKNQLDAGLRYHYETADIKY

L_kmet_LEP1GSC052_2955 PFVNRPGDTVYMLDSVGHRNRSYRFVGAESRYQLDYQFFGIKNQLDAGLRYHYETADIKY

L_alst_LEP1GSC193_3584 PFVNRPGDTVYMLDGVGHRNRSYRFVGAESRYQQDFQFFGIKNQLDAGLRYHYETADVKY

L_sant_LEP1GSC048_3462 PFVNRPGDTVYMLDSVGHRNRSYRFVGAESRYQRDYQFFGIKNQLDAGLRYHYETADIKY

L_borg_LEP1GSC103_2095 PFVNRPGDTVYMLDSVGHRNRSYRFVGAESRYQQDYQFFGIKNQLDAGLRYHYETADIKY

L_mayo_LEP1GSC190_2495 PFVNRPGDTVYMLDSVGHRNRSYRFVGAESRYQQDYQFFGIKNQLDAGLRYHYETADIKY

L_alex_LEP1GSC062_0065 PFVNRPGDTVYMLDSVGHRNRSYRFVGAESRYQQDYQFFGIKNQLDAGLRYHYETADIKY

L_weil_LEP1GSC086_1761 PFVNRPGDTVYMLDSVGHRNRSYRFVGAESRYQQDYQFFGIKNQLDAGLRYHYETADIKY

**************.************.***** *:**:*******:**********:**

L_nogu_LEP1GSC059_1295 LDGPPTPDYAMFGNGFNNPPTGVVSSEYSLAKSGNVRDHEVNNTKSIAGFAQNSFKFQNS

L_inte_LIC10714 LDGPPTPDYAIFGNGFNNPPTGVASSEYSLAKSGNVRDHEVNNTKSLAGFAQNSFQFQNR

L_kirs_LEP1GSC049_1462 LDGPPTPDYAIFGNGFNNSPTGIASSEYSLAKSGNVRDHEVNNTKSLAGFTQNSFKFQNG

L_kmet_LEP1GSC052_2955 LDGPSTPDYAVFGNGLGNPPTGTASSEYSLAKSGNLRDHEVNNTKSVAGFAQNSFKFYDK

L_alst_LEP1GSC193_3584 IDGPSTPDYAIYGNGFENPPTGAASSEYSLAKSGNIRDHEVNNTKSIAGFAQNSFKFYDC

L_sant_LEP1GSC048_3462 LDGPSTPDYAIFGNGLDKPPTGASSSEYSLAKSGNIRDHEVNQTKSVAGFAQNSFKFYDR

L_borg_LEP1GSC103_2095 LDGPPTPDYAIFGNGFDKPPTGTASSEYSLSKSGNIRDHEVNHTKSVAGFAQNSFKFYDR

L_mayo_LEP1GSC190_2495 LDGPPTPDYAIFGNGFDKPATGAASSEYSLSKSGNIRDHEVNQTKSIAGFVQNSFKFYDQ

L_alex_LEP1GSC062_0065 LDGPPTPDYAIFGNGFDKPPTGAASSEYSLSKSGNIRDHEVNHTKSIAGFAQNSLKFYDR

L_weil_LEP1GSC086_1761 LDGPPTPDYAIFGNGFDKPPTGAASSEYSLSKSGNIRDHEVNHTKSIAGFAQNSLKFYDR

:***.*****::***: :..** ******:****:******:***:***.***::* :

L_nogu_LEP1GSC059_1295 FSVIPGIRYEMFTQNRTILRQQTIDPVTNQPDPNSPSQEVDKGSKHTYHVVIPGLGLTYD

L_inte_LIC10714 FSVIPGIRYEMFTQNRTILRQQTIDPVTNQPDPNSPSQEVDKGSKHTYHVVIPGLGLTYD

L_kirs_LEP1GSC049_1462 FSVIPGIRYEMFTQNRTILRQQTIDPVTNQPDPNSPSQEVDKGSKHTYHVVIPGLGLTYD

L_kmet_LEP1GSC052_2955 FSVIPGVRYETFTQNRTILRQQAIDPATNQPDPNAPSQEVDKGSRHVYHVVIPGLGLTYD

L_alst_LEP1GSC193_3584 FSIIPGVRYETFTQNRTILRQQTVNPATNQPDPNSPSQEVDKGSRHTYHVVIPGLGLTYD

L_sant_LEP1GSC048_3462 LSVIPGVRYETFTQNRTILRQQSLDPVTNQPLANSPSQEVDKGSKHTYHVVIPGLGLTYD

L_borg_LEP1GSC103_2095 FSVIPGIRYESFVQNRTILRQQSLDPITSQPLPNSPSQEVDKGSKHTYHVVIPGLGLTYD

L_mayo_LEP1GSC190_2495 FSVIPGIRYESFAQNRTILRQQSLDPVTNQPLPNSPSQEVDKGSKHTYHVVIPGLGLTYD

L_alex_LEP1GSC062_0065 FSVIPGIRYESFSQNRTILRQQSLDPATNQPLPNSPSQEVDKGSKHTYHVVIPGLGLTYD

L_weil_LEP1GSC086_1761 FSVIPGIRYESFSQNRTILRQQSLDPATNQPLPNSPSQEVDKGSKHTYHVVIPGLGLTYD

:*:***:*** * *********:::* *.** .*:*********.*.*************

L_nogu_LEP1GSC059_1295 IRKNSNWFKDLTWFAGAHKGFSPPRYQDALNNSGIINRIDPEYSYNYETGFRGDITKYLN

L_inte_LIC10714 IRKNANWFKDLTWFAGAHKGFSPPRYQDALNNSGIVNRIDPEYSYNYETGFRGDITKYLN

L_kirs_LEP1GSC049_1462 IRKNANWFKDLTWFAGAHKGFSPPRYQDALNNSGIVNRIDPEYSYNYETGFRGDITKYLN

L_kmet_LEP1GSC052_2955 IR------KDLTWFAGAHKGFSPPRYQDALNNSGVINKIDPEYSYNYETGIRGDITNYLN

L_alst_LEP1GSC193_3584 IRKNAGWIKDVTWFAGAHKGFSPPRYQDALNNSGVVNRIDPEYSYNYETGFRGDITNYLN

L_sant_LEP1GSC048_3462 LH------KDVTWFAGAHKGFSPPRYQDALNNSGVVNRIDPEYSYNYETGFRGDITNYLN

L_borg_LEP1GSC103_2095 LN------KDVTWFAGAHKGFSPPRYQDALNNSGVVNRIDPEYSYNYETGFRGDITNYLS

L_mayo_LEP1GSC190_2495 LN------KDVTWFAGAHKGFSPPRYQDALNNSGVVNRIDPEYSYNYETGFRGDITNYLN

L_alex_LEP1GSC062_0065 LN------KDVTWFAGAHKGFSPPRYQDALNNSGIVNRIDPEYSYNYETGFRGDITNYLN

L_weil_LEP1GSC086_1761 LN------KDVTWFAGAHKGFSPPRYQDALNNSGVVNRIDPEYSYNYETGFRGDITNYLN

:. **:***********************::*.************:*****:**.

L_nogu_LEP1GSC059_1295 AQITYFHLNYINQIIITSSTSGNDSASSAKNGGRTYSKGVETNVTFDPAQLFDASFKLPI

L_inte_LIC10714 AQITYFNLNYINQIIITSSTSGNDSASSAKNGGRTYSRGVETNVTFDPAQLFDSSFKLPI

L_kirs_LEP1GSC049_1462 AQITYFNLNYINQIIITSSTSGNDSASSAKNGGRTYSRGVETNVTFDPAQLFDASFKLPI

L_kmet_LEP1GSC052_2955 AQVTYFDLNYINQIIITSSTSGNDSASSAKNGGRTYSRGLETNVTFDPAKLFDASFKLPI

L_alst_LEP1GSC193_3584 AQVTYFDLNYVNQIIITSSASGNDSASSAKNGGRTYSRGLESNVTFDPAKFFDVSFKLPI

L_sant_LEP1GSC048_3462 AQVSYFDLNYINQIIITSSTSGNDSASSAKNGGRTYSRGLETNVTFDPAKLFNASFKLPI

L_borg_LEP1GSC103_2095 AQVSYFDLNYINQIIITSSTSGNDSASSAKNGGRTYSRGLETNVTFDPAKLFGASFKLPI

L_mayo_LEP1GSC190_2495 AQVSYFDLNYINQIIITSSTSGNDSASSAKNGGRTYSRGLETNVTFDPAKLFEASFKLPI

L_alex_LEP1GSC062_0065 AQVSYFDLNYINQIIITSSTSGNDSASSAKNGGRTYSRGLETNVTFDPAKLFEASFKLPI

L_weil_LEP1GSC086_1761 AQVSYFDLNYINQIIITSSTSGNDSASSAKNGGRTYSRGLETNVTFDPAKLFEASFKLPI

**::** ***:********:*****************.*:*:*******::* ******

L_nogu_LEP1GSC059_1295 DLIYTRADARMNQYSIDTSKITADQSFLDFVVTQKDKNGNYVPYVSRDTATLAIGFIHPK

L_inte_LIC10714 DLIYTRADARMNQYSIDTSKITADQSLLDFVVTQKDKNGNYVPYVSKDTATLAIGFIHPK

L_kirs_LEP1GSC049_1462 DLIYTRADARMNQYSIDTSKITADQSLLDFVVTQKDKNGNYVPYVSRDTATLAIGFIHSK

L_kmet_LEP1GSC052_2955 DLIYTRADAKMNQYSIDTSKVTANQSLLDFIVTQKDKNGNYVPYVSRDTATLAIGFVHQK

L_alst_LEP1GSC193_3584 DLIYTRADAKMNQYSIDTSKVTANQSLLDFMVTQKDKNGNYVPYVSRDTATLALSFIHQK

L_sant_LEP1GSC048_3462 DLIYTRADAKMNQYSIDATKFAPNQSLLDFIVTQKDKNGNYVPYVSRDTATLALGFIHSK

L_borg_LEP1GSC103_2095 DLIYTRADARMNQHSIDAAKITPNQSLLDFIVTQKDKNGNYVPYVSRDTATLALGFIHQK

L_mayo_LEP1GSC190_2495 DLIYTRADARMNQYSIDVAKITPNQSLLDFTVTQKDKNGNYVPYVSRDTVTLALGFIHQK

L_alex_LEP1GSC062_0065 DLIYTRADARMNQYSIDASKITPNQSLLDFIVTQKDKNGNYVPYVSRDTVTLALGFIHQK

L_weil_LEP1GSC086_1761 DLIYTRADARMNQYSVDVAKITPNQSLLDFIVAQKDKNGNYVPYVSRDTVTLALGFIHQK

*********.***:*:*.:*.:.:**:*** *:*************.**.***:.*:* *

L_nogu_LEP1GSC059_1295 GFYVRAEYQYFSSQYHDDSNTRTVYWADSITDPLGKKVLQYLNITSNSSGETGVIPAYEL

L_inte_LIC10714 GFYARVEYQYFSSQYHDDSNTRTVYWADSITDPLGKKVLQYLNITSNSSGETGVIPAYEL

L_kirs_LEP1GSC049_1462 GFYARMEYQYFSSQYHDDSNTRTVYWADSITDPLGKKVLQYLNITSNSSGETGVIPAYEL

L_kmet_LEP1GSC052_2955 GFYARAEYQYFSAQYHDDANTRTVYWADSITDPLGKKVLQYMNITSDSSGETGVIPAYSL

L_alst_LEP1GSC193_3584 GFYARAEYQYFSAQYHDDTNTRTAYWADLVTDPLGKKVLQYLNITSNSSGETGVIPAYGL

L_sant_LEP1GSC048_3462 GFYSRVEYQYFSAQYHDDSNTRTVYWADLITDPLGKKVLQYLNITSNSSGETGVIPAYGL

L_borg_LEP1GSC103_2095 GFYSRVEYQYFSAQYHDDSNTRTVYWADLITDPLGKKVLQYLNITSNSSGETGVIPAYGL

L_mayo_LEP1GSC190_2495 GFYSRVEYQYFSAQYHDDSNTRTVYWADLITDPLGKKILQYLNITSNSSGETGVIPAYGL

L_alex_LEP1GSC062_0065 GFYSRVEYQYFSAQYHDDSNTRTVYWADLITDPLGKKVLQYLNITSNSSGETGVIPAYGL

L_weil_LEP1GSC086_1761 GFYSRVEYQYFSAQYHDDSNTRTVSWADLITDPLGKKVLQYLNITSNSSGETGVIPAYGL

*** * ******:*****:****. *** :*******:***:****:*********** *

L_nogu_LEP1GSC059_1295 INCSLGYKDPVKRWSLFITGKNLTDVRYISGRLPEGIQVGPFRQINVGASFEL

L_inte_LIC10714 INCSLGYKDPIKRWSLFITGKNLADVRYISGRLPEGIQVGPFRQINVGATFEL

L_kirs_LEP1GSC049_1462 INCSFGYKDPIKRWSLFITGKNLADVRYISGRLPEGIQVGPFRQINVGASFEL

L_kmet_LEP1GSC052_2955 INASLGYKHPVKRWSVFLTGKNLADVRYISGRLPEGIQVGPFRQINVGVTFEL

L_alst_LEP1GSC193_3584 VNVSMGYKDPVKRWTLFITGKNLADVRYISGRLPEGIQVGPFRQINVGATFEL

L_sant_LEP1GSC048_3462 INFSLGYKDPIKRWSLFVTGKNLADVRYISGRLPEGIQVGPFRQINVGVTLEL

L_borg_LEP1GSC103_2095 IHFSMGYKDPIQRWSLFVTGKNLADVRYISGRLPEGIQVGPFRQINVGVTFEL

L_mayo_LEP1GSC190_2495 INFSLGYKDPIKRWSLFVTGKNLADVRYISGRLPEGIQVGPFRQINVGVTFEL

L_alex_LEP1GSC062_0065 INFSLGYKDPIKRWNLFVTGKNLADVRYISGRLPEGIQVGPFRQINVGVTFEL

L_weil_LEP1GSC086_1761 INFSLGYKDPIQRWNLFVTGKNLADVRYISGRLPEGIQVGPFRQINVGVTFEL

:: *:*** *::**.:*:*****:************************.::**
