# Supplementary material for: Discovery of Novel Leptospirosis Vaccine Candidates Using Reverse and Structural Vaccinology
Source: Front Immunol. 2017 Apr 27;8:463. doi: 10.3389/fimmu.2017.00463 (PMC5406399; doi:10.3389/fimmu.2017.00463)
Supplement: Supplementary file 8 [file Data_Sheet_1.ZIP › Alignment Bb-OMPs/Mult_alignment_LIC10881+LIC10882_path_spp_orthol_immun_epit_highlighted.docx]

L_sant_LEP1GSC048_0398 ---------------MFVFVIRFFRANRNPLR----LLFSMLLFLTSVETLMAKVSLRLK

L_alex_LEP1GSC062_4164 ------------------------------------------------------------

L_weil_LEP1GSC086_1012 --------MYSVQTAMFANRSRFFLGNRNGFQKNLFLAFSALFSFVFAGTLWAKATLRLK

L_alst_LEP1GSC193_4079 MEPVRNKTKGYSVLATMSVRKNKRRYPQHRPQRKLLVLFSVFLSLISAENLFAKTSIRVK

L_borg_LEP1GSC103_1904 ------------------------MTYYMPIRKLIIPIFILLSISCWAQSIFSKTPIRLK

L_mayo_LEP1GSC190_0385 ------------------------------------------------------------

L_inte_LIC10881* -------------MFAFNAQPFFSSLNQKSLQRKFFLVFPVLLSLTSAGTLWAKASLRLK

L_kirs_LEP1GSC049_0746 -----------------MGKDKTGVSLNLINRNFIISIFILFYSTCWVQSLFSKTPVRLK

L_nogu_LEP1GSC059_3004 ------------------------------------------------------------

L_sant_LEP1GSC048_0398 VFA--KSDPPTEVWVRGKGYSKIFPFSDQS-ELTIDLEEQGVYDVVLTFQSGTMEQKFVN

L_alex_LEP1GSC062_4164 ------------------------------------------------------------

L_weil_LEP1GSC086_1012 VFANSKSDPPSEIWIRGNGYSKVFSFSDKS-ELTVDLKEQGVYDVIVTYQSGNMEQKSVT

L_alst_LEP1GSC193_4079 VIDRFKSDAPSEIWIRGRGYSRVFSFSEKS-ELVADLKDEGVYDIILSFKSGTMERKSVK

L_borg_LEP1GSC103_1904 ISTNSKSDIPVQLILRGKDFEKNISFFEFK-EITIELPDRGNYEVVLIWKSGFTEKRTVN

L_mayo_LEP1GSC190_0385 ----------MQLILRGKDFEKNISFFEFK-EITIELPDRGNYEVVLIWKSGFTEKRTVN

L_inte_LIC10881* IFANSKSDPPTQIWVRGNGYSKVFSLSDQSSELTIDLKEQGVYDVILTFKSGEMEHKFVT

L_kirs_LEP1GSC049_0746 ISANSKTDQPVQLLVRGKNFEKNITFSDVR-ETTIELPDQGNYETVLIWKSGFTEKRTVN

L_nogu_LEP1GSC059_3004 ------------------------------------------------------------

L_sant_LEP1GSC048_0398 VN-SETKYLEFIGKSKDAA-AINVLAKRPDSPPNYTISQEDAVRMPGGFGDALKAIQSMP

L_alex_LEP1GSC062_4164 ------------------------------------MNQEDAVKMPGGFGDALKAVQSMP

L_weil_LEP1GSC086_1012 VD-SDGKNLEFVQKQKVVN-GINVVGKKPDSPPNYVLSQEDAIRMPGGFGDALKAVQSMP

L_alst_LEP1GSC193_4079 VDLNEEEIIEFVVGQKNTA-AINVTAKKPEAPPNYVLSQEEAIRMPGGFGDALKAVQSMP

L_borg_LEP1GSC103_1904 AT-QENFSIEFVSNPNVSERAINVTAKKPEAPPNYTLNQEDAVRMPGGFGDALKAVQSMP

L_mayo_LEP1GSC190_0385 AT-QENFSIEFVSNPNVSERAINVTAKKPEAPPNYTLNQEDAVRMPGGFGDALKAVQSMP

L_inte_LIC10881* VD-SDEKNLEFVQKTKKVTNGINVVGKRPEAPPNYVLSQEDAIRMPGGFGDALKAVQSMP

L_kirs_LEP1GSC049_0746 VT-QNDFNIEFTSNPNSSADSINVTAKRPEAPPNYTLSQEDAVRMPGGFGDALKAVQSMP

L_nogu_LEP1GSC059_3004 --------------------------------------------MPGGFGDALKAVQSMP

***********:****

L_sant_LEP1GSC048_0398 GIIPLYQTYTGSSFQSALQTFNQTSTQNSKPDKPNGESGFLVMRGAGSRANQFYFNGLPM

L_alex_LEP1GSC062_4164 GIIPLYQTYIGSSFQSAIQTYNITAGSNKNPDKPNGEPGFLVMRGAGTRANQFYFNGFPM

L_weil_LEP1GSC086_1012 GISPMFQMYTGSSFQSAIQTFGQSTNQNK-PDKPNSEKGFLVMRGAGTRANQFYFNGFPV

L_alst_LEP1GSC193_4079 GVSPIYQMFNGASFQSAIQTFNQQTNQNK-PDKPNGESGFLVMRGAGARSNQFYFNGLPM

L_borg_LEP1GSC103_1904 GISPMYQTYTGASFQSAIQIFSQV-NPNK-PDKPNGEKGFLVMRGAGARANQFYFNGLPM

L_mayo_LEP1GSC190_0385 GISPMYQTYTGASFQSAIQIFSQV-NPNK-PDKPNGEKGFLVMRGAGARANQFYFNGLPM

L_inte_LIC10881* GISPMYQMYTGASFQSAIQTFAQATNPDK-PDKPNGEKGFLVMRGAGARANQFYFNGLPM

L_kirs_LEP1GSC049_0746 GISPMYQMYTGASFQSAIQTFSQVTNPDK-PDKPNGEKGFLVMRGAGSRANQFYFNGLPM

L_nogu_LEP1GSC059_3004 GISPMYQMYTGASFQSAIQTFSQVTNPDK-PDKPNGEKGFLVMRGAGARANQFYFNGLPM

*: *::* : *:*****:* : :. *****.* *********:*:*******:*:

L_sant_LEP1GSC048_0398 SYPFHADGLTSVVSNNAIRSMELYSGSYSARYGFATGGIVNIEGFRKRDSNLRVAHLNAL

L_alex_LEP1GSC062_4164 SYPFHADGLASVINNNAIRSLELYMGSYSARYGFATGGIVNIEGFQKRDSNLSVAHLNAF

L_weil_LEP1GSC086_1012 SYPFHADGLTSVINNNAIRSLELYMGSYSARYGFATGGIINIEGFQKRDSNLSVAHLNAF

L_alst_LEP1GSC193_4079 SYPFHADGLTSVINNNAIRSLELYSGSYSARYGFATGGIINIEGFQKRDSNLSVAHLNAF

L_borg_LEP1GSC103_1904 SYPFHADGLTSVINNNAIRSLELYSGSYSARYGFATGGIVNIEGFQKRDSNLSVAHLNAF

L_mayo_LEP1GSC190_0385 SYPFHADGLTSVINNNAIRSLELYSGSYSARYGFATGGIVNIEGFQKRDSNLSVAHLNAF

L_inte_LIC10881* SYPFHADGLTSVINNNAIRSLELYSGSYSARYGFATGGIINIEGFQKRDSNLSVAHLNAF

L_kirs_LEP1GSC049_0746 SYPFHADGLTSVINNNAIRSLELYSGSYSARYGFATGGIINIEGFQKRDSNLSVAHLNAF

L_nogu_LEP1GSC059_3004 SYPFHADGLTSVINNNAIRSLELYSGSYSARYGFATGGIINIEGFQKRDSNLSVAHLNAF

*********:**:.******:*** **************:*****.****** ******:

L_sant_LEP1GSC048_0398 LTDVYAYHNITKDLNVSVSGKKYYPNVVFGRVPHLIPTETFLADYNDYQARIGWDINEHH

L_alex_LEP1GSC062_4164 LTDVYAYRNIKKDLNVSVSGKKYYPNVIFGRVPNMIPTETFMSDYNDYQARIGWDVNEHH

L_weil_LEP1GSC086_1012 LTDIYAYRNITKDLNVSVSGKKYYPNVIFGRVPNLIPTETFMSDYNDYQARVGWDVNEYH

L_alst_LEP1GSC193_4079 LTDVYTYRNITKDLNVSVSGKKYYPNIIFGRVPNLIPAETFLADYNDYQARIGWDINENH

L_borg_LEP1GSC103_1904 LTDVYTYRNITKDLNVSVSGKKYYPNVIFGRVPNLIPAETFLADYNDYQARIGWDMNENH

L_mayo_LEP1GSC190_0385 LTDVYTYRNITKDLNVSVSGKKYYPNVIFGRVPNLIPAETFLADYNDYQARIGWDMNENH

L_inte_LIC10881* LTDVYTYRNITKDLNVSVSGKKYYPNIVFGRVPNLIPAETFLADYNDYQARIGWDISENH

L_kirs_LEP1GSC049_0746 LTDVYTYRNITKNFNVSVSGKKYYPNIVFGRVPNLIPTETFLSDYNDYQARIGWDINENH

L_nogu_LEP1GSC059_3004 LTDVYTYRNITKNFNVSVSGKKYYPNIVFGRVPNLIPTETFLSDYNDYQARIGWDINENH

***:*:*.**.*::************::*****::**:***::********:***:.* *

L_sant_LEP1GSC048_0398 SIAVQTFGAKDKRYPFKEFSQYTPKEAGKSGFQPPS--DSVRLDRIFRTDGFQHVWKPSD

L_alex_LEP1GSC062_4164 TVSVQTFGAKDKRYPFKELSQYNPKEMAQSFGNPPSATEGIRLDRIFRTDGIQHVWKPKD

L_weil_LEP1GSC086_1012 TISVQTFGAKDKRYPFKELSQYNPKEIAQSFGNPPSATEGIRLDRIFRTDGIQHVWKPKD

L_alst_LEP1GSC193_4079 SLAIQTFGAKDKRYPFKELSQYNPKEAAQSFGDPPSGADAARLDRIFRTDGIQHVWKPKS

L_borg_LEP1GSC103_1904 SISFQTFGAKDKRYPFKELGQYDPKEAARSFASPPSAADAARLDRIFRTDGIQHIWKPKS

L_mayo_LEP1GSC190_0385 SISFQTFGAKDKRYPFKELGQYDPKEAARSFASPPSAADAARLDRIFRTDGIQHIWKPKS

L_inte_LIC10881* SLSFQTFGAKDKRYPFKELSQYNPKETAQSFANPPSDADAARLDRIFRTDGIQHIWKPKS

L_kirs_LEP1GSC049_0746 SISFQTFGAKDKRYPFKELSQYNPKETAQSFANPPSAADAARLDRIFRTDGIQHIWKPKS

L_nogu_LEP1GSC059_3004 SVSFQTFGAKDKRYPFKELSQYNPKETAQSFANPPSAADAARLDRIFRTDGIQHIWKPKS

:::.**************:.** *** ..* .*** :. **********:**:***..

L_sant_LEP1GSC048_0398 KIMNTFNVSRNYFKEVTESGMDAFVLDFSKKANPLSLFERVYTVQNEYFNDLKQIENVSE

L_alex_LEP1GSC062_4164 SITNTFNVSRNYFNEVTETGMDTLVTDIKKIGYPPSLYQRVHTIRNEYFNDLKQVENVSE

L_weil_LEP1GSC086_1012 SITNTFNVSRNYFNEVTETGMDTLVTDIKKIGYPPSLYQRVHTIRNEYFNDLKQVENISE

L_alst_LEP1GSC193_4079 SIINTFNVSRNYFNETTENGLDMVVTDISKIGYPPSLYKRVQTIKNEYFNDLRQIENVSE

L_borg_LEP1GSC103_1904 SITNTFNVSRNYFNEVVESGLDLLVFDIAKIAYPPSLYKRVQTIQNEYFNDLRQIENVSE

L_mayo_LEP1GSC190_0385 SITNTFNVSRNYFNEVIESGLDLLVFDIAKIAYPPSLYKRVQTIQNEYFNDLRQIENVSE

L_inte_LIC10881* SITNTFNVSRNYFNEVTENGLDMLVLDITKIGYPPSLYKRVQTIQNEYFNDLRQIENVSE

L_kirs_LEP1GSC049_0746 SITNTFNVSRNYFNEVTENGLDMLVLDIAKIGYPPSLYKRVQTIQNEYFNDLRQIENVSE

L_nogu_LEP1GSC059_3004 SITNTFNVSRNYFNEVTENGLDMLVLDIAKVGYPPSLYKRVQTIQNEYFNDLRQIENVSE

.* **********:*. *.*:* .* *: * . * **::** *:.*******.*:**:**

L_sant_LEP1GSC048_0398 IELLKKKWKVVFGGQYREVDAGYKGRISQFNPDPFYEYLHKQLLSSPDAVAVLEGDSART

L_alex_LEP1GSC062_4164 IELLKRNWKIAFGGQYREVNAGYKGKVTQLNLDPALEFLQKQYLNSPEAREVLEGDSART

L_weil_LEP1GSC086_1012 IELLKRNWKIAFGGQYREVNAGYKGKVTQLSLDPALEFLQKQYLNSPEAREVLEGGSART

L_alst_LEP1GSC193_4079 IELLKRNWKIVFGGQYREVDAGYKGQVIQANLDPSYNFVHQQLLDSPNVKSVLEGDSTRT

L_borg_LEP1GSC103_1904 IELLKRNWKVVFGGQYREVDIGYKGKVSQMDLDPTYNFIHQQLFNSSDVKSVLEGDSVRT

L_mayo_LEP1GSC190_0385 IELLKRNWKVVFGGQYREVDIGYKGKVSQMDLDPTYNFIHQQLFNSSDVKSVLEGDSVRT

L_inte_LIC10881* VELLKRNWKIVFGGQYREVDTGYKGKVSQIDLDPTYNFIHQQLLNSSDVKSVLEGDSVRT

L_kirs_LEP1GSC049_0746 VELLKRNWKIVFGGQYREVDTGYKGKVSQINLDPTYNFIHQQLLNSSDVKSVLEGDSVRT

L_nogu_LEP1GSC059_3004 IELLKRNWKIVFGGQYREVDTGYKGKVSQIDLDPTYSFLHQQLLNSSDVKSVLEGDSVRT

:****.:**:.********: ****.: * . ** .::::* :.*.:. ****.*.**

L_sant_LEP1GSC048_0398 RQIGYFAENRFKFDDFNVNLGVRREYYDRAKEWKTAPRLGISKEIPYTQSRIFAGFGKHF

L_alex_LEP1GSC062_4164 RQIGYFFENRLKLKDFNLNLGVRREYYDKSGEWKTSPRVGLSKEIAYTQSRIFAGYGKHF

L_weil_LEP1GSC086_1012 RQIGYFFENRIKFKDFNLNLGVRREYYDKSGEWKTSPRVGLSKEIAYTQSRIFAGYGKHF

L_alst_LEP1GSC193_4079 RQIGAFFENRFKFNDFNLNLGVRREYYDKSREWKTAPRIGISKEIAYTQSRIFAGYGKHL

L_borg_LEP1GSC103_1904 RQIGTFFENRLRFYDFNLNLGIRREYYDKSKEWKTAPRIGISKEIAYTQSRIFAGYGRHF

L_mayo_LEP1GSC190_0385 RQIGTFFENRLRFYDFNLNLGVRREYYDKSKEWKTAPRIGISKEIAYTQSRIFAGYGRHF

L_inte_LIC10881* RQIGTFFENRFKFYDFNLNLGVRREYYDKSREWKTAPRIGISKEIAYTQSRIFAGYGKHF

L_kirs_LEP1GSC049_0746 RQIGTFFENRFKFYDFNLNLGVRREYYDKSREWKTAPRVAISKEIAYTQSRIFAGYGKHF

L_nogu_LEP1GSC059_3004 RQIGTFFENRFKFYDFNLNLGVRREYYDKSREWRTAPRVAISKEIAYTQSRIFAGYGRHF

**** * ***:.: ***:***:******.: **.*:**:.:****.*********:*.*:

L_sant_LEP1GSC048_0398 QAPVDVSRYSTRTGNPNLKMEESDHAEIGWDQKLENLWSVKIEGYRNTFSNLSVADPYAM

L_alex_LEP1GSC062_4164 QAPADVSRYSRRTGNPNLKMEESDHAEIGWDQKIGNLWNVKIEGYQNTFSNLSVADPFIM

L_weil_LEP1GSC086_1012 QAPADVSRYSRRTGNPSLKMEESDHAEIGWDQKIGNLWNVKIEGYQNTFSNLSVADPFIM

L_alst_LEP1GSC193_4079 QAPTDVSRYSARTGNPDLKMEESDHAEIGWDQRIDKFWNIKIEGYQNTFSNLAVADRFVM

L_borg_LEP1GSC103_1904 QAPADVSRYSVRTGNPKLKMEESDHAEIGWDQKIGNSWNIKIEGYQNTFSNLSVADPYAM

L_mayo_LEP1GSC190_0385 QAPADVSRYSVRTGNPKLKMEESDHAEIGWDQKIGNSWNIKIEGYQNTFSNLSVADPYAM

L_inte_LIC10881* QAPSDVSRYSARTGNPNLKMEESEHAEIGWDQKIGNFWNIKIEGYQNTFSNLSIADPYAM

L_kirs_LEP1GSC049_0746 QAPADVSRYSARTGNPNLKMEESDHAEIGWDQKIDNFWNIKIEGYQNTFSNLSIADPYAM

L_nogu_LEP1GSC059_3004 QAPADVSRYSARTGNPNLKMEESDHSEIGWDQKIGNLWNIKIEGYQNTFSNLSIADPYAM

*** ****** *****.******:*:******.: : *.:*****.******::** : *

L_sant_LEP1GSC048_0398 DPFSSHRDLARETLNPNSDGSLLRANRLNYSNSMTGYSQGIELFVKKEPVTESGFYGWLS

L_alex_LEP1GSC062_4164 DPYSVNRDLRREALDPSADPSLFRSGNLHYSNSMTGYSRGIEVFIKKEASAESGFYGWLS

L_weil_LEP1GSC086_1012 DPYSLNRDLRREALDPSADPSLFRSSNLHYSNSMTGYSRGIEVFIKKEASAESGFYGWLS

L_alst_LEP1GSC193_4079 DPFSRNRDLARESLDPSSDPSLLRSSHLNYSNSMTGYSRGVEVFIKKEPSAESGFYGWLS

L_borg_LEP1GSC103_1904 DPFSKNRDLVRESLDPSVDLSLIRRSNLNYSNSMTGYSRGVEVFVKKEPSTESGLYGWLS

L_mayo_LEP1GSC190_0385 DPFSKNRDLVRESLDPSADLSLIRRSNLNYSNSMTGYSRGVEVFVKKEPSTESGLYGWLS

L_inte_LIC10881* DPFSRNRDLMRESLDPNADLSLVRRSNLNYSNSMTGYSRGVEVFIKKEASAESGLYGWIS

L_kirs_LEP1GSC049_0746 DPFSRNRDLMRESLDPNADLSLVRRSNLNYSNSMTGYSRGVEVFIKKEPSAESGLYGWIS

L_nogu_LEP1GSC059_3004 DPFSRNRDLMRESLDPNADLSLIRRSNLNYSNSMTGYSRGVEVFIKKEPSTESGLYGWLS

**:* :*** **:*:*. * **.* ..*:*********.*:*:*:***. :***:***:*

L_sant_LEP1GSC048_0398 YTKSLTKRNRNLPTLTNQEYRSWLASSNSKDLVFQDDTKHYYANYYADGSYDILFKNSKE

L_alex_LEP1GSC062_4164 YTKSVTKRNRHMPELTNQEYNSWLAQSNSKDLVHQESTDQYYANFYRDGSYDVLFKNSKD

L_weil_LEP1GSC086_1012 YTKSVTKRNRHMPELTNQEYNSWLAQSNSKDLVHQESTDQYYANFYRDGSYDVLFKNSKD

L_alst_LEP1GSC193_4079 YTKSVTKRNRNLPELTNQEYNSWLAQSSTKELIHQENADRYYANFYRDGSYDVLFKNSKD

L_borg_LEP1GSC103_1904 YTKSITKRNRNLPELNRQEYSSWLAESSAKELIHQENADHYYANFYRDGSYDVLFKKSKE

L_mayo_LEP1GSC190_0385 YTKSITKRNRNLPELNRQEYSSWLAESSAKELIHQENADHYYANFYRDGSYDVLFKKSKE

L_inte_LIC10881* YTKSITKRNRNLPELTKQEYSSWLAESSAKDLIHQENTDYYYANFYRDGSYDVLFKNSKE

L_kirs_LEP1GSC049_0746 YTKSITKRNRNLPELTKQEYSSWLSESSAKDLIHQENTDHYYANFYRDGSYDVLFKNSKE

L_nogu_LEP1GSC059_3004 YTKSITKRNRNLPELSRQEYSAWLSESSAKELLTIA------------------------

****:*****::* *..*** :**:.*.:*:*:

L_sant_LEP1GSC048_0398 ELYDFDRTHVFTMVLGWRFSDKGQIGLKTTYLTNYAYTPLSGSNQTTLQKSLAEVFTDVT

L_alex_LEP1GSC062_4164 ELYDLDRTHIFNIVLGWKFEEKAQIGLKGTYLTNYAYTPVSGSKSMTLNQSISDLFPDLP

L_weil_LEP1GSC086_1012 ELYDLDRTHIFNVVLGWKFGEKAQIGLKGTYLTNYAYTPVSGSKSMTLNQSISDLFPDLP

L_alst_LEP1GSC193_4079 ELYDFDRTHMFNMVLGWKFGEKAQIGLKVTYLTNYAYTPVTGSKTITLQQSLSELFPSL-

L_borg_LEP1GSC103_1904 ELYDFDRTHMFNMVIGWKFGEKGQIGLRATYLTNYAYTPVVGSKTSTTQQILSELFPYL-

L_mayo_LEP1GSC190_0385 ELYDFDRTHMFNMVIGWKFGEKGQIGLRATYLTNYAYTPVVGSKISTTQQILSELFPYL-

L_inte_LIC10881* ELYDFDRTHMFNMVIGWKFGEKAQIGLRGTYLTNYAYTPVVGSKSITSEQFNSQLLPSAA

L_kirs_LEP1GSC049_0746 ELYDFDRTHMFNMVIGWKFGEKAQIGLRGTYLTNYAYTPVVGSKTITSEQLSSQLFPSAA

L_nogu_LEP1GSC059_3004 ------------------------------------------------------------

L_sant_LEP1GSC048_0398 IPAT---NNGGSSSNPPLYEPVYSNMNRSARLPHYRQLDLRFDRFINTDWGKLTLYLEFV

L_alex_LEP1GSC062_4164 IPPSTAPSSGDSSTLLPFYQPVYSDMRRSARLPHYHQFDLRFDRFIPTSWGRMTAYFELV

L_weil_LEP1GSC086_1012 IPPSTAPSSGDSSTLLPFYQSVYSDMRRSARLPHYHQFDLRFDRFIPTSWGRMTAYFELV

L_alst_LEP1GSC193_4079 -PPLPA-SSSTSSSALPLYQPIYSDMNRSARLPHYRQFDLRFDRFIPTSWGRITAYLELV

L_borg_LEP1GSC103_1904 -PQFSS-SSNSTPSGFALYQPVYSDIPRSARLPHYQQFDLRFDRFIPTSWGRMTLYLELV

L_mayo_LEP1GSC190_0385 -PQFSS-SSNSSPSGFALYQPVYSDIPRSARLPHYQQFDLRFDRFIPTSWGRMTLYLELV

L_inte_LIC10881* PPPPSS-SSNSSSSLFSIYQPVYSDMLRSARLPHYHQFDLRFDRFIPTSWGRMTAYLELV

L_kirs_LEP1GSC049_0746 PPPPSS-SSSSSSSLFSIYQPVYSDMLRSARLPHYHQFDLRFDRFIPTSWGRITAYFELV

L_nogu_LEP1GSC059_3004 ------------------------------------------------------------

L_sant_LEP1GSC048_0398 NITGSRIASGQETFIPLFPYIPGANPKTQYMYINGQQALQADKNKIPYLNFGIELRF

L_alex_LEP1GSC062_4164 NITGSRIAVSADTFSPIFPFVPGANPETQYIYLNGMQSLKTDKNKIPYINFGIELRF

L_weil_LEP1GSC086_1012 NITGSRIAVSADTFNPIFPFVSGRNPETQYIYLNGMQSLKTDKNKIPYMNFGIEFRF

L_alst_LEP1GSC193_4079 NITGSRIATTTDTFNPLFPHIPGANPETQYIYLNGLQSLRTDKNKIPYLNFGIEFRF

L_borg_LEP1GSC103_1904 NITGSRIAVSADTFNPLFPVVPGANPETRYIYLNGLQSLRTEKNKIPYLNFGIELRF

L_mayo_LEP1GSC190_0385 NITGSRIAVSADTFNPLFPVVPGANPETRYIYLNGLQSLRTEKNKIPYLNFGIELRF

L_inte_LIC10881* NITGSRIAVSADTFVPIFPFVPGANPETQYIYLNGLQSLRTEKNKIPYLNFGIEFRF

L_kirs_LEP1GSC049_0746 NITGSRIAVSADTFVLIFPFVPGTNPETQYIYLNGLQSLRTEKNKIPYLNFGIEFRF

L_nogu_LEP1GSC059_3004 ---------------------------------------------------------
