# Supplementary material for: Discovery of Novel Leptospirosis Vaccine Candidates Using Reverse and Structural Vaccinology
Source: Front Immunol. 2017 Apr 27;8:463. doi: 10.3389/fimmu.2017.00463 (PMC5406399; doi:10.3389/fimmu.2017.00463)
Supplement: Supplementary file 8 [file Data_Sheet_1.ZIP › Alignment Bb-OMPs/Mult_alignment_LIC10896+LIC10895_path_spp_orthol_immun_epit_highlighted.docx]

L_kmet_LEP1GSC052_1959 MKIYKKSLIGIFLSLGIFSIPIFAQGTGKVQGQALDAQTAEPMFGVTAVIRSINKFGRTD

L_sant_LEP1GSC048_2781 ---MKHKTICLLFFFSMITSPIFGQTTGKLGGKIVDSESGDPVFGTVVIVRSIKAATRSD

L_mayo_LEP1GSC190_4085 ---MKHKTIYLLFFFSMITSPIFGQTTGKLGGKVVDSESGDPVFGTVVIVRSIKAATRSD

L_alex_LEP1GSC062_1843 ---MKHKTIYLLFFFSMITSPIFGQTTGKLGGKVVDSESGDPVFGTVVIVRSIKAATRSD

L_weil_LEP1GSC086_0726 ---MKHKTIYLLFFFSMITSPIFGQTTGKLGGKVVDSESGDPVFGTVVIVRSIKAATRSD

L_borg_LEP1GSC103_0491 ---MKHKTIYLLFFLSMITSPIFGQTTGKLGGKVVDSESGDPVFGTVVIVRSIKAATRSD

L_alst_LEP1GSC193_2923 ---MKNKTISLFFLFSMMASPVFGQTTGKLSGKIVDSESGDPVFGTVIIVRSIKAATRSD

L_nogu_LEP1GSC059_2520 ---MKHKILSLFFFFSLIASPVFGQTTGKLVGKIVDSESGDPVFGTVIIVRSIKAATRSD

L_inte_LIC10896* ---MKHKILSLFFFLSLITSPVLGQSTGKLVGKIVDSESGDPVFGTVIIVRSIKAATRSD

L_kirs_LEP1GSC049_1657 ---MKHKILSLFFFLSLMTSPVLGQSTGKLVGKIVDSESGDPVFGTVIIVRSIKAATRSD

*:. : ::: :.::: *::.* ***: *: :*:::.:*:**.. ::***: *:*

L_kmet_LEP1GSC052_1959 VDGNYEISGVPDGNYTVEFIMQGMETQKKSVTVTGGKLVRANVAMGVKKLEEVVVEDRAL

L_sant_LEP1GSC048_2781 FDGKYELN-LPPGEHTVEFQMIGFATQFKKVTISPGTPLSLNVVMGAKLLDTVEVQGRGL

L_mayo_LEP1GSC190_4085 FDGKYELN-LPPGEHTVEFQMIGFATQSKKVTISPGTRLSLNVVMGAKLLDTVEVQGRGL

L_alex_LEP1GSC062_1843 FDGKYELN-LPPGEHTVEFQMIGFATQFKKVTINPATRLSLNVVMGAKLLDTVEVQGRGL

L_weil_LEP1GSC086_0726 FDGKYELN-LPPGEHTVEFQMIGFATQFKKVTISPETRLSLNVVMGAKLLDTVEVQGRGL

L_borg_LEP1GSC103_0491 FDGKYELN-LPPGEHTVEFQMIGFATQFKKVTISPETRLSLNVVMGAKLLDTVEVQGRGL

L_alst_LEP1GSC193_2923 FDGKYELN-LPPGEHTVEFQMIGFATQFKKITIAPGLRLPLNVVMGAQVLDTVEVKGRGL

L_nogu_LEP1GSC059_2520 FDGKYELN-LPPGEHSVEFQMIGFATQFKKITISPGARISMNVVMGAQVLDTVEVKGRGL

L_inte_LIC10896* FDGKYELN-LPPGEHSVEFQMIGFATQFKKITISPGARVSMNVVMGAQVLDTVEVKGRGL

L_kirs_LEP1GSC049_1657 FDGKYELN-LPPGEHSVEFQMIGFATQFKKVTISPGARVSMNVVMGAQVLDTVEVKGRGL

.**:**:. :* *:::*** * *: ** *.:*: : **.**.: *: * *:.*.*

L_kmet_LEP1GSC052_1959 NDTEASLLKFQKKAAAVSDGISAQAIQKTPDSSAGDVIRRVTGITLVGGRFVFVRGLGER

L_sant_LEP1GSC048_2781 ENSDSALLALQRKSGVVSDGISEEAIKKSPDSSAGDVLRRVTGITLVGGKFIFVRGLGER

L_mayo_LEP1GSC190_4085 ENSDSALLALQRKSGIISDGISEESIKKSPDSSAGDVLRRVTGITLVGGKFIFVRGLGER

L_alex_LEP1GSC062_1843 ENSDSALLALQRKSGIISDGISEESIKKSPDSSAGDVLRRVTGITLVGGKFIFVRGLGER

L_weil_LEP1GSC086_0726 ENSDSALLALQRKSGIISDGISEESIKKSPDSSAGDVLRRVTGITLVGGKFIFVRGLGER

L_borg_LEP1GSC103_0491 ENSDSALLALQRKSGIISDGISEESIKKSPDSSAGDVLRRVTGITLVGGKFIFVRGLGER

L_alst_LEP1GSC193_2923 ENSESALLALQRKSVVVSDGISEESIKKSPDSSAGDVLRRVTGITLVGGRFIFVRGLGER

L_nogu_LEP1GSC059_2520 ENSESALLALQRKSGVVSDAISEEAIKKSPDSSAGDVLRRVTGITLVGGRFIFVRGLGER

L_inte_LIC10896* ENSESALLALQRKSGVVSDAISEEAIKKSPDSSAGDVLRRVTGITLVGGRFIFVRGLGER

L_kirs_LEP1GSC049_1657 ENSESALLALQRKSGVVSDAISEEAIKKSPDSSAGDVLRRVTGITLVGGRFIFVRGLGER

::::::** :*.*: :**.** ::*:*:********:***********.*:********

L_kmet_LEP1GSC052_1959 YSNTLLNNSPLPSTEPNKRIVPLDLFPASLIKNIVVSKTFIPEDQAEFSGGTVKIETKDY

L_sant_LEP1GSC048_2781 YSNTILNDSVIPSPEPDKRIVPLDLFPAGVIKNIRVSKTASAEDSAEFSGGIVKIETKEY

L_mayo_LEP1GSC190_4085 YSNTILNDSIIPSPEPDKRIVPLDLFPAGVIKNIRVSKTASAEDSAEFSGGIVKIETKEY

L_alex_LEP1GSC062_1843 YSNTILNDSIIPSPEPDKRIVPLDLFPAGVIKNIRVSKTASAEDSAEFSGGIVKIETKEY

L_weil_LEP1GSC086_0726 YSNTILNDSMIPSPEPDKRIVPLDLFPAGVIKNIRVSKTASAEDSAEFSGGIVKIETKEY

L_borg_LEP1GSC103_0491 YSNTILNDSMIPSPEPDKRIVPLDLFPAGVIKNIRVSKTASAEDSAEFSGGIVKIETKEY

L_alst_LEP1GSC193_2923 YSNTILNDSVIPSPEPDKRIVPLDLFPAGVIKNIRVIKTASAEDSAEFSGGIVKIETKEY

L_nogu_LEP1GSC059_2520 YSNTILNDSVIPSPEPDKRIVPLDLFPAGVIKNIRVIKTASAEDSAEFSGGIVKIETKEY

L_inte_LIC10896* YSNTILNDSVIPSPEPDKRIVPLDLFPAGVIKNIRVIKTASAEDSAEFSGGIVKIETKEY

L_kirs_LEP1GSC049_1657 YSNTILNDSVIPSPEPDKRIVPLDLFPAGVIKNIRVIKTASAEDSAEFSGGIVKIETKEY

****:**:* :**.**:***********.:**** * ** .**.****** ******:*

L_kmet_LEP1GSC052_1959 PDQFFVKVGLQTGYNNNTTFQNFKTYSGGG-QDWLGLSEGNRSKPSMVDVLPDA-PFKAV

L_sant_LEP1GSC048_2781 PDNLSFSVSLGVGKNTQVAGHKFKSFDTGDMRNNFGLVSKKQELPDMISGLPKVIPF--V

L_mayo_LEP1GSC190_4085 PDNLSLSVSLGVGKNTQVAGHKFKSFNTGDIQNNFGLVSKKQELPDMISGLPKAIPF--V

L_alex_LEP1GSC062_1843 PDNLSLSVSLGVGKNTQVAGHKFKSFNTGDMHNNFGLVSKKQELPDMISGLPKAIPF--V

L_weil_LEP1GSC086_0726 PDNLSLSVSLGVGKNTQVAGHKFKSFNTGDMHNNFGLVSKKQELPDMISGLPKAIPF--V

L_borg_LEP1GSC103_0491 PDNLSLSVSLGVGKNTQVAGHKFKSFNAGDMHNNFGLVSKKQELPDMISGLPKAIPF--V

L_alst_LEP1GSC193_2923 PDTLSLSVSLGVGKNTQVAGHKFKTFNSGSAYNDFGLVSQRQELPDMISGLPKVIPF--V

L_nogu_LEP1GSC059_2520 PDTLSLSVSLGVGKNTQVAGHKFKTFNTGDINNSFGLVSKKQELPDMISGLPKVIPF--V

L_inte_LIC10896* PDTLSLSVSLGVGKNTQVAGHKFKTFDTGEMNNNFGLVSKKQELPDMISGLPKAIPF--V

L_kirs_LEP1GSC049_1657 PDTLSLSVSLGVGKNTQVAGHKFKTFDTGEMNNNFGLVSKKQELPDMIAGLPKVIPF--V

** : ..*.* .* *.:.: ::**::. * : :** . ... *.*: **.. ** *

L_kmet_LEP1GSC052_1959 GAGQY-GYNQAAVTLGSLQFSNQWTPTQMNAPLNKGFNFSIGNKFDLGGDRKLGLIFAIT

L_sant_LEP1GSC048_2781 EGDRFGGIPTNLMKVGALSFNQEWSPKEEDSPFNKSFSISAGNSFKTEKWGRFGILAGTT

L_mayo_LEP1GSC190_4085 EGDRFGGIPTNLMKVGALSFNQEWSPKEEDSPFNKSFSISAGNSFKTEKWGRFGVLAGTT

L_alex_LEP1GSC062_1843 EGDRFGGIPGNLMKVGALSFNQEWSPKEEDSPFNKSFSISAGNSFKTEKWGRFGVLAGTT

L_weil_LEP1GSC086_0726 EGDRFGGIPGNLMKVGALSFNQEWSPKEEDSPFNKSFSISAGNSFKTEKWGRFGVLAGTT

L_borg_LEP1GSC103_0491 EGDRFGGIPTNLMKVGALSFNQEWSPKEEDSPFNKSFSISAGNSFKTEKWGRFGVLAGTT

L_alst_LEP1GSC193_2923 EGDRFGGIPTGLVKVGSLSFSQEWSPKEEDSPFNKSFSISAGNSFKTEKWGRFGILAGTT

L_nogu_LEP1GSC059_2520 EGDRFGGIPTNLMKIGALSFNQEWSPKEEDSPFNKSFSISAGNSFKTEKWGRFGILAGTT

L_inte_LIC10896* EGDRFGGIPTNLMKVGALSFNQEWSPKEEDSPFNKSFSISAGNSFKTEKWGRFGILAGTT

L_kirs_LEP1GSC049_1657 EGDRFGGIPTSLIKVGALSFNQEWSPKEEDSPFNKSFSISAGNSFKTEKWGRFGILAGTT

...: * :.:*:*.*.::*:*.: ::*:**.*.:* **.*. .:*:: . *

L_kmet_LEP1GSC052_1959 YNNDYQYRRETDVLNRAGAVVPGIPVGDKNTQLNRAYKYDQHIYNESVNWGTILNTTFQV

L_sant_LEP1GSC048_2781 YNRTYNYREEANARFQASNPIS-LYLKDSNM-LRPLNQNNLKIYGEEVLWGNNLNLAYEP

L_mayo_LEP1GSC190_4085 YNRSYNYREEANARFQASNPIS-LYLKDSNI-LRPLNQNNLKIYGEEVLWGNNLNLAYEP

L_alex_LEP1GSC062_1843 YNRSYNYREEANARFQASNPIS-LYLKDSNM-LRPLNQNNLKIYGEEVLWGNNLNLAYEP

L_weil_LEP1GSC086_0726 YNRSYSYREEANARFQASNPIS-LYLKDSNM-LRPLNQNNLKIYGEEVLWGNNLNLAYEP

L_borg_LEP1GSC103_0491 YNRSYNYREEANARFQASNPIS-LYLKDSNM-LRPLNQNNLKIYGEEVLWGNNLNLAYEP

L_alst_LEP1GSC193_2923 YNRSYNYREESNARFQASNPIS-IYLKDSNM-LRPLNQNNLKIYGEEVLWGNNLNLAYEP

L_nogu_LEP1GSC059_2520 YNRSYNYREEANARFQASNPIS-IYLKDSNI-LRPLNQNNLKIYGEEVLWGNNLNLAYEP

L_inte_LIC10896* YNRSYNYREEANARFQASNPIS-IYLKDSNM-LRPLNQNNLKIYGEEVLWGNNLNLAYEP

L_kirs_LEP1GSC049_1657 YNRSYNYREEANARFQASNPIS-IYLKDSNM-LRPLNQNNLKIYGEEVLWGNNLNLAYEP

**. *.** *::. .*. :. : : *.* *. : : :**.*.* **. ** :::

L_kmet_LEP1GSC052_1959 ANGHRLHFKNFFSVNNDKEVMVYSGQNVASGQTIASDKLNYIMRNLYSSQLSGDHIINV-

L_sant_LEP1GSC048_2781 KIGQQFFIKTLYSVQSDKTVREGEGANYIDNFNFKSTNLSYISRTIFNNTLGGEHEIKA-

L_mayo_LEP1GSC190_4085 KIGQQLFIKTLYSVQSDKTVREGEGANYIDNFNFKSTNLSYISRTIFNNTLGGEHEINA-

L_alex_LEP1GSC062_1843 KIGQQFFIKTLYSVQSDKTVREGDGANYIDNFNFKSTNLSYISRTIFNNTLGGEHEINA-

L_weil_LEP1GSC086_0726 KVGQQFFIKTLYSVQSDKTVREGDGANYIDNFNFKSTNLSYISRTIFNNTLGGEHEINA-

L_borg_LEP1GSC103_0491 KIGQQFFIKTLYSVQSDKTVREGEGANYIDNFNFKSTNLSYISRTIFNNTLGGEHEINA-

L_alst_LEP1GSC193_2923 KVGQQFFIKTLYSVQSDKTVREGEGANYIDNFNFKSANLNYISRTVFNNTLGGEHEIKIK

L_nogu_LEP1GSC059_2520 KIGQQFFIKTLYSVQSDKTVREGDGANYIDNFNFKSTNLSYISRTIFNNTLGGEHEVRA-

L_inte_LIC10896* KIGQQFFIKTLYSVQSDKIVREGDGANYIDNFNFKSTNLNFISRTIFNNTLGGEHEVRA-

L_kirs_LEP1GSC049_1657 KIGQQFFIKTLYSVQSDKIVREGDGSNYIDNFNFKSTNLNFISRTIFNNTLGGEHEVRA-

*:.:.:*.::**:.** * .* * .. .: * :*.:* *.::.. *.*:* :.

L_kmet_LEP1GSC052_1959 -ANVKT-KLEWRFAQSEANRNQPDMRDTIYAVQSGLEGQMPAVLQGGTLGSSQFYSKTKD

L_sant_LEP1GSC048_2781 -FGSRSHKVEWNVNYALAERDEPNARNQAWS-QGGTDLANGFRRLGNNPDGTRYFSNTED

L_mayo_LEP1GSC190_4085 -FGSRSHKVEWNINYALAERDEPDARNQAWS-QGGTDLANGFRRLGNNPDGTRYFSNTED

L_alex_LEP1GSC062_1843 -FGSRSHKVEWNVNYALAERDEPNARNQAWS-QGGTDLANGFRRLGNNPDGTRYFSNTED

L_weil_LEP1GSC086_0726 -FGSRSHKVEWNVNYALAERDEPNARNQAWS-QGGTDLANGFRRLGNNPDGTRYFSNTED

L_borg_LEP1GSC103_0491 -FSSRPHKVEWNINYALAERDEPNARNQAWS-QGGTDLANGFRRLGNNPDGTRYFSNTED

L_alst_LEP1GSC193_2923 ELSARSHKLEWNVNYALAERDEPDARNQAWS-QGGTDLANGYRRLGNNPDGTRYFSNTAD

L_nogu_LEP1GSC059_2520 -FSARPHKVEWNVNYALAERDEPDARNQAWS-QGGTDLANGYRRLGNNPDGTRYFSNTAD

L_inte_LIC10896* -FSARPHKVEWNVNYALAERDEPDARNQAWS-QGGTDLANGYRRLGNNPDGTRYFSSTAD

L_kirs_LEP1GSC049_1657 -FSARPHKVEWNVNYALAERDEPDARNQAWS-QGGTDLANGYRRLGNNPDGTRYFSNTAD

. .. *:**.. : *:*::*: *: :: *.* : *.. ..:.::*.* *

L_kmet_LEP1GSC052_1959 LSRHAGLDYEIPFNQWDGLQSKLKIGYSAVQRERGFEAQRYFFRGQSSGSMTNLAGGAIP

L_sant_LEP1GSC048_2781 TVRSQSLKYEIPFNQWDGLQSKLKFGISNLDRFKHFE-----FREIAQRNFNGSDKDYI-

L_mayo_LEP1GSC190_4085 TVRSQSLKYEIPFNQWDGLQSKLKFGISNLDRFKHFE-----FREIAQRNFNGSDKDYI-

L_alex_LEP1GSC062_1843 TVRSQSLKYEIPFNQWDGLQSKLKFGISNLDRFKHFE-----FREIAQRNFNGSDKDYI-

L_weil_LEP1GSC086_0726 TVRSQSLKYEIPFNQWDGLQSKLKFGISNLDRFKHFE-----FREIAQRNFNGSDKDYI-

L_borg_LEP1GSC103_0491 TVRSQSLKYEIPFNQWDGLQSKLKFGISNLDRFKHFE-----FREIAQRNFNGSDKDYI-

L_alst_LEP1GSC193_2923 TVRSQSLKYEIPFSQWDGLQSKLKFGVSNLDRFKHFE-----FREIAQRNFSGSDRDSI-

L_nogu_LEP1GSC059_2520 TVRSQSLKYEIPFNQWDGLQSKLKFGISNLDRFKHFE-----FREIAQRNFTGSDRDVA-

L_inte_LIC10896* TVRSQSLKYEIPFNQWDGLQSKLKFGISNLDRFKHFE-----FREIAQRNFTGSDRDVI-

L_kirs_LEP1GSC049_1657 TVRSQSLKYEIPFNQWDGLQSKLKFGISNLDRFKHFE-----FREIAQRNFTGSDRDVI-

* .*.*****.**********:* * ::* . ** ** :. .:.. .

L_kmet_LEP1GSC052_1959 SPNYPIPPEVVYNPLNRGPKGYFVDEATQPTDKYNAKQQLFAKYLQVDMPITPKLRFIGG

L_sant_LEP1GSC048_2781 ---YPIPGEFVYNPLNYVNGNRKLYERASGNNAYDASQALRAAFTQLEVPILAKLKTILG

L_mayo_LEP1GSC190_4085 ---YPIPGEIIYNPLNYVNGNRKVYERASGNNAYDASQALRAAFTQLEVPILAKLKTIFG

L_alex_LEP1GSC062_1843 ---YPIPGEIIYNPLNYANGNRKVYERASGNNAYDASQALRAAFTQLEVPIFAKLKTIFG

L_weil_LEP1GSC086_0726 ---YPIPGEIVYNPLNYVNGNRKVYERASGNNAYDASQALRAAFTQLEVPVLAKLKTIFG

L_borg_LEP1GSC103_0491 ---YPIPGEIIYNPLNYVNGNRKVYERASGNNAYDASQALRAAFTQLEVPILAKLKTIFG

L_alst_LEP1GSC193_2923 ---YPIPGEVVYNPLTYANGNRKMYERASGNNAYDASQALRAAFTQLEVPILAKLKTIVG

L_nogu_LEP1GSC059_2520 ---YPIPGEVIYNPLAYANGNRKIYERASGNNAYDASQALRAAFAQLEIPILAKLKSIVG

L_inte_LIC10896* ---YPIPGEVIYNPLAYANGNRKIYERASGNNAYDASQALRAAFAQLEVPILAKLKSIVG

L_kirs_LEP1GSC049_1657 ---YPIPGEVIYNPLAYANGNRKIYERASGNNAYDASQALRAAFAQLEVPILAKLKSIVG

**** *.:**** . : * :. .: *:*.* * * : *:::*: .**. * *

L_kmet_LEP1GSC052_1959 ARHEDNYQSVATQNPFDPNAAFFDRYNFKSYLNGYELSIVDPTFRSPAAINA----NKNL

L_sant_LEP1GSC048_2781 VRYEDSFQKTQTYDLKNSWSGFNTSYGCKTNSEEERLLLVRSNICDANNVGIGELRTKDK

L_mayo_LEP1GSC190_4085 VRYEDSYQKTQTYDLKNSWNGFNTSYGCKTNSEEERLLLVRSNICDANNVGIGELRTQDK

L_alex_LEP1GSC062_1843 IRYEDSYQKTQTYDLKNSWSGFNTSYGCKTNSEEERLLLVRSNICDANNVGIGELRTKDK

L_weil_LEP1GSC086_0726 VRYEDSYQKTQTYDLKNSWSGFNTSYGCKTNSEEERLLLVRSNICDANNVGIGELRTKDK

L_borg_LEP1GSC103_0491 VRYEDSYQKTQTYDLKNSWNGFNTSYGCKTNSEEERLLLVRSNICDTNNVGIGELRTKDK

L_alst_LEP1GSC193_2923 VRYEDSYQKTQTYDLKNSWNGFNTSYGCKTNSEEERLLLVRSNVCDVNNVGIGELRTKDK

L_nogu_LEP1GSC059_2520 VRYEDSYQKTKTYDLKNSWNGFNTSYGCKTNSEEERLLLIRANICDSTNVGIGELRTKDK

L_inte_LIC10896* VRYEDSYQKTKTYDLKNSWNGFNTSYGCKTNSEEERLLLVRANICDATNVGIGELRTKDK

L_kirs_LEP1GSC049_1657 VRYEDSYQKTKTYDLKNSWNGFNTSYGCKTNSEEERLLLVRANICDATNVGIGELRTKDK

*:**.:*.. * : :.. .* *. *: : * :: ... . :. .::

L_kmet_LEP1GSC052_1959 LPSSNFVYAWDDKTNLRVSYTETISRPDFREMAPFQFFNVLGGGIEKGNQYLTRTYIHNY

L_sant_LEP1GSC048_2781 LPSANVVWEFAKDMNLRFGYSQTLTRPDLRELSPFGFAAFFQADRIFGNASLQRTYIHNY

L_mayo_LEP1GSC190_4085 LPSANIVWELVKDMNLRFGYSQTLTRPDLRELSPFGFAAYFQADRIFGNASLQRTYIHNY

L_alex_LEP1GSC062_1843 LPSANVVWELVKDMNLRFGYSQTLTRPDLRELSPFGFAAYFQADRIFGNASLQRTYIHNY

L_weil_LEP1GSC086_0726 LPSANVVWELVKDMNLRFGYSQTLTRPDLRELSPFGFAAYFQADRIFGNASLQRTYIHNY

L_borg_LEP1GSC103_0491 LPSANVVWELVKDMNLRFGYSQTLTRPDLRELSPFGFAAYFQADRIFGNASLQRTYIHNY

L_alst_LEP1GSC193_2923 LPSSNVVWEFAKDMNLRFAYSQTLTRPDLRELSPFGFAAYFQADRTFGNASLQRTYIHNY

L_nogu_LEP1GSC059_2520 LPSGNVVWEFAKDMNLRLGYSQTLTRPDLRELSPFGFAAYFQADRIFGNASLQRTYIHNY

L_inte_LIC10896* LPSANVVWEFAKDMNLRLGYSQTLTRPDLRELSPFGFAAYFQADRIFGNASLQRTYIHNY

L_kirs_LEP1GSC049_1657 LPSANVVWEFAKDMNLRLGYSQTLTRPDLRELSPFGFAAYFQADRIFGNASLQRTYIHNY

***.*.*: .. ***..*::*::***:**::** * : .. ** * *******

L_kmet_LEP1GSC052_1959 DFRYEKFPSADEIIAIGVFGKQMASPIEKV---MEVDSQFRYTYTNAKSAYVHGIELEVR

L_sant_LEP1GSC048_2781 DVRWEYYLTNTDYIGVGAFFKNLSNPIELIGLPVAGSASLVYKYANAQQATIRGIELDYR

L_mayo_LEP1GSC190_4085 DARWEYYLTNTDYIGVGAFFKNLSNPIELIGLPVAGSANLVYKYANAQQATIRGIELDYR

L_alex_LEP1GSC062_1843 DARWEYYLTNTDYIGVGAFFKNLSNPIELIGLPVAGSANLVYKYANAQQATIRGIELDYR

L_weil_LEP1GSC086_0726 DARWEYYLTNTDYIGVGAFFKNLSNPIELIGLPVAGSANLVYKYANAQQATIRGIELDYR

L_borg_LEP1GSC103_0491 DARWEYYLTNTDYIGVGAFFKNLSNPIELIGLPVAGSANLVYKYANAQQATIRGIELDYR

L_alst_LEP1GSC193_2923 DVRWEYYITNTDYIGAGAFFKNLSNPIELIGLPVAGSASLVYKYANAQQATIRGIELDYR

L_nogu_LEP1GSC059_2520 DLRWEYYITNTDYIGVGAFFKNLSNPIELIGLPVAGSASLVYKYANAQQATIRGIELDYR

L_inte_LIC10896* DLRWEYYITNTDYIGVGAFFKNLSNPIELIGLPVAGSASLVYKYANAQQATIRGIELDYR

L_kirs_LEP1GSC049_1657 DLRWEYYITNTDYIGVGAFFKNLSNPIELIGLPVAGSASLVYKYANAQQATIRGIELDYR

* *:* : : : *. *.* *:::.*** : : .:.: *.*:**:.* :.****: *

L_kmet_LEP1GSC052_1959 KSLNALSPKLERWAFGI--NTFFIKSEVQFQDWLYYQLSGTTQ----------RPTNLSR

L_sant_LEP1GSC048_2781 KEL--------LWWLRVEANIFFIKSRVDVIDANIYGFIATGQVDPISTYAAYAPTTLNR

L_mayo_LEP1GSC190_4085 REL--------LWWLKVEANVFFIKSRVDVIDANIYGFIATGQVDPISTYSAYAPTTLNR

L_alex_LEP1GSC062_1843 REL--------LWWLKVEANVFFIKSRVDVIDANIYGFIATGQVDPISTYSAYAPTTLNR

L_weil_LEP1GSC086_0726 REL--------LWWLKVEANVFFIKSRVDVIDANIYGFIATGQVDPISTYSAYAPTTLNR

L_borg_LEP1GSC103_0491 REL--------LWWLKVEANVFFIKSRVDVIDANIYGFIATGQVDPISTYSAYAPTTLNR

L_alst_LEP1GSC193_2923 KEL--------LWWLRVETNVFFIKSRVDVIDSNIYGFIATGQVDPISTYAAYAPTTLNR

L_nogu_LEP1GSC059_2520 KEL--------LWWLRVEANVFFIKSRVDVIDSKIYGLITTGQVDPLSTYAAYSPTTLNR

L_inte_LIC10896* KEL--------LWWLRVEANVFFIKSRVDVIDSKIYGLISTGQVDPLSTYAAYSPTTLNR

L_kirs_LEP1GSC049_1657 KEL--------LWWLRVEANVFFIKSRVDVIDSKIYGLITTGQVDPLSTYAAYSPTTLNR

..* * : : * ***** *:. * * : * * **.*.*

L_kmet_LEP1GSC052_1959 PLQGQSPYVYNVNLRYRFDDKGDHTITMLYNEFGPRINAVGGIGIPDTYERPVGMLDFVY

L_sant_LEP1GSC048_2781 PLQGQSDFVANFKVDLFVSKSKKHNIGLYYNYFSDRIALVGSDGVPNAIQKGTGTSDVVY

L_mayo_LEP1GSC190_4085 PLQGQSDFVANFKVDLFVSKSKKHNIGLYYNYFSDRIALVGSDGVPNAIQKGTGTSDVVY

L_alex_LEP1GSC062_1843 PLQGQSDFVANFKVDLFVSKSKKHNIGLYYNYFSDRIALVGSDGVPNAIQKGTGTSDVVY

L_weil_LEP1GSC086_0726 SLQGQSDFVANFKVDLFVSKSKKHNIGLYYNYFSDRIALVGSDGVPNAIQKGTGTSDVVY

L_borg_LEP1GSC103_0491 PLQGQSDFVANFKVDLFVSKSKKHNIGLYYNYFSDRIALVGSDGVPNAIQKGTGTSDVVY

L_alst_LEP1GSC193_2923 PLQGQSDFVANLKVDVFVSKSKKHNIGFYYNYFSDRIALVGSDGVPNAIQKGTGTSDVVY

L_nogu_LEP1GSC059_2520 PLQGQSDFVANLKVDVFVSKSKKHNVGFYYNYFSDRIALVGSDGVPNAIQKGTGTSDVVY

L_inte_LIC10896* PLQGQSDFVANLKIDVFVSKSKKHNIGFYYNYFSDRIALVGSDGVPNAIQKGTGTSDVVY

L_kirs_LEP1GSC049_1657 PLQGQSDFVANLKVDMFVSKSKKHNVGFYYNYFSDRIALVGSDGVPNAIQKGTGTSDVVY

.***** :* *.:: .... .*.: : ** *. ** **. *:*:: :. .* *.**

L_kmet_LEP1GSC052_1959 NLKFLEKWDIKIAARNVTDSRIKIVQENPILDSTVHSNTGFNIGNTFIPVGGHSYKGETI

L_sant_LEP1GSC048_2781 TYRHNDRLDFRSSARNVMNSQFKITQTDPLT--------------------GQEY---VF

L_mayo_LEP1GSC190_4085 IYRHNDRFDLRSSVRNIMNSQFKITQTDPLT--------------------GQEY---VF

L_alex_LEP1GSC062_1843 IYRHNDRFDFRSSVRNIMNSQFKITQTDPLT--------------------GQEY---VF

L_weil_LEP1GSC086_0726 IYRHNDRFDFRSSVRNVMNSQFKITQTDPLT--------------------GQEY---VF

L_borg_LEP1GSC103_0491 IYRHNDRFDFRSSVRNIMNSQFKITQTDPLT--------------------GQEY---VF

L_alst_LEP1GSC193_2923 TYKHNDRLDFRSSVRNVMNSQFKITQTDPLT--------------------GQEY---VF

L_nogu_LEP1GSC059_2520 TYRHNDRLDFRSSVRNIMNTQFKITQTDPLT--------------------GQEY---VF

L_inte_LIC10896* TYRHNDRLDFRSSVRNVMNTQFKITQTDPLT--------------------GQEY---VF

L_kirs_LEP1GSC049_1657 TYRHNDRLDFRSSVRNVMNTQFKITQTDPLT--------------------GQEY---VF

.. :. *:. :.**: ::.:**.* :*: *:.* .:

L_kmet_LEP1GSC052_1959 NSYRLGPTITFSVTYNLN

L_sant_LEP1GSC048_2781 QKYRTGLDISFSATYKL-

L_mayo_LEP1GSC190_4085 QKYRTGLDISFSATYKL-

L_alex_LEP1GSC062_1843 QKYRTGLDISFSATYKL-

L_weil_LEP1GSC086_0726 QKYRTGLDISFSATYKL-

L_borg_LEP1GSC103_0491 QKYRTGLDISFSATYKL-

L_alst_LEP1GSC193_2923 QKYRTGLDVSFSATYKL-

L_nogu_LEP1GSC059_2520 QKYRTGLDVSFSATYKL-

L_inte_LIC10896* QKYRTGLDVSFSATYKL-

L_kirs_LEP1GSC049_1657 QKYRTGLDVSFSATYKL-

:.** * ::**.**:*
