# Supplementary material for: Discovery of Novel Leptospirosis Vaccine Candidates Using Reverse and Structural Vaccinology
Source: Front Immunol. 2017 Apr 27;8:463. doi: 10.3389/fimmu.2017.00463 (PMC5406399; doi:10.3389/fimmu.2017.00463)
Supplement: Supplementary file 8 [file Data_Sheet_1.ZIP › Alignment Bb-OMPs/Mult_alignment_LIC10964_path_spp_orthol_immun_epit_highlighted.docx]

L_nogu_LEP1GSC059_2587 MKNHLYYYFRIFLFSILLFPVSIFALDVTLTGIVKDKYGNPISNVRIIVQESRKIAITND

L_inte_LIC10964 MKKQLYYYFRIFLFSILISPISILALDVTLTGIVKDKNGNSISNVKIILQESRKTTITNE

L_kirs_LEP1GSC049_4224 MKKQTYYYFRILLFSTFIFPISILALDVTLTGIAKDRRGNPISNVRITIQESRKIATTND

L_alst_LEP1GSC193_3780 MKKQLHSYFRILLFSIFISPASVFALDVVLTGSVKGKNGNPISNARVFIQESRKSAITDN

L_kmet_LEP1GSC052_2231 MKRQSFLYIRIFLFSIFISPFSLFALDVALTGVVKDKDGNPISNARILIQESRKGAITDD

L_sant_LEP1GSC048_0454 MKKQTHSSFGIFIVSIFISPISVFALDVALTGVVKNKDGKPISNARVLIQESRKSAVTDD

L_borg_LEP1GSC103_3191 MKEQLHLYFRIFIVSIFFSPVSVFALDVALIGVIKNKNGKPISNARVLIQESRKSAVTNE

L_mayo_LEP1GSC190_2194 MKEQVHLYFRIFIFSIFISPVSIFALDVVLTGVIKNKNGKPISNAKVLIQESRRSAVTDE

L_alex_LEP1GSC062_1651 MKEQIHLYFRIFIFSIFISPVPVFALDVALTGVIKNKNGKPISNAKVLIQESRKSAVTDE

L_weil_LEP1GSC086_3478 MKEQVYLHFIIFIFLIFISPISVFALDVALTGVVKDKNGKPISSARVLIQESRKNAVTDE

** : . : *::. :: * .::****.* * *.. *:.**...: :****. : *::

L_nogu_LEP1GSC059_2587 KGEFVLNHVPPGKYTLVAISRDHQSETISITVGEKDEKIQFILLESSLDQSAINVTAKST

L_inte_LIC10964 KGEFVLNHVPPGKYTLVAISRGYQSETISITIGEKDEKTQFTLLESSLDQSAINVTAKST

L_kirs_LEP1GSC049_4224 KGEFILSHVPPGKYTLVATSRDHQSETISITISEKDEKIQFILLESSLDQSAINVTAKST

L_alst_LEP1GSC193_3780 KGEFVLDHVPPGKYTLSATARGHQSETLSVTIGEKDQRIQFTLLEISLDESAINVTAKST

L_kmet_LEP1GSC052_2231 KGEFVLDHVPPGKYTVITIARGYQSETIGVQINDKDQKIQFVLSKSSLDESAINVTAKST

L_sant_LEP1GSC048_0454 KGEFVLDHVPPGKYVLTASARGYRSETLSVTIVEKDQKIQFVLLKSSLDDSAINVTAKST

L_borg_LEP1GSC103_3191 NGEFVLDHVPPGKYVLTASARGYQSETLSVTIGEKDQKTQFILLESSLDGSAINVTAKST

L_mayo_LEP1GSC190_2194 KGEFVLDHVPPGKYVLIASARGYQSETLSVTIGEKDQKAQFILLESSLDNSAINVTAKST

L_alex_LEP1GSC062_1651 KGEFVLDHVPPGKYVLTASARGYQSETLSVTIGEKDQKAQFVLLESSLDGSAINVTAKST

L_weil_LEP1GSC086_3478 KGEFVLDHVPPGKYILTASARGYQSETLSVTIGEKDQKAQFILLESSLDGFAINVTAKST

:***:*.******* : : :*.:.***:.: : :**:. ** * : *** *********

L_nogu_LEP1GSC059_2587 NSDFLTAPQPTTVLSGRQLDRQRGETAMSAVNNTPGVSNLTTGAGTSKPIIRGLTGQRVL

L_inte_LIC10964 NSDFLTAPQPITVLSGRQLDRQRGETAMSAINNTPGVSNLTTGSGTSKPIIRGLTGQRVL

L_kirs_LEP1GSC049_4224 NSDFLTAPQPITVLSGRQLDRQRGETAMSAINNTPGVSNLTTGAGTSKPIIRGLTGQRVL

L_alst_LEP1GSC193_3780 ISDFLTAPQPITVLSGRQLDRQRGESAMSAINNTPGVSNLTTGAGTSKPIIRGLTGQRVL

L_kmet_LEP1GSC052_2231 ISDFLTSPQPITVLSGRQLDRQRGENAMSAINNTPGVSNLTTGAGTSKPIIRGLTGQRVL

L_sant_LEP1GSC048_0454 ISDFLSSPQPITVLSGRQLDRQRGETAMSAINNTPGVSNLTTGAGTSKPIIRGLTGQRVL

L_borg_LEP1GSC103_3191 ISDFLTSPQPITVLSGRQLDRQRGETAMSAINNTPGVSNLTTGAGTSKPIVRGLTGQRVL

L_mayo_LEP1GSC190_2194 ISDFLTSPQPITVLSGRQLDRQRGETAMSAINNTPGVSNLTTGAGTSKPIIRGLTGQRVL

L_alex_LEP1GSC062_1651 ISDFLTSPQPITVLSGRQLDRQRGETAMSAINNTPGVSNLTTGAGTSKPIIRGLTGQRVL

L_weil_LEP1GSC086_3478 ISDFLTSPQPITVLSGRQLDRQRGETAMSAINNTPGVSNLTTGAGTSKPIIRGLTGQRVL

****::*** **************.****:************:******:*********

L_nogu_LEP1GSC059_2587 VMTDGIRQEEQQFGDDHTVELDSFNIQKIEIIRGPGSLLYGSDALGGVVNVIRDKAPLSG

L_inte_LIC10964 VMTDGIRQEEQQFGDDHTVELDSFNIQKIEIIRGPGSLLYGSDALGGVVNVIRDKAPLSE

L_kirs_LEP1GSC049_4224 VMTDGIRQEEQQFGDDHTVELDSFNIQKIEIIRGPGSLLYGSDALGGVVNVIRDKAPLSG

L_alst_LEP1GSC193_3780 VMTDGIRQEEQQFGDDHTVELDAFNIQKIEIIRGPGSLLYGSDALGGVVNVIRDKAPLSG

L_kmet_LEP1GSC052_2231 VMTDGIRQEEQQFGDDHTVELDAFNIQKIEIIRGPGSLLYGSDALGGVVNVIRDKAPLSG

L_sant_LEP1GSC048_0454 VMTDGIRQEEQQFGDDHTVELDSFNIQKIEIIRGPGSLLYGSDALGGVVNVIRDKAPLSG

L_borg_LEP1GSC103_3191 VMTDGIRQEEQQFGDDHTVELDSFNIQKIEIIRGPGSLLYGSDALAGVVNVIRDKAPRSG

L_mayo_LEP1GSC190_2194 VMTDGIRQEEQQFGDDHTVELDSFNIQKIEIIRGPGSLLYGSDALAGVVNVIRDKAPRSG

L_alex_LEP1GSC062_1651 VMTDGIRQEEQQFGDDHTTELDSFNIQKIEIIRGPGSLLYGSDALAGVVNVIRDKAPRSG

L_weil_LEP1GSC086_3478 VMTDGIRQEEQQFGDDHTTELDSFNIQKVEIIRGPGSLLYGSDALAGVVNVIRDKAPRSG

******************.***:*****:****************.*********** *

L_nogu_LEP1GSC059_2587 EGTPKMAGIFNSNSYSNNKQDAGNFAVYGNLNGFGYRASSNSRKAGRITTPNGTLRNTGM

L_inte_LIC10964 EGIPKMAGIFNSNSYSNNKQDAGNFAIYGNLNGFGYRASSNSRKAGKITTPNGTLRNTGM

L_kirs_LEP1GSC049_4224 EGIPRMAGIFNSNSYSNNKQDAGNFAIYGNLNGFGYRASSNSRKASRMTTPNGTLRNTGM

L_alst_LEP1GSC193_3780 EGVPKTAGLFNSNSYSNNKQDAGNFAVFGNIDGFGYRASSNVRKAGRITTPNGTLRNTGM

L_kmet_LEP1GSC052_2231 EGVPKMAGIFNSNSYSNNKQDAGNFAIYGNIDGFGYRASSNTRKAGRITTPNGTMRNTGM

L_sant_LEP1GSC048_0454 EGIPKMAGLFNSNNYSNNRQDTGNVAVYGNVEGFGYRASANTRKAGRITTPNGTLPNTGM

L_borg_LEP1GSC103_3191 EGVPQMAGLFNSNNYSNNKQDAGNFAIYGNVDGFGYHASANTRKAGRITTPNGTMPNTGM

L_mayo_LEP1GSC190_2194 EGVPKMAGFFNSNSYSNNKQDAENFAVYGNMDGFGYRASANTRKAGRITTPNGTMPNTGM

L_alex_LEP1GSC062_1651 EGVPKMAGIFNSNSYSNNKQDAGNFAIYGNMDGFGYRASANTRKAGRITTPNGTMPNTGM

L_weil_LEP1GSC086_3478 EGVPKMAGIFNSNSYSNNKQDAGNFAIYGNMDGFGYRASANTRKAGRITTPNGTMPNTGM

** *. **:****.****.**: *.*::**::****.**:* ***..:******: ****

L_nogu_LEP1GSC059_2587 MEKNQSASIGLNGDWGNFYLDSFRREQTQDLLDNPNENPGATVSQKLLHEKSHLHSFFIF

L_inte_LIC10964 IEKNQSASIGLDGDWGNFYLDSFKREQTQDLLDNPNENPGATVSQKLLHEKSHFHSFFIF

L_kirs_LEP1GSC049_4224 MEKNQSASIGLDGDWGNFYLDSFRREQTQDLLDNPNENPGATVSQKLFHEKSHFHSFFIF

L_alst_LEP1GSC193_3780 IEKNQSASVGLDGKWGNFYLDSFRREQTQDLLENPNENPGATVSQKLLHEKSHFHSFFIF

L_kmet_LEP1GSC052_2231 IEKNQSASIGSDGKWGNFYVDSFRREQTQDLLDNPNENPGATVFQKLLHEKSHFHSFFIF

L_sant_LEP1GSC048_0454 IEKNQNVSIGLDGKWGNFYLDSFRREQTQDLLDNPNENPGSTVYQKLLHEKSHFHSFLIF

L_borg_LEP1GSC103_3191 IEKNQNASIGLDGKWGNFYLDSFRREQTQDLLDNPNENPGSTVHQRLLHEKSHFHSFLIF

L_mayo_LEP1GSC190_2194 IEKNQNASIGLDGKWGNFYLDSSRREQTQDLLDNPNENPGSTVYQKLLHEKSHFHSFLIF

L_alex_LEP1GSC062_1651 IEKNQNASVGLDGKWGNFYLDSFRREQTQDLLDNPNENPGSTVYQKLLHEKSHFHSFLIF

L_weil_LEP1GSC086_3478 IEKNQNASVGLDGKWGNFYMDSFRREQTQDLLDNPNENPGSTVYQKLLHEKSHFHSFLIF

:****..*:* :*.*****:** .********:*******:** *.*:*****:***:**

L_nogu_LEP1GSC059_2587 SVGNLELDFSYQRNNRREIESKNKLLPIQNVLLDENTDILDKSFQFYQVTSREKYQGLNL

L_inte_LIC10964 SAGNLELDFSYQRNNRREIESKNKLLPIQDILLDKNAEVLDKSFQFYQVTSKEKYQGLNL

L_kirs_LEP1GSC049_4224 SAGNLELDFSYQRNNRREIESKNKLLPIQNVLLDENADVLDKSFQFYQVTSKEKYQGLNL

L_alst_LEP1GSC193_3780 SAGNLELDFSYQRNNRREIESKNKLIPIKDVLWDENADILDKSFQFYQATSRAAYQGLNL

L_kmet_LEP1GSC052_2231 SPGNLEIDLSYQRNNRREIESKNKLLPIKDVLLDESVDVFDKSFQFYQVTSRAKNQGLNL

L_sant_LEP1GSC048_0454 PAGNLELDFSYQRNNRREVESKNKLLPVKDVLWNENVDVFDKSFQFYQATSRAKYQGLNL

L_borg_LEP1GSC103_3191 PAGNLELDFSYQRNNRREIESKNKLFPIKDVLRDENVDIFDKSFQFYQATSKAKYQGLNL

L_mayo_LEP1GSC190_2194 PAGNLELDLSYQRNNRREIESKNKLLPIKDVLQNENVNIFDKSFQFYQATSKAKYQGLNL

L_alex_LEP1GSC062_1651 PAGNLELDFSYQRNNRREIESKNKLFPIKDVLRDENVDIFDKSFQFYQATYKAKRQGLNL

L_weil_LEP1GSC086_3478 PAGNLELDFSYQRNNRREIESKNKLFPIKDVLRDESVDILDKSFQFYQATYKAKYQGLNL

. ****:*:*********:******:*::::* ::..:::********.* . *****

L_nogu_LEP1GSC059_2587 FLDTATADAKFHHKPIFNFLKGTFGVSGLEQKNRTTGTEPLIPSYSIINVAGYFLEELKL

L_inte_LIC10964 FLDTTTADAKFHHKPIFNFLKGTIGISGLEQRNKTIGTEPLIPSYGIINIAGYFLEELKL

L_kirs_LEP1GSC049_4224 FLDTTTADAKFHHKPIFNFLKGTIGISGLEQKNRTIGTEPLIPSYGIINLAGYFLEELKL

L_alst_LEP1GSC193_3780 FLDTATAEAKFHHKPIFNFLKGTFGLSGLEQKNRTIGTEPLTPSYGIVNIAGYFLEELKL

L_kmet_LEP1GSC052_2231 FLDTATADAKFHHKPIFNLLKGTFGVSGLEQKNRTIGTEPLIPSYGIVNVAGYFLEELKL

L_sant_LEP1GSC048_0454 FLDTTTADAKFHHKPIFNLLQGTLGVSGLEQKNRTIGTESLIPSYGIVNIAGYFLEELKF

L_borg_LEP1GSC103_3191 LLDTTTADAKFHHKPIFNLLQGTLGVSGLEQRNRTIGTEPLIPSYGIVNIAGYFLEELKL

L_mayo_LEP1GSC190_2194 FLDTAIADVKFHHKPIFSLLQGTVGVSGSEQRNRTIGTEPLIPSYGIVNIAGYFLEELKL

L_alex_LEP1GSC062_1651 FLDTATADAKFHHKPIFNLLQGTLGVSGLEQRNRTIGTEPLIPSYGIVNIAGYFLEELKL

L_weil_LEP1GSC086_3478 FLDTATADAKFHHKPIFNLLQGTLGVSGLEQKNRTIGTEPLIPSYGIVNIAGYFLEELKL

:***: *:.********.:*:**.*:** **.*.* ***.* ***.*:*:*********:

L_nogu_LEP1GSC059_2587 GSLTFSAGIRGDKRSVDIRNNQTLSVSEQTKNYYMTTGSTGFVWRIDKSLSAVFNYGRGF

L_inte_LIC10964 GSLTFSAGIRGDKRSVDIRNNQTLGVSEQTKNYYMTTGSTGVVWRIYKSFSTIFNYGRGF

L_kirs_LEP1GSC049_4224 GSLTFSAGIRGDKRSVDVRNNQTLDVSEQTKNYYMTTGSTGLVWRIHKSFSAVFNYGRGF

L_alst_LEP1GSC193_3780 GSLTFSAGVRGDKRSAEIRNNETLAVAEQSKNYYATTGSTGLVWRIDKSFSAVFNFGRGF

L_kmet_LEP1GSC052_2231 GNVTLSAGVRADKRSADIRNNAALGVTERTKNYYATTGSTGLVWRIDKFFSTVLNYGRGF

L_sant_LEP1GSC048_0454 GSLTLSAGIRGDKRSANIRNNAALGVAEQTKNYYATTNSTGLVWRIDKSFSTIVNYGRGF

L_borg_LEP1GSC103_3191 GSLTLSAGVRGDKRSADIKNNMTLGIAEQTKNYYATTNSTGLVWRIDKSFSAILNYGRGF

L_mayo_LEP1GSC190_2194 GSLTLSAGVRGDKRSADIKNNMTLGIAEQTKNYYATTNSTGLVWRIDKSFSTILNYGRGF

L_alex_LEP1GSC062_1651 GSLILSAGVRGDKRSADIKNNTTLNITEQTKNYYATTNSTGLVWRINKSFSTILNYGRGF

L_weil_LEP1GSC086_3478 GSLILSAGIRGDKRSADIKNNTILGVTQQTKNYYATTNSTGLVWRIDKSFSAILNYGRGF

*.: :***:*.****.::.** * :::.:**** **.***.**** * :*::.*:****

L_nogu_LEP1GSC059_2587 RAPTPFELFSHGVHEGFGKFEIGNNYLKPEYSNNLDFSLRFASSKIQTEISVFQNHIQNF

L_inte_LIC10964 RAPTPFELFSYGVHEGTGKFEIGNNHLKPEYSNNLDFSLRFASSKIQTEISIFQNHIQNF

L_kirs_LEP1GSC049_4224 RAPSPFELFSHGVHEGTGKFEIGNNHLKPEYSNNLDFSLRFASSKIQTEISVFQNHIQNF

L_alst_LEP1GSC193_3780 RAPTPFELFSHGVHEGTGKFEIGKNTLKPEYSNNLDFSLRYGSSKIQTEISVFQNNIQNF

L_kmet_LEP1GSC052_2231 RAPTPFELFSNGVHEGTGKFEIGKDSLKPEYSNNLDLSFRYASSRIQTEISVFQNHIQNF

L_sant_LEP1GSC048_0454 RAPTPFELFSNGAHEGTGKFEIGKNSLKPEYSNNLDLSVRYASSRIQIEFGLFQNHIQNF

L_borg_LEP1GSC103_3191 RAPTPFELFSNGVHEGTGKFEIGKDSLKPEYSNNLDFSVRYASSRIQMEVNVFQNQIQNF

L_mayo_LEP1GSC190_2194 RAPTPFELFSNGVHEGTGKFEIGKDTLKPEYSNNLDFSIRYASSRIQMEVSVFQNHIQNF

L_alex_LEP1GSC062_1651 RAPTPFELFSNGVHEGTGKFEIGKDILKPEYSNNLDFSIRYASSRIQMEVSVFQNHIQNF

L_weil_LEP1GSC086_3478 RAPTPFELFSNGVHEGTGKFEIGKDTLKPEYSNNLDFSFRYASSRIQMEISVFQNHIQNF

***:****** *.*** ******:: **********:*.*:.**.** *..:***:****

L_nogu_LEP1GSC059_2587 IYAASIAEIDLDSGLPKYKYKQGNAVLKGGEFSFQAELFRKLIFSGGIDIVYSINQNDTH

L_inte_LIC10964 IYAASIAQIDLDSGLPKYEYKQGNAILKGGEFSIQAELFRKLVFSGGIDIVYSRNQSDTH

L_kirs_LEP1GSC049_4224 IYAASIAEINPDSGLPKYEYKQGNAVLKGGEFSIQAELFRKLIFSGGIDIIHSRNQNDTH

L_alst_LEP1GSC193_3780 IYAASIADIDADSGLPKYQYKQGDAILRGGEFSIQAALTRQLVLSGGIDIVHARNQNDTN

L_kmet_LEP1GSC052_2231 IYAASIAEIDVDSGLPKYRYKQGDAVLRGGEFSIQAELTRKLVLSGGIDIVHSRNQNDTN

L_sant_LEP1GSC048_0454 IYSTNIAEIDADTGLPKYRYKQGNAVLKGGEFSIQAELTDKLVFSGGIDVVHARNQNDTN

L_borg_LEP1GSC103_3191 IYAASIAEIDPNSGLPKYKYKQGNAVLKGGEFSIQAELTNKLVFSGGIDIVYARNQNDTN

L_mayo_LEP1GSC190_2194 IYSSSIAEIDPNSGLPKYRYKQGNAVLKGGEFSIQAELTSRLIFSGGIDIVYARNQNDTN

L_alex_LEP1GSC062_1651 IYAASIAEIDPDSGLPKYRYKQGNAVLKGGEFSIQAELTSKLVFSGGIDIVYARNQNDTN

L_weil_LEP1GSC086_3478 IYAASIAEIDPDSGLPKYRYKQGNAVLKGGEFSIQAELSSKLVFSGGIDIVYARNQNDTN

**::.**:*: ::***** ****:*:*.*****:** * .*::*****:::: **.**:

L_nogu_LEP1GSC059_2587 PLPRTTPNRARAGLRWTENSILGLKNFYFSINGRFYDSQYRIDPKETPTKSYNLMDVGLG

L_inte_LIC10964 PLPRTTPNRARAGLRWTEDSILGLKNFYFSINGRFYDSQYRVDPKETPTKGYNLIDAGLG

L_kirs_LEP1GSC049_4224 PLPRTTPNRARAGLRWTEDSILGLKNFYFSINGRFYDSQYRVDPKETPTKGYNLMDIGLG

L_alst_LEP1GSC193_3780 PLPRTTPNRARAGLRWTEDSILGLKDFYFSINGRFYDSQFRVDPKETPTKGYNLMDIGLG

L_kmet_LEP1GSC052_2231 PLPRTTPNRARAGLRWTEDSILGLKNFYVSVNGRFYDSQYRVDPKETPTKGYNLTDIGFG

L_sant_LEP1GSC048_0454 PLPRTTPNRVRAGLRWTESSILGLKDFYFSINGRFYDSQYRVDPKETPTKGYNLIDIGLG

L_borg_LEP1GSC103_3191 PLPRTTPNRARAGLRWTEGSILGLKDFYFSINGRFYDSQYRVDPKETPTKGYNLTDIGLG

L_mayo_LEP1GSC190_2194 PLPRTTPNRARAGLRWTESSILGLKDFYFSINGRFYDSQYRVDPKETPTKGYNLTDIGLG

L_alex_LEP1GSC062_1651 PLPRTTPNRARAGLRWTESSILGLKDFYFSINGKFYDSQYRVDPKETPTKGYNLTDIGLG

L_weil_LEP1GSC086_3478 PLPRTTPNRARAGLRWTENSILGLKDFYFSVNGRFYDSQYRVDPKETPTKGYNLTDIGLG

*********.********.******:**.*:**.*****:*:********.*** * *:*

L_nogu_LEP1GSC059_2587 FELPHFGDGSSKPTLDFSVQNVFNVSYVDHLSRYKDYALNPGLNAVLKISFPFTAIQ-

L_inte_LIC10964 FELPHFGDGTSKPTVDLSIQNVFNVSYVDHLSRYKDYALNPGLNAILKVSFPFTAIP-

L_kirs_LEP1GSC049_4224 FELPHFGDGTSNPTVDLSIQNVFNVSYVDHLSRYKEYALNPGLNAIFKVSFPFTAIQ-

L_alst_LEP1GSC193_3780 FELPYFGDGISKPTVDLSVQNVFNVSYVDHLSRYKDYALNPGVNAILKISFPFTAVP-

L_kmet_LEP1GSC052_2231 FELPNFGDGTSKPSLDFSVQNVFNVSYVDHLSRYKDYALNPGVNAILKVSFPFTMVQ-

L_sant_LEP1GSC048_0454 FELPHLGDGTSQPSVDLSVQNVFNVSYVDHLNRYKDYALNPGVNAILKISIPFTVGGL

L_borg_LEP1GSC103_3191 FELPYLGDGTSRPSVDLNVQNVFNVSYVDHLSRYKDYALNPGVNVILKISFPFTVVP-

L_mayo_LEP1GSC190_2194 FELPYLGDGTSQPSVDLSVQNVFNVSYVDHLSRYKDYALNPGLNVVLKISFPFTVVP-

L_alex_LEP1GSC062_1651 FELPYLGDGTSQPSVDLSVQNIFNVSYADHLSRYKDYALNPGVNAILKISFPFTVVP-

L_weil_LEP1GSC086_3478 FELPYLGDGTSQPSVDLSVQNVFNVSYVDHLSRYKDYALNPGVNAILKISFPFTVVP-
