# Supplementary material for: Discovery of Novel Leptospirosis Vaccine Candidates Using Reverse and Structural Vaccinology
Source: Front Immunol. 2017 Apr 27;8:463. doi: 10.3389/fimmu.2017.00463 (PMC5406399; doi:10.3389/fimmu.2017.00463)
Supplement: Supplementary file 8 [file Data_Sheet_1.ZIP › Alignment Bb-OMPs/Mult_alignment_LIC11086_path_spp_orthol_immun_epit_highlighted.docx]

L_kmet_LEP1GSC052_0701 MKQNILIFYILLLSFSFDVVEAHHTGMGGSEQSSTRFVDPFTGKREKPANYVVFTQDYYK

L_sant_LEP1GSC048_0761 MKQNILIFHFFIFLLCSFSLRAHHTGMGGSEQSSTRFVDPFTGKREKPANYFVVTQDFFK

L_alst_LEP1GSC193_0460 MKQNILILYFILFFTFLSSLEAHHTGMGGSEQSSTRFVDPFTGKREKPANYAVFTQDFFK

L_nogu_LEP1GSC059_1496 MKQNILIFYFILFLYFIIPLEAHHTGMGGSDQSSTRFVDPFTGKREKPANYMVLTQDFFK

L_kirs_LEP1GSC049_4303 MKQNILILYFIIFLSFIIPLEAHHTGMGGSDQSSTRFVDPFTGKREKPANYVVLTQDFFK

L_inte_LIC11086 MKQNILIFYFILFLYFIIPLEAHHTGMGGSDQSSTRFVDPFTGKREKPANYVVLTQDFFK

L_borg_LEP1GSC103_3999 MKQNILIFHFFFFFLCLFSLEAHHTGMGASEQSFTRFVDPFTGKREKPANYVVFTQDFFK

L_weil_LEP1GSC086_1478 MKHNIIILYFFIFLPCIFSLEAHHTGMGGSEQSSTRFVDPFTGKREKPANYVVFTQDFFK

L_mayo_LEP1GSC190_3404 MKQNILIFHFFFFFLCVFSLEAHHTGMGGSEQSSTRFVDPFTGKREKPANYVVFTQDFFK

L_alex_LEP1GSC062_1096 ------------------------------------------------------------

L_kmet_LEP1GSC052_0701 QTNENSNIHTTTFYGEMNLKNGMFALNLSTPYTYYEQKDRSDAARIGKTYIGIKYLPLID

L_sant_LEP1GSC048_0761 QTNENSNIHTTTVFGETNLKNGMFALNLSVPYTYYEQKRRSDAARIGKTYIGIKYLPLAD

L_alst_LEP1GSC193_0460 QTNENSNIHTTTFFGEMNLKNGMFALNLSTPYTYYEQKNRSDAARIGKTYVGVKYLPLVD

L_nogu_LEP1GSC059_1496 QTNENSNIHTSTFFGEINLKNGMFALNLSVPYTYYEQKDRSDAARIGKTYIGIKYLPLVD

L_kirs_LEP1GSC049_4303 QTNENSNIHTSTFFGEINLKNGMFALNLSVPYTYYEQKDRSDAARIGKTYIGIKYLPLVD

L_inte_LIC11086 QTNENSNIHTSTFFGEINLKNGMFALNLSVPYTYYEQKDRSDAARIGKTYIGIKYLPLVD

L_borg_LEP1GSC103_3999 QTNENSNIHTTTFFGEMNLKNGMFALNLSTPYTYYEQKNRSDAARIGKTYFGIKYLPLID

L_weil_LEP1GSC086_1478 QTNENSNIHTTTFFGEMNLKNGMFALNLSAPYTYYEQKNRSDAARIGKTYVGVKYLPLVD

L_mayo_LEP1GSC190_3404 QTNENSNIYTTTFFGEMNLKNGMFALNLSTPYTYYEQKNRSDAARIGKTYVGVKYLPLID

L_alex_LEP1GSC062_1096 ------------------------------------------------------------

L_kmet_LEP1GSC052_0701 FQKNYFVVLSANVGFPSGPDTDKFTGGNYYSGIPGLTLGYLAGKFSFVGRISGIFPLSRS

L_sant_LEP1GSC048_0761 FQKNYFIVLSANVGFPSGPDTDRFTGGNYYSGIPGLTLGYLWGKFSFVGRISGIFPLSKS

L_alst_LEP1GSC193_0460 FQKNYFIVLSANVGFPSGPDTDRFTGGNYYSGIPGLTFGYLLGKFSFVGKISGIFPLSRS

L_nogu_LEP1GSC059_1496 FQKNYFIVFSANVGFPSGPDTDKFTGGNYYSGIPGLTFGYLLGKFSFVGKVSGIFPLSKS

L_kirs_LEP1GSC049_4303 FQKNYFIVFSANVGFPSGPDTDKFTGGNYYSGIPGLTFGYLLGKFSFVGKVSGIFPLSKL

L_inte_LIC11086 FQKNYFIVFSANVGFPSGPDTDKFTGGNYYSGIPGLTFGYLLGKFSFVGKLSGIFPLSKS

L_borg_LEP1GSC103_3999 FQKNYFIVLSANVGFPSGPDTDRFTGGNYYSGIPGLTLGYLLGKFSFVGRISGIFPLSKS

L_weil_LEP1GSC086_1478 FQKNYFIVLSANVGFPSGPDTDKFTGGNYYSGIPGLTLGYLLGKFSFVVRISGIFPLSKS

L_mayo_LEP1GSC190_3404 FQKNYFIVLSANIGFPSGPDTDRFTGGNYYSGIPGLTLGYLLGKFSFVGRISGIFPLSKS

L_alex_LEP1GSC062_1096 -------------------------------------MGYLLGKFSFVGRISGIFPLSKS

:*** ****** .:*******.

L_kmet_LEP1GSC052_0701 QPSNLQDNDGIPYWLRTPSSAAPQDPYLLKKTTLFSGYITYLWKPGLSFFTGILYRTPYE

L_sant_LEP1GSC048_0761 RPANLQDNDGIEYWFRNPSSLPPEETYLLKKTSLFSGYVTYLWKPGLSFFTGFLYRTPYE

L_alst_LEP1GSC193_0460 KPSNLQDNDGIVYWLRNPSFSAPEETYLLKKTSIFSGYVTYLWKPGLSFFTGFLYRTPYE

L_nogu_LEP1GSC059_1496 QPYNLQDNDGIVYWLRNPSSSPPEEMYLLKKTSLFSAYVTYLWKPGLSFFTGFLYRTPYE

L_kirs_LEP1GSC049_4303 QPSNLQDNDGIVYWLRNPSSSPPEETYLLKKTSLFSGYVTYLWKPGLSFFTGFLYRTPYE

L_inte_LIC11086 QPSNLQDNDGIVYWLRNPSSSPPEETYLLKKTSLFSGYVTYLWKPGLSFFTGFLYRTPYE

L_borg_LEP1GSC103_3999 QPTNLQDNDGIVYWLRNPSSLPPEETYILKKTSLFSGYVTYLWKSGFSFFTGFLYRTPYE

L_weil_LEP1GSC086_1478 QPTNLQDNDGIVYWLRNPSSSPPEETYLLKKTSLFSGYVTYLWKPGLSFFTGFLYRSPYE

L_mayo_LEP1GSC190_3404 QPTNLQDNDGIFYWLRNPSSSPPEETYLLKKTSLFSGYVTYLWKSGLSFFAGFLYRTPYE

L_alex_LEP1GSC062_1096 QPTNLQDNDGIIYWLRNPSSSPPEETYLLKKTSLFSGYVTYLWKPGLSFFTGFLYRTPYE

.* ******** **:*.** .*:: *:****::**.*:*****.*:***:*:***:***

L_kmet_LEP1GSC052_0701 GVDLKRTDQGKVPSIFREVSLGFSANISEKLNFNLSYRYPLYRGDEYRLYDYALTAAVSI

L_sant_LEP1GSC048_0761 GVDLKRTNQGKVPTIFREISFGFSANISEKLNFNLSYRYPLYRGDDYRLYDYALTAAISI

L_alst_LEP1GSC193_0460 GVDLKRTDQGKVPAIFREVSFGFSANISEKLNFNLSYRYPLYRGDDYRLYDYAITAAVSI

L_nogu_LEP1GSC059_1496 GVDLKRSSQGKVPSIFREVSLGFSANISEKLNFNLSYRHPLYRGEDYRLYDYAITAAVSI

L_kirs_LEP1GSC049_4303 GVDLKRSNQGKVPSIFREVSLGFSANISEKLNFNLSYRHPLYRGEDYRLYDYAITAAVSI

L_inte_LIC11086 GVDLKRSNQGKVPSIFREISLGFSANISEKLNFNLSYRYPLYRGEDYRLYDYAITAAVSI

L_borg_LEP1GSC103_3999 GVDFKRTNQGKVPTIFREVSFGFSANISEKLNFNLSYRHPLYRGNDYRLYDYAITAAISI

L_weil_LEP1GSC086_1478 GVDLKRTNQGKVPAIFREVSFGFSANISEKLNFNLSYRYPLYRGDDYRLYDYAITAAVSV

L_mayo_LEP1GSC190_3404 GVDLKRTNQGKVPTIFREVSFGFSANISEKLNFNLSYRHPLYRGDDYRLYDYAITAAVSI

L_alex_LEP1GSC062_1096 GVDLKRTNQGKVPAIFREVSFGFSANISEKLNFNLSYRHPLYRGDDYRLYDYAITAAVSI

***:**:.*****:****:*:*****************:*****::*******:***:*:

L_kmet_LEP1GSC052_0701 EISELENSNEAETKKETKADSDPKEDVPKESTDTEKNSKETGDRN

L_sant_LEP1GSC048_0761 EISELENSK---------------EEVKKESAELETPQNTK----

L_alst_LEP1GSC193_0460 EISELENSK------------------------------------

L_nogu_LEP1GSC059_1496 EISELENSKPT-----------KVEEVKKEPDETTQETK------

L_kirs_LEP1GSC049_4303 EISELENSKPV-----------KLEEVKQEPEETTQETK------

L_inte_LIC11086 EISELENSKPA-----------KVEEVKQEPEETTQETK------

L_borg_LEP1GSC103_3999 EISELENSGSK-----------LKEEVKKESTELETP--------

L_weil_LEP1GSC086_1478 EISDLESSKPK-----------TEEEVKRESTEPENPQNAK----

L_mayo_LEP1GSC190_3404 EISGLENSEPK-----------PKEEVKKESTEP-----------

L_alex_LEP1GSC062_1096 EISELENSK------------------------------------

*** **.*
