# Supplementary material for: Discovery of Novel Leptospirosis Vaccine Candidates Using Reverse and Structural Vaccinology
Source: Front Immunol. 2017 Apr 27;8:463. doi: 10.3389/fimmu.2017.00463 (PMC5406399; doi:10.3389/fimmu.2017.00463)
Supplement: Supplementary file 8 [file Data_Sheet_1.ZIP › Alignment Bb-OMPs/Mult_alignment_LIC11211_path_spp_orthol_immun_epit_highlighted.docx]

L_kirs_LEP1GSC049_1709 -----MIFKKNPL---------LVSSFTLLILLSFSLSAQEQRKSTFLGSSLIHMPSTED

L_nogu_LEP1GSC059_1611 -----MNFKKSPF---------LIFFFLLFILLSFSLSAQEQRKSTFLGSSLIHMPSTED

L_inte_LIC11211 -----MIFKKNPF---------LISFFLLFILLPFSLSAQEQRKSTFLGSSLIHMPSTED

L_sant_LEP1GSC048_3209 -----MNFKVNWF---------LIPLFLSPIL----LSAQKREQSAFLGSSLIHMPSTED

L_alex_LEP1GSC062_3292 -----MNFRINFS---------LIPFLILSISFSFVLSAQERRKSAFLGSSLIHMPSTED

L_alst_LEP1GSC193_4480 -----MNFKINFS---------LISFFILSISFSFALFGQEQRKSAFLGSSLIHMPSTED

L_mayo_LEP1GSC190_3055 -----MNFKINSF---------LILLFLPL----FALSAQEQKKSAFLGSSLIHMPSTED

L_weil_LEP1GSC086_4170 -----MNFRINFS---------LIPFLILSTSFSFALFAQEQGRSAFLGSSLIHMPSTED

L_borg_LEP1GSC103_1191 MANQRIKFQKTPFSLAAPFPLRLTFLISVFLFFSVSLSAQEQKKSAFLGSSLIHMPSTED

L_kmet_LEP1GSC052_2469 -----MNSKIRFV---------VFLLFSVFLFVSFSLSAQEQRKSTFLGTSLIHMPSTED

: . : : * .*:. .*:***:**********

L_kirs_LEP1GSC049_1709 VGKNGLDFRFNHRFGNAKSTSYDFFGLDNGANTQLSLDYGLTDRITIGIARTSFQKTYEA

L_nogu_LEP1GSC059_1611 VGKSGLDFRFNHRFGNAKSTSYDFFGLDNGANTQLSLDYGLTDRITIGIARTSFQKTYEA

L_inte_LIC11211 VGKNGLDFRFNHRFGNAKSTSYDFFGLDNGANTQLSLDYGLTDRITIGIARTSFQKTYEA

L_sant_LEP1GSC048_3209 VGKNGLDFRFNHRFGNAKSASYDFFGLDNGANTQLSLDYGLTDRITVGIARTSFQKTYEV

L_alex_LEP1GSC062_3292 VGKNGLDFRINHRFGSAKSASYDFLGLDNGANTQLSLDYGLTDRITIGIARTSFQKTYEA

L_alst_LEP1GSC193_4480 VGKNGLDFRINHRFGSAKSTSYDFAGLDNGANTQLSLDYGLTDRITIGIARTSFQKTYEA

L_mayo_LEP1GSC190_3055 VGKNGLDFRFNHRFGNAKSASYDFLGLDNGANTQLSLDYGLTNRITIGIARTSFQKTYEA

L_weil_LEP1GSC086_4170 VGKSGLDFRFNHRFGNAKSASYDFLGLDNGANTQLSLDYGLTDRITIGIARTSFQKTYEA

L_borg_LEP1GSC103_1191 VGKNGLDFRFNHRFGNAKSASYDFLGLDNGANTQLSLDYGVTDRLTIGIARTSFQKTYEA

L_kmet_LEP1GSC052_2469 VGKNGLDFRFNHRFGDAKSTSYDFLGLDNGANTQLSLDYGVTDRVTLGVARTSFQKTYEA

***.*****:*****.***:**** ***************:*:*:*:*:**********.

L_kirs_LEP1GSC049_1709 RGKIRLLTQDSNFPVTISFFGVFGQETEEQSKFYGPYLKASTGFPGFDSQLEKRLNTYEL

L_nogu_LEP1GSC059_1611 RGKIRLLTQDSSFPVTISFFGVFGQETEEQSKFYGPYLKVSTGFPGFNSQLEKKLNTYEL

L_inte_LIC11211 RGKIRLLTQDSNFPVTISFFGVFGQETEEQSKFYGPYLKVSTGFPSFDSQLEKKLNTYEL

L_sant_LEP1GSC048_3209 RGKIRLLTQDSGFPVTVSFFGVFGQETAKQRTFYGPYLKISSGFPIVDSEANRRLNTYEL

L_alex_LEP1GSC062_3292 RGKIRLLTQDSGFPVTVSFFGVFGQETAKQNTFYGPYLKISSGYPTVDFEANKKLNTYEL

L_alst_LEP1GSC193_4480 RGKIRLLTQDSGFPVTVSFFGVFGQETEKQNRFYGPYLKVSSGYPGFDSQAGRKLNTYEL

L_mayo_LEP1GSC190_3055 RGKIRLITQNSNFPVTVSFFGVFGQETAKQNIFYGPYLKISSGYPTVDSEANKRLNTYEL

L_weil_LEP1GSC086_4170 RGKIRLITQNSNFPITVSFFGVFGQETEKQNTFYGPYLKISSGYPTVDSEANKRLNTYEL

L_borg_LEP1GSC103_1191 RGKIRLLTQDSSFPVTISFFGVFGQETEKQEKFYGPYLKVSTGLSTFDPEATKKLNTYEL

L_kmet_LEP1GSC052_2469 RGKVRLLTQDSSFPITVSFFGVFGQETQEQRMFYGPYLKVSSGNAAFDSDATKKLNTYEL

***:**:**:*.**:*:********** :* ******* *:* . .: : ..******

L_kirs_LEP1GSC049_1709 SYSDRQSSLASFLVSRRFGDVFSLQLSPMFVHRNFVKEHLSNDRTGLDVSFRIHVFKRLD

L_nogu_LEP1GSC059_1611 SYSDRQSSLVSFLISKRFGDVFSLQLSPMFVHRNFVKEHLSNDRTGLDVSFRIHVFKRLD

L_inte_LIC11211 SDSDRQSALASFLISKRFGDLFSLQLSPMFVHRNFVKEHLSNDRTGLDVSFRIHVFKRLD

L_sant_LEP1GSC048_3209 SYSDRQSTLVSFLISKRFSDFFSLQLSPMFVHRNFVKEHLSNDRTGLDVSFRIHLFKRLD

L_alex_LEP1GSC062_3292 SYSDGQSTLASFLISRRFSDFFSLQLSPMFVHRNFVKDHLSNDRTGLDVSFRIHLFKRLD

L_alst_LEP1GSC193_4480 TDSDRQSALASFLISRRFGDLFSIQLSPMFVHRNFVKDHLSNDRTGLDVSFRIHLFKRLD

L_mayo_LEP1GSC190_3055 NYSDRQSTLVSFLISRRFSDFFSLQLSPMFVHRNFVKEHLSNDRTGLDVSFRIHLFKRLD

L_weil_LEP1GSC086_4170 SDSDRQSTLTSFLISRRFSDFFSLQLSPMFVHRNFVKEHLSNDRTGLDVSFRIHLFKRLD

L_borg_LEP1GSC103_1191 TYPDRQSMLASFLISRRFGEVFSLQLSPMFVHRNFVKEHISNDRMGLDVSFRIHLFKRLD

L_kmet_LEP1GSC052_2469 SYSDRQSTLVSFLISRKFSDYFSLQLSPMFVHRNFVKDHLSNDRSGLDVSFRIHLFKRLD

. .* ** *.***:*..*.: **:*************:*:**** *********:*****

L_kirs_LEP1GSC049_1709 FTFGTILTPKRDYIGDSYTAEDRKTKINGLEYSTSEANELIANGRTLDVIINNILLSKPV

L_nogu_LEP1GSC059_1611 FTFGTILTPKRDYIGDSYTTEDRKTKINGLEYSTSEANELIANGRTLDVIVNNILLSKPV

L_inte_LIC11211 FTFGTILTPKRDYIGDSYTAEDRKTKINGLEYSTSEANELIANGRTLDVIVNNILLSKPV

L_sant_LEP1GSC048_3209 FTFATILTPKRDYIGDSYSEENRKTKINGAKYSASEINDLIATGRTLDAISNNLLLSKSV

L_alex_LEP1GSC062_3292 FTFGTILAPKRDYIGDSYAAEDRKTKINGVEYSVSEVNDLIANGRTLDAIVNNILLSKPV

L_alst_LEP1GSC193_4480 FTFGTILTPKRDYIGDSYAAEDRKTKINGVEYSASEVNDLIASGRTLDAIVNNILLSKPV

L_mayo_LEP1GSC190_3055 FTFGTIFSPKRDYIGYSYSEESRKTKINGVEYSASEINDLIANGKTLDAIINNILFSKPV

L_weil_LEP1GSC086_4170 FTFGTILSPKRDYFGHSYSEENRKTKINGAKYSASEINDLIANGRTLDAIVNNILLSKPV

L_borg_LEP1GSC103_1191 FTFGTILTSKRDYIGDSYAAENRKTKINGVEYSASEVNDLIARGKTIDTVINNILLSKPV

L_kmet_LEP1GSC052_2469 FTFGTILTPKRDYIGDSYATEDRKTKINGVEYSASEVNDLIARGKTIDAAINNILLSKPV

***.**::.****:* **: *.******* :**.** *:*** *.*:*. **:*:**.*

L_kirs_LEP1GSC049_1709 EYMSVPMSFGVDFETGGHVFQLFVTNSRSIAHTQLLRGADYDYDKKEWTLGFNIHRYFSL

L_nogu_LEP1GSC059_1611 EYMSVPMSFGVDFETGGHVFQLFVTNSRSIAHTQLLRGADYDYNKKEWTLGFNIHRYFSL

L_inte_LIC11211 EYMSVPMSFGVDFETGGHVFQLFVTNSRSIAHTQLLRGADYNYDKKEWTLGFNIHRYFSL

L_sant_LEP1GSC048_3209 EYMSVPLSFGVDFETGGHVFQLFVTNSRSIAHTQLLRGADYDYYKKEWTLGFNIHRYFSL

L_alex_LEP1GSC062_3292 EYMSVPLSFGVDFETGGHVFQLFVTNSRSIAHTQLLRGADFDYDKKEWTLGFNIHRYFSL

L_alst_LEP1GSC193_4480 KYMSVPLSFGVDFETGGHVFQLFVTNSRSIAHTQLLRGADFDYDKKEWTLGFNIHRYFSL

L_mayo_LEP1GSC190_3055 EYMSVPLSFGIDFETGGHVFQLFVTNSRSIAHTQLLRGADYDYYKKEWTLGFNIHRYFSL

L_weil_LEP1GSC086_4170 EYMSVPLSFGVDFETGGHVFQLFVTNSRSIAHTQLLRGADYDYYKKEWTLGFNIHRYFSL

L_borg_LEP1GSC103_1191 EYMSVPLSFGVDFETGGHVFQLFVTNSRSIAHTQLLRGADYDYSKKEWTLGFNIHRYFSL

L_kmet_LEP1GSC052_2469 EYMTVPLSFGVDFETGGHVFQLFVTNSRTIAQTQLLRGADYDYGKKEWTVGFNIHRYFSL

:**:**:***:*****************:**:********::* *****:**********

L_kirs_LEP1GSC049_1709 ESQDS----KTN

L_nogu_LEP1GSC059_1611 ESQDS----KTN

L_inte_LIC11211 ESQDS----KTN

L_sant_LEP1GSC048_3209 ED----------

L_alex_LEP1GSC062_3292 ESSDN-------

L_alst_LEP1GSC193_4480 ESSDNSSVPKTD

L_mayo_LEP1GSC190_3055 QDSDN-------

L_weil_LEP1GSC086_4170 QGSDN-------

L_borg_LEP1GSC103_1191 ESSNN-------

L_kmet_LEP1GSC052_2469 ESSDNNTAQKSN

:.
