# Supplementary material for: Discovery of Novel Leptospirosis Vaccine Candidates Using Reverse and Structural Vaccinology
Source: Front Immunol. 2017 Apr 27;8:463. doi: 10.3389/fimmu.2017.00463 (PMC5406399; doi:10.3389/fimmu.2017.00463)
Supplement: Supplementary file 8 [file Data_Sheet_1.ZIP › Alignment Bb-OMPs/Mult_alignment_LIC11268_path_spp_orthol_immun_epit_highlighted.docx]

L_nogu_LEP1GSC059_1672 MKKNVTFKVLTGVLFLCCVIPFNLFSQNSKKEKT---NGPVLNLDPPSDVKYEEQLNDGK

L_inte_LIC11268 MKKNAIFKVSAGALLFCCIIPLNLFSQNSKKEKT---NGPVLNLDPPSDVRYEEQPNDGK

L_kirs_LEP1GSC049_1107 MKKNIIFKISTGALLLCCIIPLNLFSQSSKKEKT---NGPVLNLDPPSDVKYEEQPNDGK

L_kmet_LEP1GSC052_1816 --------MTTGALLLCTF-TIALFPQDTKKEKPKTESAPILNLDPPSDVKYEDQPNDGK

L_alst_LEP1GSC193_4423 -MKHTRIQLAAGILFLCVI-PLSLFSQSAKKEK----PDPVLNLDPPSNVKYEEQPNDEK

L_sant_LEP1GSC048_3345 --------MTAGVLFLCAM---SLFSQDTAKEKKASPPGPVLNLDPPSHIKYEEQANDGK

L_borg_LEP1GSC103_1279 -MKHTKIKMTAGILFLCTIPPLSLFSQDIPKEKKSS-PSPVLNLDPPSNVKYEEQANDGK

L_alex_LEP1GSC062_3352 -MKHTKIKMTAGILFLCAI-PLSLFSQDIVKEKKST-PGPVLNLDPPSNIKYEEQANDGK

L_mayo_LEP1GSC190_3001 -MKHTKIKMTAGILFLCAI-PLSLFSQDITKEKKST-SGPVLNLDPPSNVKYEEQTNDGK

L_weil_LEP1GSC086_4105 -MKHTKIKMTAGILFLCAI-PLSLFSQDITKEKKST-PGPVLNLDPPSNIKYEEQANDGK

: :* *::* . **.*. *** *:******* :.**:* ** *

L_nogu_LEP1GSC059_1672 GIPEIQEELAKEEPYKSPYKGKLPGEFMKSMLLSPEHQEAVRRTDKLWFGDIFRTGFQVR

L_inte_LIC11268 GIPEIQEELAKEEPYKSPYKGKLPGEFMKSMLLSPEHQEAIRRTDKLWFGDIFRTGFQIR

L_kirs_LEP1GSC049_1107 GIPEIQEELAKEEPYKSPYKGKLPGEFMKSMLLSPEHQEAVRRTDKLWFGDIFRAGFQVR

L_kmet_LEP1GSC052_1816 GIPEIQQELAKEEPYKSPYKGKLPGEFMKSMLLSPDHQDAVRKVDRLWLGDIFRVGFQIR

L_alst_LEP1GSC193_4423 GIPEIQQELAKEEPYKSPYKGKLPGEFMKSMLLSPEHQDAVRKTDRLWFGDIFRTGFQIR

L_sant_LEP1GSC048_3345 GIPEIQQELAKEEPYKSPYKGKLPGEFMKSMLLSPEHQDAMRKTDRLWFGDMFRAGFQVR

L_borg_LEP1GSC103_1279 GIPEIQQELAKEEPYKSPYKGKLPGEFMKSMLLSPEHQDAMRKTDRLWFGDIFRAGFQVR

L_alex_LEP1GSC062_3352 GIPEIQQELAKEEPYKSPYKGKLPGEFMKSMLLSPEHQDAVRKTDRLWFGDIFRAGFQVR

L_mayo_LEP1GSC190_3001 GIPEIQQELAKEEPYKSPYKGKLPGEFMKSMLLSPEHQDAMRKTDRLWFGDIFRAGFQVR

L_weil_LEP1GSC086_4105 GIPEIQQELAKEEPYKSPYKGKLPGEFMKSMLLSPEHQDAMRKTDRLWLGDIFRAGFQVR

******:****************************:**:*:*..*.**:**:**.***:*

L_nogu_LEP1GSC059_1672 PRFDYSHNADFDKRTQDDRNYATQNSQVFFVVDPNPYVAAKVTIQDVRVFGGEQSRKDGQ

L_inte_LIC11268 PRFDYSHNADFDKRTQDDRNYATQNSQVFFVVDPNPYVAAKVTIQDVRVFGGEQSRKDGQ

L_kirs_LEP1GSC049_1107 PRFDYSHNADFDKRTQDDRNYATQNSQVFFVIDPNPYVAAKVTIQDVRVFGGEQSRKDGQ

L_kmet_LEP1GSC052_1816 PRFDYSHNADFDKRTLDDRNYATQNSQVSFIIDPNQYVAAKVTIQDVRVFGGEQSRKDGQ

L_alst_LEP1GSC193_4423 PRFDYSHNADFDKRTQDDRNYATQNSQVFFVIDPNPYVAAKVTIQDVRVFGGEQSRKDGQ

L_sant_LEP1GSC048_3345 PRFDYSHNADFDKRTQDDRNFATQNSQVFFIIDPNPYVAAKVTIQDVRVFGGEQSRKDGQ

L_borg_LEP1GSC103_1279 PRFDYSHNADFDKRTQDDRNFATQNSQVFFVIDPNPYVAAKVTIQDVRVFGGEQSRKDGQ

L_alex_LEP1GSC062_3352 PRFDYSHNADFDKRTQDDRNFATQNSQVFFAIDPNPYVAAKVTIQDVRVFGGEQSRKDGQ

L_mayo_LEP1GSC190_3001 PRFDYSHNADFDKRTQDDRNFATQNSQVFFVIDPNPYVAAKVTIQDVRVFGGEQSRKDGQ

L_weil_LEP1GSC086_4105 PRFDYSHNADFDKRTQDDRNFATQNSQVFFVIDPNPYVAAKVTIQDVRVFGGEQSRKDGQ

*************** ****:******* * :*** ************************

L_nogu_LEP1GSC059_1672 LGYLGLSNSAGIELNSAPTTNNSVSIKNNTDLREGFVQLKNFANGFEIFIGRQIFGFGDN

L_inte_LIC11268 LGYLGLSNSAGIELSSAPTANNSVSIKNNTDLREGFVQLKNFADGFEIFIGRQIFGFGDN

L_kirs_LEP1GSC049_1107 LGYLGLSNSAGIELSSAPTANNSISIKNNTDLREGFVQLKNFADGFEIFIGRQIFGFGDN

L_kmet_LEP1GSC052_1816 LGYLGLSNSAGVELSAAPTATNSVSIKNNTDLREGFIQLKNFAEGFEVYLGRQIFGFGDN

L_alst_LEP1GSC193_4423 LGYLGLSNSAGTELSAAPTATNSVSIKNNTDLREGFIQLKNFADGFEIFLGRQIFGFGDN

L_sant_LEP1GSC048_3345 LGYLGLSNSAGAELSAAPTATNSVSIKNNTDLREGFVQLKNFAEGFEVFIGRQIFGFGDN

L_borg_LEP1GSC103_1279 LGYLGLSNSAGAELSAAPTATNSVSIKNNTDLREGFVQLKNFAEGFEVFIGRQIFGFGDN

L_alex_LEP1GSC062_3352 LGYLGLSNSAGAELSTAPTATNSVSIKNNTDLREGFVQLKNFAEGFEVFIGRQIFGFGDN

L_mayo_LEP1GSC190_3001 LGYLGLSNSAGAELSAAPTATNSVSIKNNTDLREGFVQLKNFAEGFEVFIGRQIFGFGDN

L_weil_LEP1GSC086_4105 LGYLGLSNSAGAELSAAPTATNSVSIKNNTDLREGFVQLKNFAEGFEVFIGRQIFGFGDN

*********** **.:***:.**:************:******:***:::**********

L_nogu_LEP1GSC059_1672 RYVGGRNDGQTGNSFDGARLKYNSKYFNSEAFTSIIAEDSNAGAGNNTANGVKRGTVNDT

L_inte_LIC11268 RYVGGRNDGQTGNSFDGARVKYNSKYFNSEAFTSIIAEDSNAGAGNNTANGVKRGTVNDT

L_kirs_LEP1GSC049_1107 RYVGGRNDGQTGNSFDGARVKYNSKYFNSEAFTSIIAEDSNAGAGNNTANGVKRGTVNDT

L_kmet_LEP1GSC052_1816 RYIGGRNDGQTGNSFDGARVKYNSKHFNSEAFTSIIAEDSNAGSGNNTANGVKRGTVNDT

L_alst_LEP1GSC193_4423 RYVGGRNDGQTGNSFDGARVKYNSKYFNSEAFTSVIAEDSNAGSGNNTANGVKRGTVNDT

L_sant_LEP1GSC048_3345 RYVGGRNDGQTGNSFDGARVKYNSKHFNSEAFTSIIAEESNAGSGNNTANGIKRGTVNDT

L_borg_LEP1GSC103_1279 RYVGGRNDGQTGNSFDGARVKYNSKYFNSEAFTSIIAEDSNAGSGNNTANGVKRGTVNDT

L_alex_LEP1GSC062_3352 RYVGGRNDGQTGNSFDGARVKYNSKYFNSEAFTSIIAEDSNAGSGNNTANGVKRGTVNDT

L_mayo_LEP1GSC190_3001 RYVGGRNDGQTGNSFDGARVKYNSKYFNSEAFTSIIAEDSNAGSGNNTANGVKRGTVNDT

L_weil_LEP1GSC086_4105 RYVGGRNDGQTGNSFDGARVKYNSKYFNSEAFTSIIAEDSNAGSGNNTANGVKRGTVNDT

**:****************:*****:********:***:****:*******:********

L_nogu_LEP1GSC059_1672 YLSGLYNTVKFEDFHVDLYYFNVDRKWEQGPNPVTSQDRTRQRDDLNTVGFRLTNRTDNN

L_inte_LIC11268 YLSGLYNTVKFEDFNVDLYYFNVDRKWEQGPNPVTSQDRTRQRDDLNTVGFRLTNRTDNN

L_kirs_LEP1GSC049_1107 YLSGLYNTVKLEDFNLDLYYFNVDRKWEQGPNPVTSQDRTRQRDDLNTVGFRLTNRTDNN

L_kmet_LEP1GSC052_1816 YLSGLYNTLKFEDFLVDLYYFNIDKKWEQGPTPTSSLDRTRQRDDLNTVGFRLTNRTDSN

L_alst_LEP1GSC193_4423 YLSGLYNTVKFEDFLVDLYYFNIDKKWEQGPNPSTSMDRTRQRDDLNTVGFRLSNRTDNN

L_sant_LEP1GSC048_3345 YLSGLYNTVKFEDFLVDLYYFNIDKKWEQGPNPTTSLDRTRQRDDLNTVGFRLTNRTDNN

L_borg_LEP1GSC103_1279 YLSGLYNTVKLEDFLVDFYYFNIDKKWEQGPNPTTSLDRTRQRDDLNTVGLRLTNRTDNN

L_alex_LEP1GSC062_3352 YLSGLYNTVKFEDFLVDFYYFNIDKKWEQGPNPTTSLDRTRQRDDLNTVGFRLTNRTENN

L_mayo_LEP1GSC190_3001 YLSGLYNTVKFEDFLVDFYYFNIDKKWEQGPSPTTSLDRTRQRDDLNTVGFRLTNRTDSN

L_weil_LEP1GSC086_4105 YLSGLYNTVKFEDFLVDFYYFNIDKKWEQGPNPTTSLDRTRQRDDLNTVGFRLTNRTDSN

********:*:*** :*:****:*.******.* :* *************:**:***:.*

L_nogu_LEP1GSC059_1672 RLPKTKAWDWTLEASWQYGYNGQRVNAGWDTLKQTVDGNPNSKRLYTERVEYDSKFFIAQ

L_inte_LIC11268 RLPKTKAWDWTLEASWQYGYNGQRVNAGWDTLKQTVDGNPNSKRIYTERVEYDSKFFIAQ

L_kirs_LEP1GSC049_1107 RLPKTKAWDWTLEASWQYGYNGQRVNAGWDTLKQTVDGNPNSKRLYTERVEYDSKFFIAQ

L_kmet_LEP1GSC052_1816 RLPKTKAWDWTLEGSWQYGFNGQRVNAGWDTFKQTVDGKSTSQRIYTQNVEYDAKYFIAQ

L_alst_LEP1GSC193_4423 RLPKTKAWDWTLEASWQYGFNGQRINAGWDTFKQTVDGNPKSQRIHTQNVEYDSKFFIAQ

L_sant_LEP1GSC048_3345 RLPKTKSWDWTLEASWQYGHNGQKINAGWDTLKQTVDGNPKSQRLYTQNVEYDSKFFIAQ

L_borg_LEP1GSC103_1279 RLPKTKSWDWTLEASWQYGHNGQKINAGWDTLKQTVDGNPKSQRIYTQNVEYDSKFFIAQ

L_alex_LEP1GSC062_3352 RLPKTKAWDWTLEASWQYGHNGQKINAGWDTLKQTVDGNPKSQRIYTQNVEYDSKFFIAQ

L_mayo_LEP1GSC190_3001 RLPKTKSWDWTLEASWQYGHNGQKINAGWDTLKQTVDGNPKSQRIYTQNVEYDSKFFIAQ

L_weil_LEP1GSC086_4105 RLPKTKSWDWTLEGSWQYGHNGQKINAGWDTLKQTVDGNPKSQRIYTQNVEYDSKFFIAQ

******:******.*****.***.:******:******:..*:*::*:.****:*:****

L_nogu_LEP1GSC059_1672 TGYTFFDRFRVGIQYSIGSGDPNRTDNKIATYDASFATRSGGFPYFDSGNGIANATFWSN

L_inte_LIC11268 TGYTFFDRFRVGIQYSIGSGDPNRTDNKVATYDASFATRSGGFPYFDSGNGIANATFWSN

L_kirs_LEP1GSC049_1107 TGYTFFDRFRVGIQYSIASGDPNRTDNKVATYDASFATRSGGFPYFDSGNGLANATFWSN

L_kmet_LEP1GSC052_1816 TGYTFFDRFRVGLQYSIGSGDPNRTDNKVATYDASFATRSGGFPYFDSGNGIVNATFWSN

L_alst_LEP1GSC193_4423 TGYTFFDRFRIGIQYSIGSGDPNRTDNKVATYDASFATRSGGFPYFDSGNGIVNATFWSN

L_sant_LEP1GSC048_3345 TGYTFFDRFRVGIQYSIGSGDPNRTDNKVATYDASFATRSGGFPYFDSGNGIVNATFWSN

L_borg_LEP1GSC103_1279 TGYTFFDRFRIGIQYSIGSGDPNRTDNKVATYDASFATRSGGFPYFDSGNGIVNATFWSN

L_alex_LEP1GSC062_3352 TGYTFFDRFRVGIQYSIGSGDPNRTDNKVATYDASFATRSGGFPYFDSGNGIVNATFWSN

L_mayo_LEP1GSC190_3001 TGYTFFDRFRIGIQYSIGSGDPNRTDNKVATYDASFATRSGGFPYFDSGNGIVNATFWSN

L_weil_LEP1GSC086_4105 TGYTFFDRFRVGIQYSIGSGDPNRTDNKVATYDASFATRSGGFPYFDSGNGIVNATFWSN

**********:*:****.**********:**********************:.*******

L_nogu_LEP1GSC059_1672 TRTKSIHLMYNSLNYGRFIFVVYDIQKASVNDAWYSSGGTANTGLTTENSTGAAFGGYKL

L_inte_LIC11268 TRTKSIHLMYNSPNYGRFIFVVYDIQKASVNDAWYSSGGAANTGLTTENPTGAAFGGYKL

L_kirs_LEP1GSC049_1107 TRTKSIHLMYNSRNYGRFIFVVYDIQKTSVNDAWYSSGGAANTGLTTENSTGAAFGGYKL

L_kmet_LEP1GSC052_1816 TRTKSIHLMYNSPNYGRFIFVAYDVQKASVNDAWYSSGGAANTGLTTENNTGAAFGGYKL

L_alst_LEP1GSC193_4423 TRTKSVHLMYNSANYGRFIFVAYDIQKASVNDAWYSSGGTANTGLTTENTTGSAFGGYKL

L_sant_LEP1GSC048_3345 TRTKSVHLMYNSANYGRFIFVAYDIQKASENDAWYSSGGTPNTGLTTENTTGSAFGGYKL

L_borg_LEP1GSC103_1279 TRTKSVHLMYNSANYGRFIFVAYDIQKASENDAWYSSGGTPNTGLTTENSTGSAFGGYKL

L_alex_LEP1GSC062_3352 TRTKSVHLMYNSANYGRFIFVAYDIQKASENDAWYSSGGTPNTGLTTENTTGSAFGGYKL

L_mayo_LEP1GSC190_3001 TRTKSVHLMYNSANYGRFIFVAYDIQKASENDAWYSSGGTPNTGLTTENTTGSAFGGYKL

L_weil_LEP1GSC086_4105 TRTKSVHLMYNSANYGRFIFVAYDIQKASVNDAWYSSGGTPNTGLTTENTTGSAFGGYKL

*****:****** ********.**:**:* *********:.******** **:*******

L_nogu_LEP1GSC059_1672 GERGGKRLFYELDLIYQFYLKDYVSIWTGGSYLLAGDAVRNARVNPWAPNINDRYTLDNR

L_inte_LIC11268 GEKGGKRLFYEFDLIYQFYLKDYVSIWTGGSYLLAGDAVRNARVNPWAPNINDRYTLDNR

L_kirs_LEP1GSC049_1107 GEKGGKRLFYEFDLIYQFYLKDYVSIWTGGSYLLAGDAVRNARVNPWAPNINDRYTLDNR

L_kmet_LEP1GSC052_1816 GERGGKRLFYEFDLIYQFYLKDYVSIWTGGSYLLAGDSVRNARVNPWAPNVNDRYTLDNK

L_alst_LEP1GSC193_4423 GERGGKRLFYEFDVIYQYYLKDYVSIWTGGSYLLAGDSVRNARVNPWAPNVNDRYTLDHK

L_sant_LEP1GSC048_3345 GERGGKRLFYEFDLIYQFYLKDYVSIWTGGSYLLAGDSVRNARVNPWAPNVNDRYTLDHK

L_borg_LEP1GSC103_1279 GERGGKRLFYEFDLIYQFYLKDYVSIWTGGSYLLAGDSVRNARVNPWAPNVNDRYTLDHK

L_alex_LEP1GSC062_3352 GERGGKRLFYEFDLIYQFYLKDYVSIWTGGSYLLAGDSVRNARVNPWAPNVNDRYTLDHK

L_mayo_LEP1GSC190_3001 GERGGKRLFYEFDLIYQFYLKDYVSIWTGGSYLLAGDSVRNARVNPWASNVNDRYTLDHK

L_weil_LEP1GSC086_4105 GERGGKRLFYEFDLIYQFYLKDYVSIWTGGSYLLAGDSVRNARVNPWAPNVNDRYTLDHK

**.********:*:***:*******************:**********.*:*******:.

L_nogu_LEP1GSC059_1672 SYSFFLFVQFAM

L_inte_LIC11268 AYSFFLFVQFAM

L_kirs_LEP1GSC049_1107 AYSFFLFVQFAM

L_kmet_LEP1GSC052_1816 SYSFFLFVQFAM

L_alst_LEP1GSC193_4423 SYSFFLFVQFAM

L_sant_LEP1GSC048_3345 SYSFFLFVQFAM

L_borg_LEP1GSC103_1279 SYSFFLFVQFAM

L_alex_LEP1GSC062_3352 SYSFFLFVQFAM

L_mayo_LEP1GSC190_3001 SYSFFLFVQFAM

L_weil_LEP1GSC086_4105 SYSFFLFVQFAM

:***********
