# Supplementary material for: Discovery of Novel Leptospirosis Vaccine Candidates Using Reverse and Structural Vaccinology
Source: Front Immunol. 2017 Apr 27;8:463. doi: 10.3389/fimmu.2017.00463 (PMC5406399; doi:10.3389/fimmu.2017.00463)
Supplement: Supplementary file 8 [file Data_Sheet_1.ZIP › Alignment Bb-OMPs/Mult_alignment_LIC11458_path_spp_orthol_immun_epit_highlighted.docx]

L_kmet_LEP1GSC052_0949 MHLRLILILFFMLLGLPIFAQ-TEEQMLRFITGGAGSSDQSPSSSPGDDKKLAILRAKNR

L_sant_LEP1GSC048_1482 MRLRLILILFFLLFGIPIFAQ-TEEQILRSIAGGTDQ-----TSSFGDDKKLSVLRAKNR

L_borg_LEP1GSC103_3744 ----MILILFFLLFWIPIFAQ-TEEQILRSITGGTET--ADQSSSFKDDKKLAVLRAKNR

L_mayo_LEP1GSC190_0221 MCLRLILILFFLLFGTPIFAQ-TEEQILRSITETSDQ-----NSSSRDDKKLAVLRAKNR

L_weil_LEP1GSC086_2774 MRLRLILILFFLLFGIPIFAQ-TEEQILRSITGETSD----QNSSFKDDKKLAVLRAKNR

L_alex_LEP1GSC062_1270 MRLRLILILFFLLFVTPIFAQ-TEEQILRSITGETAD----QNSSFKDDKKLAVLRAKNR

L_alst_LEP1GSC193_0379 MRLRLILILSFLLFGIPILAQ-TEEQMLRFITGGTGG---PSPSSSGDDKKLAILRAKNR

L_nogu_LEP1GSC059_3496 MPLRLILILSLLLIKIPIFAQSTDEQTLRLLTGGAGS------DSSNDDKKLSILRAKNR

L_inte_LIC11458 MPLRLVLILSLLLIKIPIYAQSTDEQTLRLLTGGAGS------DSSSDDKKLAILRAKNR

L_kirs_LEP1GSC049_0906 MPLRLILILSLLLIKIPIYAQSTDEQTLRLLTGAGSD------SSSGDDKKLAILRAKNR

::*** ::*: ** ** *:** ** :: .* *****::******

L_kmet_LEP1GSC052_0949 LLGKSIDTLPDREVDDLLLSLGLTRDGSLFSRRKRLRAALEEAVPVAADPFAQIPQQKKA

L_sant_LEP1GSC048_1482 LLGKSIDTLSDREVDDLLLSLGLTRDGSLFNRRKRLRAALEETIPVSTDPMSSIPQTKKA

L_borg_LEP1GSC103_3744 LLGKSIDTLSDREVDDLLLSLGLTRDGSLFNKRKRLRAALEETVPVSADPISSLPKTKKA

L_mayo_LEP1GSC190_0221 LLGKSIDTLSDREVDDLLLSLGLTRDGSLFNRRKRLRAALEETVPASTDPISSLPQSKKA

L_weil_LEP1GSC086_2774 LLGKSIDTLSDREVDDLLLSLGLTRDGSLFNRRKRLRAALEETVPVSSDPMSSLPQTKKA

L_alex_LEP1GSC062_1270 LLGKSIDTLSDREVDDLLLSLGLTRDGSLFNRRKRLRAALEETVPVSADPMSSLPQTKKA

L_alst_LEP1GSC193_0379 LLGKSIDSLPDREVDDLLLSLGLTRDGSLFIKRKRLRAALEESVPVAVDPMTQLPQTKKA

L_nogu_LEP1GSC059_3496 LLGKSIDTLPDREVDDLLLSLGLTRDGSLFTRRKRLRAALEEPLPVAVDPMLQLPQTKKA

L_inte_LIC11458 LLGKSIDTLPDREIDDLLLSLGLTRDGSLFTRRKRLRAALEEPLPTVVDPMVQLPQTKKA

L_kirs_LEP1GSC049_0906 LLGKSIDTLPDREVDDLLLSLGLTRDGSLFTRRKRLRAALEEPLPVAVDPMVQLPQTKKA

*******:*.***:**************** .**********.:*. **: .:*: ***

L_kmet_LEP1GSC052_0949 LPISIENASEGELLQVDKNKSGVLVLRGRVRLKLRSGSIEAETITVDSERQEIYAEGGIV

L_sant_LEP1GSC048_1482 LPISIENASEGELLQVDKNKSGVLVLRGRVRLKLRSGSLEAETITVDSERQEIYAEGGIV

L_borg_LEP1GSC103_3744 LPISIENASEGELLQVDKNKSGVLVLRGRVRLKLRSGSLEAETITVDSERQEIYAEGGIV

L_mayo_LEP1GSC190_0221 LPISIENASEGELLQVDKNKSGVLVLRGRVRLKLRSGSLEAETITVDSERQEIYAEGGIV

L_weil_LEP1GSC086_2774 LPISIENASEGELLQVDKNKSGVLVLRGRVRLKLRSGSLEAETITVDSERQEIYAEGGIV

L_alex_LEP1GSC062_1270 LPISIENASEGELLQVDKNKSGVLVLRGRVRLKLRSGSLEAETITVDSERQEIYAEGGIV

L_alst_LEP1GSC193_0379 LPISIENASEGELLQVDKNKSGVLVLRGRVRLKLRSGSLEAETITVDSERQEIYAEGGII

L_nogu_LEP1GSC059_3496 LPISIENASEGELLQVDKNKSGVLVLRGRVRLKLRSGSLEAETITVDSERQEIYAEGGIV

L_inte_LIC11458 LPISIENASEGELLQVDKNKSGVLVLRGRVRLKLRSGSLEAETITVDSERQEIYAEGGIV

L_kirs_LEP1GSC049_0906 LPISIENASEGELLQVDKNKSGVLVLRGRVRLKLRSGSLEAETITVDSERQEIYAEGGIV

**************************************:********************:

L_kmet_LEP1GSC052_0949 YKDGRAVVEGDKFIYDFRLEKGVVYKTKGTFAPAHFIGEKLKKLDDKRYALEMGYFTICN

L_sant_LEP1GSC048_1482 YKDGRAIVEGDKFIYDFRLEKGVVYKTKGTFAPAHFIGEKLKKLDDKHYALEMGYFTICN

L_borg_LEP1GSC103_3744 YKDGRAIIEGDKFIYDFRLEKGVVYKTKGTFAPAHFIGEKLKKLDDKHYALEMGYFTICN

L_mayo_LEP1GSC190_0221 YKDGRAIIEGDKFIYDFRLEKGVVYKTKGTFAPAHFIGEKLKKLDDKHYALEMGYFTICN

L_weil_LEP1GSC086_2774 YKDGRAIVEGDKFIYDFRLEKGVVYKTKGTFAPAHFIGEKLKKLDDKHYALEMGYFTICN

L_alex_LEP1GSC062_1270 YKDGRAIIEGDKFIYDFRLEKGVVYKTKGTFAPAHFIGEKLKKLDDKHYALEMGYFTICN

L_alst_LEP1GSC193_0379 YKDGRAVVEGDKFIYDFRLEKGVVYKTKGTFSPAHFIGEKLKKLDDKRYALEMGYFTICN

L_nogu_LEP1GSC059_3496 YKDGRAIVEGDKFIYDFRLEKGVVYKTKGTFSPAHFIGEKLKKLDDKRYALEMGYFTICN

L_inte_LIC11458 YKDGRAIVEGDKFIYDFRLEKGVVYKTKGTFSPAHFIGEKLKKLDDKRYALEMGYFTICN

L_kirs_LEP1GSC049_0906 YKDGRAIVEGDKFIYDFRLEKGVVYKTKGTFSPAHFIGEKLKKLDDKRYALEMGYFTICN

******::***********************:***************.************

L_kmet_LEP1GSC052_0949 AEKPHYSFKVNRLYIYDDKTVMATNVRYQVGGTTVLWLPFLYNSNLGNGWITQAGKNNTQ

L_sant_LEP1GSC048_1482 AEKPHYSFKVNRLYIYEDKTVMATNVRYQVGGTTVLWLPFLYNSNLGNGWIAQAGKNNTQ

L_borg_LEP1GSC103_3744 AEKPHYSFKVNRLYIYEDKTVMATNVRYQVGGTTVLWLPFLYNSNLGNGWIAQAGKNNTQ

L_mayo_LEP1GSC190_0221 AEKPHYSFKVNRLYIYEDKTVMATNVRYQVGGTTVLWLPFLYNSNLGNGWIAQAGKNNTQ

L_weil_LEP1GSC086_2774 AEKPHYSFKVNRLYIYEDKTVMATNVRYQVGGTTVLWLPFLYNSNLGNGWIAQAGKNNTQ

L_alex_LEP1GSC062_1270 AEKPHYSFKVNRLYIYEDKTVMATNVRYQVGGTTVLWLPFLYNSNLGNGWIAQAGKNNTQ

L_alst_LEP1GSC193_0379 AEKPHYSFKVNRLYIYEDKTVMATNVRYQVGGTTVLWLPFLYNSNLGNGWIAQAGKNNTQ

L_nogu_LEP1GSC059_3496 AEKPHYSFKVNKLYIYEDKTVMATNVRYQVGGTTVLWLPFLYNSNLGNGWIAQAGKNNTQ

L_inte_LIC11458 AEKPHYSFKVNKLYIYEDKTVMATNVRYQVGGTTVLWLPFLYNSNLGNGWIAQAGKNNTQ

L_kirs_LEP1GSC049_0906 AEKPHYSFKVNKLYIYEDKTVMATNVRYQVGGTTVLWLPFLYNSNLGNGWIAQAGKNNTQ

***********.****:**********************************:********

L_kmet_LEP1GSC052_0949 GLFMQNSYQWSVMPTFAIAPMGYKVRADFYEKTGQAFHLEMWKQSPFLNYLIDIGYANHK

L_sant_LEP1GSC048_1482 GLFLQTSYQWSKIPSFALAPMGYKFRADFYEKTGQAFHLEMWNQSPSLNYLIDVGYANHR

L_borg_LEP1GSC103_3744 GLFLQTSYQWSKIPSFAMAPMGYKFRADFYEKTGQAFHLEMWNQSPFLNYLIDIGYANHR

L_mayo_LEP1GSC190_0221 GLFLQTSYQWSKIPSFAMAPMGYKFRADFYEKTGQAFHLEMWNQSPFLNYLIDIGYANHR

L_weil_LEP1GSC086_2774 GLFLQTSYQWSNVPSFAIAPMGYKFRADFYEKTGQAFHLEMWNQSPFLNYLIDIGYANHR

L_alex_LEP1GSC062_1270 GLFLQTSYQWSKIPSFAMAPMGYKFRADFYEKTGQAFHLEMWNQSPFLNYLIDIGYANHR

L_alst_LEP1GSC193_0379 GMFLQSSYQWSAIPSFPLAPMGYKFRADFYEKTGQAFHMEMWNQSPALNYLIDIGYANHR

L_nogu_LEP1GSC059_3496 GLFLQTSYQWSTIPTFPLAPMGYKFRADFYEKTGQAFHVEMWNQSPALNYLIDIAYANHR

L_inte_LIC11458 GLFLQTSYQWSTIPTFPLAPMGYKFRADFYEKTGQAFHMEMWNQSPALNYLIDIAYANHK

L_kirs_LEP1GSC049_0906 GLFLQTSYQWSTIPTFPLAPMGYKFRADFYEKTGQAFHMEMWNQSPALNYLIDIAYANHR

*:*:*.***** :*:*.:******.*************:***:*** ******:.****.

L_kmet_LEP1GSC052_0949 NYQISGAYEDRFHNFGIGTSAVTNQVDRGAYYSADPNAPLRRIGEDTEPWWKGRIMLNSK

L_sant_LEP1GSC048_1482 NYQTTPAYEDRFHNFGIGTTAVTNQVDRGPLFSTNPNAPLRNIGPDTEPWWKGRILLNSK

L_borg_LEP1GSC103_3744 NYQTTTAYEDRFHNFGIGTVAVTNQVDRGALFSTNPNAPLRNIGPDTEPWWKGRILLNSK

L_mayo_LEP1GSC190_0221 NYQTTSAYEDRFHNFGIGTVAVTNQVDRGALFSTNPNAPLRNIGPDTEPWWKGRILLNSK

L_weil_LEP1GSC086_2774 NYQTTSAYEDRFHNFGIGTVAVTNQVDRGALFSTNPNAPLRNIGPDTEPWWKGRILLNSK

L_alex_LEP1GSC062_1270 NYQTTSAYEDRFHNFGIGTVAVTNQVDRGPLFSTNPNAPLRNIGPDTEPWWKGRILLNSK

L_alst_LEP1GSC193_0379 NYQTTTAYEDRFHNFGVGTTTVTNQVDRGALFSANPNAPLRNIGPDTEPWWKGRILVNSK

L_nogu_LEP1GSC059_3496 RYQTTTAYEDRFHNFGVGTTAVTNQVDRGALYSADPNAPLRNIGPDVEPWWKGRILLNSK

L_inte_LIC11458 RYQTTTAYEDRFHNFGVGTTAVTNQVDRGALYSADPNAPLRNIGPDVEPWWKGRILLNSK

L_kirs_LEP1GSC049_0906 RYQTTTAYEDRFHNFGVGTTAVTNQVDRGALYSADPNAPLRNIGPDVEPWWKGRILLNSK

.** : **********:** :********. :*::******.** *.********::***

L_kmet_LEP1GSC052_0949 FHNTEKDVTRNISLQYENYTNRLFEYEYGNRYEPANTLQSLYTARNVRLGFIRNTLEWKF

L_sant_LEP1GSC048_1482 MNNTEKDVTRNISLQYENYSDRLFEYEYGNRYEPANTLQSLYTARNVRMGFIRNSLEWKL

L_borg_LEP1GSC103_3744 MNNTEKDVTRNISFQYENYSDRLFEYEYGNRYEPANTLQSLYTARNVRMGFVRNSLEWKL

L_mayo_LEP1GSC190_0221 MNNTEKDVTRNISFQYENYSDRLFEYEYGNRYEPANTLQSLYTARNVRMGFVRNSLEWKL

L_weil_LEP1GSC086_2774 MNNTEKDVTRNISFQYENYSDRLFEYEYGNRYEPANTLQSLYTARNVRMGFVRNSLEWKL

L_alex_LEP1GSC062_1270 MNNTEKDVTRNISFQYENYSDRLFEYEYGNRYEPANTLQSLYTARNVRMGFVRNSLEWKL

L_alst_LEP1GSC193_0379 MNNTEKDVTRNISLQYENYTDRLFEYEYGNRYEPANTLQSLYTARNVRMGFVRNTLEWKL

L_nogu_LEP1GSC059_3496 MNNTEKDVTRNISLQYENYTDRLFDYEYGNRYEPANTLQSLYTARNVRMGFIRNTLEWKL

L_inte_LIC11458 TNNTEKDVTRNISLQYENYTDRLFDYEYGNRYEPANTLQSLYTARNVRMGFIRNTLEWKL

L_kirs_LEP1GSC049_0906 TNNTEKDVTRNISLQYENYTDRLFDYEYGNRYEPANTLQSLYTARNVRMGFIRNTLEWKL

:***********:*****::***:***********************:**:**:****:

L_kmet_LEP1GSC052_0949 DYTENRGDLSINVGMKRNMLYYILNPTGKSGYFPTVDVLPTTTIRNSSEITRLPYFNAPV

L_sant_LEP1GSC048_1482 DYTENRGDLSINVGMKRNLIYYILNPADKSGYFPTVDTIPTTTIRNSSEIGRIPYFNSAV

L_borg_LEP1GSC103_3744 DYTENRGDLSINVGMKRNLIYYILNPADKSGYFPTVDTIPTTTIRNSSEIGRIPYFNSAV

L_mayo_LEP1GSC190_0221 DYTENRGDLSINVGMKRNLLYYILNPADKSGYFPTVDTIPTTTIRNSSEIGRIPYFNSAV

L_weil_LEP1GSC086_2774 DYTENRGDLSINVGMKRNLIYYILNPADKSGYFPTVDTIPTTTIRNSSEIGRIPYFNSAV

L_alex_LEP1GSC062_1270 DYTENRGDLSINVGMKRNLIYYILNPADKSGYFPTVDTIPTTTIRNSSEIGRIPYFNSAV

L_alst_LEP1GSC193_0379 DYTENRGDLSINVGMKRNLLYYILNPADKSGYFPTVDTVPTTTIRNSSEIGRIPYFNSSV

L_nogu_LEP1GSC059_3496 DYTENRGDLSINIGMKRNLLYYILNPADKSGYFPTVDTIPTTTIRNSSEIGRIPYFNSAV

L_inte_LIC11458 DYTENRGDLSINIGMKRNLLYYILNPADKSGYFPTVDTIPTTTIRNSSEIGRIPYFNSAV

L_kirs_LEP1GSC049_0906 DYTENRGDLSINIGMKRNLLYYILNPADKSGYFPTVDTIPTTTIRNSSEIGRIPYFNSAV

************:*****::******:.*********.:*********** *:****:.*

L_kmet_LEP1GSC052_0949 YWDVYLTNMILRYYGVPTRENLNIPTLDGSYQDPWGNYKENVLRTQYFTQGESGLRTTLN

L_sant_LEP1GSC048_1482 YWDVFLNNTILRYYGTPIRENLRIPTLEGSFQDPWGGYRENVFRTQYFTQGESGLRTTLN

L_borg_LEP1GSC103_3744 YWDVFLNNTILRYYGVPIRENLRIPTLEGSFQDPWGGYRENIFRTQYFTQGESGLRTTLN

L_mayo_LEP1GSC190_0221 YWDVFLNNTILRYYGVPIRENLRIPTLEGSFQDPWGGYRENVFRTQYFTQGESGLRTTLN

L_weil_LEP1GSC086_2774 YWDVFLNNTILRYYGVPTRENLRIPTLEGSFQDPWGSYKENVFRTQYFTQGESGLRTTLN

L_alex_LEP1GSC062_1270 YWDVFLNNTILRYYGVPIRENLRIPTLEGSFQDPWGSYKENVFRTQYFTQGESGLRTTLN

L_alst_LEP1GSC193_0379 YWDVFLNNVILRYYGVPTRENLKIPTLEGSFQDPWGSYKENVFRTQYFTQGESGLRTTLN

L_nogu_LEP1GSC059_3496 YWDVFLNNTILRYYGAPVRENLKIPTLDGSFQDPWGNYKENVFRTQYFTQGESGLRTTLN

L_inte_LIC11458 YWDVFLNNTILRYYGAPIRENLKIPTLDGSFQDPWGDYKENVFRTQYFTQGESGLRTTLN

L_kirs_LEP1GSC049_0906 YWDVFLNNTILRYYGAPIRENLKIPTLDGSFQDPWGSYKENVFRTQYFTQGESGLRTTLN

****:*.* ******.* ****.****:**:*****.*.**::*****************

L_kmet_LEP1GSC052_0949 FGSYLTFTPNAFFGAKKQSATVRNNAAVTGVTDNAFTSLERFLARESYEYVRTSSNLRFG

L_sant_LEP1GSC048_1482 FGSYLTFTPNAFLGAKKQSATVRNNTAVTGVTDSSFTSLERYLARESYEYLRTSSNLRFG

L_borg_LEP1GSC103_3744 FGSYLTFTPNAFFGAKKQSATVRNNTAVTGVTDSSFTSLERYLARESYEYLRTSSNLRFG

L_mayo_LEP1GSC190_0221 FGSYLTFTPNAFFGAKKQSATVRNNTAVTGVTDSSFTSLERYLARESYEYLRTSSNLRFG

L_weil_LEP1GSC086_2774 FGSYLTFTPNAFFGARKQSATVRNNTAVTGVTDSSFTSLERYLARESYEYLRTSSNLRFG

L_alex_LEP1GSC062_1270 FGSYLTFTPNAFFGAKKQSATVRNNTAVTGVTDSSFTSLERYLARESYEYLRTSSNLRFG

L_alst_LEP1GSC193_0379 FGSYLTFTPNAFFGAKKQSATVRNNSAVTGVTDNSFTSLERFLARESYEYLRTSSNLRFG

L_nogu_LEP1GSC059_3496 FGSYLTFTPNAFFGAKKQSANVRNNTAVTGVTDNAFTSLERYLARESYEYLRTSSNLRFG

L_inte_LIC11458 FGSYLAFTPNAFFGAKKQSANVRNNTAVTGVTDNAFTSLERYLARESYEYLRTSSNLRFG

L_kirs_LEP1GSC049_0906 FGSYLAFTPNAFFGAKKQSANVRNNTAVTGVTDNAFTSLERYLARESYEYLRTSSNLRFG

*****:******:**.****.****:*******.:******:********:*********

L_kmet_LEP1GSC052_0949 VPLLFFNATYRKLEAYKPELQDPILAKTRQHELELSLESYALENFEISVRTIRDLRNFSP

L_sant_LEP1GSC048_1482 IPLLFFNTTYRKSEAYKPELQDPILARTRQHELELSLESYALENFEISIRTIRDLRSFSP

L_borg_LEP1GSC103_3744 IPLLFFNTTYRKSEAYKPELQDPILARTRQHELELSLESYALENFEISVRTIRDLRNFSS

L_mayo_LEP1GSC190_0221 IPLLFFNTTYRKSEAYKPELQDPILSRTRQHELELSLESYALENFEISIRTIRDLRNFSS

L_weil_LEP1GSC086_2774 IPLLFFNTTYRKLEAYKPELQDPILARTRQHELELSLESYALENFEISVRTIRDLRNFSS

L_alex_LEP1GSC062_1270 IPLLFFNTTYRKSEAYKPELQDPIFARTRQHELELSLESYALENFEISIRTIRDLRNFSS

L_alst_LEP1GSC193_0379 IPLLFFNTTYRKMEVYKPELQDPVLAKTRQHELEVSLESYALENFEMSIRTIRDLRTFSS

L_nogu_LEP1GSC059_3496 VPLLFFNTTYRKTEIYKPELQDPILGKTRQHELELSLESYALENFEISIRTIRDLRTFSD

L_inte_LIC11458 IPLLFFNTTYRKTEIYKPELQDPILGKTRQHELELSLESYALENFEISIRTIRDLRSFSD

L_kirs_LEP1GSC049_0906 IPLLFFNTTYRKTEIYKPELQDPILGKTRQHELELSLESYALENFEISIRTIRDLRTFSD

:******:**** * ********::..*******:***********:*:*******.**

L_kmet_LEP1GSC052_0949 EYKPQPTDAERWYFTVARFSGYFDFLDGFRPKRASLLEKKRSFYSGLFINNDYVHHNPKA

L_sant_LEP1GSC048_1482 AYKPQPTDSERWYFTIARFSGYFDFLDGFRPKRVSLLEKKRSFYSGLFLNNDYVHHTPKA

L_borg_LEP1GSC103_3744 DYKPQPTDSERWYFTIARFSGYFDFLDGFRPKRVSLLEKKRSFYSGLFLNNDYVHHTPKA

L_mayo_LEP1GSC190_0221 DYKPQPTDSERWYFTIARFSGYFDFLDGFRPKRVSLLEKKRSFYSGLFLNNDYVHHTPKA

L_weil_LEP1GSC086_2774 DYKPQPTDSERWYFTIARFSGYFDFLDGFRPKRVSLLEKKRSFYSGLFLNNDYVHHTPKA

L_alex_LEP1GSC062_1270 DYKPQPTDSERWYFTIARFSGYFDFLDGFRPKRVSLLEKKRSFYSGLFLNNDYVHHTPKA

L_alst_LEP1GSC193_0379 DYKPQPTDSERWYFTIARFSGYFDFLDGFSPKRVSLLEKKRSFYSGLFLSNDYVHHTPKA

L_nogu_LEP1GSC059_3496 EYKPQPTNAERWYFTVARFSGYFDFLDGLRPKRVSLLERKRSFYSGLFLNNDYVHHNPKA

L_inte_LIC11458 EYKPQPTDAERWYFTVARFSGYFDFLDGLRPKRVSLLERKRSFYSGLFLNNDYVHHNPKA

L_kirs_LEP1GSC049_0906 EYKPQPTDAERWYFTVARFSGYFDFLDGLRPKRVSLLERKRSFYSGLFLNNDYVHHNPKA

******::******:************: ***.****.*********:.******.***

L_kmet_LEP1GSC052_0949 KPLSNSFTASYKMGGFTLPFIRLIRELELGGTWYHVYNSPILDGYRVFVKANVDFTRHLG

L_sant_LEP1GSC048_1482 KPLYNSFTASYKMGGFTLPLIRLVRELELGGTWYHVYNSPILDGYRVFVKANVDFTRYLG

L_borg_LEP1GSC103_3744 KPLYNSLTASYKMGGFTLPLVRLIRELELGGTWYHVYNSPILDGYRVFVKANVDFTRYLG

L_mayo_LEP1GSC190_0221 KPLYNSLTASYKMGGFTLPLVRLVRELELGGTWYHVYNSPILDGYRVFVKANVDFTRYLG

L_weil_LEP1GSC086_2774 KPLYNSLTASYKMGGFTLPLVRLIRELELGGTWYHVYNSPILDGYRVFVKANVDFTRYLG

L_alex_LEP1GSC062_1270 KPLYNSLTASYKMGGFTLPLVRLIRELELGGTWYHVYNSPILDGYRVFVKANVDFTRYLG

L_alst_LEP1GSC193_0379 KPLYNSLTASYKMGGFTLPLIRLIRELELGGTWYHVYNSPILDGYRVFVKANVDFTRFLG

L_nogu_LEP1GSC059_3496 KPLYNSLTASYKMGGFTLPLVRLIRELELGGTWYHVYNSPILDGYRVFVKANVDFTRYLG

L_inte_LIC11458 KPLYNSLTASYKMGGFTLPFVRLIRELELGGTWYHVYNSPILDGYRVFVKANVDFTRYLG

L_kirs_LEP1GSC049_0906 KPLYNSLTASYKMGGFTLPLVRLIRELELGGTWYHVYNSPILDGYRVFVKANVDFTRYLG

*** **:************::**:*********************************.**

L_kmet_LEP1GSC052_0949 IEAELDSRVSQPWRYTNQVGNTYDTFYYGNDPTTSVANLNLDRTTLQRDLVDGTGVNGNG

L_sant_LEP1GSC048_1482 IEAELDSRVSQPWRYTNQVGNSYDTFYYGNDPTASVASINLERTTLQKDLLDGTGVNGNG

L_borg_LEP1GSC103_3744 IEAELDSRVSQPWRYTNQVGNTYDTFYSGNDPTASVSSINLERTTLQKDLLDGTGVNGNR

L_mayo_LEP1GSC190_0221 IEAELDSRVSQPWRYTNQVGNAYDTFYYGNDPTASVASINLERTTLQKDLLDGTGVNGNG

L_weil_LEP1GSC086_2774 IEAELDSRVSQPWRYTNQVGNTYDTFYYGNDPTASVASINLERTTFQKDLLDGTGVNGNG

L_alex_LEP1GSC062_1270 IEAELDSRVSQPWRYTNQVGNTYDTFYYGNDPTASVSSINLERTTLQKDLLDGTGVNGNG

L_alst_LEP1GSC193_0379 IEAELDSRVSQPWRYTNQVGNTYDTFYSGNDPTTSVSTLNLERTSLQRDLLDGTGVNGNG

L_nogu_LEP1GSC059_3496 IEAELDSRVSQPWRYTNQVGNTYDTFYYGNDPTMSVATINLDRTSLQKDLLDGTGVNGNG

L_inte_LIC11458 IEAELDSRVSQPWRYTNQVGNTYDTFYYGNDPTMSVATINLDRTSLQKDLLDGTGVNGNG

L_kirs_LEP1GSC049_0906 IEAELDSRVSQPWRYTNQVGNTYDTFYYGNDPTMSVATINLDRTSLQKDLLDGTGVNGNG

*********************:***** ***** **:.:**:**::*.**:********

L_kmet_LEP1GSC052_0949 ARQNTALNINRFMGTIKYNLHTANFRLGYSMDLRAVPGGRTDGLVSFYDQSVFFSISISD

L_sant_LEP1GSC048_1482 ARQNTALNINRFMGTIKYNLHTANFRLGYSMDLRSVPGGRTDGLVSFYDQSVFFSISITD

L_borg_LEP1GSC103_3744 AGQNTALNINRFMGTIKYNLHTANFRLGYSMDLRSVPGGRTDGLVSFYDQSVFFSISITD

L_mayo_LEP1GSC190_0221 ARQNTALNINRFMGTIKYNLHTANFRLGYSMDLRSVPGGRTDGLVSFYDQSVFFSISITD

L_weil_LEP1GSC086_2774 ARQNTALNINRFMGTIKYNLHTANFRLGYSMDLRSVPGGRTDGLVSFYDQSVFFSISITD

L_alex_LEP1GSC062_1270 ARQNTALNINRFMGTIKYNLHTANFRLGYSMDLRSVPGGRTDGLVSFYDQSVFFSISITD

L_alst_LEP1GSC193_0379 AKQNTALNINRFMGTIKYNLHTANFRLGYSMDLRAVPGGRTDGLVSFYDQSIFFSISITD

L_nogu_LEP1GSC059_3496 ARQNTALNINRFMGTIKYNLHTANFRLGYSMDLRSVPGGRTDGLVSFYDQSVFFSISITD

L_inte_LIC11458 SRQNTALNINRFMGTIKYNLHTANFRLGYSMDLRSVPGGRTDGLVSFYDQSVFFSISITD

L_kirs_LEP1GSC049_0906 ARQNTALNINRFMGTIKYNLHTANFRLGYSMDLRSVPGGRTDGLVSFYDQSVFFSISITD

: ********************************:****************:******:*

L_kmet_LEP1GSC052_0949 FTLGQQDSSELTRVRLFRFRKRPFQTGDRAGISSENP

L_sant_LEP1GSC048_1482 FTLGQQDSSELTRVRLFRFRKRPFQAGDSAGISSENP

L_borg_LEP1GSC103_3744 FTLGQQDSSELTRVRLFRFRKRPFQAGDSAGISSENP

L_mayo_LEP1GSC190_0221 FTLGQQDSSELTRVRLFRFRKRPFQAGDSAGISSENL

L_weil_LEP1GSC086_2774 FTLGQQDSSELTRVRLFRFRKRPFQAGDSAGISSENL

L_alex_LEP1GSC062_1270 FTLGQQDSSELTRVRLFRFRKRPFQAGDSAGISSENL

L_alst_LEP1GSC193_0379 FTLGQQDSSELTRVRLFRFRKRPFQAGDATGITSENP

L_nogu_LEP1GSC059_3496 FTLGQQDSSELTRVRLFRFRKRPFQAGDSTGISSENP

L_inte_LIC11458 FTLGQQDSSELTRVRLFRFRKRPFQAGDSTGISSENP

L_kirs_LEP1GSC049_0906 FTLGQQDSSELTRVRLFRFRKRPFQAGDSTGISSENP

*************************:** :**:***
