# Supplementary material for: Discovery of Novel Leptospirosis Vaccine Candidates Using Reverse and Structural Vaccinology
Source: Front Immunol. 2017 Apr 27;8:463. doi: 10.3389/fimmu.2017.00463 (PMC5406399; doi:10.3389/fimmu.2017.00463)
Supplement: Supplementary file 8 [file Data_Sheet_1.ZIP › Alignment Bb-OMPs/Mult_alignment_LIC11506_path_spp_orthol_immun_epit_highlighted.docx]

L_alex_LEP1GSC062_3772 ------------------------------------------------------------

L_weil_LEP1GSC086_0146 ------------------------------------------------------------

L_alst_LEP1GSC193_2649 MSKPKRIIYQSFFSARMEIQKGAFKLSERGRAPIQTQQMISKILKKSLILDFSRNRIATF

L_borg_LEP1GSC103_3352 ------------------------------------------------------------

L_mayo_LEP1GSC190_0024 ------------------------------------------------------------

L_inte_LIC11506 ------------------------------------------------------------

L_kirs_LEP1GSC049_0855 ------------------------------------------------------------

L_nogu_LEP1GSC059_3436 ------------------------------------------------------------

L_kmet_LEP1GSC052_0587 ------------------------------------------------------------

L_sant_LEP1GSC048_1139 ------------------------------------------------------------

L_alex_LEP1GSC062_3772 ----------MRKNLLCFLFQKRYFG-IWIFLCIYSLQTDPTFSQQKEKNQEGGKKKSAF

L_weil_LEP1GSC086_0146 ----------MRENLLCFLFRKRYFG-IWIFLCIYFLQTDPTFSQQKEKNQEGEKKKSVF

L_alst_LEP1GSC193_2649 LEGIKQRGKKMRENLFCFLFRNRYLG-IWIFLCIYSLQAHPLFSQKQEENREGEKKKSVF

L_borg_LEP1GSC103_3352 ----------MRENLIYFLFRKRYFG-IWIFLYIYSLQADPTYSQQKEENQEGGKKKSFF

L_mayo_LEP1GSC190_0024 ----------MRENLLYFLFLKRYFG-IWIFLCIYSLQANPIFSQEKEKNQEGVKKKSFF

L_inte_LIC11506 ----------MLKKTSYFSIWQKGFK-VSIFLIFYILFPSSVLSQAEEN------KKNTF

L_kirs_LEP1GSC049_0855 ----------MLKKNSYFWIRKKRFKMIYIFLIFYIFVSNSVLSQTEDN------KKNTF

L_nogu_LEP1GSC059_3436 ----------MFKKVLCFSIWKKRFK-IYTFLIFYIFFLNSVLSQTQED------KKNTF

L_kmet_LEP1GSC052_0587 ----------MQTRYLRFPKWKRILE-VAILLCLFGVVLKSVNAETEEEGSDKNAKKNSF

L_sant_LEP1GSC048_1139 ----------MTKKTSYFSARKKGFK-ICIFLFVCVSLWKPVLSQTAEN------KKSAF

* . * :. : : :* . . :: :. **. *

L_alex_LEP1GSC062_3772 EFWIKRQTYRWTPYDYTSFSEHSSLESSNKTDSVKQNQKVLIPLAFRYDRHDKKYRIELS

L_weil_LEP1GSC086_0146 EFWIKRQTYRWTPYDYTSFSEHSPLESSNKTDSVKQNQKVLVPLAFRYDRHDKKYRIELS

L_alst_LEP1GSC193_2649 EFWIKRQTYRWTPYDYTSFSEHSPLESSNKTDSVKQNRKVLVPLAFRYDNLEKKFRIEIS

L_borg_LEP1GSC103_3352 ELRVKRQTYQWTPYDYTSFSEHSSLESSNKTDSVKQNQKVLVPLAFRYDNLEKEFRIEIS

L_mayo_LEP1GSC190_0024 EFWIKRQTYRWTPYDYTSFSEHSSLESSNKTDSIKQNQKVLVPLVFRYDNLEKKFRIEIS

L_inte_LIC11506 ELLIKRQTYRWTPYDYTSYTERSILETTIKTDSIKQNQKVLIPLVFRYDQLEKKFRIEVS

L_kirs_LEP1GSC049_0855 ELLIKRQTYQWTPYDYTSYTERSILETTVKTDSIKQNQKVLIPLVFRYDQLEKKFRIEIS

L_nogu_LEP1GSC059_3436 ELLIKRQTYRWTPYDYTSYTERSILETTIKTDSIKENQKVLIPLVFRYDQLEKKFRIEIS

L_kmet_LEP1GSC052_0587 ELILKRQTYQWTPYDYTSYTERSPLETSIKTDSVKQNQKVLTPFVFRFDQKERKFRVEIS

L_sant_LEP1GSC048_1139 ELLIKRQTYQWTPYDYTSYTERSISEISTKTDSVKQNQKVLIPFAFRYDQLERKFRIEIS

*: :*****.********::*.* * : ****:*:*.*** *:.**:*. :.::*:*:*

L_alex_LEP1GSC062_3772 AYEIELANPNTIVTKAGDGGIAIQRHYFNPMIRSEAEFNYYKILNLSEDWDLFAGAGIRN

L_weil_LEP1GSC086_0146 AYEIELANPNAIVTKAGGGGIEIQRHYFNPMIRSEAEFNYYKILNLSENWDLFAGVGIRN

L_alst_LEP1GSC193_2649 AYEIELANANTNFTRSGD---QVRRHYFNPMLRSEAEFNFYKILIWNEDWKVFAGAGIRN

L_borg_LEP1GSC103_3352 VYEIELANANTNFTQAGD---QVRRHYFNPMLRSEAEFNFYKILNWDEDWKVFAGAGIRN

L_mayo_LEP1GSC190_0024 AYEIELANANTNFTRAGD---RVRRYYFNPMLRSEAEFNFYKILNWSEDWKVFAGAGIRN

L_inte_LIC11506 AYEIELANPNTNVIESGTLGYETRKQYFNPMLRSEAELNYYKILNLYPNWDLFVGAGIRN

L_kirs_LEP1GSC049_0855 AYEVELANPNTNVVQSGSLGYETRRQYFNPMLRSEAEFNYYKILNLYPNWDFFAGAGIRN

L_nogu_LEP1GSC059_3436 AYEIELVNPNTNVVQFGSLGFDARRQYFNPMLRSEAEFNYYKIINLHPNWDFFTGAGIRN

L_kmet_LEP1GSC052_0587 AYEIELANPNMIVTRTGSSGFEIGRQYLNPMIRSEAELNLFKIFDLHEDWRIFAGAGIRN

L_sant_LEP1GSC048_1139 VYEIELANANTIVTRADSGGYSTKRQYFNPMLRSEAELNLYKIFDRIEDWKFFLGLGIRN

.**:**.*.* . . . *:***:*****:* :**: :* .* * ****

L_alex_LEP1GSC062_3772 INKYKYGYFLQEGAYQEYFYTYGPQFVFRTDYRFMENFSLSFAADLFYTEGNRFYKPKTV

L_weil_LEP1GSC086_0146 INKYKYGYFLQEGAYQEYFYTYGPQFVFRTDYRFMENFSLSFAADLFYTEGNRFYKPKTV

L_alst_LEP1GSC193_2649 INKYKYGYFLKEGSYQEYFYTYGPQIVLNTEYKLWKEISVHLGLDLFYTEGNRFYKDRTI

L_borg_LEP1GSC103_3352 INKYKYGYFLKEGSYQEYFYTYGPQIVLNTEYKLWKEISIHLGLDFFYTEGNRFYKDRMI

L_mayo_LEP1GSC190_0024 INKYKYGYFLKEGSYQEYFYTYGPQIVLNTECKLWKEISVHLSLDLFYTEGNRFYKDRMI

L_inte_LIC11506 INKYKYGYFLREGAYQEYFYTYGPQFVFRTEYRFAENWSFGLAADLFYTQGTRFYKPQAL

L_kirs_LEP1GSC049_0855 INKYKYGYFLREGVYEEYFYTYGPQIVFRTDFRPIENFSFSLAADFFYTEGSRFYKQKTL

L_nogu_LEP1GSC059_3436 INKYKYGYFLREGVYEEYFYTYGPQIVFRTDFRPIENLYFSLAADFFYTEGNRFYKQKTL

L_kmet_LEP1GSC052_0587 INKYKYGYYLREGAYEEYFYTYGPQLVFRTDYRIWEKFFLSLGADLFYTEGNRFYKPKTF

L_sant_LEP1GSC048_1139 INKYRYGYFLREGAYEEFFYTYGPQIVFRTDYRIWERFFLGFAVDLFYTEGNRFYKSKTM

****.***:*.** *:*:*******:*:.*: . : . :. *:***:*.**** . .

L_alex_LEP1GSC062_3772 SLDFVTISSGTAGVRGIYRGYELDLSFGYKIFETMKLYVGYGYIYSYFSYLGFNQTDFAL

L_weil_LEP1GSC086_0146 SLDSVTISNGTVGVRGIYRGYELDLSLGYKIFETVKLYVGYNYIYSYFSYLGFNQTDFVL

L_alst_LEP1GSC193_2649 TPDAIVVSNGNAGVKGIYRGYEVDLSLSYKIFDTVKLYVGYNYIYSYFSYYGFRQTDFNL

L_borg_LEP1GSC103_3352 MPDSIVVSNGNAGVKGIYRGYEIDFSLSYKIFETVKFYAGYNYIYSYFSYLGFNQIDFTL

L_mayo_LEP1GSC190_0024 TPDLIVVSNGNAGVKGIYRGYEIDFSLSYKIFETVKLYAGYNYIYSYFSYLGFNQIDFTL

L_inte_LIC11506 ALESITVSAGTAGAQGIYRGYELDVSLGYRVFDSVKFFVGYNYIYSYFSYYSFHQTDIRL

L_kirs_LEP1GSC049_0855 TPDWIVVPIGSAGAKGIYRGYEIDFSISYRIFEKFKIYLGYNYIYSFFSYYGFQQTNINF

L_nogu_LEP1GSC059_3436 TPDWMVVSFGGAGAKGIYRGYEIDFSISYRIFEKFKIYLGYNYIYSFFSYYGFQQTNLNF

L_kmet_LEP1GSC052_0587 TLDSVSLSSGTAGVRGIYRGYEIDFSFAYQISQSLKFYLGYSYIDSYFSYYGFNQTDLRF

L_sant_LEP1GSC048_1139 TLESISISTGNSGVRGIYRGSEIDFSFVYQFSEAFKFYVGYSYIYSYFSYYGFNQTDLNL

: : :. * *..***** *:*.*: *.. : .*:: **.** *:*** .*.* :: :

L_alex_LEP1GSC062_3772 GNSQISPIQMHTNDVP------FMTRPIRSGNHDILQGFYFGVAVGF

L_weil_LEP1GSC086_0146 GNSQVSPIQMHTNDVP------FMTRPIRSGNHDILQGFYFGVSVGF

L_alst_LEP1GSC193_2649 GNPQISPFQTQVDNSP------SLSHSIRSGNHDILQGLYLGIGVNF

L_borg_LEP1GSC103_3352 GNVQISPIQTQTNNVP------FMIRPIRSGNHDILQGFYFGAAVGF

L_mayo_LEP1GSC190_0024 GNSQNSPIQTQANDVP------FMVRPIRSGNHDILHGFYFGVAVGF

L_inte_LIC11506 QTSSLDPFISENSSAP-------ILHMFRSGNHDILQGLYLGMSVGF

L_kirs_LEP1GSC049_0855 QTNSNDPFVKERTPP-------ITTHSSRSGNHDILQGVYLGISVCF

L_nogu_LEP1GSC059_3436 QTNSNDPFTKEQTPA--------ITHPSRSGNHDILQGVYLGISVCF

L_kmet_LEP1GSC052_0587 GNASTDPFQIGTSNTQPNYNTFQISHPILSGHRDLLRGVYLGLSVQF

L_sant_LEP1GSC048_1139 GTVPSNPFFNTAAFT-------MLSHPIKSGDHDILRGFYFGISVCF

. .*: . ** .*:*.*.*:* .* *
