# Supplementary material for: Discovery of Novel Leptospirosis Vaccine Candidates Using Reverse and Structural Vaccinology
Source: Front Immunol. 2017 Apr 27;8:463. doi: 10.3389/fimmu.2017.00463 (PMC5406399; doi:10.3389/fimmu.2017.00463)
Supplement: Supplementary file 8 [file Data_Sheet_1.ZIP › Alignment Bb-OMPs/Mult_alignment_LIC11623_path_spp_orthol_immun_epit_highlighted.docx]

L_inte_LIC11623 MIHCKYPGGFSFTLK-RIFSPILKGILLAILVGLLFYSGELTQILSKRSDFLGKTIKEVK

L_kirs_LEP1GSC049_2475 -------MVLDFTLK-RIFSPILKGTLFAILVGLLFYSGELTQILSKRSDFLGKTIKEVK

L_nogu_LEP1GSC059_0588 MIHCKYPGGFSFTLKRRIFSPILKGTLFAVLVGLLFYSGELTQILSKRNDFLGKTIKEVK

L_kmet_LEP1GSC052_1064 -------MALDFTLK-RIFSLTLKGSVIAILLGLLFNSGELTQLLSKRSDFLGKVIKEVK

L_alst_LEP1GSC193_1910 -------MALVFTLK-RTFSPILKGSVFAILLGLLFYSGELTQILSKRNDFFGKTIKEIK

L_sant_LEP1GSC048_3025 --------------------------MFAVLLGLLFYSGELTQILSKRNDFLGKVIKEVK

L_mayo_LEP1GSC190_2598 ----------------------------MILLGLLFYSGELTQIFSKRNDFLGKVIKEIK

L_borg_LEP1GSC103_1744 ----------------------------MILLGLLFYSGELTQIFSKRNDFLGKVIKEVK

L_alex_LEP1GSC062_2892 ----------------------------MILLGLLFYSGELTQILSKRNDFLGKVIKEVK

L_weil_LEP1GSC086_3808 ----------------------------MILLGLLFYSGELTQILSKRNDFLGKVIKEVK

:*:**** ******::***.**:**.***:*

L_inte_LIC11623 FKGNKNTPDADLESMIEIKVGKILTKRILDRDLKNLFNSGFFYFVDIQAEDFQDGVRIIF

L_kirs_LEP1GSC049_2475 FKGNKNTPDADLESMIEIKVGKILTKRILDRDLKNLFNSGFFYFVDIQAEDFQDGVRIIF

L_nogu_LEP1GSC059_0588 FKGNKNTPDADLESMIEIKVGKILTKRILDRDLKNLFNSGFFYFVDIQAEDFQDGIRIIF

L_kmet_LEP1GSC052_1064 FKGNKNTPDGDLESMIEMKVGKTLTKKILDRDLKTLFNSGFFYFVDIQAEDLQDGVRIIV

L_alst_LEP1GSC193_1910 FKGNKNTPDGDLESMIEMKVGKTLTKKILDRDLKNLFNSGFFYFVDIQAEDLQDGVKIIF

L_sant_LEP1GSC048_3025 FKGNKNTPDSDLESMIEIKVGKTLTKKILDRDLKNLFNSGFFYFVDIQAEEVSDGIRIIF

L_mayo_LEP1GSC190_2598 FKGNKNTPDSDLESMIEIKIGKILTKKILDRDLKNLFNSGFFYFVDIQAEEVSDGIRIIF

L_borg_LEP1GSC103_1744 FKGNKNTPDSDLESMIEIKIGKTLTKKILDRDLKNLFNSGFFYFVDIQAEEVSDGIRIIF

L_alex_LEP1GSC062_2892 FKGNKNTPDSDLESMIEIKIGKILTKKILDRDLKNLFNSGFFYFVDIQAEEVSDGIRIIF

L_weil_LEP1GSC086_3808 FKGNKNTPDSDLESMIEIKIGKTLTKKILDRDLKNLFNSGFFYFVDIQAEEVSDGIRIIF

*********.*******:*:** ***.*******.***************:..**:.**.

L_inte_LIC11623 DLKERPIVREIEFVGADEVFPADLRDKLPLKDNEVITPQKITKSRDLILQKYRDEGFFLA

L_kirs_LEP1GSC049_2475 DLKERPRVREIEFVGADEVFPADLRDKLPLKDNEVITPQKITKSRDLILQKYRDEGFFLA

L_nogu_LEP1GSC059_0588 DLKERPRVREIEFVGADEVFPADLRDKLPLKDNEVITPQKITKSRDLILQKYRDEGFFLA

L_kmet_LEP1GSC052_1064 DLKERPRVRDVEFVGADEVFPADLRDKLPLKDNEVITPQKITKSRDLILQKYRDEGFFLA

L_alst_LEP1GSC193_1910 DLKERPRVKELEFVGADEVFPADLRDKLPLKDNEVITPQKITKSRDLILQKYRDEGFFLA

L_sant_LEP1GSC048_3025 DLKERPRVKEIEFVGADEVFPADLRDKLPLKDNEVITPQKITKSRDLILQKYRDEGFFLA

L_mayo_LEP1GSC190_2598 DLKERPRVKEIEFVGADEVFPADLRDKLPLKDNEVITPQKITKSRDLILQKYRDEGFFLA

L_borg_LEP1GSC103_1744 DLKERPRVKEIEFVGADEVFPADLRDKLPLKDNEVITPQKITKSRDLILQKYRDEGFFLA

L_alex_LEP1GSC062_2892 DLKERPRVKEIEFVGADEVFPADLRDKLPLKDNEVITPQKITKSRDLILQKYRDEGFFLA

L_weil_LEP1GSC086_3808 DLKERPRVKEIEFVGADEVFPADLRDKLPLKDNEVITPQKIAKSRDLILQKYRDEGFFLA

****** *.::******************************:******************

L_inte_LIC11623 YVKVELGKPDPKTNLVRVRFIIDEGEEIPVSKINVYGNESIETSEILSIMEMKEEGVFEG

L_kirs_LEP1GSC049_2475 YVKVELGKPDPKTNLVRVRFIIDEGEEIPVSKINVYGNESIETSEILSIMEMKEEGVFEG

L_nogu_LEP1GSC059_0588 YVKVELGKPDPKTNLVRVRFIIDEGEEIPVSKINVYGNESIETSEILSIMEMKEEGVFEG

L_kmet_LEP1GSC052_1064 YVKVELGKPDPKTNLVRVRFIIDEGEEIPVSKINVYGNESIETSEILSVMEMKEEGVFEG

L_alst_LEP1GSC193_1910 YVKVELGKPDPKTNLVRVRFIIDEGEEIPVSKINVYGNESIETSEILSVMEMKEEGVFEG

L_sant_LEP1GSC048_3025 YVKVELGKPDSKTNLVRVRFVIDEGEEIPVSKINVYGNESIETSEILSVMEMKEEGVFEG

L_mayo_LEP1GSC190_2598 YVKVELGKPDSKTNLVRVRFVIDEGEEIPVSKINVYGNESIETSEILSVIEMKEEGIFEG

L_borg_LEP1GSC103_1744 YVKVELGKPDAKTNLVRVRFVIDEGEEIPVSKINVYGNESIETSEILSVMEMKEEGVFEG

L_alex_LEP1GSC062_2892 YVKVELGKPDSKTNLVRVRFVIDEGEEIPVSKINVYGNESIETSEILSVIEMKEEGVFEG

L_weil_LEP1GSC086_3808 YVKVELGKPDSKTNLVRVRFVIDEGEEIPVSKINVYGNESIETSEILSVIEMKEEGVFEG

**********.*********:***************************::******:***

L_inte_LIC11623 GNFKESSFEKDKDTIVAYLKSKGYLDAELIREGTNWEIHWENPEKKDRRVIIVNIKISEG

L_kirs_LEP1GSC049_2475 GNFKESSFEKDKDMIVAYLKSKGYLDAELIREGTNWEIHWENPEKKDRRVIIVNIKISEG

L_nogu_LEP1GSC059_0588 GNFKESSFEKDKDMIVAYLKSKGYLDAELIREGTNWEIHWENPEKKDRRVIIVNIKISEG

L_kmet_LEP1GSC052_1064 GNFKESSFEKDKDTIVAYLKSKGYLDAELIREGTNWEIHWENPEKKDRRVIIVNIKISEG

L_alst_LEP1GSC193_1910 GNFKESSFEKDKDTIVAYLKSKGYLDAELIREGTNWEIHWENPEKKDRRVIIVNIKISEG

L_sant_LEP1GSC048_3025 GNFKESSFEKDKDVIVAYLKSKGYLDAELVREGTNWEIHWENPEKKDRRVIIVNIKISEG

L_mayo_LEP1GSC190_2598 GNFKESSFEKDKDTIVAYLKSKGYLDAELIREGTNWEIHWENPEKKDRRVIIVNIKISEG

L_borg_LEP1GSC103_1744 GNFKESSFEKDKDTIVAYLKSRGYLDAELIREGTNWEIHWENPEKKDRRVIIVNVKISEG

L_alex_LEP1GSC062_2892 GNFKESSFEKDKDTIVAYLKSKGYLDAELIREGTNWEIHWENPEKKDRRVIIVNIKISEG

L_weil_LEP1GSC086_3808 GNFKESSFEKDKDTIVAYLKSKGYLDAELIREGTNWEIHWENPEKKDRRVIIVNIKISEG

************* *******.*******:************************:*****

L_inte_LIC11623 QVYFFNGYTLNHDMSLDGEGRPLFLNKEKNPPETTKEELKPLFTSKEIEKSLDYSDADVG

L_kirs_LEP1GSC049_2475 QVYFFNGYTLNHDMSLDGEGRPLFLNKEKNPPETTKEELKPLFTPKEIEKSLDYNDGDVG

L_nogu_LEP1GSC059_0588 QVYFFNGYNLNHDMSLDGEGRPLFLNKEKNPPETTKEELKPLFTPKEIEKSLDYNDADVG

L_kmet_LEP1GSC052_1064 QVYFFNGYTLNHDMSLDGEGRPLFLNKEKNPPETSKDELKPLFTPKEIERGLDYSDGDVG

L_alst_LEP1GSC193_1910 QVYFFNGYTLNHDMSLDGEGRPLFLNKEKNPPETTKDELKPLFVPKEIERTLDYNDGDVG

L_sant_LEP1GSC048_3025 QIYFFNGYTLNHDMSLDGEGRPLFLNKEKNPPETAKEELKPLFPPKEIERSLDYSDGDVG

L_mayo_LEP1GSC190_2598 QIYFFNGYTVNHDMSLDGEGRPLFLNKEKNPPETTKDELKPLFPPKEIERSLDYSDGDVG

L_borg_LEP1GSC103_1744 QIYFFNGYTVNHDMSLDGEGRPLFLNKEKNPPETAKDELKPLFSPKEIERSLDYSDGEVG

L_alex_LEP1GSC062_2892 QIYFFNGYTVNHDTSLDGEGRPLFLNKEKNPPETAKDELKPLFSPKEIEKSLDYSDGDVG

L_weil_LEP1GSC086_3808 QIYFFNGYTVNHDTSLDGEGRPLFLNKEKNPPETAKDELKPLFPPKEIEKSLDYSDGDVG

*:******.:*** ********************:*:****** .****. ***.*.:**

L_inte_LIC11623 VIFDETRFMRDRGAMNELYSSRGYLFAQVIPRRKIVSLDRENLEYYENCYSRKSEEERRI

L_kirs_LEP1GSC049_2475 VIFDETRFMRDRGTMNELYSSRGYLFAQVIPRRKIVSLDRENLEYYENCYSRKSEEERRI

L_nogu_LEP1GSC059_0588 VIFDETRFMRDRGTMNELYSSRGYLFAQVIPRRKIVSLDRENLEYYENCYSRKSEEERRI

L_kmet_LEP1GSC052_1064 VIFDETRFMRDRGAMNELYSSKGYLFAQVIPRRKVISLDRENIEYYENCYSRKSEEERKI

L_alst_LEP1GSC193_1910 VIFDETRFMRDRGAMNELYSSRGYLFAQVIPRRKVISLDRENLEYYENCYGRKSEEERKI

L_sant_LEP1GSC048_3025 AIFDETRFMRDRGTVNEMYSSRGYLFAQVIPRRKVVSLDRENLEYYENCYSRKSEEERRV

L_mayo_LEP1GSC190_2598 AIFDETRFMRDRGTVNEMYSSKGYLFAQVIPRRKVISLDRENLEYYENCYSRKSEEERKI

L_borg_LEP1GSC103_1744 AIFDETRFMRDRGTVNEMYSSKGYLFAQVIPRRKVVSLDRENLEYYENCYSRKSEEERKI

L_alex_LEP1GSC062_2892 AIFDETRFMRDRGTVNEMYSSKGYLFAQVIPRRKVISLDRENLEYYENCYSRKSEEERKI

L_weil_LEP1GSC086_3808 AIFDETRFMRDRGTVNEMYSSRGYLFAQVIPRRKVISLDRENLEYYENCYSRKSEEERKI

.************::**:***.************::******:*******.*******.:

L_inte_LIC11623 CENEYSQLHVKRLRQLYNTKPELHGKKFVHVDFNIRENNLAYVENVIIKGNKKTQDRVIR

L_kirs_LEP1GSC049_2475 CENEYSQLHVKRLRQLYNTKPELHGKKFVHVDFNIRENNLAYVENVVIKGNKKTQDRVIR

L_nogu_LEP1GSC059_0588 CENEYSQLHIKRLRQLYNTKPELHGKKFVHVDFNIRENNLAYVENVVIKGNKKTQDRVIR

L_kmet_LEP1GSC052_1064 CETEYTQLHVKRLRQLYNTKPELHGKKFVHVDFNIRENNLAYVENVVIKGNKKTQDRVIR

L_alst_LEP1GSC193_1910 CENEYSQLHIKRLRQLYNTKPELHGKKFVHVDFNIRENNLAYVENVVIKGNKKTQDRVIR

L_sant_LEP1GSC048_3025 CENEYSQLHIKRLRQLYNTKPELHGKKFVHVDFNIRENNLAYVENVIIKGNKKTQDRVIR

L_mayo_LEP1GSC190_2598 CENEYSQLHIKRLRQLYNTKSELHGKKFVHVDFNIRENNLAYVENVIIKGNKKTQDRVIR

L_borg_LEP1GSC103_1744 CENEYSQLHIKRLRQLYNTKPELHGKKFVHVDFNIRENNLAYVENVIIKGNKKTQDRVIR

L_alex_LEP1GSC062_2892 CENEYSQLHIKRLRQLYNTKPELHGKKFVHVDFNIRENNLAYVENVIIKGNKKTQDRVIR

L_weil_LEP1GSC086_3808 CENEYSQLHIKRLRQLYNTKPELHGKKFVHVDFNIRENNLAYVENVIIKGNKKTQDRVIR

**.**:***:**********.*************************:*************

L_inte_LIC11623 RELLFKQGDLFNSILVNRSRERIFNLGYFKEVNFNMRPGSDQTKMNLIIEVVEQPTGTVS

L_kirs_LEP1GSC049_2475 RELLFKQGDLFNSILVNRSRERIFNLGYFKEVNFNMRPGSDQTKMNLIIEVVEQPTGTVS

L_nogu_LEP1GSC059_0588 RELLFKQGDLFNSILVNRSRERIFNLGYFKEVNFNMRPGSDQTKMNLIIEVVEQPTGTVS

L_kmet_LEP1GSC052_1064 RELLFKQGDLFNSTLVNRSRERIYNLGYFKEVNFNMRPGSDQTKMNLIIEVLEQPTGTVS

L_alst_LEP1GSC193_1910 RELLFKQGDLFNSILVNRSRERIYNLGYFKEVNFNMRPGSDQTKMNLIIEVLEQPTGTVS

L_sant_LEP1GSC048_3025 RELLFKQGDLFNSILVNRSRERIYNLGYFKEVNFNMRPGSDQTKMNLIIEVLEQPTGTVS

L_mayo_LEP1GSC190_2598 RELLFKQGDLFNSILVNRSRERIYNLGYFKEVNFNMRPGSDQTKMNLIIEVLEQPTGTVS

L_borg_LEP1GSC103_1744 RELLFKQGDLFNSILVNRSRERIYNLGYFKEVNFNMRPGSDQTKMNLIIEVLEQPTGTVS

L_alex_LEP1GSC062_2892 RELLFKQGDLFNSILVNRSRERIYNLGYFKEVNFNMRPGSDQTKMNLIIEVLEQPTGTVS

L_weil_LEP1GSC086_3808 RELLFKQGDLFNSILVNRSRERIYNLGYFKEVNFNMRPGSDQTKMNLIIEVLEQPTGTVS

************* *********:***************************:********

L_inte_LIC11623 MGGGYGTITGFSIFTEVGENNLNGTGQKISGRLEFGPFRRLFQITWTEPWLYNKPWSLSL

L_kirs_LEP1GSC049_2475 MGGGYGTITGFSIFTEVGENNLNGTGQKISGRLEFGPFRRLFQITWTEPWLYNKPWSLSL

L_nogu_LEP1GSC059_0588 MGGGYGTITGFSIFTEVGENNLNGTGQKISGRLEFGPFRRLFQITWTEPWLYNKPWSLSL

L_kmet_LEP1GSC052_1064 MGGGYGTITGFSIFTEVGENNLNGTGQKISGRLEFGPYRRLFQITWTEPWLYNKPWSLSL

L_alst_LEP1GSC193_1910 MGGGYGTITGFSIFTEVGENNLNGTGQKISGRLEFGPFRRLFQITWTEPWLYNKPWSLSL

L_sant_LEP1GSC048_3025 MGGGYGTITGFSIFTEVGENNLNGTGQKISGRLEFGPFRRLFQITWTEPWLYNKPWSLSL

L_mayo_LEP1GSC190_2598 MGGGYGTITGFSIFTEVGENNLNGTGQKISGRLEFGPFRRLFQITWTEPWLYNKPWSLSL

L_borg_LEP1GSC103_1744 MGGGYGTITGFSIFTEVGENNLNGTGQKISGRLEFGPFRRLFQITWTEPWLYNKPWSLSL

L_alex_LEP1GSC062_2892 MGGGYGTITGFSIFTEVGENNLNGTGQKISGRLEFGPFRRLFQITWTEPWLYNKPWSLSL

L_weil_LEP1GSC086_3808 MGGGYGTITGFSIFTEVGENNLNGTGQKISGRLEFGPFRRLFQITWTEPWLYNKPWSLSL

*************************************:**********************

L_inte_LIC11623 SLFYSSRIYNVGAVSITENNNQQSIKEQAIYSRDGVGFTVGIGHRIFINWTHFHRYSPSI

L_kirs_LEP1GSC049_2475 SLFYSSRIYNVGAVSITENNNQQSIKEQAIYSRDGVGFTVGIGHRIFINWTHFHRYSPSI

L_nogu_LEP1GSC059_0588 SLFYSSRIYNVGAVSITENNNQQSIKEQAIYSRDGVGFTVGIGHRIFINWTHFHRYSPSI

L_kmet_LEP1GSC052_1064 SLFYSSRIYNVGAVSITENNNQSSIKEQAIYSRDGVGFTVGIGHRIFINWTHFHRYSPSI

L_alst_LEP1GSC193_1910 SIFYSSRIYNVGAVSITENNNQQSIKEQAVYSRDGVGFTVGIGHRIFINWTHFHRYSPSI

L_sant_LEP1GSC048_3025 SLFYSSRIYNVGAVSITENNNQQSIKEQAIYSRDGVGFTVGIGHRIFINWTHFHRYSPSI

L_mayo_LEP1GSC190_2598 SLFYSSRIYNVGAVSITENNNQQSIKEQAVYSRDGVGFTVGIGHRIFINWTHFHRYSPSI

L_borg_LEP1GSC103_1744 SLFYSSRIYNVGAVSITENNNQQSIKEQAIYSRDGVGFTVGIGHRIFINWTHFHRYSPSI

L_alex_LEP1GSC062_2892 SLFYSSRIYNVGAVSITENNNQQSIKEQAIYSRDGVGFTVGIGHRIFINWTHFHRYSPSI

L_weil_LEP1GSC086_3808 SLFYSSRIYNVGAVSITENNNQQSIKEQAIYSRDGVGFTVGIGHRIFINWTHFHRYSPSI

*:********************.******:******************************

L_inte_LIC11623 YASTNPSSLVSDQVLAEVRRGWQFRSQISNGIAYDIRDNVFNPTQGYDLLFQIDNVGQAL

L_kirs_LEP1GSC049_2475 YASTNPSSLVSDQVLAEVRRGWQFRSQISNGIAYDIRDNVFNPTQGYDLLFQIDNVGQAL

L_nogu_LEP1GSC059_0588 YASTNPSSLVSDQVLAEVRRGWQFRSQISNGIAYDIRDNVFNPTQGYDLLFQIDNVGQAL

L_kmet_LEP1GSC052_1064 YASTNPSSLVSDQVLAEVRRGWQFRSQISNGIAYDIRDNVFNPTQGYDLLFQMDNVGQLL

L_alst_LEP1GSC193_1910 YASTNPSSLVSDQVLAEVRRGWQFRSQVSNGIVYDIRDNVFNPTQGYDLLFQMDNVGQAL

L_sant_LEP1GSC048_3025 YASTNPSSLVSDQVLAEVRRGWQFRSQISNGIAYDIRDNVFNPTQGYDLLFQMDNVGQVL

L_mayo_LEP1GSC190_2598 YASTNPSSLVSDQVLAEVRRGWQFRSQISNGIAYDIRDNVFNPTQGYDLLFQMDNVGQAL

L_borg_LEP1GSC103_1744 YASTNPSSLVSDQVLAEVRRGWQFRSQISNGIAYDIRDNVFNPTQGYDLLFQMDNVGQAL

L_alex_LEP1GSC062_2892 YASTNPSSLVSDQVLAEVRRGWQFRSQISNGIAYDIRDNVFNPTQGYDLLFQMDNVGQAL

L_weil_LEP1GSC086_3808 YASTNPSSLVSDQVLAEVRRGWQFRSQVSNGIAYDIRDNVFNPTQGYDLLFQMDNVGQAL

***************************:****.*******************:***** *

L_inte_LIC11623 GGQSHFDQYRVLAEYYHTWFDYSFFGLFRNNALRRWRVVQEFRSSSLFTYQRVPYYGKQD

L_kirs_LEP1GSC049_2475 GGQSHFDQYRVLAEYYHTWFDYSLFGLFRNNALRRWRVVQEFRSSSLFTYQRVPYYGKQD

L_nogu_LEP1GSC059_0588 GGQSHFDQYRVLAEYYHTWFDYSLFGLFRNNALRRWRVVQEFRSSSLFTYQRVPYYGKQD

L_kmet_LEP1GSC052_1064 GGQSHFDQYRILAEYYHTWFDYSFFGLFRNNALRRWRVVQEFRSSSLFTFQRVPSYGKQD

L_alst_LEP1GSC193_1910 GGQSHFDQYRILAEYYHTWFDYSLFGLFRNNALRRWRVVQEFRSSSLFTYQRVPYYGKQD

L_sant_LEP1GSC048_3025 GGQSHFDQYRILAEYYHTWFDYSFFGLFRNNALKRWRVVQEFRSSSLFTYQRVPYYGRQD

L_mayo_LEP1GSC190_2598 GGQSHFDQYRVLAEYYHTWFDYSFFGLFRNNALRRWRVVQEFRSSSLFTFQRVPYYGKQD

L_borg_LEP1GSC103_1744 GGQSHFDQYRVLAEYYHTWFDYSFFGLFRNNALKRWRVVQEFRSSSLFTYQRVPYYGKQD

L_alex_LEP1GSC062_2892 GGQSHFDQYRVLAEYYHTWFDYSFFGLFRNNALKRWRVVQEFRSSSLFTYQRVPYYGKQD

L_weil_LEP1GSC086_3808 GGQSHFDQYRVLAEYYHTWFDYSFFGLFRNNALKRWRVVQEFRSSSLFTYQRVPYYGKQD

**********:************:*********.***************:**** **.**

L_inte_LIC11623 PIQKPYIQLQDLQFLGGYESLRGWFYNDAKYPAEWRDGAATRMMFGSELRFPIEPTLLWL

L_kirs_LEP1GSC049_2475 PIQKPYIQLQDLQFLGGYESLRGWFYNDAKYPTEWRDGAATRMMFGSELRFPIEPTLLWL

L_nogu_LEP1GSC059_0588 PIQKPYIQLQDLQFLGGYESLRGWFYNDAKYPTEWRDGAATRMMFGSELRFPIEPTLLWL

L_kmet_LEP1GSC052_1064 PIQNPYIQLQDLQFLGGYESLRGWFYNDAKYPTEWRDGAASRMIFGSELRFPIEPTLLWL

L_alst_LEP1GSC193_1910 PIQKPYIQLQDLQFLGGYESLRGWFYNDAKYPTEWRDGAATRMIFGSELRFPIEPTLLWL

L_sant_LEP1GSC048_3025 PIQRPYIQLQDLQFLGGYESLRGWFYNDAKYPTEWRDGAATRMVFGTELRFPIEPTLLWL

L_mayo_LEP1GSC190_2598 PIQRPYIQLQDLQFLGGYESLRGWFYNDAKYPTEWRDGAATRMLFSSELRFPIEPTLLWL

L_borg_LEP1GSC103_1744 PIQRPYIQLQDLQFLGGYESLRGWFYNDAKYPTEWRDGAATRMLFSSELRFPIEPTLLWL

L_alex_LEP1GSC062_2892 PIQRPYIQLQDLQFLGGYESLRGWFYNDAKYPTEWRDGAATRMLFSSELRFPIEPTLLWL

L_weil_LEP1GSC086_3808 PIQRPYIQLQDLQFLGGYESLRGWFYNDAKYPTEWRDGAATRMLFSSELRFPIEPTLLWL

***.****************************:*******:**:*.:*************

L_inte_LIC11623 VAFFDAGALYEEVNRATGVRKDLFETYDQRVREAQMKDPVGYYLANNYNLTALRKADYTF

L_kirs_LEP1GSC049_2475 VAFFDAGALYEEVNRATGVRKDLFESYDQRVKEAQMKDPVGYYLANNYNLTALRKSDYTF

L_nogu_LEP1GSC059_0588 VAFFDAGALYEEVNRATGVRKDLFETYDQRVREAQMKDPVGYYLANNYNLTALRKADYTF

L_kmet_LEP1GSC052_1064 VAFFDAGALYEEVNRATGVRRDLFTTYDQRIREAQLKDPVGYALTNNYNLSALRKADYTY

L_alst_LEP1GSC193_1910 VAFFDAGALYEEVNRATGVRRDLFTTYDQRVREAQLKDPVGYALANNYNLNALRKADYTY

L_sant_LEP1GSC048_3025 VAFFDAGALYEEVNRATGVRRDLFTTYDQRIKEAQLKDPVAYALSNHYNLNALRKADYTY

L_mayo_LEP1GSC190_2598 VAFFDAGALYEEVNRATGVRRDLFTTYDQRVREAQLKDPVGYALSNHYNLNALRKADYTY

L_borg_LEP1GSC103_1744 VAFFDAGALYEEVNRATGVRRDLFTTYDQRVREAQLKDPIGYALSNYYNLNALRKADYTY

L_alex_LEP1GSC062_2892 VAFFDAGALYEEVNRATGVRRDLFTTYDQRIREAQLKDPIGYALSNHYNLNALRKADYTY

L_weil_LEP1GSC086_3808 VAFFDAGALYEEVNRATGVRRDLFTTYDQRVREAQLKDPMGYALSNHYNLNALRKADYTY

********************.*** :****:.***:***:.* *:* ***.****:***:

L_inte_LIC11623 EELNNPANLVLSGNNVALDKFRFSWGVGLRIQIPVLPLRIYFAQKLKYTGVADHPFTKFE

L_kirs_LEP1GSC049_2475 EELNNPANLVLSGNNIALDKFRFSWGIGLRIQIPVLPLRIYFAQKLKYTGVADHPFTKFE

L_nogu_LEP1GSC059_0588 EELNNPANLVLSGNNIALDRFRFSWGIGLRIQIPVLPLRIYFAQKLKYTGVADHPFTKFE

L_kmet_LEP1GSC052_1064 EELNNPANLVLSGNNIALDKFRFSWGVGLRIQIPVLPLRIYFAQKLKYSGVADHPFTKFD

L_alst_LEP1GSC193_1910 EELNNPANLVLSGNNVALDKFRFSWGIGLRIQIPVLPLRIYFAQKLKYTGVADHPFTKFE

L_sant_LEP1GSC048_3025 EELNNPANLVLSGNNIALDKFRFSWGIGLRIQIPVLPLRIYFAQKLKYSGVPDHPFTKFE

L_mayo_LEP1GSC190_2598 EELNNPANLVLSGNNIALDKFRFSWGIGLRIQIPVLPLRIYFAQKLKYSGVPDHPFTKFE

L_borg_LEP1GSC103_1744 EELNNPANLVLSGSNIALDKFRFSWGIGLRIQIPVLPLRIYFAQKLKYSGVPDHPFTKFE

L_alex_LEP1GSC062_2892 EELNNPANLVLSGNNIALDKFRFSWGIGLRIQIPVLPLRIYFAQKLKYSGVPDHPFTKFE

L_weil_LEP1GSC086_3808 EELNNPANLVLSGNNIALDKFRFSWGIGLRIQIPVLPLRIYFAQKLKYSGVPDHPFTKFE

*************.*:***.******:*********************:**.*******:

L_inte_LIC11623 SDNAFQFVFGIGDYRF

L_kirs_LEP1GSC049_2475 SDNAFQFVFGIGDYRF

L_nogu_LEP1GSC059_0588 SDNAFQFVFGIGDYRF

L_kmet_LEP1GSC052_1064 SDNAFQFVFGIGDYRF

L_alst_LEP1GSC193_1910 SDNAFQFVFGIGDYRF

L_sant_LEP1GSC048_3025 SDNAFQFVFGIGDYRF

L_mayo_LEP1GSC190_2598 SDNAFQFVFGIGDYRF

L_borg_LEP1GSC103_1744 SDNAFQFVFGIGDYRF

L_alex_LEP1GSC062_2892 SDNAFQFVFGIGDYRF

L_weil_LEP1GSC086_3808 SDNAFQFVFGIGDYRF

****************
