# Supplementary material for: Discovery of Novel Leptospirosis Vaccine Candidates Using Reverse and Structural Vaccinology
Source: Front Immunol. 2017 Apr 27;8:463. doi: 10.3389/fimmu.2017.00463 (PMC5406399; doi:10.3389/fimmu.2017.00463)
Supplement: Supplementary file 8 [file Data_Sheet_1.ZIP › Alignment Bb-OMPs/Mult_alignment_LIC12254_path_spp_orthol_immun_epit_highlighted.docx]

L_kmet_LEP1GSC052_1741 MKKFLKLVSIATCLFALTLTAGSVYAQDKEDCSKLAFVDYSNRKPRTDLPFEISEMRRLR

L_sant_LEP1GSC048_3800 MKKFLKIVGVTTSLF--FLTAGAAYG--QEDCSKLAFMEDVSRKPRTDLPFPISEMRRLR

L_weil_LEP1GSC086_4082 MKKFLKIVGVATSLF--FLTVGAAYG--QEDCSKLAFMEDVARKPRTDLPFQISEMRRLR

L_mayo_LEP1GSC190_2981 MKRFLKIVSVAAGSF--FLTAGVAYG--QEDCSKLAFMDDVSRKPRTDLPFQISEMRRLR

L_borg_LEP1GSC103_1305 MKRFLKIVGVAAGLF--FLTAGAAYG--QEDCSKLSFMDDVSRKPRTDLPFQISEMRRLR

L_alex_LEP1GSC062_3377 MKRFLKIIGVVAGLF--FLTAGAAYA--QEDCSKLAFMDDVSRKPRTDLPFQISEMRRLR

L_alst_LEP1GSC193_4396 MKKFLKIVSVAMSLL--FLTAGSTYG--QEDCSKLAFTDDVARKPRTDLPFFISEMRRLR

L_inte_LIC12254 MKKFLIFVSFVISLF--FVTTGAVYGQ-QEDCSKLAFMDDVQRKPRTDLPFPISEMRRLR

L_kirs_LEP1GSC049_1839 MKKFLIFVSFAISLL--FVATGAAYGQ-QEDCSKLAFMDDVQRKPRTDLPFPISEMRRLR

L_nogu_LEP1GSC059_1782 MKKFLIFVSFAISLF--FVTTGAAYGQ-QQDCSKLAFMDDVQRKPRTDLPFPISEMRRLR

**.** ::... : ::.* .*. ::*****:* : ********* ********

L_kmet_LEP1GSC052_1741 PEDICKKKEGWFPTGLPLLNSDPNVGVGYGVRVFLINNGKKSDPFFEYAPYRFRMFAQYF

L_sant_LEP1GSC048_3800 PEDICKKKEGWFPTGLPLLNSDPNVGVGYGVRVFLINNGKKTDPFFEYTPYRFRMFAQYF

L_weil_LEP1GSC086_4082 PEDICKKKEGWFPTGLPLLNSDPNVGVGYGVRVFLINNGKKTDPFFEYTPYRFRMFAQYF

L_mayo_LEP1GSC190_2981 PEDICKKKEGWFPTGLPLLNSDPNVGVGYGVRVFLINNGKKTDPFFEYTPYRFRMFAQYF

L_borg_LEP1GSC103_1305 PEDICKKKEGWFPTGLPLLNSDPNVGVGYGVRVFLINNGKKTDPFFEYTPYRFRMFAQYF

L_alex_LEP1GSC062_3377 PEDICKKKEGWFPTGLPLLNSDPNVGVGYGVRVFLINNGKKTDPFFEYTPYRFRMFAQYF

L_alst_LEP1GSC193_4396 PEDICKKKEGWFPTGLPLLNSDPNVGVGYGVRVFLINNGKKSDPFFEYTPYRFRMFAQYF

L_inte_LIC12254 PEDICKKKEGWFPTGLPLLNSDPNVGVGYGVRVFLINNGKKSDPFFEYAPYRFRMFAQYF

L_kirs_LEP1GSC049_1839 PEDICKKKEGWFPTGLPLLNSDPNVGVGYGVRVFLINNGKKSDPFFEYAPYRFRMFAQYF

L_nogu_LEP1GSC059_1782 PEDICKKKEGWFPTGLPLLNSDPNVGVGYGVRVFLINNGKKSDPFFEYAPYRFRMFAQYF

*****************************************:******:***********

L_kmet_LEP1GSC052_1741 NTTKNRQYQDVSFDAPYIFDTKWRLRGDLIYDTNPNTLYYGIGEKSLETLSYQERNQPGG

L_sant_LEP1GSC048_3800 NTTKNRQYQDISFDAPYIFDTKWRLRGDLIYDTNPNTLYYGVGERSLQSLSYQERNQPGG

L_weil_LEP1GSC086_4082 NTTKNRQYQDISFDAPYIFDTKWRLRGDLIYDTNPNTLYYGIGERSLQNLSYQERNQPGG

L_mayo_LEP1GSC190_2981 NTTKNRQYQDISFDAPYIFDTKWRLRGDLIYDTNPNTLYYGIGERSLQNLSYQERNQPGG

L_borg_LEP1GSC103_1305 NTTKNRQYQDISFDAPYIFDTKWRLRGDLIYDTNPNTLYYGIGERSLQNLSYQERNQPGG

L_alex_LEP1GSC062_3377 NTTKNRQYQDISFDAPYIFDTKWRLRGDLIYDTNPNTLYYGVGERSLQSLSYQERNQPGG

L_alst_LEP1GSC193_4396 NTTKNRQYQDISFDAPYVFDTKWRLRGDLIYDTNPNTLYYGIGEKSLETLSYQERNQSGG

L_inte_LIC12254 NTTKNRQYQDISFDAPYIFDTKWRLRGDLIYDTNPNTLYYGIGERSLETLSYQERNQSGG

L_kirs_LEP1GSC049_1839 NTTKNRQYQDISFDAPYVFDTKWRLRGDLIYDTNPNTLYYGIGEKSLESLSYQERNQPGG

L_nogu_LEP1GSC059_1782 NTTKNRQYQDISFDAPYVFDTKWRLRGDLIYDTNPNTLYYGIGEKSLETLSYQERNQPGG

**********:******:***********************:**.**:.********.**

L_kmet_LEP1GSC052_1741 EVVRNATYHDREKNIYFTRPGGPGDPVEFQGNNYSGFPTNDAFRVTDRMYNRYDIRSPQA

L_sant_LEP1GSC048_3800 EIVRNSTYHEREKNIYFTRPGGPGDPIDFQGANYSGFPTNDAFRVTDRMYNRYDIRSPQF

L_weil_LEP1GSC086_4082 EIARNATYHDREKNIYFTRPGGPGDPVDFQGTNYSGFPTNDAFRVTDRMYNRYDIRSPQL

L_mayo_LEP1GSC190_2981 EIARNATYHEREKNIYFTRLGGPGDPIDFQGANYSGFPTNDAFRVTDRMYNRYDIRSPQF

L_borg_LEP1GSC103_1305 EIARNSTYHEREKNIYFTRPGGPGDPIDFQGANYSGFPVNDAFRVTDRMYNRYDIRSPQF

L_alex_LEP1GSC062_3377 EIARNSTYHEREKNIYFTRPGGPGDPVDFQGANYSGFPTNDAFRVTDRMYNRYDIRSPQF

L_alst_LEP1GSC193_4396 EIVRNATYHEREKNIYFTRPGGPGDPLDFQGTNYSGFPANDAFRVTDRMYNRYDIRSPQA

L_inte_LIC12254 EVVRNATYHEREKNIYFTRPGGPGDPVDFQGTNYSGFPNNDAFRVTDRMYNRYDIRSPQA

L_kirs_LEP1GSC049_1839 EVVRNATYHEREKNIYFTRPGGPGDPVDFQGTNYSGFPNNDAFRVTDRMYNRYDIRSPQA

L_nogu_LEP1GSC059_1782 EVVRNATYHEREKNIYFTRPGGPGDPLDFQGTNYSGFPNNDAFRVTDRMYNRYDIRSPQA

*:.**:***:********* ******::*** ****** ********************

L_kmet_LEP1GSC052_1741 NVSGEHIFFGGLVRMVAGLRVSQNIVKTFDGQFVKSTDPLTDGTPFSNSGLAPAGKTKVT

L_sant_LEP1GSC048_3800 NLSGEHIFFGGLIRMVAGLRVSQNIIKTFDGQFVKSKDPLTEGTPFSNSGMTPNGKTKIT

L_weil_LEP1GSC086_4082 NLSGEHIFFGGLVRMVAGLRVSQNIVKTFDGQFVKSRDPLTEGTPFSNSGMAPNGKTKVI

L_mayo_LEP1GSC190_2981 NLSGEHIFFGGLVRMVAGLRVSQNIVKTFDGQFVKSRDPLTEGTPLSNSGMAPNGKTKIT

L_borg_LEP1GSC103_1305 NLSGEHIFFGGLVRMVAGLRVSQNTVKTFDGQFVKSRDPLTEGTPFSNSGMTPNGKTKVT

L_alex_LEP1GSC062_3377 NLSGEHIFFGGLVRMVAGLRVSQNIVKTFDGQFVKSRDPLTEGTPFSNSGMAPNGKTKVT

L_alst_LEP1GSC193_4396 NISGEHIFFGGLVRTVAGLRVSQNIIKTFDGQFVKSTDPLTEGTPLSNSGTAPNAKTKVT

L_inte_LIC12254 NLSGEHIFFGGLVRAVAGIRVSQNIIKIFDGQFVKSTDPLTEGTPFSNSGITPNGKTKVT

L_kirs_LEP1GSC049_1839 NLSGEHIFFGGLVRTVAGVRVSQNIIKTFDGQFVKSTDPLTEGTPFSNSGMTPNGKTKVT

L_nogu_LEP1GSC059_1782 NLSGEHIFFGGLVRTVAGIRVSQNIIKTFDGQFVKSTDPLTEGTPFSNSGMTPNAKTKVT

*:**********:* ***:***** :* ******** ****:***:**** :* .***:

L_kmet_LEP1GSC052_1741 EDAQAGRIIGANGGNVNSVRFGLVLDTRDLEPDPNRGVFLEATYEKVAKSLGSDFQYSKY

L_sant_LEP1GSC048_3800 EDAEAGKIIGANGGNVNSVRFGLVLDTRDLEPDPNRGIFLEATYEKVAKSFGSDFQYSKY

L_weil_LEP1GSC086_4082 EDAEAGKIIGANGGNVNSVRFGLVLDTRDLEPDPNRGVFLEATYEKIAKSFGSDFQYSKY

L_mayo_LEP1GSC190_2981 EDAEAGKIIGANGGNVNSLRFGLVLDTRDLEPDPNRGIFLEATYEKIAKSFGSDFQYSKY

L_borg_LEP1GSC103_1305 EDADAGKIIGANGGNVNSVRFGLVLDTRDLEPDPNRGIFLEATYEKIAKSFGSDFQYSKY

L_alex_LEP1GSC062_3377 EDAEAGKIIGANGGNVNSMRFGLVLDTRDLEPDPNRGVFLEATYEKIAKSFGSDFQYSKY

L_alst_LEP1GSC193_4396 EDAEAGKIIGANGGNVNSIRFGLVLDTRDLEPDPNRGMFLETTYEKVAKSFGSDFQYSKY

L_inte_LIC12254 EDAEAGKIIGANGGNVKSVRFGLVLDTRDLEPDPNRGMFVEATYEKVAKAFGSDFQYSKY

L_kirs_LEP1GSC049_1839 EDAEARKIIGANGGNVNSVRFGLVLDTRDLEPDPNRGMFVEATYEKVAKAFGSDFQYSKY

L_nogu_LEP1GSC059_1782 EDAEAGKIIGANGGNVNSVRFGLVLDTRDLEPDPNRGMFVEATYEKVAKAFGSDFQYSKY

***:* .*********:*:******************:*:*:****:**::*********

L_kmet_LEP1GSC052_1741 FTQMKVFYSPFPKTFDKLVIAGRGAFGMTDGDAPFFEYRNLWSTEGGITGLGGLRTLRGY

L_sant_LEP1GSC048_3800 FTQIKLFYSPFPKVFDKLVIAGRGAFGLTEGDAPFFEYRNLWSTEGGITGLGGLRTLRGY

L_weil_LEP1GSC086_4082 FTQIKLFYSPFPKVFDKLVIAGRGAFGLTEGEAPFFEYRNLWSTEGGITGIGGLRTLRGY

L_mayo_LEP1GSC190_2981 FTQIKLFYSPFPKVFDKLVIAGRGAFGLTEGDAPFFEYRNLWSTEGGITGLGGLRTLRGY

L_borg_LEP1GSC103_1305 FTQIKLFYSPFPKVFDKLVIAGRGAFGLTEGEAPFFEYRNLWSTEGGITGLGGLRTLRGY

L_alex_LEP1GSC062_3377 FTQIKLFYSPFPKVFDKLVIAGRGAFGLTEGEAPFFEYRNLWSTEGGITGLGGLRTLRGY

L_alst_LEP1GSC193_4396 FTQLKLFYSPFPKVFDKLVIAGRGAFGLTEGDAPFFEYRNLWSTEGGITGLGGLRTLRGY

L_inte_LIC12254 FTQVKLFYSPFPKVFDKLVIAGRGAFGLTEGDAPFFEYRNLWSTEGGITGLGGLRTLRGY

L_kirs_LEP1GSC049_1839 FTQIKLFYSPFPKVFDKLVIAGRGAFGLTEGDAPFFEYRNLWSTEGGITGLGGLRTLRGY

L_nogu_LEP1GSC059_1782 FTQIKLFYSPFPKVFDKLVIAGRGAFGLTEGDAPFFEYRNLWSTEGGITGLGGLRTLRGY

***:*:*******.*************:*:*:******************:*********

L_kmet_LEP1GSC052_1741 KQDRFAGKAMGWGNVEVRWKFFDFTVAGQHFALNLVPFMDFGRVWDDEHNVGLKDYKYSR

L_sant_LEP1GSC048_3800 KQDRFTGKAMGWGNVELRWKFFDFNVAGQHFALNLVPFVDFGRVWDDEHNVGLKDYKYSR

L_weil_LEP1GSC086_4082 KQDRFTGKAMGWGNIELRWKFFDFNIAGQHFALNLVPFVDFGRVWDDEHNVGLKDYKYSR

L_mayo_LEP1GSC190_2981 KQDRFTGKAMGWGNVELRWKFFDFNVAGQHFALNLVPFMDFGRVWDDEHNVGLKDYKYSR

L_borg_LEP1GSC103_1305 KQDRFTGKAMGWGNVELRWKFFDFNVAGQHFALNLVPFVDFGRVWDDEHNVGLKDYKYSR

L_alex_LEP1GSC062_3377 KQDRFTGKAMGWGNIELRWKFFDFNVAGQHFALNLVPFVDFGRVWDDEHNVGLKDYKYSR

L_alst_LEP1GSC193_4396 KQDRFAGKAMGWGNIELRWKFFDFNVAGQHFALNLVPFMDFGRVWDDEHKVGLKDYKYSR

L_inte_LIC12254 KQDRFAGKAMGWGNIELRWKFFDFNIAGQHFALNLVPFMDFGRVWDDEHNVGLKDYKYSR

L_kirs_LEP1GSC049_1839 KQDRFTGKAMGWGNIELRWKFFDFNIAGQHFALNLVPFMDFGRVWDDEHNVGLKDYKYSR

L_nogu_LEP1GSC059_1782 KQDRFAGKAMGWGNLELRWKFFDFNIAGQHFALNLVPFVDFGRVWDDEHNVGLKDYKYSK

*****:********:*:*******.:************:**********:*********.

L_kmet_LEP1GSC052_1741 GMGFRIAWNQSTILMLDYAVSKEDKQIFMNFNHIF

L_sant_LEP1GSC048_3800 GLGFRIAWNQSTILMLDYAVSKEDKQIFMNFNHIF

L_weil_LEP1GSC086_4082 GLGFRIAWNQSTILMLDYAVSKEDKQIFMNFNHIF

L_mayo_LEP1GSC190_2981 GLGFRIAWNQSTILMLDYAISKEDKQIFMNFNHIF

L_borg_LEP1GSC103_1305 GLGFRIAWNQSTILMLDYAVSKEDKQIFMNFNHIF

L_alex_LEP1GSC062_3377 GLGFRIAWNQSTILMLDYAVSKEDKQIFMNFNHIF

L_alst_LEP1GSC193_4396 GLGFRIAWNQSTILMLDYAISKEDKQVFMNFNHIF

L_inte_LIC12254 GLGFRIAWNQSTILMLDYAVSKEDKQVFMNFNHIF

L_kirs_LEP1GSC049_1839 GLGFRIAWNQSTILMLDYAVSKEDKQVFMNFNHIF

L_nogu_LEP1GSC059_1782 GLGFRIAWNQSTILMLDYAVSKEDKQVFMNFNHIF

*:*****************:******:********
