# Supplementary material for: Discovery of Novel Leptospirosis Vaccine Candidates Using Reverse and Structural Vaccinology
Source: Front Immunol. 2017 Apr 27;8:463. doi: 10.3389/fimmu.2017.00463 (PMC5406399; doi:10.3389/fimmu.2017.00463)
Supplement: Supplementary file 8 [file Data_Sheet_1.ZIP › Alignment Bb-OMPs/Mult_alignment_LIC12374_path_spp_orthol_immun_epit_highlighted.docx]

L_nogu_LEP1GSC059_2710 MGSNQYIQILKYSVTLFFLVLGRNLAAQDRTPNIVPVTKTGTVQKAETVNVVGKPVDAGS

L_inte_LIC12374 MGKNQYIQILKYSVIPFLLVFALTLSAQNQVP----------ATKAETVQVIGKVPDSSS

L_kirs_LEP1GSC049_1720 MGSNRYIQILKYSVILFFPVLGKTLNSQDHTSNVV------SSPKTETVQVIGKVSNSGP

L_sant_LEP1GSC048_0703 MGSTKRILILEYSIILFFSVFVSEIFAQDDRNRNT-TT-TESAQKTETVRVIGKVSDSGS

L_alex_LEP1GSC062_4342 MKILKYILFLEYSIILFFYIFVSGIIAQDNSSSTI-SQKTDSVQKAETVQVIGKVSDSDS

L_weil_LEP1GSC086_0503 MGSIKYIRLLEYSIILFFSIFVSGIAARDNSSSKI-AQKTDSVQKAETVRVIGKVSDSGS

L_borg_LEP1GSC103_1969 MRLFNYILILEYSIILLFSIFVSGIVAQDNSDSII-------VQKAETVRVIGKVSDSGS

L_mayo_LEP1GSC190_2376 MRLFNYILILEYSIILFFSIFVSGIAAQDNLNSTT-------AQKTETVQVIGKVPDSGS

L_alst_LEP1GSC193_0115 MGSIKYIRILEYSIILFFSIFVSGIIAQDNSSSTA-AQKKDSVQKAETVRVVGKVADSGS

L_kmet_LEP1GSC052_2138 MALKKYIRRLEYSFFLFF-LIVRGLSAQEDRNRNS-VT-TEPTQKAETVQVVGKVSDSGS

* . * *:**. :: :: : :.: *:***.*:** ::..

L_nogu_LEP1GSC059_2710 QNFRSNPSGFQTAIQLDETSARYTSLPEVLEREAGLRVRSFGGLGSYSTLSIRGTNPNQS

L_inte_LIC12374 QNFRSNPSGFQTAIKLDETSARYTSLPEVLEREAGLRVRSFGGLGSYSTLSIRGTNPNQS

L_kirs_LEP1GSC049_1720 QNFRSNPSGFQTAIQLDETSARYTSLPEVLEREAGLRVRSFGGLGSYSTLSIRGTNPNQS

L_sant_LEP1GSC048_0703 QNFRSNPSGFQSAIQLDETSARYTSLPEVLEREAGLRIRSFGGLGSYSTLSIRGTNPNQS

L_alex_LEP1GSC062_4342 QNFRSNPSGFQSAIKLDGASARYTSLPEVLEREAGLRVRSFGGLGSYSTLSIRGTNPNQS

L_weil_LEP1GSC086_0503 QNFRSNPSGFQSAIKLDETSARYTSLPEVLEREAGLRVRSFGGLGSYSTLSIRGTNPNQS

L_borg_LEP1GSC103_1969 QNFRSNPSGFQSAIKLDETSARYTSLPEVLEREAGLRVRSFGGLGSYSTLSIRGTNPNQS

L_mayo_LEP1GSC190_2376 QNFRSNPSGFQSAIKLDETSVRYTSLPEVLEREAGLRVRSFGGLGSYSTLSIRGTNPNQS

L_alst_LEP1GSC193_0115 SNFRSNPSGFQSAIKLDETSARYTSLPEVLEREAGLRVRSFGGLGSYSTLSIRGTNPNQS

L_kmet_LEP1GSC052_2138 QNFRSNPSGFQSAIKLDETSARYTSLPEVLEREAGLRVRSFGGLGSYSTLSIRGTNPNQS

.**********:**:** :*.****************:**********************

L_nogu_LEP1GSC059_2710 RIYLDGIPLNNSQGGEVNLADLPFDSLESVEVYRSGNPIGFSGSAIGGSVNLVTKKDSGK

L_inte_LIC12374 RIYLDGIPLNNSQGGEVNLADLPFDSLESVEVYRSGNPIGFSGSAIGGSVNLVTKKDSQK

L_kirs_LEP1GSC049_1720 RIYLDGIPLNNSQGGEVNLADLPFDSLESVEVYRSGNPIGFSGSAIGGSVNLVTKKDSQK

L_sant_LEP1GSC048_0703 RIYLDGIPLNNSQGGEVNLADLPFDSLESVEVYRSGNPIGFSGSAIGGSVNLVTRKNTGK

L_alex_LEP1GSC062_4342 RIYLDGIPLNNSQGGEVNLADLPFDSLESVEVYRSGNPIGFSGSAIGGGVNLVTRKDTSK

L_weil_LEP1GSC086_0503 RIYLDGIPLNNSQGGEVNLADLPFDSLESVEVYRSGNPIGFSGSAIGGGVNLVTRKDTNK

L_borg_LEP1GSC103_1969 RIYLDGIPLNNSQGGEVNLADLPFDSLESVEVYRSGNPIGFSGSAIGGSVNLVTRKDTGK

L_mayo_LEP1GSC190_2376 RIYLDGIPLNNSQGGEVNLADLPFDSLESVEVYRSGNPIGFSGSAIGGSVNLVTRKDSGK

L_alst_LEP1GSC193_0115 RIYLDGIPINNSQGGEVNLADLPFDSLESVEVYRSGNPIGFSGSAIGGSVNLVTRKDSGK

L_kmet_LEP1GSC052_2138 RIYLDGIPLNNSQGGEVNLADLPFDSLESVEVYRSGNPIGFSGSAIGGSVNLVTRKDSGK

********:***************************************.*****.*:: *

L_nogu_LEP1GSC059_2710 PKTRINLGGGSFNTGKASVSHAGNYKGIGTSFLVLGEKSDQNFSYKNDHGTVVLNTLDDT

L_inte_LIC12374 PKTRINLGGGSFNTGKASVSHTGNYKGIGTSFLALGEKSDQNFSYKNDHGTVVLNTLDDT

L_kirs_LEP1GSC049_1720 PKTRINLGGGSFNTGKASVSHTGNYKGIGTSFLVLGEKSDQNFSYKNDHGTVVLNTLDDT

L_sant_LEP1GSC048_0703 PRTRFNLGGGSFNTGKASVSHTGTYNGIGASFLALGEKSDQNFSFKNDHGTVVLNTLDDT

L_alex_LEP1GSC062_4342 PRTRINIGGGSFHTGKASVSHTGTYNGIGASFLALGEKSDQNFSFKNDHGTVVLNTLDDT

L_weil_LEP1GSC086_0503 PRTRINIGGGSFNTGKASLSHTGTYNGVGASFLALGEKSDQNFSFKNDHGTVVLNTLDDT

L_borg_LEP1GSC103_1969 PRTRVNLGGGSFNTGKASVSHTGTYNGIGASFLALGEKSDQNFSFKNDHGTVVLNTLDDT

L_mayo_LEP1GSC190_2376 PRTRVNLGGGSFNTGKASISHTGTYNGIGASFLALGEKSDQNFSFKNDHGTVVLNTLDDT

L_alst_LEP1GSC193_0115 PKTRINLGGGSFNTGKASISHTGTYKGIGTSFLALGEKSDQNFSFKNDHGTVVLNALDDT

L_kmet_LEP1GSC052_2138 PKTRINLGGGSFNTGKASVSHTGTYNGIGTSFLALGEKSDQNFSFKNDHGTVVLNTLDDT

*.**.*:*****:*****:**:*.*:*:*:***.**********:**********:****

L_nogu_LEP1GSC059_2710 IDRRKNAAFERAALFGSLKYQIGKTELKFLNDFNHRIHGLPGPGSNQADRVHRKYDRNTG

L_inte_LIC12374 IDRRKNASFERAALFGSLKYQIGKTELKFLNDFNHRIHGLPGPGSNQTDRVHRKYDRNTS

L_kirs_LEP1GSC049_1720 IDRRKNAAFERAALFGSLKYQIGKTELKFLNDFNHRIHGLPGPGSNQTDRVHRKYDRNTS

L_sant_LEP1GSC048_0703 IDRRKNAAFERSALFGTLKYQIGKTELKLLNDFNHRIHGLPGPGSNQTNRVHRKYDRYTG

L_alex_LEP1GSC062_4342 IDRRRNAAFERAALFGTLKYQIGKTELKLLNDFNHRIHGLPGPGSNQTNRVHRKYDRYMG

L_weil_LEP1GSC086_0503 IDRRRNAAFERAALFGTLKYQIGKTELKLLNDFNHRIHGLPGPGSNQTNRVHRKYDRYMG

L_borg_LEP1GSC103_1969 IDRRRNAAFEKAALFGTLKYQMGKTELKLLNDFNHRIHGLPGPGSNQTNRVHRKYDRYTG

L_mayo_LEP1GSC190_2376 IDRRRNSAFERAALFGTLKYQIGKTELKLLNDFNHRIHGLPGPGSNQTNRVHRKYGRYTG

L_alst_LEP1GSC193_0115 MDRRRNAAFERAALFGTLKYQIGKTELKLLNDFNHRIHGLPGPGSNQTNQVHRKYDRYTG

L_kmet_LEP1GSC052_2138 IDRRKNAAFERAALFGTLKYQIGKTELKLLNDFNHRIHGLPGPGSNQTNQVHRKYDRYTG

:***.*::**.:****:****:******:******************::.*****.* .

L_nogu_LEP1GSC059_2710 SFATDTKGLFVDSFRLETRSFYTAARDDLYDPLSEFSKGTPNSRAEIRQAGFQIMPTLYL

L_inte_LIC12374 SFATDTKGLFVDSFRLETRGFYTAAKDDLYDPLSEFSKGTPNSRAEIRQAGFQVMPTLYL

L_kirs_LEP1GSC049_1720 SFATDTKGLFVDSFRLETRSFYTAAKDDLYDPLSEFSKGTPNSRAEIRQAGFQIIPTLYL

L_sant_LEP1GSC048_0703 SFSTDTKELWIDSLRLETRSFYTAAKDDLFDPGSEFSKGIPNSRAEIRQAGIQVIPTLYL

L_alex_LEP1GSC062_4342 SFATDTKGLFVDSFRLESRSFYTAAKDDLFDPGSEFSKGTPNSRAEIRQMGVQLMPTLYL

L_weil_LEP1GSC086_0503 SFATDTKGLFVDSFRLETRSFYTAAKDDLFDPGSEFSKGTPNSRAEIRQMGVQLIPTLYL

L_borg_LEP1GSC103_1969 SFATDTKGLFVDFFRLETRSFYTAAKDDLFDPGSEFSKGTPNSRAEIRQAGIQIMPTLYL

L_mayo_LEP1GSC190_2376 SFATDTKGLFVDSFRLESRSFYTAAKDDLFDPGSEFSKGTPNSRAEIRQAGVQIMPTLYL

L_alst_LEP1GSC193_0115 SFATDTKALFVDSFRLESRNFYTVAKDELFDPGSEFSKGTPNSRAEIRQAGVQLMPTLYL

L_kmet_LEP1GSC052_2138 SFATDTKGLFVDSFRLETRSFYTAAKDDLFDPGSEFSKGTPNARAEIRQTGIQLMPTLYL

**:**** *::* :***:*.***.*.*:*:** ****** **:****** *.*::*****

L_nogu_LEP1GSC059_2710 TDYHQILRGFVSLEKESFDRERLTSSNVVGRVEPKKERTYSSFRLEDEIRFWNSKILLIP

L_inte_LIC12374 TDYHQILRGFVSLEKESFDRERLTSSNLVGRVEPKKERTYSSFRLEDEIRIWNSKILLIP

L_kirs_LEP1GSC049_1720 TDYHQILRGFMSLEKESFDRERLTSSNVVGRVEPKKERTYSSFRLEDEIRFWNSKILLIP

L_sant_LEP1GSC048_0703 TDYYQIVRGFVSLEKESFDRQRLTPSNVVGKVEPLKERTYSSFRLEDEVRLWNAKILLIP

L_alex_LEP1GSC062_4342 TDYYQILRAFASLEKESFDRNRLTPSNVIGRVEPLKERMYSSFRLEDEVRLWNAKVLLIP

L_weil_LEP1GSC086_0503 TDYYQILRAFVSLEKESFDRNRLTPSNVIGRAEPLKERMYSSFRLEDEVRLWNAKVLLIP

L_borg_LEP1GSC103_1969 TDYHQILRAFVSLERESFDRNRLTPSNVIGRVEPLKERTYSSFRLEDEVRLWNAKVLLVP

L_mayo_LEP1GSC190_2376 TDYYQILRVFASLEKESFDRNRLTPSNIIERVEPLKERMYSSFRLEDEVRLWNAKVLLVP

L_alst_LEP1GSC193_0115 TDYYQIVRGFASLEKESFDRDRLTPTNIVGRVEPLKERTYSSFRLEDEIRLWDAKVLLIP

L_kmet_LEP1GSC052_2138 TDYNQIIRGFASLEKESFDRERLTPSNIIGRVEPMKERTYSSFRLEDEVRLWNAKVLLIP

*** **:* * ***.*****:***.:*:: ..** *** *********:*:*::*:**:*

L_nogu_LEP1GSC059_2710 SVTWDHYKDRFPSEEPWYRRQDPFASDLKKTTFTNPKLGFVWKIFEKESWDVQFQANVSK

L_inte_LIC12374 SVTWDHYKDRFPSEEPWYRRQDPLSGDIKKTTFTNPKLGFVWKIFEKESWDVQFQANISK

L_kirs_LEP1GSC049_1720 SVTWDHYKDRFPSEEPWYRRQDPLASDLKKTTFTNPKLGFVWKVFEKESWDVQFQANASR

L_sant_LEP1GSC048_0703 SVTWDHYKDRFPSEEPWYRKQDPFAGDRKKTAFTNPKFGFVWKLFEKETWDIQFQANVSK

L_alex_LEP1GSC062_4342 SVTWDHYKDRFPSEEPWYRRQDPLAGDQKKTTFTNPKLGFVWKLFEKETWDIQFQANVSK

L_weil_LEP1GSC086_0503 SVTWDHYKDRFPSEEPWYRRQDPLAGDQKKTTFTNPKFGFVWKLFEKETWDIQFQANVSK

L_borg_LEP1GSC103_1969 SVTWDHYRDRFPSEEPWYRRQDLFASDSKKITFTNPKLGFVWKLFEREAWDVQFQANVSR

L_mayo_LEP1GSC190_2376 SVTWDHYKDRFPSEEPWYRRQDPLAGDRKKTTFTNPKFGFVWKLFEKETWDIQFQANVSK

L_alst_LEP1GSC193_0115 SVTWDHYKDRFPSEEPWYRRQDPLAGDQKKTTFTNPKFGFVWKLFEKENWDVQFQANVSK

L_kmet_LEP1GSC052_2138 SVTWDHYKDRFPSEEPWYRRQDPLASDQKKTAFTNPKFGFVWKLFEKETWDVQFQANVSK

*******.***********.** ::.* ** :*****:*****:**.* **:***** *.

L_nogu_LEP1GSC059_2710 QYRIPSFLEMFGEQGSIVANPNLKPEQSENGDGGFIYKTNHSFLKTKTSISYFTKDMRDM

L_inte_LIC12374 QYRIPSFLEMFGEQGSIVANPNLKPEQSENGDAGFIYKTNHSFLKTKSSISYFKKDMRDM

L_kirs_LEP1GSC049_1720 QYRIPSFLEMFGEQGSIVANPNLKPEQSENGDAGFIYKTNHSFLKTKTSVSCFKKDMRDM

L_sant_LEP1GSC048_0703 QYRIPSFLETFGEQGSIIANPNLRPERSGNGDVGFVFKTNHSHLKTKTSVSYFSKDIKDM

L_alex_LEP1GSC062_4342 QYRIPSFLEMFGEQGSIIANPNLRPERSGNGDAGFVFKTNHSYLKTKTSVSYFSKDIKDM

L_weil_LEP1GSC086_0503 QYRIPSFLEMFGEQGSIIANPNLRPEKSGNGDAGFVFKTNHSHLKTKTSVSYFSKDIKDM

L_borg_LEP1GSC103_1969 QYRIPSFLEMFGEQGSIIANPNLRPERSGNGDAGFVFKTNHSYLKTKTSVSYFSKDIKDM

L_mayo_LEP1GSC190_2376 QYRIPSFLEMFGEQGSIIANPNLRPERSGNGDAGFVFKTNHSYLKTKTSVSYFSKDIKDM

L_alst_LEP1GSC193_0115 QYRIPSFLEMFGEQGSIIANPNLKPEQSGNGDGGIVFKTDHSFLKTKTSVSYFSKDIKDM

L_kmet_LEP1GSC052_2138 QYRIPSFLEMFGEQGSIIANPNLKPEQSGNGDAGFVLKSDHSFLKTKTSVSYFSKDIKDM

********* *******:*****.**.* *** *:: *::**.****:*:* *.**:.**

L_nogu_LEP1GSC059_2710 ILFLPNSQFTLRPENVDSAKIRGLEFSHREDWKYGIKFLFNYTYQEAINTSSSPYLHGKI

L_inte_LIC12374 ILFLPNSQFTLRPENVDSAKIRGLEFSHRGDWKYGIKFLFNYTYQEAINTSSSPYLHGKI

L_kirs_LEP1GSC049_1720 ILFLPNSQFTLRPENVDSAKIRGLEFSHRGDWKYGIKFLFNYTYQEAINTSSSPYLHGKT

L_sant_LEP1GSC048_0703 ILFLPNSQFTLRPENVDSARIRGVEFSHREDWKYGIKFLFNYTYQDAINASNSGYLRGKI

L_alex_LEP1GSC062_4342 ILFLPNSQFTLRPENVDSARIRGVEFSHRIDWKYGIKFLFNYTYQDAINTSSSLYLRGKI

L_weil_LEP1GSC086_0503 ILFLPNSQFTLRPENVDSARIRGVEFSHRIDWKYGIKFLFNYTYQDAINTSSSVYLRGKI

L_borg_LEP1GSC103_1969 ILFLPNSQFTLRPENVDSARIRGIEFSHRVDWKYGIKFLFNYTYQDAINTSSSVYLRGKI

L_mayo_LEP1GSC190_2376 ILFLPNSQFTLRPENVDSARIRGVEFSHRVDWKYRIKFLFNYTYQDAINTSSSVYLHGKI

L_alst_LEP1GSC193_0115 ILFLPNSQFTLRPENVDSARIRGLEFSHREDWKFGFKFLFNYTYQEAINNSSSPYLNGKI

L_kmet_LEP1GSC052_2138 ILFLPNSQFTLRPENVDSARIRGLEFSHREDWKYGIKFLFNYTYQEAINTSSSPYLHGKI

*******************.***:***** ***: :*********:*** *.* **.**

L_nogu_LEP1GSC059_2710 LPLRPRHEFSSTFSWKGKKLETGIELLYIGAVFRDRTNEYINYIPERQIWNYFFTWVIDS

L_inte_LIC12374 LPLRPRHEFSSTFSWKGKKLETGIELLYIGAVFRDRTNEYINYIPERQIWNYFFTWVIYS

L_kirs_LEP1GSC049_1720 LPLRPRHEFSSTFSWKGKKLETGIELLYIGAVFRDRTNEYINYIPERQIWNYFFTWVLDS

L_sant_LEP1GSC048_0703 LPLRPRHEFASTLSWKGKRLETGIELLYIGAVFRDRTNEYVNYIPERQIWNCFLTLIL--

L_alex_LEP1GSC062_4342 LPLRSRHEFASTLSWKGKRLEIGIELLYIGAVFRDRTNEYINYIPERQIWNYFFTWVLYS

L_weil_LEP1GSC086_0503 LPLRSRHEFASTLSRKGKRSEIGIELLYIGAVFRDRTNEYVNYIPARQIWNYFFTWVLYS

L_borg_LEP1GSC103_1969 LPLRSRHEFASTLSWKRKRLETGIELLYIGAVFRDRTNEYINYIPERQIWNYFFTWILDS

L_mayo_LEP1GSC190_2376 LPLRSRHEFASTLSWKGKRLETGIELLYIGAVFRDRTNEYINYIPERQIWNYFFTWVLDS

L_alst_LEP1GSC193_0115 LPLRPRHEFSSTVSWRGKKLETGIELLYIGAVFRDRTNEYINYIPARQIWNYFFTWILYT

L_kmet_LEP1GSC052_2138 LPLRPRHEFSSTLSWKGKKLETGIELLYIGAVFRDRTNEYINYIPARQIWNYFFTWVLYS

****.****:**.*.. *. * ******************:**** ***** *:* ::

L_nogu_LEP1GSC059_2710 ELKDS--DVNANSKEI--TREILLTFEVKNFTDKRISDLIGYPLPGRSWYTTLSMRF

L_inte_LIC12374 ESKDS--DLNGNSKEI--TREVLLTFEVKNFTDKRISDLIGYPLPGRSWYTTLSMRF

L_kirs_LEP1GSC049_1720 ESKDS--DLNGNSKEI--TREVLLTFEVKNFTDKRISDLIGYPLPGRSWYTTLSMRF

L_sant_LEP1GSC048_0703 DPGEPIQDSPGNPKESVPSKEVLLTLEAKNFTDQRISDLIGYPLPGRSWYATLSMRF

L_alex_LEP1GSC062_4342 EPGEPLKDSLGNSKESAPAKEFLLTFEVKNFTDRRVSDLIGYPLPGRSWYVTLSMRF

L_weil_LEP1GSC086_0503 EPGEPVKDSLGNSKESAPAKEFLLTFEVKNFTDRRVSDLIGYPLPGRSWYATLSVRF

L_borg_LEP1GSC103_1969 EPGESVKNSLGDLKESAPSREVLLTFETKNFTDRRVSDLIGYPLPGRSWYVTLSMRF

L_mayo_LEP1GSC190_2376 EPGESVKNSLGDLKESVPSKEVLLTFEVKNFTDRRVADLIGYPLPGRSWYATLSVRF

L_alst_LEP1GSC193_0115 EPGEPVKDSLGNLKESAPAKEFLLTFEVKNFTDKKVSDLIGYPLPGRSWYATLSMRF

L_kmet_LEP1GSC052_2138 EPGEITKDALGNPKESAPSKEFLLTFEVKNFTDKRVSDLIGYPLPGRSWYATLSMRF

: : : .: ** :.*.***:*.*****..::*************.***:**
