# Supplementary material for: Discovery of Novel Leptospirosis Vaccine Candidates Using Reverse and Structural Vaccinology
Source: Front Immunol. 2017 Apr 27;8:463. doi: 10.3389/fimmu.2017.00463 (PMC5406399; doi:10.3389/fimmu.2017.00463)
Supplement: Supplementary file 8 [file Data_Sheet_1.ZIP › Alignment Bb-OMPs/Mult_alignment_LIC12575_path_spp_orthol_immun_epit_highlighted.docx]

L_kmet_LEP1GSC052_1941 MENKVKNSQTRIPKVREISRILLGSFVLLLSTT-LFAEGT-LLKLSTEETVKRALESNYN

L_nogu_LEP1GSC059_2939 MENKFKNWKQRILK---LSVFLLGNLILLSST--LFAEGS-TLKLTTEETVKRALESNYN

L_kirs_LEP1GSC049_0132 MENKFKNWKQRILK---LSLFLLGNLIFLSST--LFAEGS-TLKLTTEETVKRALESNYN

L_inte_LIC12575 MENKFKNWKQRILKFQRLSLFLLGNLILLSST--LFAESS-TLKLTTEETVKRALESNYN

L_alst_LEP1GSC193_3408 MENKIKNWKQRILKIQKLSGILLVNSVLLPFMT-LFADGSALLKLTTEETVKRALESNYN

L_sant_LEP1GSC048_0583 MEDKVQHQKQR-LRVQKLSRILLGNFVLLSTS--LFADNS-ILKLTTEETVKRALENNYN

L_mayo_LEP1GSC190_2420 MKDKVKNWKQR-LRIQKLSGVLLGNFVLLSTS--LFADNS-ILKLTTEETVKRALESNYN

L_alex_LEP1GSC062_0380 MKDKVKNWKQR-LRIQKLSRILLGNFVLLSTT--LFADDS-ILKLTTEETVKRALESNYN

L_borg_LEP1GSC103_2007 MKDKVKNWKQR-LRIQKLSLTLLGNFVLLSLSIPLFADDS-VLKLTTEETVKRALESNYN

L_weil_LEP1GSC086_0552 MKDKVKNWKQR-LRIRKLSRILLGNLVLLSTT--LFADDS-VLKLTTEETVKRALESNYN

*::*.:: : * . :* ** . ::* ***:.: ***:**********.***

L_kmet_LEP1GSC052_1941 LQNLRYELAKSDTNFLKNDSKYSWRLVADGRASQSILPFNQANLLSGTKISDDTIKGGIE

L_nogu_LEP1GSC059_2939 LQNLRYELAKSDTNFLKNDSKYSWRLIADGKSSQSILPFNQANFFTGTKISDDTIKGGIE

L_kirs_LEP1GSC049_0132 LQNLRYELAKSDTNFLKNDSKYSWRLVADGRSSQSILPFNQANFSTGTKISDDTIKGGIE

L_inte_LIC12575 LQNLRYELAKSDTNFLKNDSKYSWRLVADGRSSQSILPFNQANFFTGTKISDDTIKGGIE

L_alst_LEP1GSC193_3408 LQNLRYELAKSDTNFLKNDSKYSWRLVADGKSSQSILPFNQANFFTGTKISDDTIKGGIE

L_sant_LEP1GSC048_0583 LQNLRYELAKSDTNFLKNDSKYSWRLVADGRSSQSILPFNQTNIFTGTKISDDTIKGGIE

L_mayo_LEP1GSC190_2420 LQNLRYELAKSDTNFLKNDSKYSWRLVADGRSSQSILPFNQTNIFTGTKISDDTIKGGIE

L_alex_LEP1GSC062_0380 LQNLRYELAKSDTNFLKNDSKYSWKLVADGRSSQSILPFNQTNIFTGTKISDDTIKGGIE

L_borg_LEP1GSC103_2007 LQNLRYELAKSDTNFLKNDSKYSWRLVADGRSSQSILPFNQTNIFTGTKISDDTIKGGIE

L_weil_LEP1GSC086_0552 LQNLRYELAKSDTNFLKNDSKYSWRLVADGRSSQSILPFNQTNIFTGTKISDDTIKGGIE

************************.*:***.:*********:*: :**************

L_kmet_LEP1GSC052_1941 KTLQTTGTYFKVEAGTRRFDSNAFENAATTPAGFAALGIPPLYTGFVRATISQDLLKNSF

L_nogu_LEP1GSC059_2939 KILQTTGTYFKLEAGSRRFDSNAFENPSTTPAGFSSLGIPPLYTGFVRATISQDLLKNSF

L_kirs_LEP1GSC049_0132 KILQTTGTYFKVEAGSRRFDSNAFENPSTTPAGFSSLGIPPLYTGFVRATISQDLLKNSF

L_inte_LIC12575 KVLQTTGTYFKIEAGSRRFDSNAFENPSTTPAGFSSLGIPPLYTGFVRATISQDLLKNSF

L_alst_LEP1GSC193_3408 KIFQTTGTYFKVEAGNRRFDSNAFENPATTPAGFSALGIPPLYTGFVRATISQDLLKNSF

L_sant_LEP1GSC048_0583 KILQTTGTYFKMEAGTRRFDSNAFENPATTPAGFSALGIPPLYTGFVRATISQDLLKNSF

L_mayo_LEP1GSC190_2420 KILQTTGTYFKVEAGTRRFDSNAFENPATTPAGFSALGIPPLYTGFVRATISQDLLKNSF

L_alex_LEP1GSC062_0380 KILQTTGTYFKVEAGTRRFDSNAFENAATTPAGFSALGIPPLYTGFVRATISQDLLKNSF

L_borg_LEP1GSC103_2007 KILQTTGTYFKVEAGTRRFDSNAFENPATTPAGFSALGIPPLYTGFVRATISQDLLKNSF

L_weil_LEP1GSC086_0552 KILQTTGTYFKVEAGTRRFDSNAFENPATTPAGFSALGIPPLYTGFVRATISQDLLKNSF

* :********:***.**********.:******::************************

L_kmet_LEP1GSC052_1941 GYKGRNEVKILESQAEIAKNQVSQQISGVIVDSLVDFWDYSIKTQAVKTYRQLVENTKNI

L_nogu_LEP1GSC059_2939 GYKGRNEVKILESQAEIVKNQVSQQISSVIVDSLVDFWDYSIKTQAVKTYKQLVENTRNI

L_kirs_LEP1GSC049_0132 GYKGRNEVKILESQAEIVKNQVSQQISSVIVESLVDFWDYSIKTQAVKTYKQLVENTRNI

L_inte_LIC12575 GYKGRNEVKILESQAEIVKNQVSQQISSVIVESLVDFWDYSIKTQAVKTYKQLVENTRNI

L_alst_LEP1GSC193_3408 GYKGRNEVKILESQAEMMKNQVSQQISAVIVESLVDFWDYSIKTQAVKTYKQLVDNTKNI

L_sant_LEP1GSC048_0583 GYKGRNEIKILESQAEIAKNQVSQQISAVIVESLVDFWDYSIKTQAVKTYKQLVENTKNI

L_mayo_LEP1GSC190_2420 GYKGRNEVKILESQAEIVKNQVSQQISAVIVESLVDFWDYSIKTQAVKTYKQLVENTKNI

L_alex_LEP1GSC062_0380 GYKGRNEVKILESQTEMMKNQVSQQISAVIVESLVDFWDYSIKTQAVKTYKQLVENTKNI

L_borg_LEP1GSC103_2007 GYKGKNEVKILESQAEIVKNQVSQQISAVIVESLVDFWDYSIKTQAVKTYKQLVENTKNI

L_weil_LEP1GSC086_0552 GYKGKNEVKILESQAEIVKNQVSQQISAVIVESLVDFWDYSIKTQAVKTYKQLVENTKNI

****.**:******:*: *********.***:******************.***:**.**

L_kmet_LEP1GSC052_1941 RNLTARKQGLGLSESFEVNQWNALLAQAENQLETAQVQKEEAKRKLVRSLKIPDGTTLSE

L_nogu_LEP1GSC059_2939 RNLTARKQSLGLSESFEVNQWNALLAQAQNQLETAQVQKEESKRKLIRSLKIPDDTTLSE

L_kirs_LEP1GSC049_0132 RNLTARKQSLGLSESFEVNQWNALLAQAQNQLETAQVQKEESKRKLIRSLKIPDDTTLSE

L_inte_LIC12575 RNLTARKQSLGLSESFEVNQWNALLAQAQNQLETAQVQKEESKRKLIRSLKIPDDTTLSE

L_alst_LEP1GSC193_3408 RNLTVRKQNLGLSESFEINQWNALLAQAENQLETAQVQKEESKRKLVRSLKIPDGTSLSE

L_sant_LEP1GSC048_0583 RNLTIRKQSLGLSESFEVNQWNALLAQAENQLETAQVQKEEAKRKLVRSLKIPDGTSLSE

L_mayo_LEP1GSC190_2420 RNLTIRKQNLGLSESFEVNQWNALLAQAENQLETAQVQKEESKRKLVRSLKIPDGTSLSE

L_alex_LEP1GSC062_0380 RNLTIRKQSLGLSESFEVNQWNALLAQAENQLETAQVQKEESKRKLVRSLKIPDGTSLSE

L_borg_LEP1GSC103_2007 RNLTIRKQSLGLSESFEVNQWNALLAQAENQLETAQVQKEESKRKLVRSLKIPDGTSLSE

L_weil_LEP1GSC086_0552 RNLTIRKQSLGLSESFEVNQWNALLAQAENQLETAQVQKEESKRKLVRSLKIPDGTSLSE

**** ***.********:**********:************:****:*******.*:***

L_kmet_LEP1GSC052_1941 ETNLLEELAEKPEYTKDLEYAYKHRADFLNALKQKEIAEAALKNANNDRLPTLTLSGTGA

L_nogu_LEP1GSC059_2939 ETNLLEELIEKPEYTKDLEYAYKHRADFLNALKQKEIAEAALKNANNDRLPTLTLSGTGA

L_kirs_LEP1GSC049_0132 ETNLLEELTEKPEYTKDLEYAYKHRADFLNALKQKEIAEAALKNANNDRLPTLTLSGTGA

L_inte_LIC12575 ETNLLEELTEKPEYTKDLEYAYKHRADFLNALKQKEIAEAALKNANNDRLPTLTLSGTGA

L_alst_LEP1GSC193_3408 ETNLLEELVEKPDYTKDLEYAYKHRADFLNALKQKEIAEAALKNANNDRLPTLTLSGTGA

L_sant_LEP1GSC048_0583 ETNLLEELIEKPDYIKDLEYAYKHRADFLNALKQKEIAEAALKNANNDRLPTLTISGTGA

L_mayo_LEP1GSC190_2420 ETNLLEELIEKPDYIKDLEYAYKHRADFLNTLKQKEIAEATLKNANNDRLPTLTISGTGA

L_alex_LEP1GSC062_0380 ETNLLEELIEKPDYIKDLEYAYKHRADFLNALKQKEIAEAALKNANNDRLPTLTLSGTGA

L_borg_LEP1GSC103_2007 ETNLLEELIERPNYIKDLEYAYKHRADFLNALKQKEIAEAALKNANNDRLPTLTISGTGA

L_weil_LEP1GSC086_0552 ETNLLEELLEKPDYIKDLEYAYKHRADFLNALKQKEIAEAALKNANNDRLPTLTISGTGA

******** *.*:* ***************:*********:*************:*****

L_kmet_LEP1GSC052_1941 SQAQNIVSPQENYVDSTHGITTAKYKEWTGQMNFVYPIADKGIYAGVRDANIGMRQATLR

L_nogu_LEP1GSC059_2939 SQAQNIISPQDNYNDANQGITTAKYKEWTGQVNFVYPIADKGIYAGVRDANIGMRQATLK

L_kirs_LEP1GSC049_0132 SQAQNIISPQDNYNDTNQGITTAKYKEWTGQVNFVYPIADKGIYAGVRDANIGMRQATLK

L_inte_LIC12575 SQAQNIISPQDNYNDTNQGITTAKYKEWTGQVNFVYPIADKGIYAGVRDANIGMRQATLK

L_alst_LEP1GSC193_3408 SQAQNVLSPQDNYSDSNQGITTAKYKEWTGQMNFVYPLADKGIYAGVRDANIGMRQATLK

L_sant_LEP1GSC048_0583 SQAQNIISPQENYTNSNQGITTAKYKEWTGQMNFVYPLADKGIYAGVRDATIGMRQATLK

L_mayo_LEP1GSC190_2420 SQAQNIISPQENYINPNQGITTAKYKEWTGQMNFAYPLADKGIYAGVRDATIGIRQAILK

L_alex_LEP1GSC062_0380 SQAQNIISPQENYIDSNQGITTAKYKEWTGQMNFTYPLADKGIYAGVRDATIGMRQAILK

L_borg_LEP1GSC103_2007 SQAQNIISPQENYIDSNQGITTAKYKEWTGQMNFAYPLADKGIYAGVRDATIGMRQATLK

L_weil_LEP1GSC086_0552 SQAQNIISPQENYIDSNQGITTAKYKEWTGQMNFAYPLADKGIYAGVRDATIGMRQAILK

*****::***:** :..:*************:**.**:************.**:*** *.

L_kmet_LEP1GSC052_1941 EEELKNEVRDDVKTRIEALEASHRIYKNNIITERESNNYYNGVLRSFRQGRADAVSVKNA

L_nogu_LEP1GSC059_2939 EEELKNEVRDDVKTRIEALEASHRIYKNNIVTERETSNYYNGVLRSFRQGRADAVAVKNA

L_kirs_LEP1GSC049_0132 EDELKNEVRDDVKTRIEALEASHRIYKNNIITERETANYYNGVLRSFRQGRADAVAVKNA

L_inte_LIC12575 EEELKNEVRDDVKTRIEALEASHRIYKNNIVTERETANYYNGVLRSFRQGRADAVAVKNA

L_alst_LEP1GSC193_3408 EEELKNEVRDDVKTRIEALEASHRIYKNNITTERETQNYYNGVLRSFRQGRADAVSVKNA

L_sant_LEP1GSC048_0583 EEELKNEVRDDVKTRIEALEASHRIYKNNITTERETENYYNGVLRSFRQGRADAVSVKNA

L_mayo_LEP1GSC190_2420 EEELKNEVRDDVKTRIEALEASHRIYKNNITTERETENYYNGVLRSFRQGRADAVSVKNA

L_alex_LEP1GSC062_0380 EEELKNEVRDDVKTRIEALEASHRIYKNNITTERETENYYNGVLRSFRQGRADAVSVKNA

L_borg_LEP1GSC103_2007 EEELKNEVRDDVKTRIEALEASHRIYKNNITTERETQNYYNGVLRSFRQGRADAVSVKNA

L_weil_LEP1GSC086_0552 EEELKNEVRDDVKTRIEALEASHRIYKNNITTERETENYYNGVLRSFRQGRADAVSVKNA

*:**************************** ****: ******************:****

L_kmet_LEP1GSC052_1941 LDTYVQDQLRLTQAKVNFNIDLLRYYLAKNSLLERFQVERDKLLPNLD

L_nogu_LEP1GSC059_2939 LDTHVQDQLRLTQAKVNFNIDLLRYYLAKNALMERFQVDRDKLIPHLD

L_kirs_LEP1GSC049_0132 LDTHVQDQLRLTQAKVNFNIDLLRYYLSKNALMERFQVDRDKLIPHLD

L_inte_LIC12575 LDTHVQDQLRLTQAKVNFNIDLLRYYLAKNALMERFQVDRDKLIPHLD

L_alst_LEP1GSC193_3408 LDTHVQDQLRLTQARVNFNIDLLRYHLAKNALLERFQVDRDKLLPHLD

L_sant_LEP1GSC048_0583 LDTHVQDQLRLTQAKVNFNIDLLRYYLAKNALLEHFQVDRDKLLPHLN

L_mayo_LEP1GSC190_2420 LDTHVQDQLRLTQAKVNFNIDLLRYYLAKNALLEHFQVDRDKLLPHLN

L_alex_LEP1GSC062_0380 LDTHVQDQLRLTQAKVNFNIDLLRYYLAKNSLLEHFQVDRDKLLPHLN

L_borg_LEP1GSC103_2007 LDTHVQDQLRLTQAKVNFNIDLLRYYLAKNALLEHFQVDRDKLLPHLN

L_weil_LEP1GSC086_0552 LDTHVQDQLRLTQAKVNFNIDLLRYYLAKNALLEHFQVDRDKLLPHLN

***:**********.**********:*:**:*:*.***:****:*:*:
