# Supplementary material for: Discovery of Novel Leptospirosis Vaccine Candidates Using Reverse and Structural Vaccinology
Source: Front Immunol. 2017 Apr 27;8:463. doi: 10.3389/fimmu.2017.00463 (PMC5406399; doi:10.3389/fimmu.2017.00463)
Supplement: Supplementary file 8 [file Data_Sheet_1.ZIP › Alignment Bb-OMPs/Mult_alignment_LIC13477_path_spp_orthol_immun_epit_highlighted.docx]

L_sant_LEP1GSC048_2057 MFNDLLKKAYRFCYLTVILFPFLVFSQEVEEKTKLDFQGNYRVRGFNLARDIYTLRQTSA

L_borg_LEP1GSC103_2481 MFNNLSKKAYCFYYLAVILSPSLIFSQEVEEKTKFDFQGNYRVRGFNLARDIYTSRQTSA

L_mayo_LEP1GSC190_1628 MFNNLLKKAYGFYCLAMILFPSLVFSQEVEEKTKLDFQGNYRVRGFNLARDIYTSRQTSA

L_alex_LEP1GSC062_2390 MFNNLLKKAYGFYYLAVILFPSLIFSQEVEEKTKLDFQGNYRVRGFNLARDIYTSRQTSA

L_weil_LEP1GSC086_2488 MFNNLLKKAYGFYYLAVILFPSLIFSQEAEEKTKLDFQGNYRVRGFNLARDIYTSRQTSA

L_kmet_LEP1GSC052_3542 MSIDLLKKAYRLYYLALILFPSLLFSQEIEEKTKLDFQGNYRVRGFNLGRDIYTSRQTAA

L_alst_LEP1GSC193_2428 MSNDLSKKAYHFYYLAMILFPSLLFSQEIEEKTKLDFQGNYRVRGFNLGRDIYTSRQTAA

L_kirs_LEP1GSC049_2731 MSENLLKKAYLF---VLVLFPSLLFSQEIEEKTKLDFQGNYRVRGFNLGRDIYTSRQTAA

L_inte_LIC13477 MSDNLLKKAYLFYFLVLILFPSLLFSQEIEEKTKLDFQGNYRVRGFNLGRDIYTSRQTAA

L_nogu_LEP1GSC059_3810 MSDNLLKKAYLFYFLAIILFPSLLFSQEIEEKTKLDFQGNYRVRGFNLGRDIYTSRQTAA

* :* **** : .::* * *:**** *****:*************.***** ***:*

L_sant_LEP1GSC048_2057 TPYDKNAFKTEQQQKNQNIAETEFAERLKGNPSTLSPQKEDISYFDTRMTVNMNFSTSKY

L_borg_LEP1GSC103_2481 TPYDKGAFKTEQQQRNQNIAETEITEKLKGNPSTLSPQKEDISYFDTRMTVNMNFNTSKY

L_mayo_LEP1GSC190_1628 TPYDKDAFKTEQQQRNQNIAETEITEKLKGNPSTLSPQKEDISYFDTRMTINMNFSTSKY

L_alex_LEP1GSC062_2390 TPYDKDAFKTEQQQRNQNIAETEITERLKGNPSTLSPQKEDISYFDTRMTINMNFSTSKY

L_weil_LEP1GSC086_2488 TPYDKEAFKTEQQQRNQNIAETEITERLKGNPSTLSPQKEDISYFDTRMTINMNFNTSKY

L_kmet_LEP1GSC052_3542 TPYDKNAFKADQQQQNQAVADNEIAERLKGNPTTLSPQKEDISYFDTRMTVNMNFNTSKY

L_alst_LEP1GSC193_2428 TPYDKNAFKTEEQQKKQTAAETEIAERLKGNPSTLSPQKEDISYFDTRMTVNMNFNTSKY

L_kirs_LEP1GSC049_2731 TPYDRNAFKTEEEQKNQAAADTEIAERLKGNPSTLSPRKEDISYFDTRMTVNMNFNTSKY

L_inte_LIC13477 TPYDKNTFKTEEEQRNQAAVDTEIAERLKGNPSTLSPRKEDISYFDTRMTVNMNFNTSKY

L_nogu_LEP1GSC059_3810 TPYDKNVFKTEEEQRNQTAVDTEIAERLKGNPTTLSPRKEDISYFDTRMTVNMNFNTSKY

****. .**::::*.:* .:.*::*.*****:****.************:****.****

L_sant_LEP1GSC048_2057 FEALWGVQVGDITFGGKGFGQNSTTGPGQGGEANSTSPVNIQTTFLYLNFKLPEDAFSLR

L_borg_LEP1GSC103_2481 FEALWGVQVGDITFGGKGFGQNSTTGPGQGGEANSTSPVNIQTTFLYLNFKLPEDAFSLR

L_mayo_LEP1GSC190_1628 FEALWGVQVGDITFGGKGFGQNSTTGPGQGGEANSTSPVNIQTTFLYLNFKLPEDAFSLR

L_alex_LEP1GSC062_2390 FEALWGVQVGDITFGGKGFGQNSTTGPGQGGEANSTSPVNIQTTFLYLNFKLPEDAFSLR

L_weil_LEP1GSC086_2488 FEALWGVQVGDITFGGKGFGQNSTTGPGQGGEANSTSPVNIQTTFLYLNFKLPEDAFSLR

L_kmet_LEP1GSC052_3542 FEALWGVQVGDITFGGKGFGQNSTTGPGQGGEAGFTSPVNIQTTFLYLNFKLPEDAFSLR

L_alst_LEP1GSC193_2428 FEALWGVQVGDITFGGKGFGQNSTTGPGQGGEAGFTSPVNIQTTFLYLNFKLPEDAFSLR

L_kirs_LEP1GSC049_2731 FEALWGVQVGDITFGGKGFGQNSTTGPGQGGEAGFTSPVNIQTTFLYLNFKLPEDAFSLR

L_inte_LIC13477 FEALWGVQVGDITFGGKGFGQNSTTGPGQGGEAGFTSPVNIQTTFLYLNFKLPEDAFSLR

L_nogu_LEP1GSC059_3810 FEALWGVQVGDITFGGKGFGQNSTTGPGQGGEAGFTSPVNIQTTFLYLNFKLPEDAFSLR

*********************************. *************************

L_sant_LEP1GSC048_2057 VGQQLFFSTRGRVIFASGTGITINKDFRLWNTTIESGWFVARQNAQLDLDKNTYADKNYV

L_borg_LEP1GSC103_2481 VGQQLFFSPRGRVIFASGTGITINKDFRLWNTTIESGWFVARQNAQLDLDKNAYADKNYV

L_mayo_LEP1GSC190_1628 VGQQLFFSPRGRVIFASGTGITINKDFRLWNTTIESGWFVARQNAQLDLDKNSYADKNYV

L_alex_LEP1GSC062_2390 VGQQLFFSPRGRVIFASGTGITINKDFRLWNTTIESGWFVARQNAQLDLDKNSYADKNYV

L_weil_LEP1GSC086_2488 VGQQLFFSPRGRVIFASGTGITINKDFRLWNTTIESGWFVARQNAQLDLDKNSYADKNYV

L_kmet_LEP1GSC052_3542 VGQQLFSSARGRVVFTPGTGVTLNKDFRLWNTTIEAGWFVARQNAQLDLDKNTYADKNYI

L_alst_LEP1GSC193_2428 VGQQLFSSPRGRVIFSPGTGVTVNKDFRLWNTTIEAGWFVARQNAQLDLDKNTYADRNYI

L_kirs_LEP1GSC049_2731 VGQQLFSSPRGRVVLTPGTGVTVNKDFRLWNTTIEAGWFVARQNAQLDLDKNTYADKNYV

L_inte_LIC13477 VGQQLFSSPRGRVVFTPGTGVTVNKDFRLWNTTIEAGWFVARQNAQLDLDKNTYADKNYV

L_nogu_LEP1GSC059_3810 VGQQLFSSPRGRVVFTPGTGVTVNKDFRLWNTTIEAGWFVARQNAQLDLDKNTYADKNYV

****** *.****:::.***:*:************:****************:***.**:

L_sant_LEP1GSC048_2057 GTNIYFYKVKTSFLNNVKHELYSYFLDDTIKSIEKTTNAKGTVIALDSEFGGLFWHGFMN

L_borg_LEP1GSC103_2481 GTNIYFYRIKTSFFNNVKHELYSYFLDDTIKSIEKTTNAKGTVIALDSEFGQLFWHGFMN

L_mayo_LEP1GSC190_1628 GTNIYFYKVKTSFFNNVKHELYSYFLDDTIKSIEKTTNARGTVIALDSEFGQLFWHGFMN

L_alex_LEP1GSC062_2390 GTNIYFYKVKTSFFNNVKHELYSYFSDDTIKSIDKTTNAKGTVIALDSEFGQLFWHGFMN

L_weil_LEP1GSC086_2488 GTNIYFYKVKTSFFNNVKHELYSYFSDDTIKSIDKTTNAKGTVIALDSEFGQLFWHGFMN

L_kmet_LEP1GSC052_3542 GTNIYFYEIKTSFFNNVKHELYSYFLDDTIASIDKTTNAKGNVIALDSEVGQLFWHGFMN

L_alst_LEP1GSC193_2428 GTNIYFYEIKTSFFNNVKHELYSYFLDDTIKSIDKTTNANGTVIALDSEVGQLFWHGFMN

L_kirs_LEP1GSC049_2731 GTNIYFYEIKTSFLNNVKHELYSYFLDDTIKSIDKATNAKGTVIALDSEVGQLFWHGLMN

L_inte_LIC13477 GTNIYFYEIKTSFLNNIKHELYSYFLDDTIKSIDKTTNVRGAVIALDSEVGQLFWHGLMN

L_nogu_LEP1GSC059_3810 GTNIYFYEIKTSFLNNIKHELYSYFLDDTIKSIDKTTNAKGTVIALDSEVGQLFWHGLMN

******* :****:**:******** **** **:*:**..* *******.* *****:**

L_sant_LEP1GSC048_2057 EINLSNFGFVIHGIYNHGTVHVLEPYRDNAGNVLYNRFNKHNISGGMVDLQFSYRYSENF

L_borg_LEP1GSC103_2481 EINLSNFGFVVHGIYNHGTVHVLEPYRDNTGNVLYNRFSRHNISGGMLDLQFSYRYSENF

L_mayo_LEP1GSC190_1628 EINLSNFGFVVHGIYNHGTVHVLEPYRDNAGNVLYNQFSKHNISGGMADLQFSYRYSENL

L_alex_LEP1GSC062_2390 EINLSNFGFVVHGIYNHGAVHVLEPYRDNAGNVLYNQFSRHNISGGMVDLQFSYRYSENF

L_weil_LEP1GSC086_2488 EINLSNFGFVVHGIYNHGTVHVLEPYRDNAGNVLYNQFSRHNISGGMADLQFSYRYSENF

L_kmet_LEP1GSC052_3542 EINLSNFGFVVHGIYNHGTVHSLAPYRDAAGNVLYNRFSKHNISGGMADLQFSYRYSENL

L_alst_LEP1GSC193_2428 EINLSNFGFVIHGIYNHGTVRSLDPYRDEIGTVLYNRYSRHSISGGMADLQFSYRYSENL

L_kirs_LEP1GSC049_2731 EINLSNFGFVIHGIYNHGTVRTLDPYRDEIGNVLYNRYSKHTISGGMVDLQFSYRYSENL

L_inte_LIC13477 EINLSNFGFVIHGIYNHGTVQTLDPYRDGIGNILYNRYNKHTISGGMVDLQFSYRYSENL

L_nogu_LEP1GSC059_3810 EINLSNFGFVIHGIYNHGTVRTLDPYRDDIGNVLYNRYSKHTISGGMVDLQFSYRYSENL

**********:*******:*. * **** *.:***.:..*.***** ***********:

L_sant_LEP1GSC048_2057 TFNLVGVGTTGRPGFDKDGTKANLRGGGYKTLMPGYSISNIGNDFTGGYALFSGKDSSGL

L_borg_LEP1GSC103_2481 TFNLIGVGTTGRPGFDKDGTRANLRGGGYKTLMPGYSISNIGHDFTGGYALFSGKDSSGL

L_mayo_LEP1GSC190_1628 TFNLIGVGTTGRPGFDKDGTKANLRGGGYKTLMPGYSISNIGHDFTGGYALFSGKDSSGL

L_alex_LEP1GSC062_2390 TFNLIGVGTTGRPGFDKDGTKANLRGGGYKTLIPGYSISNIGHDFTGGYALFSGKDSSGL

L_weil_LEP1GSC086_2488 TFNLIGVGTTGRPGFDKDGTKANLRGGGYKTLMPGYSISNIGHDFTGGYALFSGKDSSGL

L_kmet_LEP1GSC052_3542 TFNLVGVGSTGRAGFDKDGTKANLRGGGYKTLMPGYSISNIANDFTGGYALFSGKDSSGL

L_alst_LEP1GSC193_2428 TFNLVGVGSTGRPGFDKDGTKANLRAGGYKTLMPGYSISNIANDFTGGYALFSGKDSSGL

L_kirs_LEP1GSC049_2731 TFNLVGVGSTGRPGFDKDGNRANLKGGGYKTLMPGYSISNIANDFTGGYALFSGKDSSGL

L_inte_LIC13477 TFNLVGVGSTGRPGFDKDGNRANLKGGGYKTLMPGYSISNIANDFTGGYALFSGKDSSGL

L_nogu_LEP1GSC059_3810 TFNLVGVGSTGRPGFDKDGNRANLKAGGYKTLMPGYSISNIANDFTGGYALFSGKDSSGL

****:***:***.******..***..******:********.:*****************

L_sant_LEP1GSC048_2057 YEYGINGDFVVYGPLLLTLGYYRLYGTKSPYIENNRYFNFDNGYKTSTFFGHEVNINLRW

L_borg_LEP1GSC103_2481 YEYGVNGDFVVYGPLLLTLGYYRLYGTKSPYIENNRYFNFDNGYKTSTFFGHEVNINLRW

L_mayo_LEP1GSC190_1628 YEYGVNGDFVVYGPLLLTLGYYRLYGTKSPYVENNRYFNFDNGYRTNTFFGHEVNINLRW

L_alex_LEP1GSC062_2390 YEYGVNGDFVVYGPLLLTLGYYRLYGTKSPYIENNRYFNFDNGYKTSTFFGHEVNINLRW

L_weil_LEP1GSC086_2488 YEYGVNGDFVIYGPLLLTLGYYRLYGTKSPYIENNRYFNFDSGYKTSAFFGHEVNINLRW

L_kmet_LEP1GSC052_3542 YEYGINGDFVVYGPLLLTLGYYRLYGTKSPYIENNRYFNFENGYKTSAFFGHEININLRW

L_alst_LEP1GSC193_2428 YEYGINGDFVVYGPLLLTLGYYRLYGTKSPFIENNRYFNFDNGYKTSAFFGHEVNINLRW

L_kirs_LEP1GSC049_2731 YEYGINSDFVVYGPLLLTLGYYRLYGTKSPFIENNRYFNYENGYKTSAFFGHEVNVNLRW

L_inte_LIC13477 YEYGINGDFVVYGPLLLTLGYYRLYGTKSPFIENNRYFNYENGYKTSAFFGHEVNVNLRW

L_nogu_LEP1GSC059_3810 YEYGINGDFVVYGPLLLTLGYYRLYGTKSPFIENNRYFNYENGYKTNAFFGHEVNINLRW

****:*.***:*******************::*******::.**.*.:*****:*:****

L_sant_LEP1GSC048_2057 NAFRDMQILMRSGYFIAGDGLKAYLDTTYGKILREFFVTAEHRF

L_borg_LEP1GSC103_2481 NAFRDMQILMRSGYFIAGDGLKAYLDTTYGKILREFFVTAEHRF

L_mayo_LEP1GSC190_1628 NAFRDMQILMRSGYFVAGDGLKAYLDTTYGKILREFFVTAEHRF

L_alex_LEP1GSC062_2390 NAFRDMQILMRSGYFIAGDGLKAYLDTTYGKILREFFVTAEHRF

L_weil_LEP1GSC086_2488 NAFRDMQILMRSGYFIAGDGLKAYLDTTYGKILREFFVTAEHRF

L_kmet_LEP1GSC052_3542 NAFRDMQILMRSGYFVAGDGLKAYLDTTQGKILREFFVTAEHRF

L_alst_LEP1GSC193_2428 SAFRDMQILMRSGYFVAGDGLKAYLDTTQGKILREFFVTAEHRF

L_kirs_LEP1GSC049_2731 NAFRDMQILMRSGYFVAGDGLKAYLDTTQGKILREFFVTAEHRF

L_inte_LIC13477 NAFRDMQILMRSGYFVAGDGLKAYLDTTQGKILREFFVTAEHRF

L_nogu_LEP1GSC059_3810 NAFRDMQILMRSGYFVAGDGLKAYLDTTHGKILREFFVTAEHRF

.**************:************ ***************
