# Supplementary material for: Discovery of Novel Leptospirosis Vaccine Candidates Using Reverse and Structural Vaccinology
Source: Front Immunol. 2017 Apr 27;8:463. doi: 10.3389/fimmu.2017.00463 (PMC5406399; doi:10.3389/fimmu.2017.00463)
Supplement: Supplementary file 8 [file Data_Sheet_1.ZIP › Alignment Bb-OMPs/Mult_alignment_LIC20019_path_spp_orthol_immun_epit_highlighted.docx]

L_kmet_LEP1GSC052_0460 MNKKKHLVNTILVFIILNSSLLRSIQAQTLNAGEPDFLKPHVEKEKPIAEEETFLKKLIR

L_nogu_LEP1GSC059_1024 MKIKQIP---LLILVLHLFS-FAPIYSQITIPNSDEITKPFPEKEKPITNEETFFKKLLR

L_inte_LIC20019 MKIKQTS---FLILVLHFFS-FVSIYSQAAVTNPEET-KPFSTKEKPVPAEETFFKKLIQ

L_kirs_LEP1GSC049_3085 MKIKQTS---FLILALHFFS-FSPIYSQISTSNSDETTKPFSTKEKPIIAEETFFKKLIR

L_alst_LEP1GSC193_1569 MKIKKLSAKNLAILSIYLLS-IGPAYTQVALPSPEEPPKPVQEKEKQIP-EETFFKKLIR

L_sant_LEP1GSC048_1364 MKIQKKTLKTFLIFILPLLQ-IGSIYAQASPPISEESAKSFQ--EKPIVTEESFFKKLLQ

L_weil_LEP1GSC086_1907 MKIQKHTLKIFLILIGPFLQ-IGSIYAQTS---SGEPPKSFQEKEKQAVPEETFLKKLLQ

L_alex_LEP1GSC062_4441 MKIQKHTLKIFLILIAPLLQ-IGSIYAQTPLPISEESPKSSQEKEKQTLPEETFFKKLLR

L_borg_LEP1GSC103_0776 MKIQKHILKISLIFITPLLQ-IGSIYAQASLPIPEESPKSFQEKEKQAATEETFFKKLLQ

L_mayo_LEP1GSC190_1280 MKIQKHILKISLILITPLLQ-IGSIYAQASLPISEESPKTFQEKEKQSTAEETFLKKLLQ

*: :: :: . : . :* : *. ** **:*:***:.

L_kmet_LEP1GSC052_0460 QSSFTILAGRNGGDNIFETGTKYANLSGLRGGSRITYERDFNYGGLGFTLRWKKWEADLN

L_nogu_LEP1GSC059_1024 QSSFTILAGRNGGDNIFETGTKYPNLSGLKGGSRITYARDFNYAGLGFTLRWEKWEADLN

L_inte_LIC20019 QSSFTILAGRNGGDNIFETGTKYPNLSGLKGGSRITYARDFNYAGLGFTLRWEKWEADLN

L_kirs_LEP1GSC049_3085 QSSFTILAGRNGGDNIFETGTKYPNLSGLKGGSRITYARDFNYAGLGFTLRWEKWEADLN

L_alst_LEP1GSC193_1569 QSSFTILAGRNGGDNIFETGTKYPNLSGLRGGSRITYARDFNYGGLGFTLRWQKWEADLN

L_sant_LEP1GSC048_1364 QSSFTILAGRNGGDNIFETGTKYPNLSGLKGGSRITYARDFNYGGLGFTLRWQKWEADLN

L_weil_LEP1GSC086_1907 QSSLTILAGRNGGDNIFETGTKYPNLSGLRGGSRITYARDFNYGGLGFTLRWQKWEADLN

L_alex_LEP1GSC062_4441 QSSLTILAGRNGGDNIFETGTKYPNLSGLRGGSRITYARDFNYGGLGFTFRWQKWEADLN

L_borg_LEP1GSC103_0776 QSSFTILAGRNGGDNIFETGTKYPNLSGLRGGSRITYARDFNYGGLGFTLRWQKWEADLN

L_mayo_LEP1GSC190_1280 QSSLTILAGRNGGDNIFETGTKYPNLSGLRGGSRITYARDFNYGGLGFTLRWQKWEADLN

***:*******************.*****.******* *****.*****:**:*******

L_kmet_LEP1GSC052_0460 VKTTGRYVNAGEGRDEDFYLGDPTVERGTKISTREFSYYDTPYTFIGSRNFADGKGRLSM

L_nogu_LEP1GSC059_1024 IKTTGRYVNAGEGRDEDFFLGDPTVERGTKISTREFSYYDTPYTFIGSRNFADGRGRLSM

L_inte_LIC20019 LKTTGRYVNAGEGRDEDFFLGDPTVERGTKISTREFSYYDTPYTFIGSRNFADGKGRLSM

L_kirs_LEP1GSC049_3085 LKTTGRYVNAGEGRDEDFFLGDPTVERGTKISTREFSYYDTPYTFIGSRNFADGKGRLSM

L_alst_LEP1GSC193_1569 LKTTGRYVNAGEGRDEDFFLGAPTIERGTKISTREFTFYDTPYTFIGSRNFADGKGRLSM

L_sant_LEP1GSC048_1364 LKTTGRYVNAGEGRDEDFFLGDPTVERGTKISTRELSYYDTPYTFIGSRNFADGKGRLSM

L_weil_LEP1GSC086_1907 LKTTGRYINAGEGRDEDFFLGDPTVERGTKISTRELSYYDTPYTFIGSRNFADGKGRLSM

L_alex_LEP1GSC062_4441 LKTTGRYVNAGEGRDEDFFLGDPTVERGTKISTRELSYYDTPYTFIGSRNFADGKGRLSM

L_borg_LEP1GSC103_0776 LKTTGRYVNAGEGRDEDFFLGDPTVERGTKISTRELSYYDTPYTFIGSRNFADGKGRLSM

L_mayo_LEP1GSC190_1280 LKTTGRYVNAGEGRDEDFFLGDPTIERGTKISTRELSYYDTPYTFIGSRNFADGKGRLSM

:******:**********:** **:**********:::****************.*****

L_kmet_LEP1GSC052_0460 KQDRQSLILRRYFGDGDPDSRKEGKGLYLTGGFQYTFMKYVLYDVFQFFDSNPVFLNRIG

L_nogu_LEP1GSC059_1024 KNNSQSLILRRYFGDGEADYRKEGKGFYLTGGFQYTFMKYILYDVFQFFDSSPVFLNRIG

L_inte_LIC20019 KNNSQSLILRRYFGDGEADYRKEGKGFYLTGGFQYTFMKYILYDVFQFFDSSPVFLNRIG

L_kirs_LEP1GSC049_3085 KNNSQSLILRRYFGDGEADYRKEGKGFYLTGGFQYTFMKYILYDVFQFFDSSPVFLNRIG

L_alst_LEP1GSC193_1569 KQQRQSLILRRYFGDTDPDFRKEGKGLFLTGGFQYTFMKYVLYDVFQFFDSNPIFLNRIG

L_sant_LEP1GSC048_1364 MQNRQSLVLRRYFGNGDSDFRKEGKGLYLTGGFQYTFMKYVLYDVFQFFDSNPVFLNRIG

L_weil_LEP1GSC086_1907 IQHRQSLILRRYFGDSDPDFRKEGKGVYLTGGFQYTFMKYVLYDVFQFFDSNPIFLNRIG

L_alex_LEP1GSC062_4441 IQNRQSLILRRYFGDSDSDFRKEGKGIYLTGGFQYTFMKYVLYDVFQFFDSNPIFLNRIG

L_borg_LEP1GSC103_0776 IQNRQSLILRRYFGDSDSDFRKEGKGMYLTGGFQYTFMKYVLYDVFQFFDSNPVFLNRIG

L_mayo_LEP1GSC190_1280 IQNRQSLILRRYFGDSDSDFRKEGKGIYLTGGFQYTFMKYVLYDVFQFFDSNPIFLNRIG

: ***:******: :.* ******.:************:**********.*:******

L_kmet_LEP1GSC052_0460 LGLSLSYSTYEFPLGLGYRYSNGEWIWETSLSGIFWSGHFRDFHYQRALNFIGDVSGFGV

L_nogu_LEP1GSC059_1024 LGLSFSYSTYEFPLGLGYRYSNGEWVFETSFSGIFWTGHFRDFHYQRALNFIGDVSGFGI

L_inte_LIC20019 LGLSFSYSTYEFPLGLGYRYSNGEWVFETSFSGIFWTGHFRDFHYQRALNFIGDVSGFGI

L_kirs_LEP1GSC049_3085 LGLSFSYSTYEFPLGLGYRYSNGEWVFETSFSGIFWTGHFRDFHYQRALNFIGDVSGFGI

L_alst_LEP1GSC193_1569 SGLSLSYSTYEFPLGLGYRYSNGEWFFETSFSGIFWTGHFRDFHYQRALNFIGDVSGFGI

L_sant_LEP1GSC048_1364 LGLSLSYSTYEFPLGLGYRYSNGEWLFETSFSGIFWTGHFRDFHYQRTLNFIGDLSGFGI

L_weil_LEP1GSC086_1907 LGLSLSYSTYEFPLGLGYRYSNKEWLFETSLSGIFWTGHFRDFHYQRSLNFIGDLSGFGI

L_alex_LEP1GSC062_4441 LGLSLSYSTYEFPLGLGYRYSNKEWLFETSLSGIFWTGHFRDFHYQRSLNFIGDLSGFGI

L_borg_LEP1GSC103_0776 LGLSLSYSTYEFPLGLGYRYSNKEWLFETSLSGIFWTGHFRDFHYQRSLNFIGDLSGFGI

L_mayo_LEP1GSC190_1280 LGLSLSYSTYEFPLGLGYRYSNKEWLFETSLSGIFWTGHFRDFHYQRSLNFIGDLSGFGI

***:***************** **.:***:*****:**********:******:****:

L_kmet_LEP1GSC052_0460 DFNMGAGRIFGNYVLFLKLNEHRLFGDGHFSTKGGLSYNDILSQHLGQYKNYMNLKEWNV

L_nogu_LEP1GSC059_1024 DFNIGAGKIFGNYLMFLKLNEHRLFGDGHFFTKGGLSENDILSQHLGHYKNYMNLKEWNV

L_inte_LIC20019 DFNIGAGKIFGNYLMFLKLNEHRLFGDGHFVTKGGLSESDILSQHLGHYKNYMNLKEWNV

L_kirs_LEP1GSC049_3085 DFNIGAGKIFGNYLMFLKLNEHRLFGDGHFVTKGGLSESDILSQHLGHYKNYMNLKEWNI

L_alst_LEP1GSC193_1569 DFNASAGRIFGNYLVFIKLNEHRLFGDGHFATKGGLNESDILSQHLGHYKNYMNLKEWNV

L_sant_LEP1GSC048_1364 DFNLGVGRIFGNYLVFLKLNEHRLFGDGHFSTKGGLNNNDILSQYLGHYRNYMNLKEWNV

L_weil_LEP1GSC086_1907 DFNLGVGKIFGNYLAFLKLNEHRLFGDGHFSTKGGLNNNDILSQYFGHYKNYMNLKEWNV

L_alex_LEP1GSC062_4441 DFNLGVGKIFGNYLAFLKLNEHRLFGDGHFSTKGGLNNNDILSQYFGHYKNYMNLKEWNI

L_borg_LEP1GSC103_0776 DFNLGVGRIFGNYLTFLKLNEHRLFGDGHFSTKGGLNNNDILSQYLGHYKNYMNLKEWNV

L_mayo_LEP1GSC190_1280 DFNLGVGKIFGNYLAFLKLNEHRLFGDGHFSTKGGLNNNDILSQYFGHYKNYMNLKEWNV

*** ..*.*****: *:************* *****. .*****::*:*.*********:

L_kmet_LEP1GSC052_0460 ELSLTGFLY

L_nogu_LEP1GSC059_1024 ELSLTGFLY

L_inte_LIC20019 ELSLTGFLY

L_kirs_LEP1GSC049_3085 ELSLTGFLY

L_alst_LEP1GSC193_1569 ELSLTGFLY

L_sant_LEP1GSC048_1364 ELSLTGFLY

L_weil_LEP1GSC086_1907 ELSLTGFLY

L_alex_LEP1GSC062_4441 ELSLTGFLY

L_borg_LEP1GSC103_0776 ELSLTGFLY

L_mayo_LEP1GSC190_1280 ELSLTGFLY

*********
