# Supplementary material for: Discovery of Novel Leptospirosis Vaccine Candidates Using Reverse and Structural Vaccinology
Source: Front Immunol. 2017 Apr 27;8:463. doi: 10.3389/fimmu.2017.00463 (PMC5406399; doi:10.3389/fimmu.2017.00463)
Supplement: Supplementary file 8 [file Data_Sheet_1.ZIP › Alignment Bb-OMPs/Mult_alignment_LIC20151_path_spp_orthol_immun_epit_highlighted.docx]

L_alst_LEP1GSC193_1731 -----------MPFL-IRRSFQFKS--TLLLILFCIPIQAQEEKTKTETEVAEKRSPNIF

L_kmet_LEP1GSC052_0212 -----------MYFS-LYRSFQLKTIQILFLILFCVPIQAQEEKTKTEIKTGGNESPNTN

L_nogu_LEP1GSC059_1080 METNPVLNKLNMYFR-LSRFFSSNWIQILFLFLFYVSIQAQDENPKTENKVAPKESPNTI

L_inte_LIC20151 METNPILNQLNMFFS-FRRFFSSNWIQILFLFLFYVSIQAQDENIKTENKVIQKESPNTT

L_kirs_LEP1GSC049_3174 METNTILNKLNMFFPHLSRSFSF----VLFLFLFYVSIQAQDENTKIENKVTQKKSPNTI

L_sant_LEP1GSC048_0186 -----------MRFS-IRRFFQLKSIRILFLIFFCISVRAQDESTKAENKVAKEKSPNKI

L_borg_LEP1GSC103_0620 -----------MFFS-ILRF---KSIQILFPILFCISIQAQDESTKIGNKVVEEESPNRI

L_alex_LEP1GSC062_3199 -----------MFFS-IRRLFQFKSIQILLPILFCISVQAQDESTKVRNKVIEEESPNRI

L_weil_LEP1GSC086_2084 -----------MFFS-IRRFFQFKSIQILFPILFCISVQAQDESTKVRNKVVEEESPNKI

L_mayo_LEP1GSC190_1091 -----------MFFS-IRRFFQLKSIQILFPILFYIPIQAQDERTKTETKVVEGESPNRI

* * : * *: ::* :.:.**:* * :. ***

L_alst_LEP1GSC193_1731 TTKENGIPPGSKKKNVEKILPNTVTPKDNDKEPPQKENGNEQNGLS--------------

L_kmet_LEP1GSC052_0212 TTKDNNTITDPPKKE------------------NGNGPNQNQNGSNIQTGSSTNAQIGQT

L_nogu_LEP1GSC059_1080 TTKDNTITTGPKKDN-----------------------GNGQNGQII-------------

L_inte_LIC20151 TT-------DSKKDN-----------------------GNGQNGQIV-------------

L_kirs_LEP1GSC049_3174 TTKENPITDDSKKDS-----------------------GNGQNGQIV-------------

L_sant_LEP1GSC048_0186 TTKNDDAIVESSEK---------------------------ENGQDP-------------

L_borg_LEP1GSC103_0620 ITKNNNTTTESLRK---------------------------ENGQNI-------------

L_alex_LEP1GSC062_3199 TTKNDNTAAESLRK---------------------------ENGQNI-------------

L_weil_LEP1GSC086_2084 TTKNDSVAAESLRK---------------------------ENGQNI-------------

L_mayo_LEP1GSC190_1091 TTKNNTTVTESLRK---------------------------ENGQNI-------------

* . . :**

L_alst_LEP1GSC193_1731 --QEDSQIVVTGSRGERRLKDSTVATEVISRKKIEASGARNAAEVLETQLGIDVVPFFGG

L_kmet_LEP1GSC052_0212 LAPEDSQIVVTGSRGERRLKDSTVATEVISRKKIEASGARNAAEVLETQLGIDVVPFFGG

L_nogu_LEP1GSC059_1080 --PDVSQIVVTGSRGERRLKDSTVATEVISRKKIEASGARNAAEVLETQLGIDVVPFFGG

L_inte_LIC20151 --PEESQIVVTGSRGERRLKDSTVSTEVISRKKIEASGARNAAEVLETQLGIDVVPFFGG

L_kirs_LEP1GSC049_3174 --PEESQIVVTGSRGERRLKDSTVATEVISRKKIEASGARNAAEVLETQLGIDVVPFFGG

L_sant_LEP1GSC048_0186 --PEESSIVVTGSRGERKLKDSTVATEVISRKKIEASGARNAAEVLETQLGIDVVPFFGG

L_borg_LEP1GSC103_0620 --PEESQIVVTGSRGERRLKDSTVATEVISRKKIEASGARNAAEVLETQLGIDVVPFFGG

L_alex_LEP1GSC062_3199 --PEESQIVVTGSRGERRLKDSTVATEVISRKKIEASGARNAAEVLETQLGIDVVPFFGG

L_weil_LEP1GSC086_2084 --PEESQIVVTGSRGERRLKDSTVATEVISRKKIEASGARNAAEVLETQLGIDVVPFFGG

L_mayo_LEP1GSC190_1091 --PEESQIVVTGSRGERRLKDSTVATEVISRKKIEASGARNAAEVLETQLGIDVVPFFGG

: *.**********.******:***********************************

L_alst_LEP1GSC193_1731 SRVRMLGLDSQYVLILIDGERISGRLNNAVDLSRFKVQNLERIEIVKGASSALYGADAIG

L_kmet_LEP1GSC052_0212 SRVRMLGLDSQYVLILIDGERISGRLNNAVDLSRFKVQNLERIEIVKGASSALYGADAIG

L_nogu_LEP1GSC059_1080 SRVRMLGLDSQYVLILIDGERISGRLNNAVDLSRFKVQNIERIEIVKGASSALYGADAIG

L_inte_LIC20151 SRVRMLGLDSQYVLILIDGERISGRLNNAVDLSRFKVQNIERIEIVKGASSALYGADAIG

L_kirs_LEP1GSC049_3174 SRVRMLGLDSQYVLILIDGERISGRLNNAVDLSRFKVQNIERIEIVKGASSALYGADAIG

L_sant_LEP1GSC048_0186 SRVRMLGLDSQYVLILIDGERIAGRLNNAVDLSRFKVQNLERIEIVKGASSALYGADAIG

L_borg_LEP1GSC103_0620 SRVRMLGLDSQYVLILIDGERIAGRLNNAVDLSRFKVQNLERIEIVKGASSALYGADAIG

L_alex_LEP1GSC062_3199 SRVRMLGLDSQYVLILIDGERIAGRLNNAVDLSRFKVQNLERIEIVKGASSALYGADAIG

L_weil_LEP1GSC086_2084 SRVRMLGLDSQYVLILIDGERIAGRLNNAVDLSRFKVQNLERIEIVKGASSALYGADAIG

L_mayo_LEP1GSC190_1091 SRVRMLGLDSQYVLILIDGERIAGRLNNAVDLSRFKVQNLERIEIVKGASSALYGADAIG

**********************:****************:********************

L_alst_LEP1GSC193_1731 GVINLITREADKKLSYEMRTTYGNGSRKNFNTEGEFNTTANMGFKNDIVSGAVSAGYNKN

L_kmet_LEP1GSC052_0212 GVINLITREADKKLSYEMRTTYGNGSRKNFNTEGEFNTTANMGFRNEMVSGAVSAGYNKN

L_nogu_LEP1GSC059_1080 GVINLITREADKKLSYEMRTTYGNGSSKNFNTEGEFNTTANMGFRNEHVSGAVSAGYNKN

L_inte_LIC20151 GVINLITREADKKLSYEMRTTYGNGSRKNFNTEGEFNTTANMGFRNEYVSGAVSAGYNKN

L_kirs_LEP1GSC049_3174 GVINLITREADKKLSYEMRTTYGNGSRKNFNTEGEFNTTANMGFRNEYVSGAVSAGYNKN

L_sant_LEP1GSC048_0186 GVINLITREADKKLSYEMRTTYGNGSRKNFNTEGEFNTNANMGFRNEFVSGSVSAGYNKN

L_borg_LEP1GSC103_0620 GVINLITREADKKLSYEMRTTYGNGSRKNFNTEGEFNTNANMGFRNEFVSGSVSSGYNKN

L_alex_LEP1GSC062_3199 GVINLITREADKKLSYEMRTTYGNGSRKNFNTEGEFNTSANMGFRNEFVSGAVSAGYNKN

L_weil_LEP1GSC086_2084 GVINLITREADKKLSYEMRTTYGNGSRKNFNTEGEFNTNANMGFRNEFVSGAVSAGYNKN

L_mayo_LEP1GSC190_1091 GVINLITREADKKLSYEMRTTYGNGSRKNFNTEGEFNTNANMGFRNEFVSGAVSAGYNKN

************************** ***********.*****.*: ***:**:*****

L_alst_LEP1GSC193_1731 PGYRLVPDSLATTGNAYQDLNTGMNLTFNPDGKFKGKTRVLYQHRDQSGVDVTQSKAVFD

L_kmet_LEP1GSC052_0212 PGYRLVPDSQATTGNAYQDLNAGMNLTFNPDGKFKGKTRILYQHRDQNGVDVTQSKAVFD

L_nogu_LEP1GSC059_1080 PGYRLVPNSQATTGNAYQDLNAGINLTFNPDGKFKGKTRILYQHRDQNGVDVTQSKAVFD

L_inte_LIC20151 PGYRLVPNSQATTGNAYQDLNTGINLTFNPDGKFKGKTRILYQHRDQNGVDVTQSKAVFD

L_kirs_LEP1GSC049_3174 PGYRLVPNSQATTGNAYQDLNTGINLTFNPDGKFKGKTRILYQHRDQNGVDVTQSKAVFD

L_sant_LEP1GSC048_0186 PGYRLVPNSQATTGNAYQDLNTGMNLTFNPDGKFKGKTRILYQHRDQNGVDVTQSKAVFD

L_borg_LEP1GSC103_0620 PGYRLVPNSQATTGNAYQDLNTGINLTFNPDGNFKGKTRILYQHRNQNGVDVTQSKAIFD

L_alex_LEP1GSC062_3199 PGYRLVSNSQATTGNAYQDLNTGMNLTFNPDGNFKGKTRILYQHRDQNGVDVTQSKATFD

L_weil_LEP1GSC086_2084 PGYRLVPNSQATTGNAYQDLNTGMNLTFNPDGNFKGKTRILYQHRDQSGVDVTQSKAIFD

L_mayo_LEP1GSC190_1091 PGYRLVPNSQATTGNAYQDLNTGINLTFNPDGNFKGKTRILYQHRDQNGVDVTQSKAIFD

******.:* ***********:*:********:******:*****:*.********* **

L_alst_LEP1GSC193_1731 RNNKTHDFLATGSLEYGFGKKNLVSFRGNISKWENKYYNNQRGSDELDVKQLNSELTSQG

L_kmet_LEP1GSC052_0212 RNNKTHDFLATGSLEYGFGKRNLLSFRGNISKWENKYYNNQRGSDELDVKQLNAELTSQG

L_nogu_LEP1GSC059_1080 RNNKTHDFLATGSLEYGFGKRNLVSFRGNISKWENKYYNNQRGSDELDVKQLNSELTSQG

L_inte_LIC20151 RNNKTHDFLATGSLEYGFGKRNLISFRGNISKWENKYYNNQRGSDELDVKQLNSELTSQG

L_kirs_LEP1GSC049_3174 RNNKTHDFLATGSLEYGFGKRNLISFRGNISKWENKYYNNQRGSDELDVKQLNSELTSQG

L_sant_LEP1GSC048_0186 RNNKTHDFLATGSLEYGFGKRNLISFRGNISKWENKYYNNQRGSDELDVKQLNAELTSQG

L_borg_LEP1GSC103_0620 RNNKTHDFLATGSLEYGFGKRNLISFRGNISKWENKYYNNQRGSDELDVKQLNAELTSQG

L_alex_LEP1GSC062_3199 RNNKTHDFLATGSLEYGFGKRNLISFRGNISKWENKYYNNQRGSDELDVKQLNAELTSQG

L_weil_LEP1GSC086_2084 RNNKTHDFLATGSLEYGFGKRNLISFRGNISKWENKYYNNQRGADELDVKQLNAELTSQG

L_mayo_LEP1GSC190_1091 RNNKTHDFLATGSLEYGFGKRNLISFRGNISKWENKYYNNQRGANELDVKQLNAELTSQG

********************.**:*******************::********:******

L_alst_LEP1GSC193_1731 TVQLDMEASERHFLTMGVESFANELESDRLQNRYVYRTRKAVFFQDEWTVSRSPRIRMIP

L_kmet_LEP1GSC052_0212 TVQLDMEASDKHFITMGAESFANELESDRLQNRYVYRTRKAAFFQDEWTISRSPRIRVIP

L_nogu_LEP1GSC059_1080 TAQLDMEASERHFITMGAENFANELESDRLQSRYVYRTRRAVFFQDEWTVSRSPRIRVVP

L_inte_LIC20151 TVQLDMEASEKHFITVGAESFANELESDRLQSRYVYRTRKAVFFQDEWTVSRSPRIRVVP

L_kirs_LEP1GSC049_3174 TVQLDMEASERHFITVGAESFANELESDRLQSRYVYRTRKAVFFQDEWTVSRSPRIRVVP

L_sant_LEP1GSC048_0186 TVQLDMEAFEKHFITIGAESFANELESDRLQSRYVYRTRKAVFLQDEWTVSSSPRIRVIP

L_borg_LEP1GSC103_0620 TVQLDMEASERHFITIGAESFANELESDRLQSRYVYRTRKAVFFQDEWIISSSPRIRMIP

L_alex_LEP1GSC062_3199 TVQLDMEASEKHFITIGAESFANELESDRLQSRYVYRTRKAVFFQDEWTVSSSPRIRVIP

L_weil_LEP1GSC086_2084 TVQLDMEASEKHFITIGAESFANELESDRLQSRYVYRTRKAVFFQDEWTVSTSPRIRVIP

L_mayo_LEP1GSC190_1091 TVQLDMEASERHFITIGAESFANELESDRLQSRYVYRTRKAVFFQDEWTVSSSPRIRVIP

*.****** :.**:*:*.*.***********.*******.*.*:**** :* *****::*

L_alst_LEP1GSC193_1731 GVRYDDDSQFGNQTTPKLAVRYDIFQSLVWRTSYGRGFRPPSFQELYLRFENPAVGYVVE

L_kmet_LEP1GSC052_0212 GVRYDDDSQFGNQTTPKLAARYDILQNLVWRASYGRGFRPPSFQELYLRFENPAVGYVVE

L_nogu_LEP1GSC059_1080 GVRYDDDSQFGNQTTPKLAARYDIFQNLVWRASYGRGFRPPSFQELYLRFENPAVGYVVE

L_inte_LIC20151 GVRYDDDSQFGNQTTPKLAARYDIFQNLVWRASYGRGFRPPSFQELYLRFENPAVGYVVE

L_kirs_LEP1GSC049_3174 GVRYDDDSQFGNQTTPKLAARYDIFQNLVWRASYGRGFRPPSFQELYLRFENPAVGYVVE

L_sant_LEP1GSC048_0186 GLRYDDDSQFGNQTTPKFAVRYDIFQNLVWRTSYGRGFRPPSFQELYLRFENPAVGYVVE

L_borg_LEP1GSC103_0620 GVRYDDDSQFGNQTTPKLAVRYDIFQNLVWRASYGRGFRPPSFQELYLRFENPAVGYVVE

L_alex_LEP1GSC062_3199 GVRYDDDSQFGNQTTPKLAVRYDIFQNLVWRTSYGRGFRPPSFQELYLRFENPAVGYVVE

L_weil_LEP1GSC086_2084 GVRYDDDSQFGNQTTPKLAARYDIFQNLVWRTSYGRGFRPPSFQELYLRFENPAVGYVVE

L_mayo_LEP1GSC190_1091 GVRYDDDSQFGNQTTPKLAVRYDIFQNLVWRTSYGRGFRPPSFQELYLRFENPAVGYVVE

*:***************:*.****:*.****:****************************

L_alst_LEP1GSC193_1731 GNPNLKPERSITINSDLEYSPFSFLTFSFSVYRNDIINLIQYKFDSNRGREFAEFQLQNV

L_kmet_LEP1GSC052_0212 GNPNLRPEKSITINSDLEYSPFSFLTFSFSLYRNDIINLIQYKFDSNKGREFAEFQLQNI

L_nogu_LEP1GSC059_1080 GNPNLKPERSITINSDLEYSPFNFLTFSLSVYRNDIINLIQYKFDSNKGREFAEFQLRNI

L_inte_LIC20151 GNPNLKPERSITINSDLEYSPFSFLTFSLSVYRNDIINLIQYKFDSNKGREFAEFQLQNI

L_kirs_LEP1GSC049_3174 GNPNLKPERSITINSDLEYSPFSFLTFSLSVYRNDIINLIQYKFDSNKGREFAEFQLQNI

L_sant_LEP1GSC048_0186 GNPNLKPERSSTINSDLEYSPFNFLTFSFSLYRNDIVNLIQYKFDSSKGKEFAEFQLQNV

L_borg_LEP1GSC103_0620 GNPNLKPERSITINSDLEYSPFSFLTLSLSLYRNDIINLIQYKFDSNKGKEFAEFQLQNI

L_alex_LEP1GSC062_3199 GNPNLKPERSITINSDLEYSPFSFLTFSLSLYRNDIINLIQYKFDSNKGKEFAEFQLQNI

L_weil_LEP1GSC086_2084 GNPNLKPERSITINSDLEYSPFRFLTFSLSLYRNDIINLIQYKFDSNKGKEFAEFQLQNI

L_mayo_LEP1GSC190_1091 GNPNLKPERSITINSDLEYSPFSFLTFSLSLYRNDIINLIQYKFDSNKGKEFAEFQLQNI

*****.**.* *********** ***:*:*:*****:*********..*.*******.*:

L_alst_LEP1GSC193_1731 AKAYTRGGEFGVHYRFLKYFTLELGYNHTDTRDLTTDRPLEGRALHQASANFIYNSPGGF

L_kmet_LEP1GSC052_0212 AKAYTRGGEFGVQYRFLKHFTLELGYNHTDTRDETTNRPLEGRALHQASANFIYNSPSGF

L_nogu_LEP1GSC059_1080 AKAYTRGGEFGVQYRFLKYFTLELGYNHTDTRDLSSDRPLEGRALHQASANFIYNSPGGF

L_inte_LIC20151 AKAYTRGGEFGVQYRFLKYFTLELGYNHTDTRDLSSDRPLEGRALHQASANFIYNSPGGF

L_kirs_LEP1GSC049_3174 AKAYTRGGEFGVQYRFLKYFTLELGYNHTDTRDLSSNRPLEGRALHQASANFIYNSPGGF

L_sant_LEP1GSC048_0186 AKAYTRGGEFGIQYRFLKYFTLELGYNHTDTRDLSTDRPLEGRALHQASANFIYNSPGGF

L_borg_LEP1GSC103_0620 AKAYTRGGEFGVQYRFLKYFTLELGYNHTDTRDLNTDRPLEGRALHQASANFIYNSPGGF

L_alex_LEP1GSC062_3199 AKAYTRGGEFGVQYKFLKYFTLELGYNHTDTRDLNTDRPLEGRALHQASANFIYNSPGGF

L_weil_LEP1GSC086_2084 AKAYTRGGEFGVQYKFLKYFTLELGYNHTDTRDLNTDRPLEGRALHQASANFIYNSPGGF

L_mayo_LEP1GSC190_1091 AKAYTRGGEFGVQYRFLKYFTLELGYNHTDTRDLNTDRPLEGRALHQASANFIYTSPGGF

***********::*.***:************** .::*****************.**.**

L_alst_LEP1GSC193_1731 QFNLRGKHLDKRPFYSSTNNLSASGQDYIPTEVKLNENPPVIYGKPFTILNVRIEQKFFD

L_kmet_LEP1GSC052_0212 QFNLRGKHLDKRPFYSSTNNLSAAGQDYIPTEVKLNENPPVTYGKPFTIINIRMEQKFFD

L_nogu_LEP1GSC059_1080 QFNLRGKHLDKRPFYSSTNNLSAAGQDYIPSEVKLNENPPVIYGKPFTILNVRIEQKFFN

L_inte_LIC20151 QFNLRGKHLDKRPFYSSTNNLSAAGQDYIPSEVKLNENPPVIYGKPFTILNVRIEQKFFN

L_kirs_LEP1GSC049_3174 QFNLRGKHLDKRPFYSSTNNLSAAGQDYIPSEVKLNENPPVIYGKPFTILNVRIEQKFFN

L_sant_LEP1GSC048_0186 QFNLRGKHLDKRPFYSSTNNLSAAGQDYIPTEVKLNEKPPVIYGKPFTILNVRIEQKFFD

L_borg_LEP1GSC103_0620 QFNLRGKHLDKRPFYSATNNL--AGQDSIPTEVKLNENPPVIYGKPFSILNVRIEQKFFN

L_alex_LEP1GSC062_3199 QFNLRGKHLDKRPFYSATNNLSAAGQDYIPTEVKLNENPPVIYGKPFTILNVRIEQKFFN

L_weil_LEP1GSC086_2084 QFNLRGKHLDKRPFYSATNNL--GGQDYIPTEVKLNENPPVIYGKPFTILNVRIEQKFFN

L_mayo_LEP1GSC190_1091 QFNLRGKHLDKRPFYSATNNL--AGQDYIPTEVKLNENPPVIYGKPFTILNVRIEQKFFN

****************:**** .*** **:******:*** *****:*:*:*:*****:

L_alst_LEP1GSC193_1731 KHFALFLGVDNVLNQYELAYNPTRPRFYYGGFSAQF

L_kmet_LEP1GSC052_0212 KHFGLFVGVDNVLNQYELAYNPTRPRFYYGGFSAQF

L_nogu_LEP1GSC059_1080 KHFALFLGVDNLLNQYELAYNPTRPRFYYGGFSAQF

L_inte_LIC20151 KHFALFLGVDNLLNQYELAYNPTRPRFYYGGFSAQF

L_kirs_LEP1GSC049_3174 KHFALFLGIDNLLNQYELAYNPTRPRFYYGGFSAQF

L_sant_LEP1GSC048_0186 NRFSLFLGVDNVLNQYELAYNPTRPRFYYGGFSSQF

L_borg_LEP1GSC103_0620 KHFSLFLGVDNVLNQYELTYNPIRPRFYYGGFSAQF

L_alex_LEP1GSC062_3199 KHFSLFLGVDNVLNQYELAYNPTRPRFYYGGFSAQF

L_weil_LEP1GSC086_2084 KHFSLFLGVDNVLNQYELAYNPTRPRFYYGGFSAQF

L_mayo_LEP1GSC190_1091 KHFSLFLGVDNVLNQYELAYNPTRPRFYYGGFSAQF

:.*.**:*:**:******:*** **********:**
