# Supplementary material for: Discovery of Novel Leptospirosis Vaccine Candidates Using Reverse and Structural Vaccinology
Source: Front Immunol. 2017 Apr 27;8:463. doi: 10.3389/fimmu.2017.00463 (PMC5406399; doi:10.3389/fimmu.2017.00463)
Supplement: Supplementary file 9 [file Data_Sheet_2.DOCX]

**CoVIRA: Proof of concept for signal peptide prediction**

***Datasets***

Datasets of curated bacterial proteins with and without signal peptides, name “Positive (Full)” and “Negative (Full)”, were download from Uniprot-Swissprot using the keywords showed in Table SDS1. From each dataset, 1000 proteins were randomly selected and named “Positive (Selected)” and “Negative (Selected)”.

**Table SDS1.** Overview of the datasets of proteins obtained from Uniprot-Swissprot with and without signal peptide

| **Dataset** | **Number of entries** | **Keywords** |
| --- | --- | --- |
| Positive (Full) | 2,108 | keyword:"Cell outer membrane [KW-0998]" keyword:"Signal [KW-0732]" taxonomy:bacteria AND reviewed:yes |
| Negative (Full) | 322,978 | NOT keyword:"Cell outer membrane [KW-0998]" NOT keyword:"Signal [KW-0732]" taxonomy:bacteria AND reviewed:yes |

***Signal peptide prediction***

Signal peptide prediction was performed using SignalP, PreDiSi and SignalCF. The results were analyzed using a naïve voting was performed by applying an equal weight (0.333…) to each predictor. Based on the same results, CoVIRA was executed to calculate a final score and prediction for each protein.

***ROC analysis***

A Receiver operating characteristic (ROC) curve was generated using the Scikit-learn package in Python. The final predictions based on naïve voting and CoVIRA were compared to the original annotation of each protein. As demonstrated by the Area Under the Curve (AUC) metric, CoVIRA showed a slightly improved result compared to the prediction based on naïve voting.

**
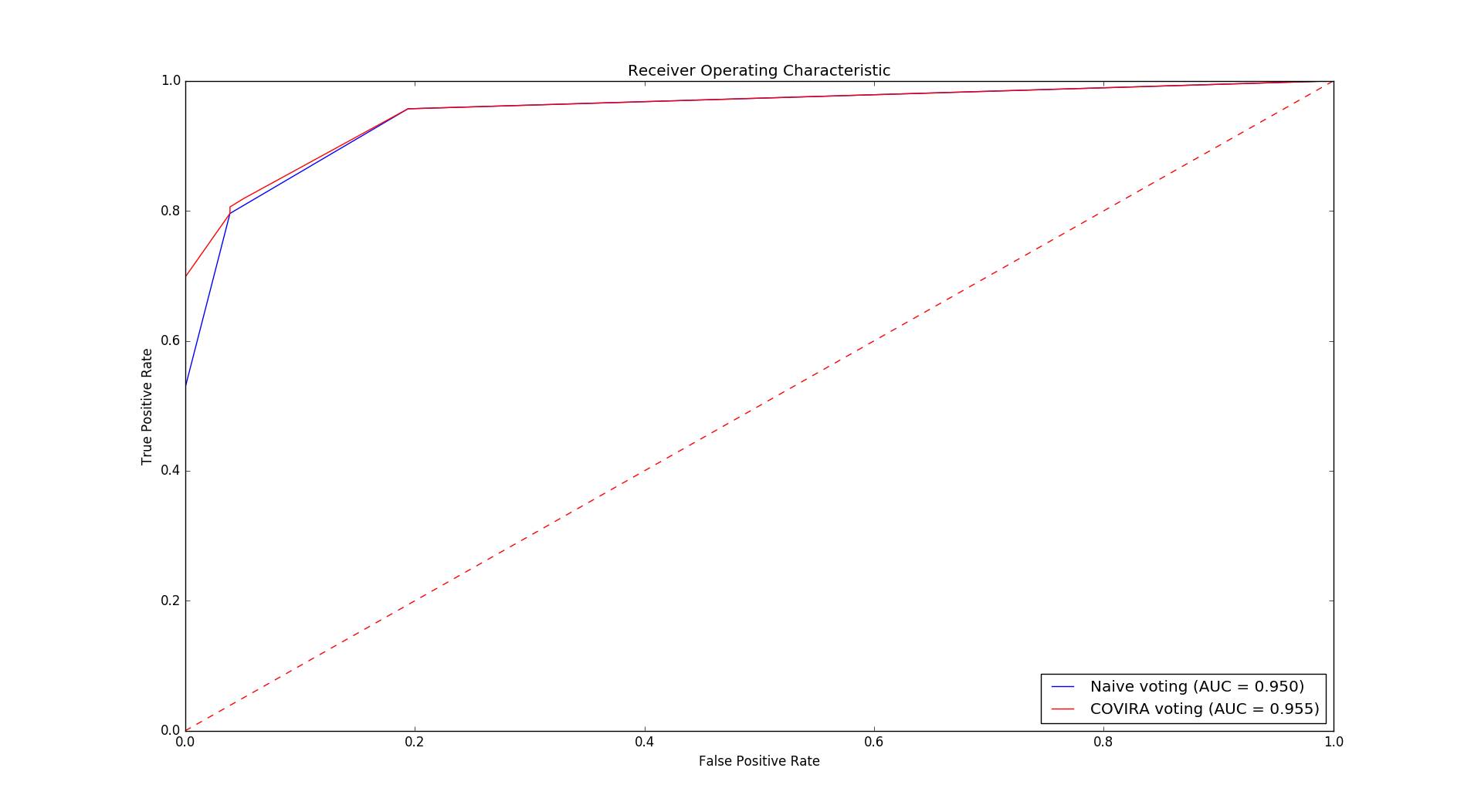
**

**Figure 1.** Receiver operating characteristic (ROC) curve comparing the results generated by naïve voting and CoVIRA for a consensus prediction of proteins with signal peptides.
