# Supplementary material for: Discovery of Novel Leptospirosis Vaccine Candidates Using Reverse and Structural Vaccinology
Source: Front Immunol. 2017 Apr 27;8:463. doi: 10.3389/fimmu.2017.00463 (PMC5406399; doi:10.3389/fimmu.2017.00463)
Supplement: Supplementary file 10 [file Data_Sheet_3.PDF]

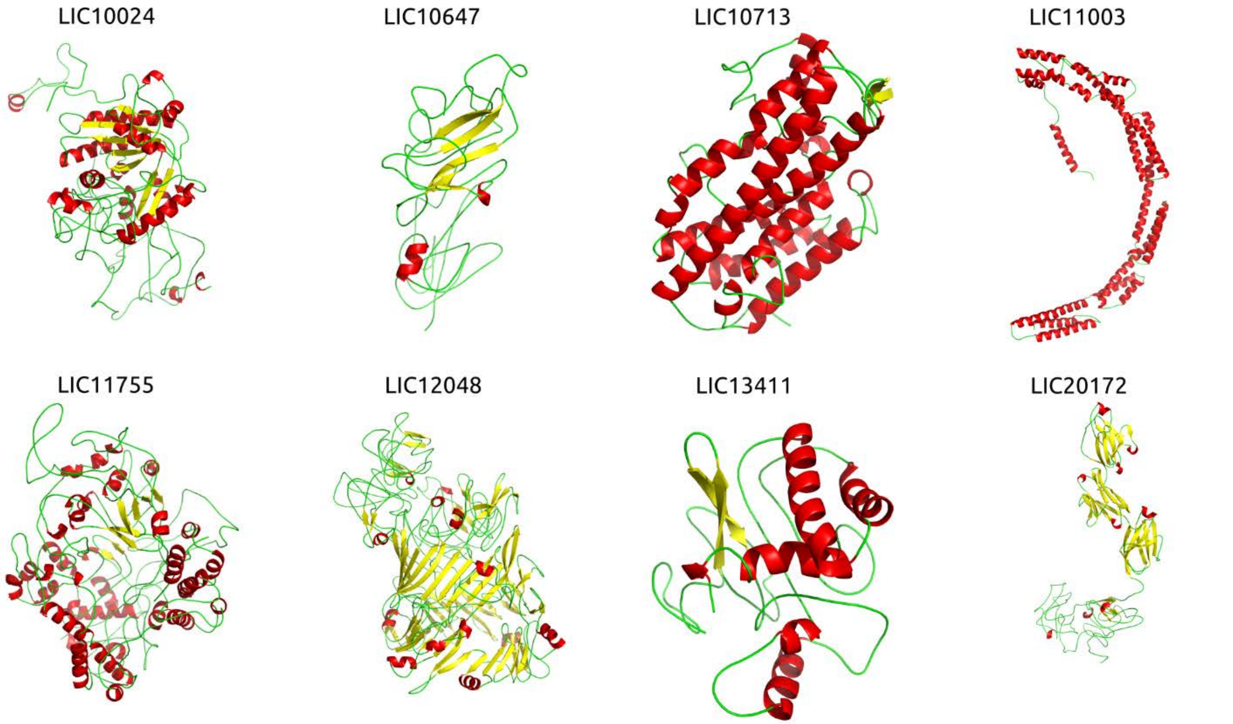

Supplementary Figure S1. 3D structures predicted for the OM lipoproteins selected in this work. The structure modelling was performed using I-TASSER and the structures were visualized and the images generated using PyMOL.
